# Radical C−N Borylation of Aromatic Amines Enabled by a Pyrylium Reagent

**DOI:** 10.1002/chem.202000412

**Published:** 2020-03-09

**Authors:** Yuanhong Ma, Yue Pang, Sonia Chabbra, Edward J. Reijerse, Alexander Schnegg, Jan Niski, Markus Leutzsch, Josep Cornella

**Affiliations:** ^1^ Max-Planck-Institut für Kohlenforschung Kaiser-Wilhelm-Platz 1 Mülheim an der Ruhr 45470 Germany; ^2^ Max-Planck-Institut für Chemische Energiekonversion Stiftstrasse 34–36 Mülheim an der Ruhr 45470 Germany

**Keywords:** aromatic amine, C−N bond functionalization, pyridinium salts, pyrylium, radical borylation

## Abstract

Herein, we report a radical borylation of aromatic amines through a homolytic C(sp^2^)−N bond cleavage. This method capitalizes on a simple and mild activation via a pyrylium reagent (^Sc^Pyry‐OTf) thus priming the amino group for reactivity. The combination of terpyridine and a diboron reagent triggers a radical reaction which cleaves the C(sp^2^)−N bond and forges a new C(sp^2^)−B bond. The unique non‐planar structure of the pyridinium intermediate, provides the necessary driving force for the aryl radical formation. The method permits borylation of a wide variety of aromatic amines indistinctively of the electronic environment.

## Graphical abstract

**A radical borylation** of aromatic amines through a homolytic C(sp^2^)−N bond cleavage is presented. This method capitalizes on a simple and mild activation via a pyrylium reagent (^Sc^Pyry‐OTf) that primes the amino group for reactivity. The combination of terpyridine and a diboron reagent triggers a radical reaction which cleaves the C(sp^2^)−N bond and forges a new C(sp^2^)−B bond. The non‐planar structure of the pyridinium, provides the necessary driving force for the challenging aryl radical formation.

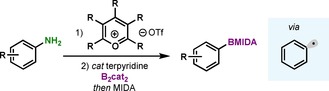

Primary aromatic amines represent a class of relevant functionalities present in a wide variety of contexts — from natural sources such as DNA or vitamins to synthetic molecules as part of their structure.^1^ Despite their potential as anchor points for further manipulation, direct functionalization of primary amino groups in (hetero)aromatic compounds has been a tremendous challenge in catalysis^2^ due to high energy of the C(sp^2^)−NH_2_ bonds (BDE of C_6_H_5_–NH_2_: 102.6±1.0 kcal mol^−1^),^3^ coordination of the lone pair of the nitrogen to metal catalysts, and acid‐base interactions with polar functionalities. To circumvent such drawbacks, approaches to cleave C−N bonds have relied on the preactivation of the amino group, converting them into virtuous leaving groups, for example by diazotization,^4^ polyalkylation^5^ and others^6^ (Figure 1 A). However, despite the wealth of reports in this area, several challenges remain. For example, diazotization reactions require the use of strong oxidants and acids, to generate the corresponding diazonium salts, which are thermally unstable and explosive (Figure 1 A, *path a*).^4^ The use of an excess of toxic alkylating reagents restricts the functional group tolerance in complex settings for polyalkylation strategies (Figure 1 A, *path b)*.^*[*5]^ Although limited in functional group tolerance and scope, approaches based on transition metals have recently appeared, enabling the cleavage and functionalization of aniline derivatives (Figure 1, *path c*).^6^

**Figure 1:**
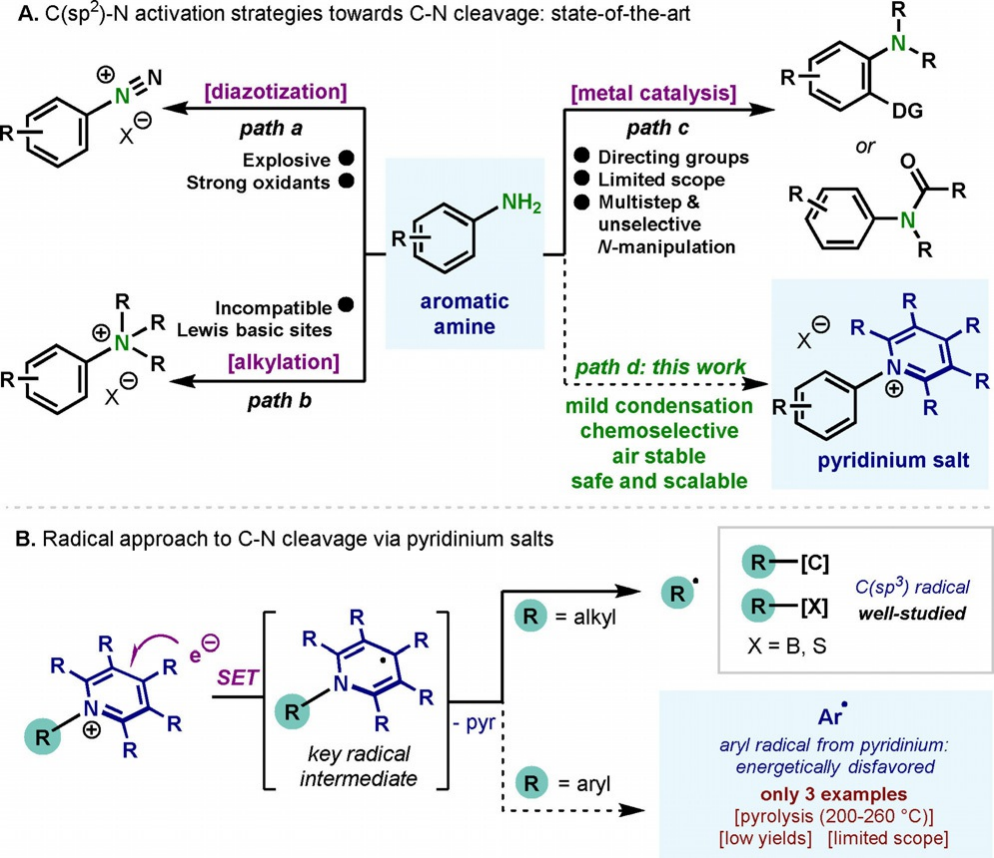
A) Strategies to prime the amino group for reactivity. B) Generation of aromatic radicals is a thermodynamically disfavored process.

Seminal work by Katritzky demonstrated the possibility of converting amino groups into good leaving groups by condensation with a pyrylium salt (Figure 1 A, *path d*).^7^ This strategy is characterized by the remarkable stability of the pyridinium salt intermediates, high selectivity for the amino groups and benefits from the high practicality and simplicity. Indeed, pyridinium salts have recently been employed to unlock SET processes based on transition‐metal or photoredox catalysis, and have been shown to be a powerful tool for constructing a myriad of chemical bonds.^8^, ^9^, ^10^, ^11^ However, the wealth of literature in this area has been focused on the generation of alkyl radicals (Figure 1 B, top). Yet, methods which capitalize on pyridinium salts to generate aryl radicals through SET are largely underdeveloped. (Figure 1 B, bottom),^12^ mainly due to the disfavored thermodynamics for the aryl radical formation. Although examples of this approach have been reported in the past (3 examples),^12^ pyrolysis of the reagents is required (ca. 200 °C), obtaining low yields for very specific substrates, thus relegating these approaches to proof of concept examples with limited synthetic applicability. Based on our recent interest on pyrylium reagents,^13^ we set out to explore this approach in the context of radical borylations using diboron reagents, as they have been shown to be excellent radical acceptors.^14^, ^15^, ^16^, ^17^ Herein, we report a protocol for the borylation of (hetero)aromatic amines through a SET process, enabled by the use of a tethered pyrylium salt (^Sc^Pyry‐OTf).^18^ The structure of this pyrylium reagent proved unique in assisting the cleavage of the C(sp^2^)−N bond, a feature beyond the capabilities of other common pyrylium activators. Moreover, the choice of the solvent was also crucial to achieve high yields of the corresponding organoboron compounds. The protocol has been demonstrated to be scalable and tolerant to a wide variety of functionalities.

Based on recent reports on the borylation of alkyl pyrydinium salts,^11^ we started our investigations on the borylation of pyridinium salts using B_2_cat_2_ (bis(catecholato)diboron). After screening of the reaction parameters, terpyridine (terpy) was identified as the Lewis‐base of choice, performing the reaction at 130 °C, using ^*i*^Pr_2_NC(O)Me as solvent.^19^ Interestingly, under the optimized conditions, none of the classical pyridinium salts commonly employed proved efficient in the borylation reaction (Table 1 A, **1**–**3**). Then, we turned our attention to the tethered pyrylium reagent initially reported by Katritzky in the context of alkyl amine activation.^20^ It was the pyridinium **4‐OTf** that delivered excellent yields of C−B bond formation **5** (Table 1 A, entry 1, 82 %). When the counterion in **4** was replaced by BF_4_ (**4‐BF_4_**), a lower yield was obtained (57 %). The effect of the solvent was also remarkable: whereas DMF and DMAc failed to deliver good yields of product (entries 2 and 3), the use of a more sterically hindered amide such as ^*i*^Pr_2_NC(O)Me proved to be crucial for obtaining high yields. Although in the absence of Lewis‐base the reaction afforded only 10 % of **5** (entry 4), the use of bipyridine derivatives did not reach the levels of reactivity of terpy (entries 5 and 6). Although borylation strategies based on B_2_pin_2_ and aromatic Lewis bases have recently appeared in the literature,16b the use of this diboron reagent resulted in no conversion of **4‐OTf** (entry 7). Heating the reaction further had no effect on the reactivity (entry 8) and 120 °C proved insufficient to obtain high yields of **5** (entry 9). Isolation of **5** proceeded through the conversion of the sensitive Ar−B(cat) into the corresponding Ar−Bpin reagent, by a simple quench with pinacol and Et_3_N. However, a quenching protocol based on MIDA resulted in slightly higher yields and afforded a more robust organoboron compound (**6**).^21^, ^22^ Of note, the synthesis of the ^Sc^Pyry‐OTf (**7**) could be conducted similarly to the parent 2,4,6‐triphenylpyrylium reagent.20a Commercially available tetralone ($0.26 g^−1^),^23^ condenses with benzaldehyde, which upon addition of TfOH, pure **7** precipitates as a bright‐yellow solid. The protocol could be scaled‐up to >30 grams in one run, without any complicated setup (Table 1 B).

**Table 1:** Optimization of the borylation of arylpyridinium salts.^[a]^

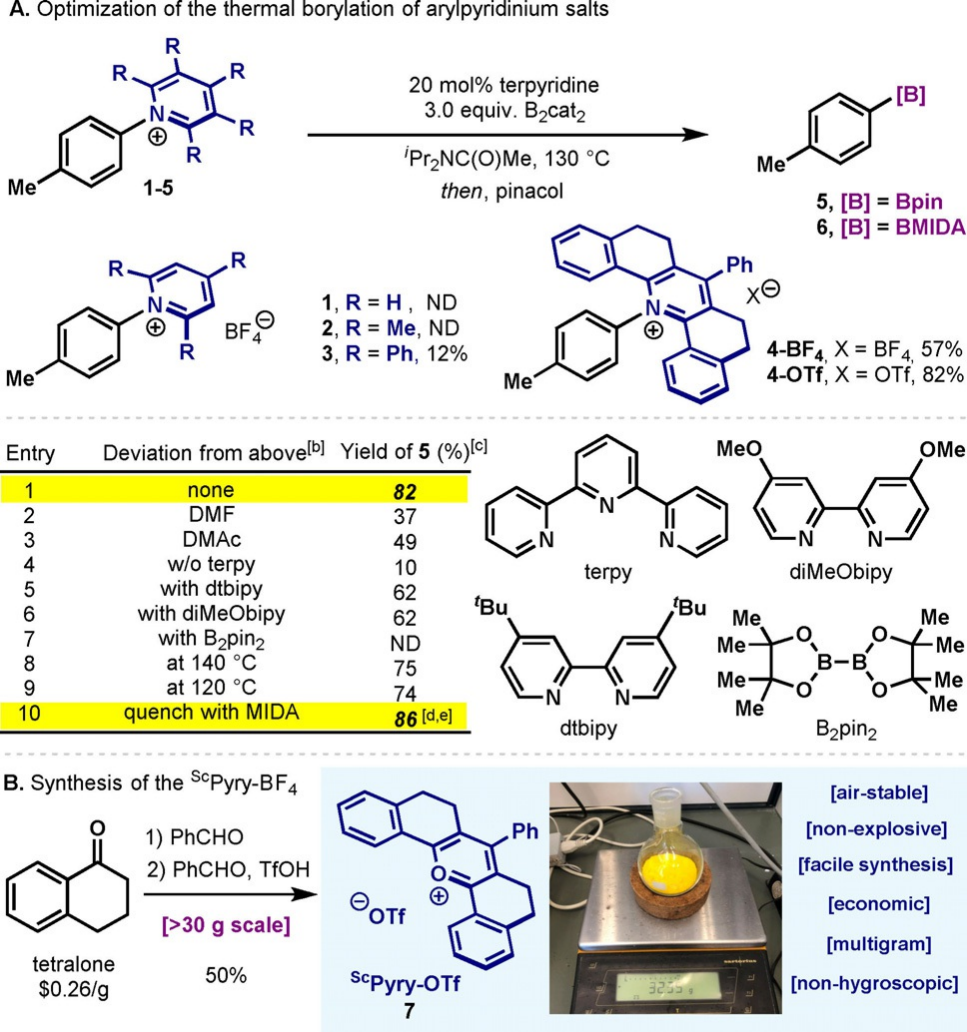

[a] **1**–**4** (0.1 mmol), B_2_cat_2_ (0.3 mmol), terpy (20 mol %), ^*i*^Pr_2_NC(O)Me (0.5 mL) at 130 °C for 24 h; then pinacol (0.6 mmol) and Et_3_N (0.5 mL) were added and stirred for additional 2 h at 25 °C. [b] Using **4‐OTf** as starting material. [c] Yields determined by GC using *n*‐dodecane as internal standard. [d] Yield of isolated product **6**. [e] Reaction performed at 0.25 mmol for 12 h, then MIDA (1.5 mmol) for 4 h at 90 °C. MIDA=*N*‐methyliminodiacetic acid. ND=not detected.

With the optimal protocol in hand, we explored the scope of this new borylation strategy. It is worth noting that condensation of aromatic amines with **7** proceeded smoothly across the whole range of substrates (**8**–**32**) with an average yield of >85 %.^19^ As shown in Table 2 A, the borylation protocol boded well with anilines substituted at the *meta*‐ (**33**) and *para*‐positions (**34**, **35**). The presence of electron‐deficient fluorinated moieties such as CF_3_ (**36**), OCF_3_ (**37**) or F (**38**, **39**) did not affect the reactivity and provided good yields of product. The reaction could also be performed in a one‐pot fashion as exemplified by **39**; albeit in moderate yield.

**Table 2:** Scope of the radical borylation of (hetero)aromatic amines enabled by the pyrylium salt **7**.^[a]^

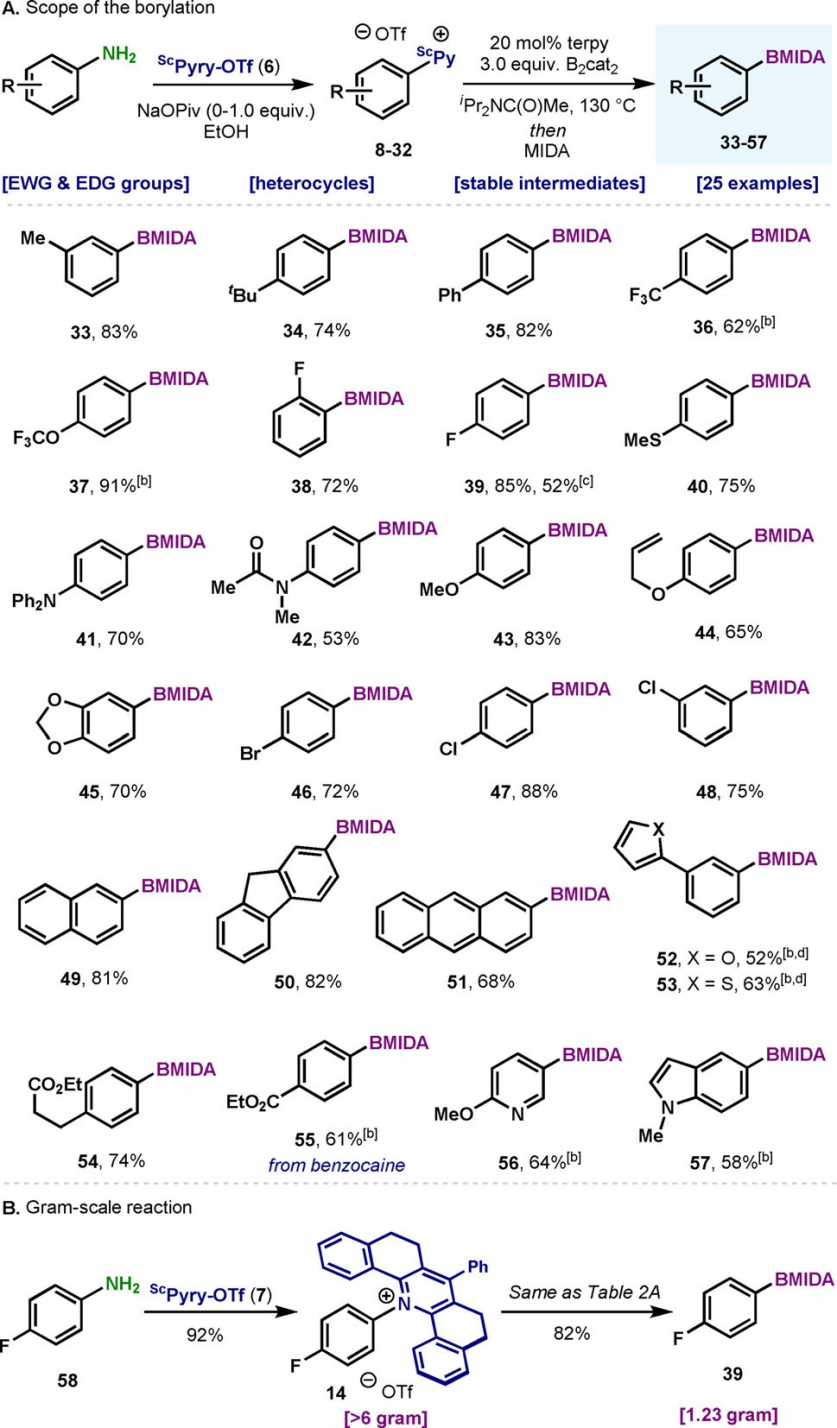

[a] Reaction conditions: Step 1: aromatic amine (1.05–1.50 equiv), **7** (1.0 equiv), NaOPiv (0–1.0 equiv) in EtOH (0.2 m) at 85 °C; Step 2: pyridinium salt (0.25 mmol), B_2_cat_2_ (0.75 mmol), terpyridine (20 mol %) in ^*i*^Pr_2_NC(O)Me (0.2 m) at 130 °C for 12 h; then MIDA (1.5 mmol) at 90 °C for 4 h. Isolated yields for the borylation step. [b] B_2_cat_2_ (1.0 mmol) was used and 24 h reaction time. [c] Yield from aniline, without isolating the pyridinium intermediate (in situ). [d] ^*i*^Pr_2_NC(O)Me (0.125 m).

Electron‐releasing substituents in the aniline were also amenable, as exemplified by the presence of thioethers (**40**), tertiary amines (**41**), amides (**42**) and ethers (**43**–**45**). Notably, no Claisen rearrangement by‐products were observed for product **44**. Bromo‐ (**46**) and chloroanilines (**47**, **48**) were also compatible under the reaction conditions, thus providing boronic acid derivatives bearing orthogonal handles for further derivatization. Boronic acid derivatives of π‐extended anilines such as naphthyl (**49**), fluorenyl (**50**) or anthracenyl (**51**) could also be synthesized in high yields. The presence of oxygen‐ (**52**) or sulfur‐containing heterocycles (**53**) did not affect the formation of the C−B bond. Anilines bearing aliphatic esters (**54**) or a benzoate motif, such as the anesthetic drug benzocaine, could also be borylated (**55**) in good yields. Finally, heterocyclic N‐containing compounds such as pyridine (**56**) and indole (**57**) were amenable for borylation under the optimal conditions. As depicted in Table 2 B, both the condensation and the borylation proved to be highly scalable, as demonstrated by the >6 grams of pyrylium **14** provided and the 1.23 grams of borylated compound **39**.

At this point, we set out to explore the remarkable effects for both the solvent and the structure of the pyrylium. As shown in Table 1, when **3** was subjected to the optimized conditions using DMAc, no borylation was obtained and >90 % of starting pyridinium salt **3** was recovered (Figure 2 A). When ^*i*^Pr_2_NC(O)Me was used instead, a minimal yield of **5** was obtained (12 %). However, the conversion was low and the reaction was plagued with several unidentified by‐products. In stark contrast, when **4‐OTf** was subjected to the borylation conditions in DMAc, acceptable yields of borylation were obtained (49 %, Table 1, entries 3). Analysis of the reaction mixture revealed the formation of a major by‐product, which was identified as the reduced compound **59**.^24^ Gratifyingly, when the solvent was replaced by the optimal ^*i*^Pr_2_NC(O)Me, formation of by‐product **59** was suppressed (<5 %), and excellent yields of **5** were obtained (82 %, Table 1, entry 1). As suggested by Katritzky's report,12c **59** possibly resulted from HAT from solvent molecules.^24^

**Figure 2:**
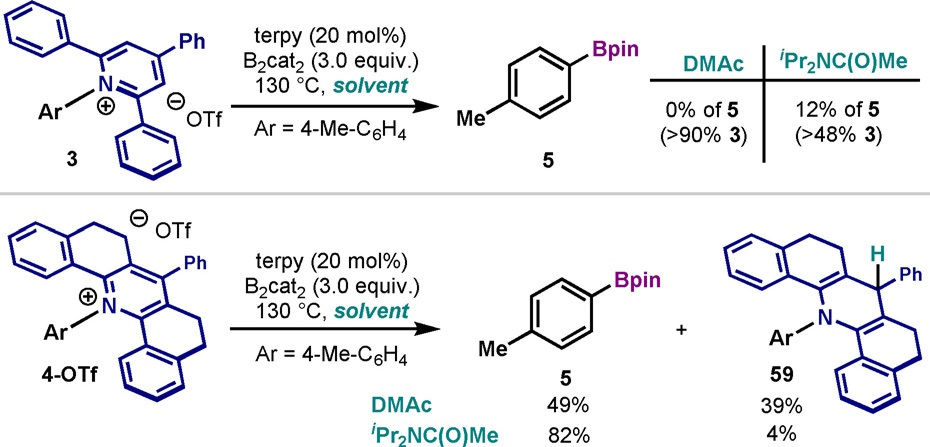
Reactivity of pyridinium salts **3** and **4‐OTf** with different solvents.

Motivated by the striking differences in reactivity between **3** and **4‐OTf**, we initially interrogated their electronic properties. Cyclic voltammetry experiments conducted in both compounds revealed a reversible behavior and a similar first reduction potential (*E*_red_ (**3**)=−1.39 V vs. Fc/Fc^+^ in DMF, *E*_red_ (**4‐OTf**)=−1.30 V vs. Fc/Fc^+^ in DMF).^19^ This result suggests that the oxidation capabilities of both pyridinium salts are similar, and reduction through SET processes should be equally facile using the terpy/B_2_cat_2_ system.11b, 25 However, X‐ray analysis of the crystal structure for **3** and **4‐BF_4_** was far more revealing. The pyridine moiety in **3** is planar, with almost no torsion observed in the pyridinium ring (Figure 3 A, left). On the other hand, the ethane‐bridged moiety in **4‐BF_4_**, renders a much more constraint environment and results in a heavily tensioned aromatic pyridinium motif, ^[20c]^ as judged by the remarkable 11.2°, −174.4° and 5.8° of torsion for the three different angles explored (Figure 3 A, right). Based on all the experimental data, a putative mechanism for this transformation is depicted in Figure 3 B. In an initiation phase, the combination of B_2_cat_2_, terpy and amide solvent affords the highly reducing **int‐2** radical species, as suggested in previous Lewis‐base‐promoted borylation strategies (Figure 3 B).11a **Int‐2** would then engage in the reduction of the pyridinium moiety to generate **int‐1**. The high degree of distortion of the aromatic ring in **4‐OTf** led us to postulate that **int‐1** would be highly unstable, and homolytic C−N cleavage occurs at high temperatures. We speculate that the restoration of the planarity renders a higher degree of conjugation and aromaticity for the leaving pyridine **61** 26 and provides the necessary driving force for the homolysis of the C−N bond. As aforementioned, when DMAc was used competing formation of **59** occurs. Yet, the use of ^*i*^Pr_2_NC(O)Me circumvents HAT, and aryl radical formation through C−N scission is largely operative. Although the nature of this difference in reactivity is still under investigation, we propose that the success of this solvent hinges on providing an adequate balance for a successful radical chain towards productive radical borylation. The aryl radical formed, is then rapidly trapped by B_2_cat_2_, delivering the desired C−B bond, with concomitant generation of the reducing solvent‐ligated boron radical **int‐2**.16g

**Figure 3:**
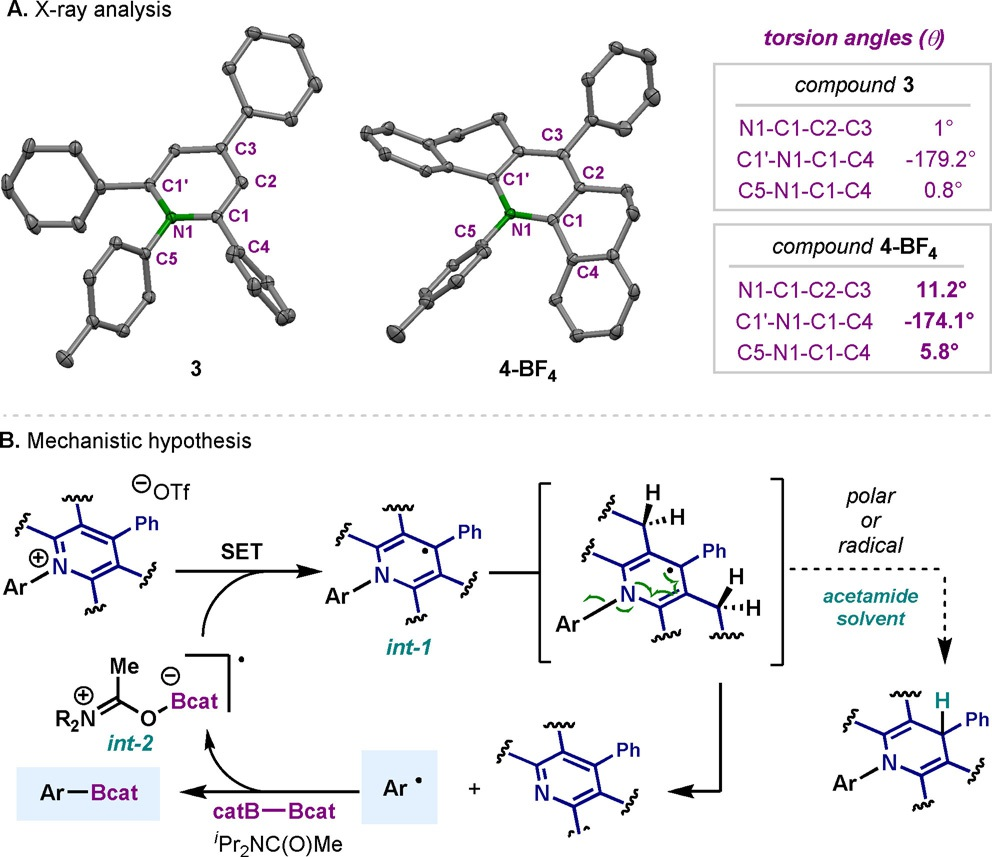
A) X‐ray structures of **3** and **4‐BF_4_**. Counterions are omitted for clarity. B) Putative mechanism for the catalytic borylation reaction of pyridinium salts.

The involvement of radical intermediates in the reaction was verified by continuous wave (CW) electron paramagnetic resonance (EPR) experiments. Sample 1 (Figure 4 A‐1) was extracted from the reaction mixture of **4‐OTf** with B_2_cat_2_ and terpy in DMAc. According to the proposed mechanism (Figure 3 B), two long‐lived radical intermediates can occur: the solvent‐ligated boron radical **int‐2** as well as **int‐1** formed from the SET reduction of **4‐OTf. Int‐2** would be boron‐centered whereas the latter is expected to be carbon centered. The dominant boron isotope ^11^B (80 %) has nuclear spin *I*=3/2, potentially giving rise to a quartet hyperfine pattern in the EPR. Carbon, on the other hand, has no dominant isotope with nuclear spin (^13^C with *I*=1/2 has 1.1 % natural abundance). Therefore, no strong hyperfine interaction (HFI) pattern is expected for **int‐1**. The two proposed radical intermediates were separately generated. The **int‐2** was expected in a mixture of B_2_cat_2_ with terpy in DMAc, that is, the reaction mixture without **4‐OTf** (Figure 4 A‐2). Indeed, the EPR spectrum showed well resolved hyperfine lines but more than a mere quartet. Possibly, also ^1^H and/or ^14^N hyperfine interactions contribute to this multiline (12) pattern. In any case, sample 1 showed an EPR spectrum virtually identical to that of the sample 2. In contrast, sample 3 which was generated by direct reduction of **4‐OTf** with TDAE, showed a strong EPR signal with weak HFI features containing very small splitting not resembling the EPR spectrum of sample 1. It therefore can be concluded that the reaction mixture is dominated by the species **int‐2** generated from sample 2, which is consistent with **int‐2**. In addition, performing the borylation reaction in the presence of 1,1‐diphenylethene resulted in the formation of **5** (53 %) and the radical addition product **60** (22 %) (Figure 4 B). This result offers additional evidence for the homolytic cleavage of the C−N bond and the generation of aryl radicals in solution.

**Figure 4:**
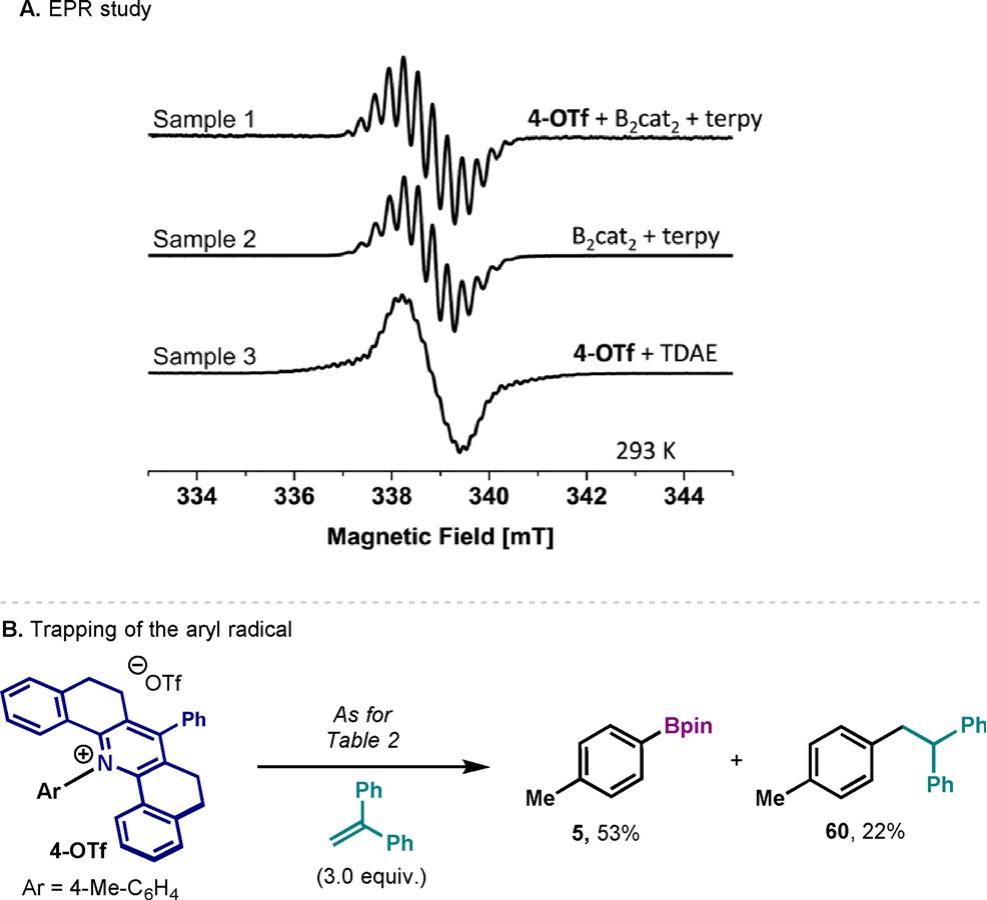
A) Room temperature CW EPR spectrum (9.5 GHz) of a reaction mixture containing B_2_cat_2_ and terpy in DMAc 1) with and 2) without **4‐OTf**, respectively. 3) reduction of **4‐OTf** substrate by addition of TDAE in DMAc. TDAE=tetrakis(dimethylamino)ethylene. All the spectra were recorded under identical conditions with 10 mW microwave power, 100 KHz modulation frequency and 0.1 mT modulation amplitude. See supporting information for details. B) Radical trap experiments confirming the presence of aryl radicals.

In summary, we have developed a novel strategy for the C−N borylation of aromatic amines, capitalizing on the mild and selective condensation of ^Sc^Pyry‐OTf (**7**) with amino groups. Additionally, the rationally designed solvent permits the smooth generation of highly reactive aryl radicals to engage in a C−B bond forming event. The borylation protocol is demonstrated to be scalable and is tolerant to various functional groups. The ability of pyridinium salts derived from ^Sc^Pyry‐OTf (**7**) to successfully generate aryl radicals, represents a new approach in the area of C−N functionalization. Research exploiting ^Sc^Pyry‐OTf (**7**) for other applications in organic synthesis is currently ongoing in our research laboratories.

## Conflict of interest

The authors declare no conflict of interest.

## Supporting information

As a service to our authors and readers, this journal provides supporting information supplied by the authors. Such materials are peer reviewed and may be re‐organized for online delivery, but are not copy‐edited or typeset. Technical support issues arising from supporting information (other than missing files) should be addressed to the authors.

Supplementary
