# Supplementary material for: Radical C−N Borylation of Aromatic Amines Enabled by a Pyrylium Reagent
Source: Chemistry. 2020 Mar 9;26(17):3738–43. doi: 10.1002/chem.202000412 (PMC7155052; doi:10.1002/chem.202000412)
Supplement: Supplementary file 1 — Supplementary [file CHEM-26-3738-s001.pdf]

CHEMISTRY
A European Journal

Supporting Information

Radical C—N Borylation of Aromatic Amines Enabled by a Pyrylium Reagent

Yuanhong Ma+,[a] Yue Pang+,[a] Sonia Chabbra,[b] Edward J. Reijerse,[b] Alexander Schnegg,[b] Jan Niski,[a] Markus Leutzsch,[a] and Josep Cornella*[a]

chem_202000412_sm_miscellaneous_information.pdf

Table of Contents

1. General Information……….…………………………………………………………….………S 3

2. Synthesis of Pyrylium Salts, Pyridinium Salts and Solvent.........…………………………….............S 4

3. Optimization Studies………………………………………………………..…………..….....S 39

4. General Procedure for C–N Borylation of Aromatic Amines…………………………….……….…..S 42

5. Characterization Data ………………………………………………………….….…………....S 43

6. Gram-scale Synthesis and One-pot Reaction ……………………………………………...………... S 55

7. Mechanistic Studies…………………………………………………………..……….……….......S 57

8. References…………………………………………………………………..………………….S 68

9. NMR spectra ………………………………………………………………………………..S 69

1. General Information

Unless otherwise stated, all manipulations were performed using standard Schlenk techniques under dry argon. Flash chromatography: Merck silica gel 60 (40-63 µm). GC-MS (FID): GC-MS-QP2010 equipped (Shimadzu Europe Analytical Instriuments). ESI-MS: ESQ 3000 (Bruker). Accurate mass determinations: Bruker APEX III FT-MS (7 T magnet) or MAT 95 (Finnigan). NMR spectra were recorded using a Bruker Avance VIII-300, Bruker Avance III HD 400 MHz, Bruker AVIII 500 MHz or Bruker *Ascend*^{TM} 500 MHz spectrometer. The chemical shifts were measured relative to solvent residual peak as an internal standard. For ^{1}H NMR: $CDCl_3$, δ 7.26; $(CD_3)_2SO$, δ 2.50; $(CD_3)_2CO$, δ 2.05. For ^{13}C NMR: $CDCl_3$, δ 77.16; $(CD_3)_2SO$, δ 39.52; $(CD_3)_2CO$, δ 29.84. EPR spectra were obtained using a commercial X-band spectrometer (MS5000, Magnettech GmbH) at 293 K using 10 mW microwave power, 100 kHz modulation frequency and 0.1 mT modulation amplitude. Anhydrous DMPU (CaH_2) was distilled and transferred under argon. Anhydrous Et_3N, DMF, DMSO and Dioxane were taken from solvent purification system. Anhydrous NMP and DMAc purchased from Millipore-Sigma and stored in an argon-filled glove box. Unless otherwise noted, all reagents were obtained from commercial suppliers and used without further purification. Most of amino-(hetero)arenes, bis(catecholato)diboron and methyliminodiacetic acid were purchased from TCI and Millipore-Sigma in Germany. 2,4,6-Triphenylpyrylium tetrafluoroborate was purchased from Millipore-Sigma in Germany. 2,4,6-Trimethylpyrylium tetrafluoroborate was purchased from TCI in Germany. Terpyridine was purchased from Millipore-Sigma in Germany or synthesized according to literature.[1] 4,4',6,6'-Tetra-tert-butyl-2,2'-bibenzo[*d*][*1*,*3*,*2*]dioxaborole and 5,5'-Dimethoxy-2,2'-bibenzo[*d*][*1*,*3*,*2*]dioxaborole were synthesized according to literature procedure.[2]

2. Synthesis of Pyrylium Salts, Pyridinium Salts and Solvent

2.1 Synthesis of Pyrylium Salts[3]

General Procedure I

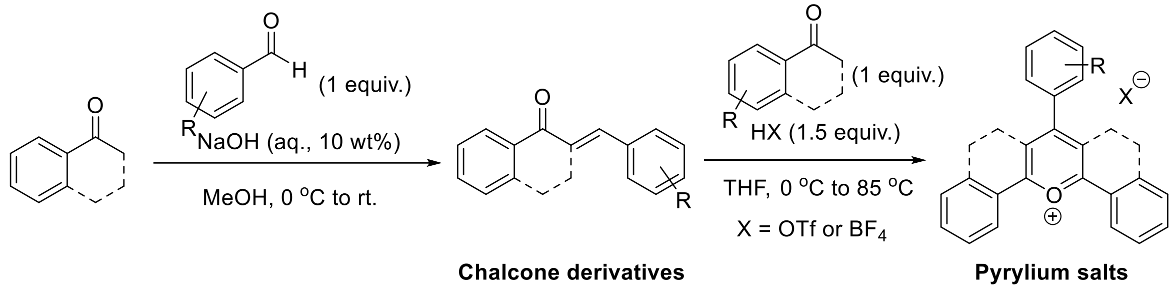

(a) Synthesis of chalcone derivatives

To a round-bottom flask the corresponding acetophenone, benzaldehyde (1.0 equiv.) and MeOH (0.5 mL/mmol) were added. The reaction system was cooled with an ice bath, to which NaOH aqueous solution (10 wt%, 5 equiv.) was added. The Ice bath was removed after addition. The mixture was stirred at room temperature until complete conversion was judged by TLC. If corresponding chalcone crushed out during reaction, the crude product was collected by filtration and washed twice with cool MeOH. And the product was dried under high vacuum. If the product doesn't precipitate, MeOH was evaporated and EtOAc was used to exact the product. The crude product was taken to the next step without further purification.

(b) Synthesis of pyrylium salts

To a 100 mL Schlenk flask the corresponding chalcone was charged, then the Schlenk flask was evacuated and refilled with argon three times. The corresponding acetophenone (1.0 equiv.) and anhydrous THF (0.5 mL/mmol) were added successively under argon. The solution was cooled to 0 °C and TfOH (1.5 equiv., for the synthesis of pyrylium trifluoromethanesulfonate) or HBF_4 (1.5 equiv., corresponding Et_2O complex, for the synthesis of pyrylium tetrafluoroborate) was added dropwise under stirring, during which the reaction mixture became dark. The reaction mixture was heated to reflux (temperature of oil bath: 85 °C) for 12 h, during which the pyrylium salt crushed out. After cooling down to room temperature, the pyrylium salt was collected by filtration, washed with Et_2O three times and dried under high vacuum.

Note 1: Since half equivalent of chalcone serves as hydride acceptor during pyrylium formation process, the yields are less than 50% for each case. The reaction scale and yield of this step are given below for each pyrylium salt.

Note 2: Pyrylium salts are not sensitive to air and moisture and not very sensitive to light, however, it was observed that pyrylium salts decomposed slowly in solution when they were not kept from air and light. The pyrylium salts with OTf or BF_4 as counterion don't tend to absorb moisture. As a result, pyrylium salts can be stored on bench-top in the dark for at least two months.

For large scale preparation, 250 mL two-necked round-bottom flask equipped with a condenser, a bubbler and a dropping funnel was used. Acid was added dropwise via dropping funnel. Preparation of ^{Sc}Pyry-OTf at 30 gram scale is illustrated with several images as follows:

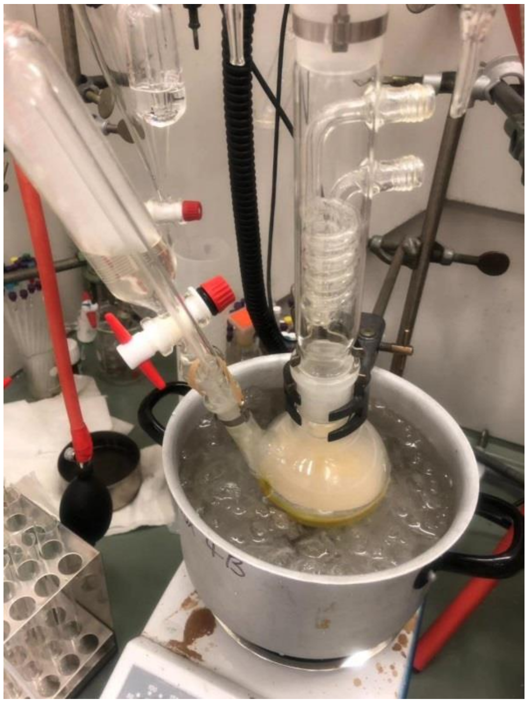

The reaction set-up

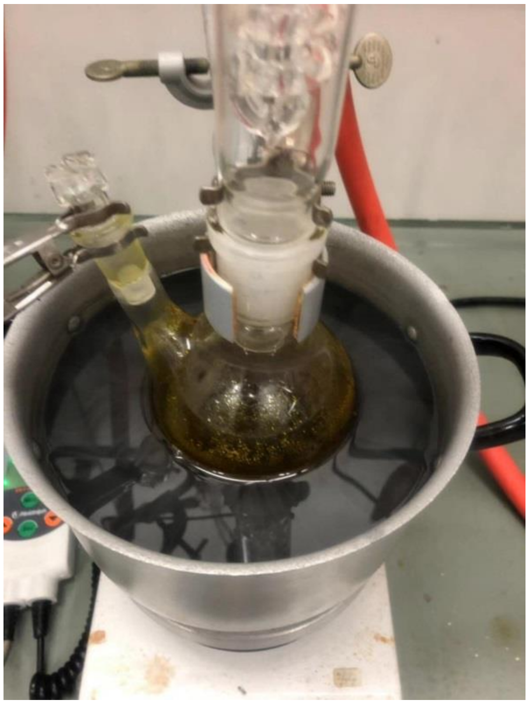

Reaction at reflux

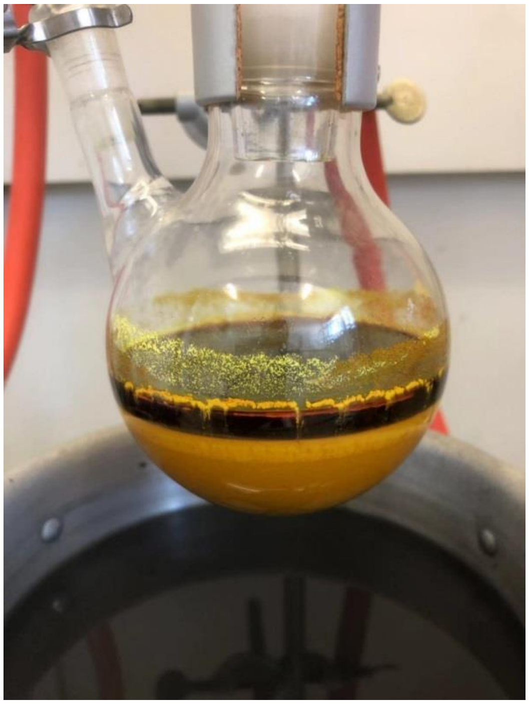

After reaction

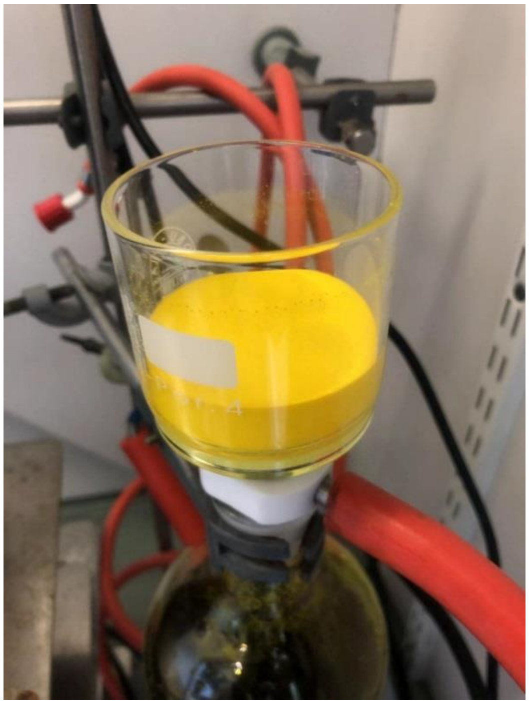

Filtration

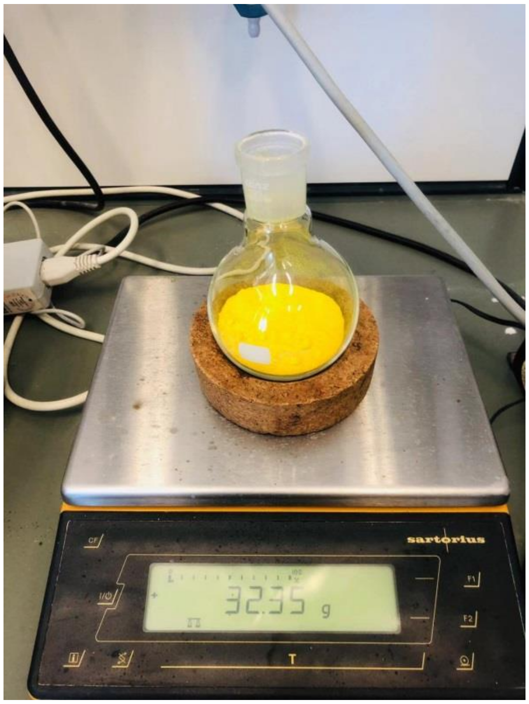

Final product

Characterization of Pyrylium Salts

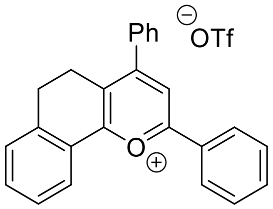

2,4-Diphenyl-5,6-dihydrobenzo[*h*]chromen-1-ium trifluoromethanesulfonate, S1

Prepared via general procedure I starting from α-tetralone and chalcone on 7.5 mmol scale to give **S1** (0.95 g, 26%) as a yellow solid. The compound contains THF which cannot be removed under high vacuum but doesn't affect subsequent condensation.

^{1}H NMR (500 MHz, DMSO-*d*$_6$): δ 8.83 (s, 1H), 8.55 (d, *J* = 7.6 Hz, 2H), 8.43 (d, *J* = 7.7 Hz, 1H), 7.89–7.81 (m, 3H), 7.81–7.71 (m, 6H), 7.66 (t, *J* = 7.6 Hz, 1H), 7.58 (d, *J* = 7.5 Hz, 1H), 3.25 (t, *J* = 7.4 Hz, 2H), 3.09 (t, *J* = 7.4 Hz, 2H).

^{13}C NMR (126 MHz, DMSO-*d*$_6$): δ 167.4, 166.6, 165.7, 142.0, 135.6, 134.7, 134.1, 132.1, 129.9, 129.4, 129.3, 129.2, 129.0, 128.8, 128.4, 128.4, 126.8, 126.0, 120.7 (q, *J* = 322.5 Hz, OTf), 119.1, 25.8, 24.3.

^{19}F NMR (470 MHz, DMSO-*d*$_6$): δ -77.7.

HRMS (ESI): calc'd for $C_{25}H_{19}O^+$ [M-OTf]$^+$ 335.143040; found 335.142620.

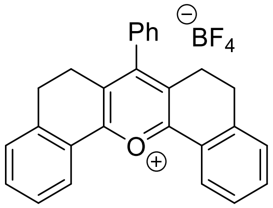

7-Phenyl-5,6,8,9-tetrahydrodibenzo[*c*,*h*]xanthen-14-ium tetrafluoroborate, S2

Prepared via general procedure I starting from α-tetralone and chalcone on 7.5 mmol scale to give **S2** (1.20 g, 16%) as an orange solid.

^{1}H NMR (400 MHz, DMSO-*d*$_6$): δ 8.44 (dd, *J* = 7.9, 1.2 Hz, 2H), 7.76 (td, *J* = 7.5, 1.2 Hz, 2H), 7.73–7.62 (m, 5H), 7.55 (d, *J* = 7.5 Hz, 2H), 7.50–7.45 (m, 2H), 3.06 (t, *J* = 7.5 Hz, 4H), 2.91–2.85 (m, 4H).

^{13}C NMR (101 MHz, DMSO-*d*$_6$): δ 165.5, 164.8, 141.9, 135.7, 133.0, 131.0, 130.6, 129.8, 129.5, 128.9, 127.9, 126.7, 126.1, 26.1, 24.6.

^{11}B NMR (128 MHz, DMSO-d_6): δ -1.3.

^{19}F NMR (471 MHz, DMSO-d_6): **δ** -148.3, -148.3.

HRMS (ESI): calc'd for $C_{27}H_{21}O^+$ [M-BF_4]$^+$ 361.158690; found 361.158510.

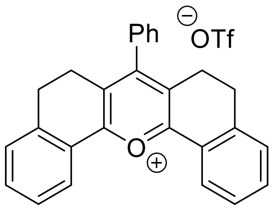

7-Phenyl-5,6,8,9-tetrahydrodibenzo[*c*,*h*]xanthen-14-ium trifluoromethanesulfonate, 7

Prepared via general procedure I starting from α-tetralone and benzaldehyde on 128 mmol scale to give **7** (32.4 g, 50%) as a bright yellow solid.

^{1}H NMR (500 MHz, DMSO-d_6): δ 8.43 (dd, *J* = 7.9, 1.2 Hz, 2H), 7.75 (td, *J* = 7.5, 1.3 Hz, 2H), 7.73–7.69 (m, 2H), 7.69–7.66 (m, 1H), 7.64 (td, *J* = 7.6, 1.2 Hz, 2H), 7.53 (d, *J* = 7.5 Hz, 2H), 7.52–7.49 (m, 2H), 3.06 (t, *J* = 7.6 Hz, 4H), 2.91–2.85 (m, 4H).

^{13}C NMR (126 MHz, DMSO-d_6): δ 165.1, 164.3, 141.4, 135.2, 132.5, 130.4, 130.1, 129.3, 129.0, 128.4, 127.4, 126.2, 125.6, 120.7 (q, *J* = 322.3 Hz, OTf), 25.6, 24.1.

^{19}F NMR (470 MHz, DMSO-d_6): δ -77.7.

HRMS (ESI): calc'd for $C_{27}H_{21}O^+$ [M-OTf]$^+$ 361.158690; found 361.158630.

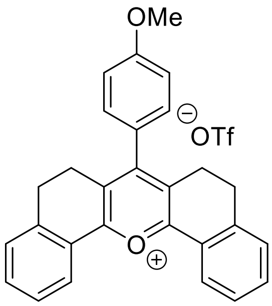

7-(4-Methoxyphenyl)-5,6,8,9-tetrahydrodibenzo[*c*,*h*]xanthen-14-ium trifluoromethanesulfonate, S3

Prepared via general procedure I starting from α-tetralone and 4-methoxybenzaldehyde on 1.9 mmol scale to give **S3** (0.49 g, 48%) as an orange solid.

^{1}H NMR (500 MHz, DMSO-*d_6*): δ 8.40 (dd, *J* = 7.9, 1.2 Hz, 2H), 7.74 (td, *J* = 7.5, 1.2 Hz, 2H), 7.64 (td, *J* = 7.6, 1.2 Hz, 2H), 7.54 (d, *J* = 7.5 Hz, 2H), 7.49–7.45 (m, 2H), 7.27–7.23 (m, 2H), 3.89 (s, 3H), 3.08–3.03 (m, 4H), 2.98–2.93 (m, 4H).

^{13}C NMR (126 MHz, DMSO-*d_6*): δ 165.1, 163.9, 161.0, 141.3, 135.1, 130.0, 129.9, 129.0, 128.3, 126.2, 125.8, 124.3, 120.7 (q, *J* = 322.4 Hz, OTf), 114.6, 55.5, 25.7, 24.3.

^{19}F NMR (470 MHz, DMSO-*d_6*): δ -77.7.

HRMS (ESI): calc'd for $C_{28}H_{23}O_2^+$ [M-OTf]$^+$ 391.169255; found 391.168900.

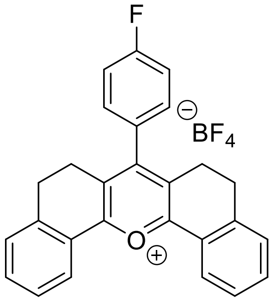

7-(4-Fluorophenyl)-5,6,8,9-tetrahydrodibenzo[*c*,*h*]xanthen-14-ium tetrafluoroborate, S4

The compound **S4** was prepared in one-pot version: α-tetralone (2.90 g, 20 mmol, 2.0 equiv.), 4-fluorobenzaldehyde (1.24 g, 10 mmol, 1.0 equiv.) and $BF_3{\cdot}OEt_2$ (3.1 mL, 25 mmol, 2.5 equiv.) were added into Schlenk flask successively and the reaction mixture was heated to 100 ^{o}C overnight. The reaction mixture was slurry at the end of the reaction. Acetone (5 mL) was used to dissolve the mixture and Et_2O (20 mL) was used to reprecipitate the pyrylium salt. After stirring for 30 min, the mixture was filtered to give **S4** (2.00 g, 43%) as a grass green powder. The compound contains certain impurities which can be removed in condensation step.

^{1}H NMR (500 MHz, DMSO-*d_6*): δ 8.43 (d, *J* = 7.8 Hz, 2H), 7.75 (t, *J* = 7.2 Hz, 2H), 7.64 (t, *J* = 7.7 Hz, 2H), 7.61–7.55 (m, 4H), 7.54 (d, *J* = 7.5 Hz, 2H), 3.07 (t, *J* = 7.5 Hz, 4H), 2.89 (t, *J* = 7.5 Hz, 4H).

^{13}C NMR (126 MHz, DMSO-*d_6*): δ 164.3, 164.2, 163.1 (d, *J* = 248.5 Hz), 141.5, 135.2, 130.3, 130.3, 129.0, 128.78 (d, *J* = 3.4 Hz), 128.4, 126.3, 125.6, 116.49 (d, *J* = 22.0 Hz), 25.6, 24.1.

^{11}B NMR (160 MHz, DMSO-*d_6*): δ -1.3.

^{19}F NMR (470 MHz, DMSO-*d_6*): δ -109.9, -148.4 (minor, $^{11}BF_4$), -148.5 (major, $^{10}BF_4$).

HRMS (ESI): calc'd for $C_{27}H_{20}OF^+$ [M-BF_4]$^+$ 379.149269; found 379.149200.

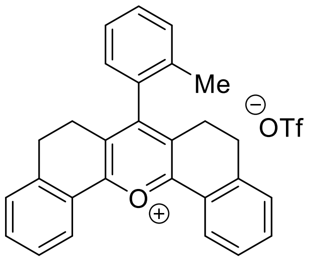

7-(*o*-Tolyl)-5,6,8,9-tetrahydrodibenzo[*c*,*h*]xanthen-14-ium trifluoromethanesulfonate, S5

Prepared via general procedure I starting from α-tetralone and 2-methylbenzaldehyde on 20 mmol scale to give **S5** (1.04 g, 10%) as orange solid. During work-up, Et_2O (ca. 150 mL) was used to precipite the pyrylium salt.

^{1}H NMR (500 MHz, DMSO-*d*$_6$): δ 8.47 (d, *J* = 7.3 Hz, 2H), 7.76 (td, *J* = 7.5, 1.3 Hz, 2H), 7.65 (td, *J* = 7.7, 1.2 Hz, 2H), 7.56–7.46 (m, 5H), 7.23 (d, *J* = 7.4 Hz, 1H), 3.07 (t, *J* = 7.6 Hz, 4H), 2.72 (tq, *J* = 16.7, 8.9, 8.3 Hz, 4H), 2.18 (s, 3H).

^{13}C NMR (126 MHz, DMSO-*d*$_6$): δ 165.9, 164.8, 142.0, 135.6, 134.7, 132.9, 131.3, 130.8, 130.5, 129.5, 128.8, 127.2, 126.7, 126.6, 126.1, 121.1 (q, *J* = 322.4 Hz, OTf), 26.0, 24.0, 19.3.

^{19}F NMR (470 MHz, DMSO-*d*$_6$): δ -77.7.

HRMS (ESI): calc'd for $C_{28}H_{23}O^{+}$ [M-OTf]$^{+}$ 375.174340; found 375.174300.

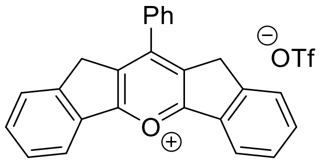

11-Phenyl-10,12-dihydrodiindeno[1,2-*b*:2',1'-*e*]pyran-5-ium trifluoromethanesulfonate, S6

Prepared via general procedure I starting from 2,3-dihydro-*1H*-inden-1-one and benzaldehyde on 8.1 mmol scale to give **S6** (0.81 g, 21%) as golden yellow solid.

^{1}H NMR (500 MHz, DMSO-*d*$_6$): δ 8.41 (dd, *J* = 7.7, 0.9 Hz, 2H), 8.15–8.11 (m, 2H), 7.91 (d, *J* = 7.6 Hz, 2H), 7.85 (td, *J* = 7.5, 1.1 Hz, 2H), 7.81–7.75 (m, 5H), 4.46 (s, 4H).

^{13}C NMR (126 MHz, DMSO-*d*$_6$): δ 169.6, 158.0, 147.9, 134.3, 133.4, 132.4, 132.3, 131.6, 129.8, 129.5, 128.9, 126.4, 122.8, 120.7 (q, *J* = 322, OTf), 33.9.

^{19}F NMR (470 MHz, DMSO-*d*$_6$): δ -77.7.

HRMS (ESI): calc'd for $C_{25}H_{17}O^{+}$ [M-OTf]$^{+}$ 333.127390; found 333.127090.

2.2 Synthesis of Pyridinium Salts[4]

General Procedure II

A 50 mL Schlenk flask equipped with a stirring bar was charged with corresponding pyrylium salt (1.5 mmol) and aniline (2.25 mmol, 1.5 equiv), and evaporated and refilled with argon (argon protection is not necessary but recommended). For less reactive anilines (*vide infra*), sodium pivalate (1.0 equiv) was required. Then EtOH (10 mL) was added to the Schlenk flask and the reaction was refluxed at 85 ^{o}C (temperature of oil bath). Full conversion was judged by TLC (DCM/Acetone) or crude ^{1}H NMR (the reaction rates depend significantly on anilines and pyrylium salts, and generally reactions were performed for 12 h). The product crushed out during the reaction or after cooling down, which was collected by filtration, washed with Et_2O three times and dried under high vacuum. The product that doesn't crush out can be purified by flash chromatography (DCM/Acetone, tailing).

Note: The pyridinium salts are stable on bench-top for at least two months and don't absorb moisture, however, it is recommended that they be stored under inert gas and in dark for the reproducibility of reactions.

General Remarks on NMR of Pyridinium Salts

In some cases, several ^{13}C NMR signals of pyridinium salts couldn't be observed due to overlap of signals. Whereas the carbon count for unsubstituted and *para*-substituted pyridinium salts posed no problem, substituted anilines at the *meta*- and *ortho*- position presented some difficulties due to the different symmetry they offer. Such overlap of signals has been confirmed by VT ^{13}C NMR (DMSO-d_6 or $CDCl_3$) of some pyridinium salts. Each overlap of signals occurs at different temperatures for different signals and for every pyridinium salt. The VT ^{13}C NMR of compounds **23** and **25** are given as examples (see below). ^{13}C NMR data for all of pyridinium salts were recorded in DMSO-d_6 or $CDCl_3$ at room temperature (25 ^{o}C) and reported as observed.

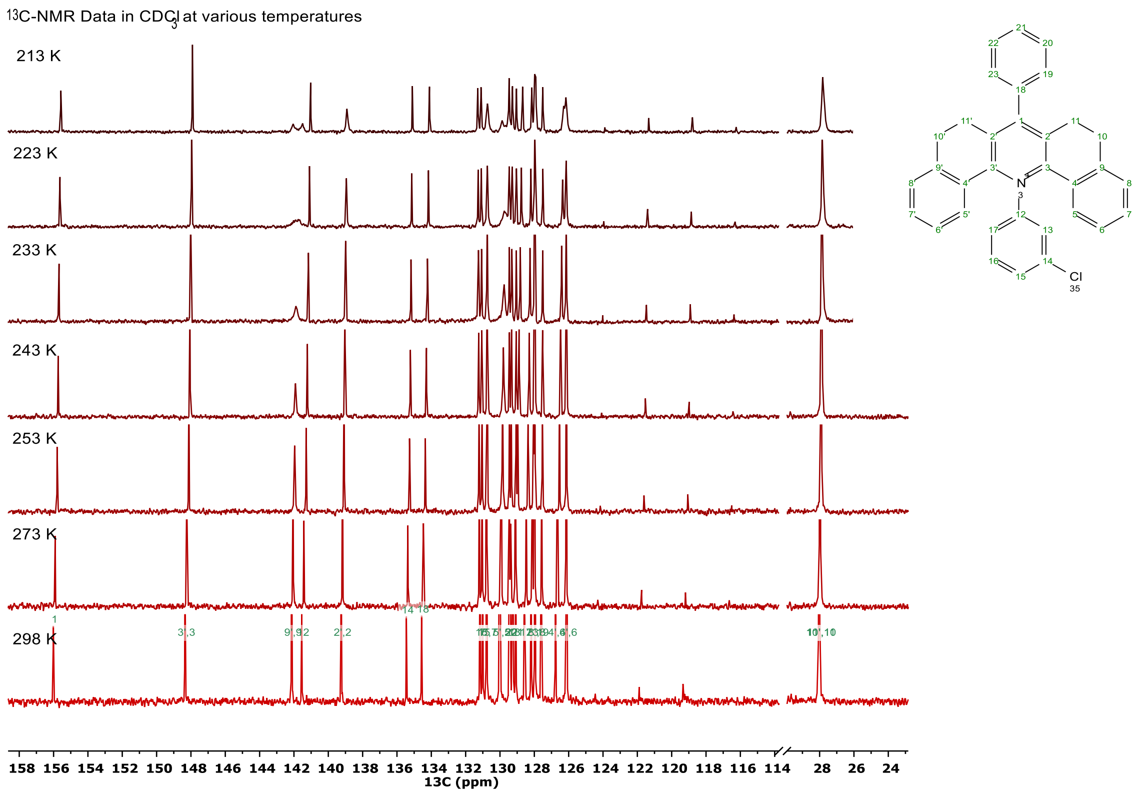

VT ^{13}C NMR (126 MHz, $CDCl_3$) of **23**

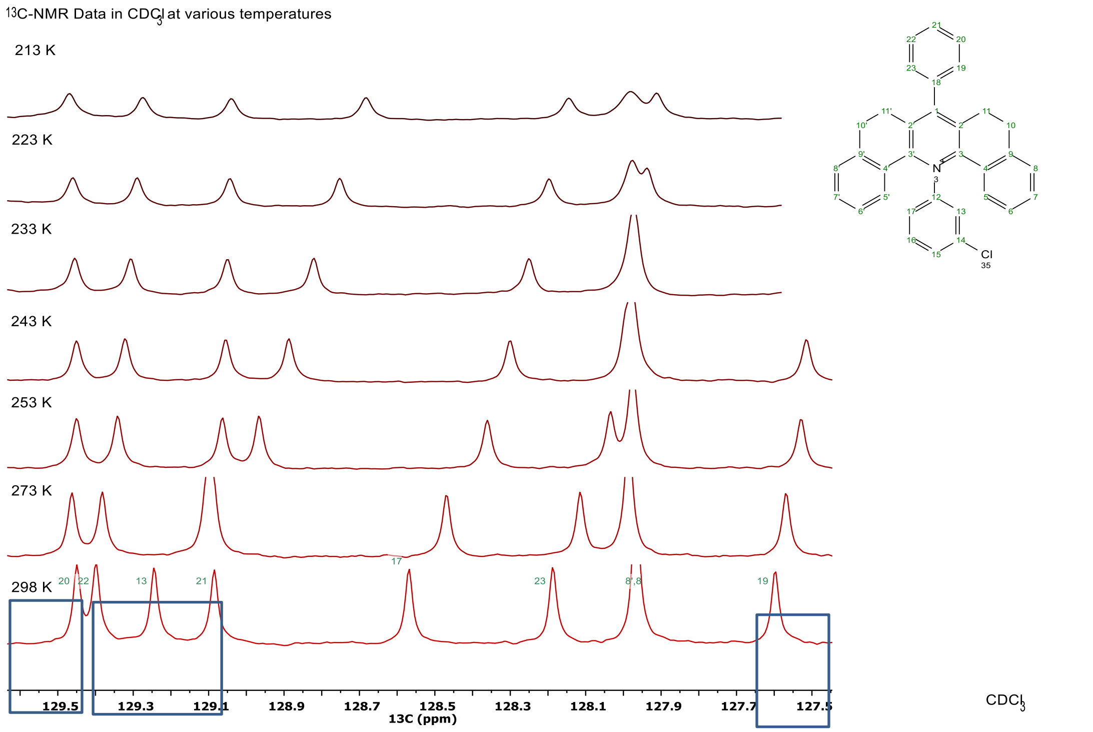

VT ^{13}C NMR (126 MHz, $CDCl_3$) of **23**

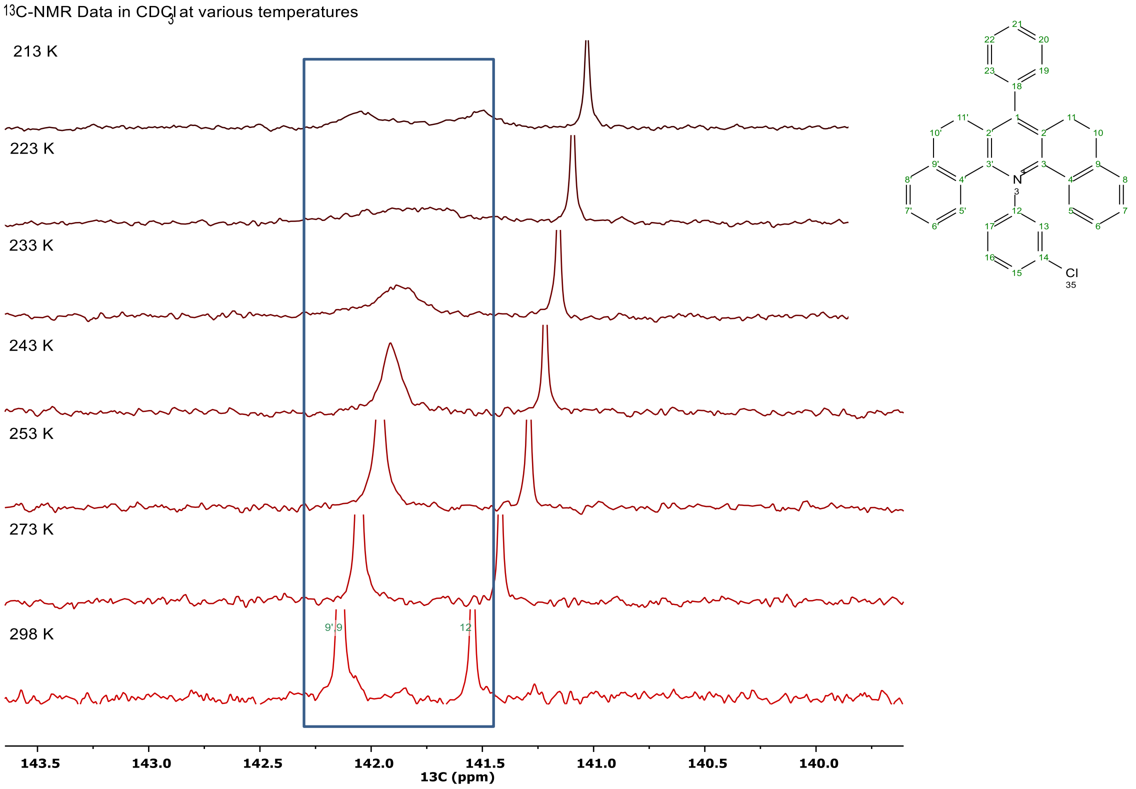

VT ^{13}C NMR (126 MHz, $CDCl_3$) of **23**

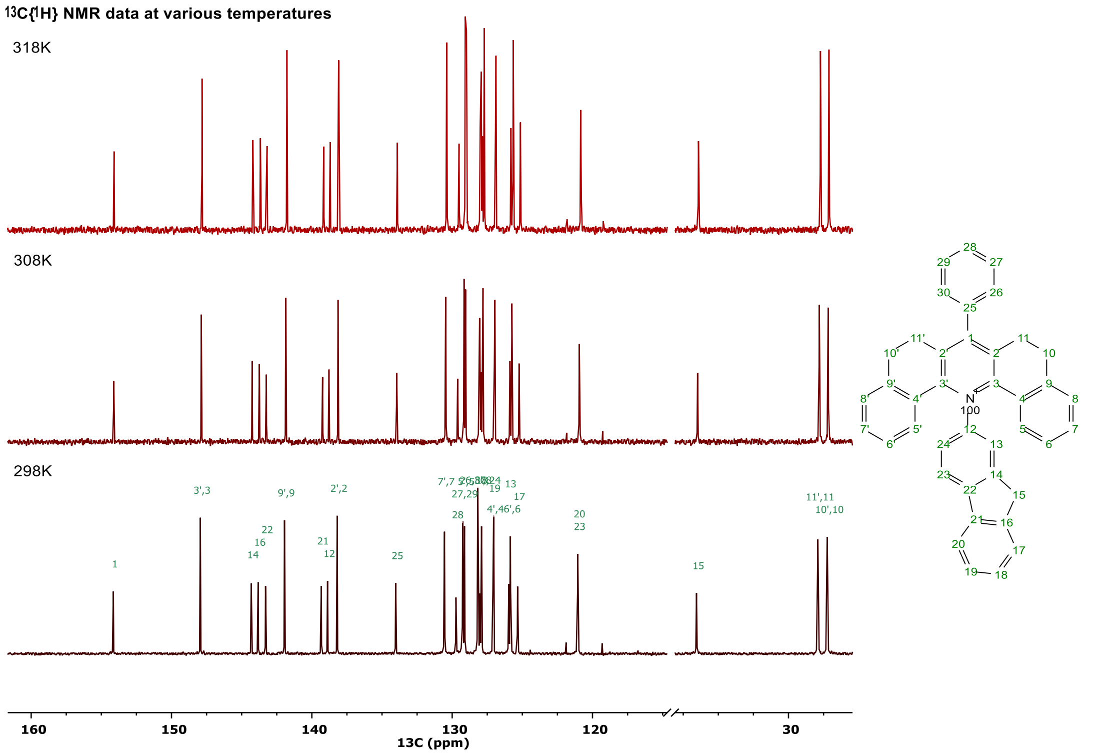

VT ^{13}C NMR (126 MHz, DMSO-d_6) of **25**

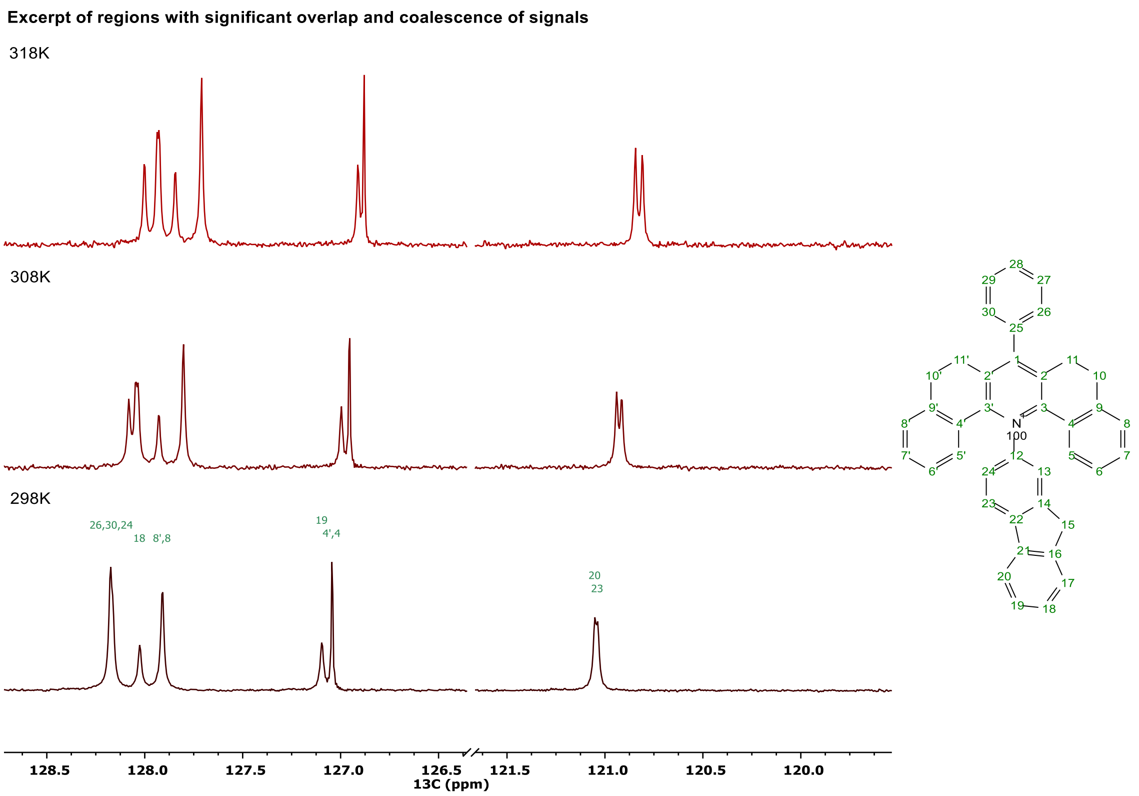

VT ^{13}C NMR (126 MHz, DMSO-*d*$_6$) of **25**

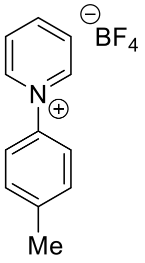

1-(*p*-Tolyl)pyridin-1-ium tetrafluoroborate, 1

The compound **1** was prepared as brownish red solid (0.27 g, 53%) starting from pyrylium tetrafluoroborate (0.34 g, 2.0 mmol) and *p*-toluidine (3.0 mmol, 1.5 equiv.) according to the procedure reported by our group.[5]

^{1}H NMR (400 MHz, $CDCl_3$): δ 8.93–8.88 (m, 2H), 8.60 (tt, *J* = 7.8, 1.3 Hz, 1H), 8.24–8.17 (m, 2H), 7.61–7.55 (m, 2H), 7.44–7.38 (m, 2H), 2.43 (s, 3H).

^{13}C NMR (101 MHz, $CDCl_3$): δ 146.5, 144.1, 142.7, 140.5, 131.5, 129.2, 123.9, 21.3.

^{11}B NMR (160 MHz, $CDCl_3$): δ -1.1.

^{19}F NMR (470 MHz, $CDCl_3$): δ -151.3 (minor, $^{11}BF_4$), -151.3 (major, $^{10}BF_4$).

HRMS (ESI): calc'd for $C_{12}H_{12}N^+$ $[M-BF_4]^+$ 170.096424; found 170.096360.

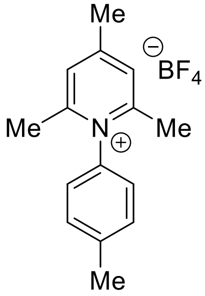

2,4,6-Trimethyl-1-(*p*-tolyl)pyridin-1-ium tetrafluoroborate, 2

Prepared via general procedure **II** with minor modification starting from commercially available 2,4,6-trimethylpyrylium tetrafluoroborate (0.63 g, 3.0 mmol) and *p*-toluidine (4.5 mmol, 1.5 equiv.). MeOH (10 mL) was used as solvent. After reaction, solvent was removed by evaporation and Et_2O (30 mL) was added to disperse the crude product. The mixture afforded fine powder upon stirring. Filtration, washing with Et_2O and drying under high vacuum gave a NMR pure off-white solid (0.72 g, 80%) **2** as product.

^{1}H NMR (400 MHz, $CDCl_3$): δ 7.63 (s, 2H), 7.49–7.43 (m, 2H), 7.30–7.24 (m, 2H), 2.62 (s, 3H), 2.48 (s, 3H), 2.37 (s, 6H).

^{13}C NMR (126 MHz, $CDCl_3$): δ 159.9, 154.9, 141.8, 135.9, 131.7, 127.8, 125.2, 22.0, 21.9, 21.3.

^{11}B NMR (160 MHz, $CDCl_3$): δ -1.3.

^{19}F NMR (470 MHz, DMSO-d_6): δ -148.3 (minor, $^{11}BF_4$), -148.3 (major, $^{10}BF_4$).

HRMS (ESI): calc'd for $C_{15}H_{18}N^+$ $[M-BF_4]^+$ 212.143374; found 212.143290.

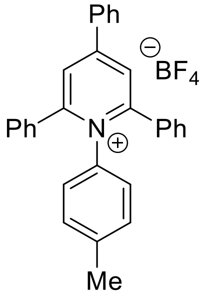

2,4,6-Triphenyl-1-(*p*-tolyl)pyridin-1-ium tetrafluoroborate, 3

Prepared via general procedure **II** with minor modification starting from commercially available 2,4,6-triphenylpyrylium tetrafluoroborate (1.58 g, 4.0 mmol) and *p*-toluidine (6.0 mmol, 1.5 equiv.) to give **3** (1.5 g, 77%) as off-white solid. After reaction, the mixture was poured into *n*-hexane and stirred until pyridinium salt became fine powder. Filtration, washing with Et_2O and drying under high vacuum gave NMR pure product 3 as white solid.

^{1}H NMR (500 MHz, DMSO-*d*$_6$): δ 8.66 (s, 2H), 8.38 – 8.34 (m, 2H), 7.73 – 7.64 (m, 3H), 7.47 – 7.43 (m, 4H), 7.42 – 7.35 (m, 6H), 7.33 (d, *J* = 8.3 Hz, 2H), 6.99 (d, *J* = 8.3 Hz, 2H), 2.12 (s, 3H).

^{13}C NMR (126 MHz, DMSO-*d*$_6$): δ 156.4, 155.3, 139.4, 136.7, 133.4, 133.2, 132.5, 129.9, 129.7, 129.6, 128.9, 128.8, 128.4, 128.1, 125.1, 20.5.

^{11}B NMR (160 MHz, DMSO-*d*$_6$): δ -1.3.

^{19}F NMR (470 MHz, DMSO-*d*$_6$): δ -148.3 (minor, $^{11}BF_4$), -148.3 (major, $^{10}BF_4$).

HRMS (ESI): calc'd for $C_{30}H_{24}N^+$ $[M-BF_4]^+$ 398.190324; found 398.190200.

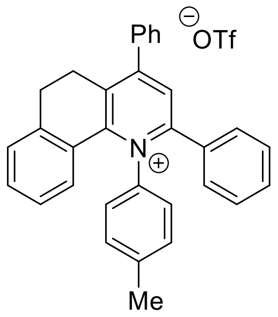

2,4-Diphenyl-1-(*p*-tolyl)-5,6-dihydrobenzo[*h*]quinolin-1-ium trifluoromethanesulfonate, S7

Prepared via general procedure **II** starting from **S1** (0.48 g, 1.0 mmol) and *p*-toluidine (0.16 g, 1.5 equiv) to give **S7** (0.41 g, 72%) as a yellow powder.

^{1}H NMR (500 MHz, DMSO-*d*$_6$): δ 8.18 (s, 1H), 7.82–7.76 (m, 2H), 7.69–7.62 (m, 3H), 7.47 (d, *J* = 7.5 Hz, 1H), 7.42–7.31 (m, 8H), 7.15 (d, *J* = 8.2 Hz, 2H), 6.98 (t, *J* = 7.7 Hz, 1H), 6.82 (d, *J* = 8.1 Hz, 1H), 3.13–3.06 (m, 2H), 2.98–2.91 (m, 2H), 2.26 (s, 3H).

^{13}C NMR (126 MHz, DMSO-*d*$_6$): δ 155.6, 153.8, 150.4, 142.5, 140.1, 137.9, 137.5, 135.3, 133.3, 131.3, 130.6, 129.6, 129.3, 129.1, 128.7, 128.0, 127.9, 127.9, 127.1, 126.0, 120.7 (q, *J* = 322.4 Hz, OTf), 27.8, 27.5, 20.6.

^{19}F NMR (470 MHz, DMSO-*d*$_6$): δ -77.7.

HRMS (ESI): calc'd for $C_{32}H_{26}N^+$ [M-OTf]$^+$ 424.205974; found 424.205840.

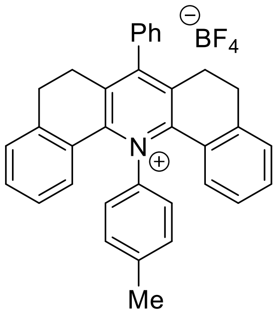

7-Phenyl-14-(*p*-tolyl)-5,6,8,9-tetrahydrodibenzo[*c*,*h*]acridin-14-ium tetrafluoroborate, 4-BF$_4$

Prepared via general procedure **II** starting from **S2** (1.12 g, 2.5 mmol) and *p*-toluidine (0.16 g, 5.0 mmol) for 72 h to give **4-BF$_4$** (1.08 g, 80%) as a pale yellow powder.

^{1}H NMR (400 MHz, DMSO-*d*$_6$): δ 7.68–7.59 (m, 3H), 7.53–7.42 (m, 6H), 7.36–7.29 (m, 4H), 6.96 (t, *J* = 7.6 Hz, 2H), 6.51 (d, *J* = 8.1 Hz, 2H), 2.94–2.86 (m, 4H), 2.76–2.68 (m, 4H), 2.40 (s, 3H).

^{13}C NMR (101 MHz, DMSO-*d*$_6$) δ 154.1, 147.9, 142.0, 141.1, 138.2, 138.1, 134.1, 130.6, 130.4, 129.8, 129.3, 129.1, 129.1, 128.2, 127.9, 127.1, 125.9, 27.9, 27.3, 20.8.

^{11}B NMR (128 MHz, DMSO-*d*$_6$): δ -1.3.

^{19}F NMR (470 MHz, DMSO-*d*$_6$): δ -148.3 (minor, $^{11}BF_4$), -148.3 (major, $^{10}BF_4$).

HRMS (ESI): calc'd for $C_{34}H_{28}N^+$ [M-BF$_4$]$^+$ 450.221624; found 450.221320.

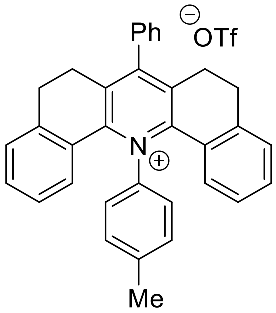

7-Phenyl-14-(*p*-tolyl)-5,6,8,9-tetrahydrodibenzo[*c*,*h*]acridin-14-ium trifluoromethanesulfonate, 4-OTf

Prepared via general procedure **II** starting from **7** (1.53 g, 3 mmol) and *p*-toluidine (0.48 g, 1.5 equiv) to give **4-OTf** (1.71 g, 95%) as a pale yellow powder.

^{1}**H NMR (400 MHz, DMSO-*d*$_6$)**: δ 7.68–7.58 (m, 3H), 7.54–7.46 (m, 4H), 7.44 (d, *J* = 7.4 Hz, 2H), 7.36–7.29 (m, 4H), 6.96 (t, *J* = 7.4 Hz, 2H), 6.52 (d, *J* = 8.0 Hz, 2H), 2.94–2.86 (m, 4H), 2.76–2.68 (m, 4H), 2.40 (s, 3H).

^{13}**C NMR (101 MHz, DMSO-*d*$_6$)**: δ 154.1, 147.9, 142.0, 141.1, 138.2, 138.1, 134.1, 130.6, 130.3, 129.7, 129.3, 129.1, 129.1, 128.2, 127.9, 127.1, 125.9, 120.7 (q, *J* = 322.6 Hz, OTf), 27.9, 27.3, 20.8.

^{19}**F NMR (470 MHz, DMSO-*d*$_6$)**: δ -77.7.

HRMS (ESI): calc'd for $C_{34}H_{28}N^+$ [M-OTf]$^+$ 450.221624; found 450.221470.

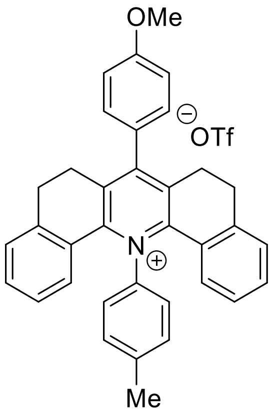

7-(4-Methoxyphenyl)-14-(*p*-tolyl)-5,6,8,9-tetrahydrodibenzo[*c*,*h*]acridin-14-ium trifluoromethanesulfonate, S8

Prepared via general procedure **II** starting from **S3** (1.00 g, 1.85 mmol) and *p*-toluidine (0.30 g, 1.5 equiv) to give **S8** (1.10 g, 95%) as a pale yellow powder.

^{1}**H NMR (500 MHz, DMSO-*d*$_6$)**: δ 7.48 (d, *J* = 8.6 Hz, 4H), 7.44 (d, *J* = 7.5 Hz, 2H), 7.35–7.30 (m, 4H), 7.21–7.16 (m, 2H), 6.96 (td, *J* = 7.9, 1.4 Hz, 2H), 6.51 (d, *J* = 8.1 Hz, 2H), 3.87 (s, 3H), 2.93–2.88 (m, 4H), 2.81–2.76 (m, 4H), 2.39 (s, 3H).

^{13}**C NMR (126 MHz, DMSO)**: δ 160.2, 154.1, 147.8, 141.9, 141.0, 138.4, 138.1, 130.5, 130.3, 130.1, 129.1, 129.1, 127.8, 127.2, 125.9, 125.8, 120.7 (q, *J* = 322.5 Hz, OTf), 114.6, 55.4, 28.1, 27.4, 20.8.

^{19}**F NMR (470 MHz, DMSO-*d*$_6$)**: δ -77.7.

HRMS (ESI): calc'd for $C_{35}H_{30}NO^+$ [M-OTf]$^+$ 480.232189; found 480.232440.

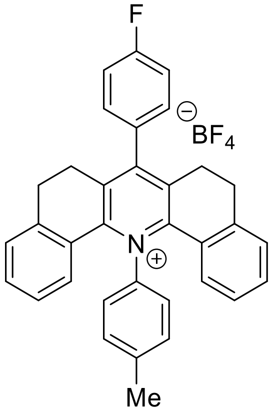

7-(4-Fluorophenyl)-14-(*p*-tolyl)-5,6,8,9-tetrahydrodibenzo[*c*,*h*]acridin-14-ium tetrafluoroborate, S9

Prepared via general procedure **II** starting from **S4** (0.47 g, 1.0 mmol) and *p*-toluidine (0.16 g, 1.5 equiv.) to give **S9** (0.47 g, 85%) as a yellow powder. *The condensation was run for 2 days.*

^{1}H NMR (500 MHz, DMSO-*d*$_6$): δ 7.63–7.57 (m, 2H), 7.52–7.47 (m, 4H), 7.45 (d, *J* = 7.6 Hz, 2H), 7.37–7.30 (m, 4H), 6.97 (t, *J* = 7.8 Hz, 2H), 6.52 (d, *J* = 8.1 Hz, 2H), 2.96–2.87 (m, 4H), 2.78–2.70 (m, 4H), 2.40 (s, 3H).

^{13}C NMR (126 MHz, DMSO-*d*$_6$): δ 162.7 (d, *J* = 247.4 Hz), 153.3, 147.9, 142.0, 141.1, 138.5, 138.1, 130.9 (d, *J* = 8.6 Hz), 130.6, 130.4, 129.1 (d, *J* = 5.4 Hz), 127.9, 127.1, 125.9, 116.4 (d, *J* = 21.9 Hz), 27.9, 27.3, 20.8.

^{11}B NMR (160 MHz, DMSO-*d*$_6$): δ -1.3.

^{19}F NMR (470 MHz, DMSO-*d*$_6$): δ -111.1, -148.4 (minor, $^{11}BF_4$), -148.4 (major, $^{10}BF_4$).

HRMS (ESI): calc'd for $C_{34}H_{27}NF^+$ $[M-BF_4]^+$ 468.212202; found 468.212350.

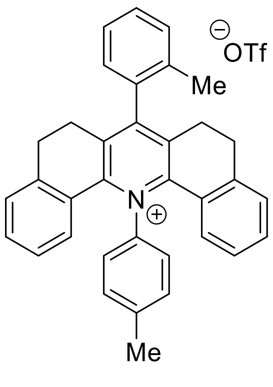

7-(*o*-Tolyl)-14-(*p*-tolyl)-5,6,8,9-tetrahydrodibenzo[*c*,*h*]acridin-14-ium trifluoromethanesulfonate, S10

Prepared via general procedure **II** starting from **S5** (0.53g, 1.0 mmol) and *p*-toluidine (0.16 g, 1.5 equiv.) to give **S10** (0.50 g, 82%) as a yellow powder.

^{1}H NMR (500 MHz, DMSO-d_6): δ 7.56–7.41 (m, 7H), 7.38–7.29 (m, 4H), 7.26 (d, *J* = 7.4 Hz, 1H), 6.96 (t, *J* = 7.6 Hz, 2H), 6.52 (d, *J* = 8.1 Hz, 2H), 2.92 (t, *J* = 6.4 Hz, 4H), 2.61–2.45 (m, 4H), 2.41 (s, 3H), 2.15 (s, 3H).

^{13}C NMR (126 MHz, DMSO-d_6): δ 154.1, 147.7, 142.1, 141.1, 138.4, 138.1, 134.8, 133.8, 130.8, 130.6, 130.4, 130.4, 129.5, 129.2, 129.1, 129.0, 128.0, 127.3, 127.0, 126.9, 125.9, 120.65 (q, *J* = 322.5 Hz, OTf), 27.1, 27.0, 20.8, 19.1.

^{19}F NMR (470 MHz, DMSO-d_6): δ -77.7.

HRMS (ESI): calc'd for $C_{35}H_{30}N^+$ $[M\text{-}OTf]^+$ 464.237274; found 464.237400.

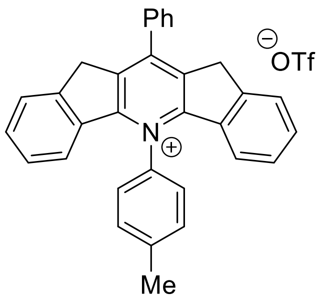

11-Phenyl-5-(*p*-tolyl)-10,12-dihydrodiindeno[1,2-*b*:2',1'-*e*]pyridin-5-ium trifluoromethanesulfonate, S11

Prepared via general procedure **II** starting from **S6** (0.48g, 1.0 mmol) and *p*-toluidine (0.16 g, 1.5 equiv.) to give **S11** (0.12 g, 21%) as a golden crystallized solid.

^{1}H NMR (500 MHz, $CDCl_3$): δ 7.78–7.73 (m, 4H), 7.71 (d, *J* = 8.1 Hz, 2H), 7.64 (t, *J* = 7.3 Hz, 2H), 7.61–7.56 (m, 3H), 7.46 (t, *J* = 7.4 Hz, 2H), 7.16 (t, *J* = 7.7 Hz, 2H), 6.15 (d, *J* = 8.2 Hz, 2H), 4.08 (s, 4H), 2.71 (s, 3H).

^{13}C NMR (126 MHz, $CDCl_3$): δ 152.4, 151.7, 146.6, 143.4, 138.9, 136.0, 134.0, 133.5, 132.7, 131.8, 130.2, 129.4, 128.3, 128.0, 126.3, 126.0, 124.1, 120.7 (q, *J* = 321.3 Hz, OTf), 35.0, 21.9.

^{19}F NMR (470 MHz, $CDCl_3$): δ -78.4.

HRMS (ESI): calc'd for $C_{32}H_{24}N^+$ $[M\text{-}OTf]^+$ 422.190324; found 422.190000.

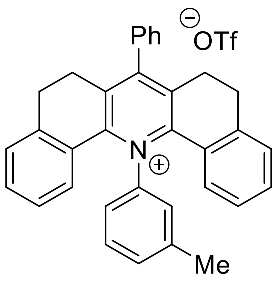

7-Phenyl-14-(*m*-tolyl)-5,6,8,9-tetrahydrodibenzo[*c*,*h*]acridin-14-ium trifluoromethanesulfonate, 8

Prepared via general procedure **II** starting from **7** (0.77 g, 1.5 mmol) and *m*-toluidine (0.24 g, 1.5 equiv.) to give **8** (0.72 g, 80%) as a pale yellow powder.

^{1}H NMR (400 MHz, DMSO-*d*$_6$): δ 7.68–7.59 (m, 3H), 7.54–7.47 (m, 2H), 7.47–7.37 (m, 6H), 7.33 (t, *J* = 7.4 Hz, 2H), 6.95 (t, *J* = 7.5 Hz, 2H), 6.51 (d, *J* = 8.1 Hz, 2H), 2.94–2.86 (m, 4H), 2.76–2.69 (m, 4H), 2.22 (s, 3H).

^{13}C NMR (101 MHz, DMSO-*d*$_6$): δ 154.3, 147.8, 142.0, 140.3, 139.8, 138.2, 134.1, 131.6, 130.6, 129.7, 129.5, 129.3, 129.1, 128.2, 127.9, 127.0, 126.5, 125.8, 120.7 (q, *J* = 322.3 Hz, OTf), 27.9, 27.3, 20.8. Two aryl-Cs are overlapped.

^{19}F NMR (470 MHz, DMSO-*d*$_6$): δ -77.7.

HRMS (ESI): calc'd for $C_{34}H_{28}N^+$ $[M\text{-}OTf]^+$ 450.221624; found 450.221550.

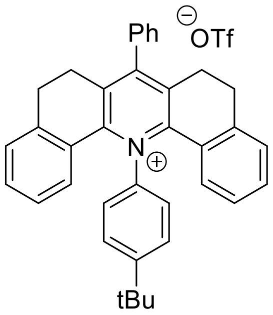

14-(4-(*tert*-Butyl)phenyl)-7-phenyl-5,6,8,9-tetrahydrodibenzo[*c*,*h*]acridin-14-ium trifluoromethanesulfonate, 9

Prepared via general procedure **II** starting from **7** (0.77 g, 1.5 mmol) and 4-(*tert*-butyl)aniline (0.34 g, 1.5 equiv.) to give **9** (0.72 g, 75%) as a pale yellow powder.

^{1}H NMR (500 MHz, DMSO-*d*$_6$): δ 7.68–7.59 (m, 3H), 7.55–7.47 (m, 6H), 7.43 (d, *J* = 7.4 Hz, 2H), 7.33 (td, *J* = 7.4, 0.7 Hz, 2H), 6.93–6.88 (m, 2H), 6.48 (d, *J* = 8.1 Hz, 2H), 2.90 (t, *J* = 6.6 Hz, 4H), 2.76–2.69 (m, 4H), 1.31 (s, 9H).

^{13}C NMR (101 MHz, DMSO-*d*$_6$): δ 154.5, 154.2, 147.9, 142.0, 138.2, 138.0, 134.1, 130.7, 129.8, 129.3, 129.2, 128.9, 128.2, 127.9, 127.0, 126.7, 125.6, 120.7 (q, *J* = 322.4 Hz, OTf), 34.8, 30.9, 27.9, 27.3.

^{19}F NMR (470 MHz, DMSO-*d*$_6$): δ -77.8.

HRMS (ESI): calc'd for $C_{37}H_{34}N^+$ $[M\text{-}OTf]^+$ 492.268574; found 492.268490.

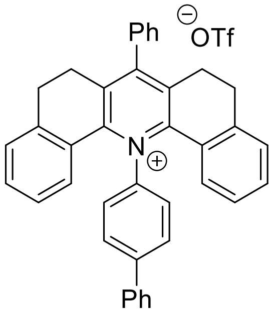

14-([1,1'-Biphenyl]-4-yl)-7-phenyl-5,6,8,9-tetrahydrodienzo[*c*,*h*]acridin-14-ium trifluoromethanesulfonate, 10

Prepared via general procedure **II** starting from **7** (0.51 g, 1.0 mmol) and [1,1'-biphenyl]-4-amine (0.20 g, 1.2 equiv.) to give **10** (0.61 g, 92%) as an off-white powder. Sodium pivalate was used for this condensation.

^{1}H NMR (400 MHz, DMSO-*d*$_6$): δ 7.90–7.85 (m, 2H), 7.81–7.75 (m, 2H), 7.73–7.60 (m, 5H), 7.55–7.48 (m, 4H), 7.48–7.42 (m, 3H), 7.33 (td, *J* = 7.5, 1.1 Hz, 2H), 6.96 (td, *J* = 7.8, 1.4 Hz, 2H), 6.60 (d, *J* = 8.1 Hz, 2H), 2.93 (t, *J* = 6.4 Hz, 4H), 2.80–2.70 (m, 4H).

^{13}C NMR (101 MHz, DMSO-*d*$_6$): δ 154.3, 147.9, 142.2, 142.1, 139.7, 138.3, 137.8, 134.1, 130.7, 129.9, 129.8, 129.3, 129.2, 129.1, 128.6, 128.2, 128.0, 127.7, 127.0, 126.9, 125.9, 120.7 (q, *J* = 322.4 Hz, OTf), 27.9, 27.3.

^{19}F NMR (470 MHz, DMSO-*d*$_6$): δ -77.7.

HRMS (ESI): calc'd for $C_{39}H_{30}N^{+}$ [M-OTf]$^{+}$ 512.237274; found 512.237370.

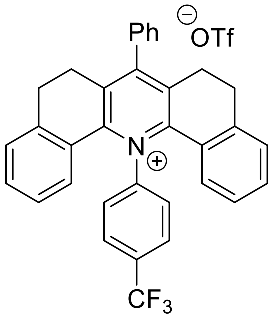

7-Phenyl-14-(4-(trifluoromethyl)phenyl)-5,6,8,9-tetrahydrodibenzo[*c*,*h*]acridin-14-ium trifluoromethanesulfonate, 11

Prepared via general procedure **II** starting from **7** (0.51 g, 1.0 mmol) and 4-(trifluoromethyl)aniline (0.17 g, 1.5 equiv.) to give **11** (0.55 g, 84%) as a pale yellow powder. Sodium pivalate was used for this condensation.

^{1}H NMR (400 MHz, DMSO-*d*$_6$): δ 7.96 (d, *J* = 8.6 Hz, 2H), 7.90 (d, *J* = 8.5 Hz, 2H), 7.71–7.61 (m, 3H), 7.55–7.50 (m, 2H), 7.48 (d, *J* = 7.3 Hz, 2H), 7.36 (td, *J* = 7.5, 1.2 Hz, 2H), 6.97 (td, *J* = 7.9, 1.4 Hz, 2H), 6.43 (d, *J* = 8.0 Hz, 2H), 2.98–2.88 (m, 4H), 2.81–2.72 (m, 4H).

^{13}C NMR (101 MHz, DMSO-*d*$_6$): δ 154.5, 147.9, 143.6, 142.3, 138.4, 133.9, 131.2 (q, *J* = 32.6 Hz), 130.9, 130.8, 129.9, 129.3, 129.3, 128.2, 128.1, 127.2 (q, *J* = 3.8 Hz), 126.6, 125.9, 123.4 (q, *J* = 272.8 Hz), 120.6 (q, *J* = 323.6 Hz, OTf), 27.9, 27.2.

^{19}F NMR (470 MHz, DMSO-*d*$_6$): δ -61.2, -77.8.

HRMS (ESI): calc'd for $C_{34}H_{25}F_3N^+$ [M-OTf]$^+$ 504.193359; found 504.193410.

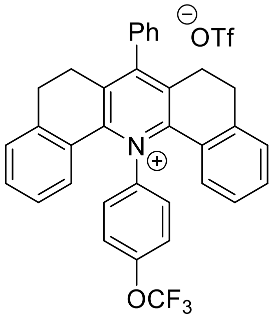

7-Phenyl-14-(4-(trifluoromethoxy)phenyl)-5,6,8,9-tetrahydrodibenzo[*c*,*h*]acridin-14-ium trifluoromethanesulfonate, 12

Prepared via general procedure **II** starting from **7** (0.77 g, 1.5 mmol) and 4-(trifluoromethoxy)aniline (0.40 g, 1.5 equiv.) to give **12** (0.82 g, 82%) as a pale yellow powder. The pyridinum salt didn't crush out after cooling down. It was purified by flash chromatography [DCM (100%) to DCM/Acetone (2/1, v/v)] and dispersed in Et_2O until the pyridinium salt became fluffy powder and collected by filtration.

^{1}H NMR (400 MHz, DMSO-*d*$_6$): δ 7.82–7.76 (m, 2H), 7.65 (d, *J* = 7.3 Hz, 3H), 7.58 (d, *J* = 8.4 Hz, 2H), 7.53–7.49 (m, 2H), 7.46 (d, *J* = 7.3 Hz, 2H), 7.36 (td, *J* = 7.5, 1.2 Hz, 2H), 6.98 (td, *J* = 7.8, 1.4 Hz, 2H), 6.46 (d, *J* = 8.0 Hz, 2H), 2.95–2.87 (m, 4H), 2.79–2.71 (m, 4H).

^{13}C NMR (101 MHz, DMSO-*d*$_6$): δ 154.5, 149.56 (q, *J* = 1.8 Hz), 148.0, 142.3, 139.3, 138.3, 134.0, 131.8, 130.8, 129.8, 129.3, 129.3, 128.2, 128.0, 126.8, 125.8, 122.6, 120.6 (q, *J* = 323.6 Hz, OTf), 119.87 (q, *J* = 257.8 Hz), 27.9, 27.2.

^{19}F NMR (470 MHz, DMSO-*d*$_6$): δ -57.1, -77.8.

HRMS (ESI): calc'd for $C_{34}H_{25}F_3NO^+$ [M-OTf]$^+$ 520.188274; found 520.188240.

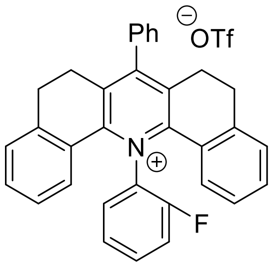

14-(2-Fluorophenyl)-7-phenyl-5,6,8,9-tetrahydrodibenzo[*c*,*h*]acridin-14-ium trifluoromethanesulfonate, 13

Prepared via general procedure **II** starting from **7** (0.77 g, 1.5 mmol) and 2-fluoroaniline (0.25 g, 1.5 equiv.) to give **13** (0.53 g, 59%) as a pale yellow powder. Sodium pivalate was used for this condensation. The crude product was further purified by flash chromatography (DCM/Acetone = 5:1, v/v).

^{1}H NMR (400 MHz, CDCl$_3$): δ 8.00 (td, *J* = 7.8, 1.7 Hz, 1H), 7.69–7.65 (m, 1H), 7.62–7.49 (m, 4H), 7.33–7.21 (m, 6H), 7.09 (ddd, *J* = 9.7, 8.4, 1.2 Hz, 1H), 6.88 (ddd, *J* = 8.7, 7.3, 1.6 Hz, 2H), 6.60 (d, *J* = 8.1 Hz, 2H), 3.19–3.07 (m, 2H), 2.86–2.71 (m, 4H), 2.71–2.56 (m, 2H).

^{13}C NMR (101 MHz, DMSO): δ 155.8 (d, *J* = 252.2 Hz), 155.5, 148.1, 142.2, 138.6, 134.3 (d, *J* = 8.0 Hz), 133.9, 131.3, 131.0, 129.9, 129.2, 128.5, 128.2, 128.1, 127.9, 127.7 (d, *J* = 11.7 Hz), 120.7 (q, *J* = 322.4 Hz, OTf), 126.4, 126.3, 126.2, 117.7 (d, *J* = 18.6 Hz), 27.8, 27.3.

^{19}F NMR (470 MHz, DMSO): δ -77.7, -121.3.

HRMS (ESI): calc'd for $C_{33}H_{25}FN^{+}$ [M-OTf]$^{+}$ 454.196552; found 454.196320.

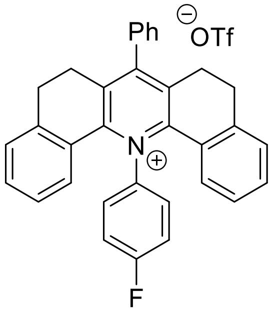

14-(4-Fluorophenyl)-7-phenyl-5,6,8,9-tetrahydrodibenzo[*c*,*h*]acridin-14-ium trifluoromethanesulfonate, 14

Prepared via general procedure **II** starting from **7** (0.51 g, 1.0 mmol) and 4-fluoroaniline (0.17 g, 1.5 mmol) to give **14** (0.55 g, 92%) as a pale yellow powder. Sodium pivalate was used for this condensation.

^{1}H NMR (400 MHz, DMSO-*d*$_6$): δ 7.73–7.59 (m, 5H), 7.54–7.48 (m, 2H), 7.48–7.38 (m, 4H), 7.38–7.32 (m, 2H), 7.03 (td, *J* = 7.8, 1.4 Hz, 2H), 6.49 (d, *J* = 8.0 Hz, 2H), 2.91 (t, *J* = 6.6 Hz, 4H), 2.79–2.69 (m, 4H).

^{13}C NMR (101 MHz, DMSO-*d*$_6$): δ 162.8 (d, *J* = 250.4 Hz), 154.3, 148.0, 142.2, 138.2, 136.8 (d, *J* = 3.2 Hz), 134.0, 131.8 (d, *J* = 9.1 Hz), 130.7, 129.8, 129.3, 129.2, 128.2, 128.0, 126.9, 126.0, 120.7 (q, *J* = 322.3 Hz, OTf), 117.1 (d, *J* = 23.5 Hz), 27.9, 27.2.

^{19}F NMR (470 MHz, DMSO-*d*$_6$): δ -77.7, -108.6.

HRMS (ESI): calc'd for $C_{33}H_{25}FN^+$ $[M\text{-}OTf]^+$ 454.196552; found 454.196460.

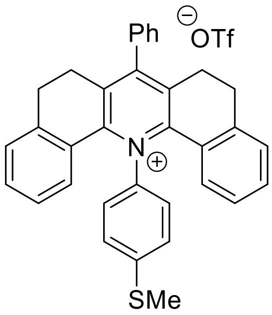

14-(4-(Methylthio)phenyl)-7-phenyl-5,6,8,9-tetrahydrodibenzo[*c*,*h*]acridin-14-ium trifluoromethanesulfonate, 15

Prepared via general procedure **II** starting from **7** (0.77 g, 1.5 mmol) and 4-(methylthio)aniline (0.31 g, 2.25 mmol) to give **15** (0.88 g, 93%) as a pale yellow solid.

^{1}H NMR (400 MHz, DMSO-*d*$_6$): δ 7.68 – 7.59 (m, 3H), 7.55 – 7.48 (m, 4H), 7.45 (d, *J* = 7.3 Hz, 2H), 7.39 – 7.31 (m, 4H), 7.01 (td, *J* = 7.8, 1.4 Hz, 2H), 6.56 (d, *J* = 8.0 Hz, 2H), 2.94 – 2.86 (m, 4H), 2.76 – 2.69 (m, 4H), 2.52 (s, 3H).

^{13}C NMR (101 MHz, DMSO-*d*$_6$): δ 154.1, 147.9, 142.7, 142.0, 138.2, 137.0, 134.0, 130.7, 129.8, 129.6, 129.3, 129.1, 128.2, 128.0, 127.0, 126.2, 126.0, 120.7 (d, *J* = 322.2 Hz, OTf), 27.9, 27.3, 14.3.

^{19}F NMR (470 MHz, DMSO-*d*$_6$): δ -77.8.

HRMS (ESI): calc'd for $C_{34}H_{28}NS^+$ $[M\text{-}OTf]^+$ 482.193696; found 482.193760.

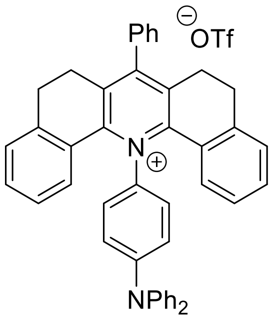

14-(4-(Diphenylamino)phenyl)-7-phenyl-5,6,8,9-tetrahydrodibenzo[*c*,*h*]acridin-14-ium trifluoromethanesulfonate, 16

Prepared via general procedure **II** starting from **7** (2.45 g, 4.8 mmol) and *N'*,*N'*-diphenylbenzene-1,4-diamine (1.87 g, 7.2 mmol, prepared via two-step synthesis starting from diphenylamine and 1-fluoro-4-nitrobenzene according to literature report[6]) to give **16** (2.15 g, 60%) as a pale yellow solid.

^{1}H NMR (400 MHz, DMSO-*d*$_6$): δ 7.69–7.58 (m, 3H), 7.53–7.48 (m, 2H), 7.47–7.34 (m, 10H), 7.24–7.18 (m, 2H), 7.14 (t, *J* = 7.4 Hz, 2H), 7.09 (d, *J* = 7.9 Hz, 4H), 7.01–6.95 (m, 2H), 6.76 (d, *J* = 8.1 Hz, 2H), 2.94–2.84 (m, 4H), 2.77–2.68 (m, 4H).

^{13}C NMR (101 MHz, DMSO-*d*$_6$): δ 154.1, 149.4, 147.8, 146.3, 142.0, 138.1, 134.1, 133.9, 130.7, 130.4, 129.8, 129.7, 129.4, 129.3, 128.2, 128.0, 127.1, 125.8, 124.6, 124.2, 122.8, 120.7 (q, *J* = 322.3 Hz, OTf), 27.9, 27.3.

^{19}F NMR (470 MHz, DMSO-*d*$_6$): δ -77.7.

HRMS (ESI): calc'd for $C_{45}H_{35}N_2^+$ [M-OTf]$^+$ 603.279472; found 603.279100.

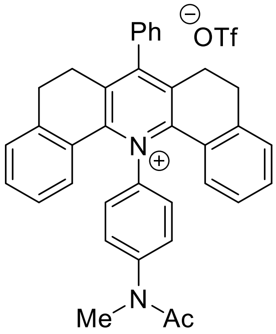

14-(4-(*N*-Methylacetamido)phenyl)-7-phenyl-5,6,8,9-tetrahydrodibenzo[*c*,*h*]acridin-14-ium trifluoromethanesulfonate, 17

Prepared via general procedure **II** starting from **7** (0.46 g, 0.9 mmol) and *N*-(4-aminophenyl)-*N*-methylacetamide (0.23 g, 1.5 equiv.) to give **17** (0.56 g, 92%) as an off-white powder.

^{1}H NMR (300 MHz, DMSO-*d*$_6$): δ 7.73 – 7.59 (m, 5H), 7.58 – 7.48 (m, 4H), 7.46 (d, *J* = 7.3 Hz, 2H), 7.35 (t, *J* = 7.3 Hz, 2H), 6.97 (t, *J* = 7.4 Hz, 2H), 6.55 (d, *J* = 8.0 Hz, 2H), 3.21 (s, 3H), 2.97 – 2.85 (m, 4H), 2.81 – 2.69 (m, 4H), 1.89 (s, 3H).

^{13}C NMR (101 MHz, DMSO-*d*$_6$): δ 168.9, 154.4, 147.9, 146.6, 142.2, 138.9, 138.2, 134.1, 130.8, 130.6, 129.8, 129.4, 129.3, 128.5, 128.2, 128.0, 126.9, 125.7, 120.7 (q, *J* = 322.4 Hz, OTf), 36.7, 27.9, 27.2, 22.3.

^{19}F NMR (470 MHz, DMSO-*d*$_6$): δ -77.7.

HRMS (ESI): calc'd for $C_{32}H_{31}N_2O^+$ [M-OTf]$^+$ 507.243088; found 507.242780.

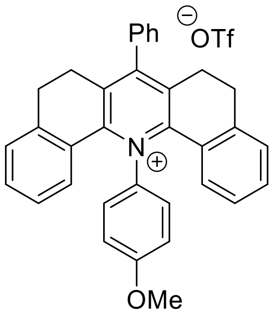

14-(4-Methoxyphenyl)-7-phenyl-5,6,8,9-tetrahydrodibenzo[*c*,*h*]acridin-14-ium trifluoromethanesulfonate, 18

Prepared via general procedure **II** starting from **7** (0.51 g, 1.0 mmol) and 4-methoxyaniline (0.18 g, 1.5 equiv.) to give **18** (0.53 g, 85%) as a pale yellow powder.

^{1}H NMR (400 MHz, DMSO-*d*$_6$): δ 7.69–7.59 (m, 3H), 7.56–7.48 (m, 4H), 7.45 (d, *J* = 7.4 Hz, 2H), 7.34 (t, *J* = 7.4 Hz, 2H), 7.06 (d, *J* = 9.0 Hz, 2H), 7.01 (t, *J* = 7.7 Hz, 2H), 6.57 (d, *J* = 8.1 Hz, 2H), 3.83 (s, 3H), 2.97–2.87 (m, 4H), 2.77–2.69 (m, 4H).

^{13}C NMR (101 MHz, DMSO-*d*$_6$): δ 160.7, 154.1, 148.0, 142.0, 138.1, 134.1, 133.4, 130.6, 130.5, 129.7, 129.3, 129.1, 128.2, 127.9, 127.2, 125.9, 120.7 (q, *J* = 322.6 Hz, OTf), 115.0, 55.8, 28.0, 27.3.

^{19}F NMR (470 MHz, DMSO-*d*$_6$): δ -77.7.

HRMS (ESI): calc'd for $C_{34}H_{28}NO^+$ [M-OTf]$^+$ 466.216539; found 466.216460.

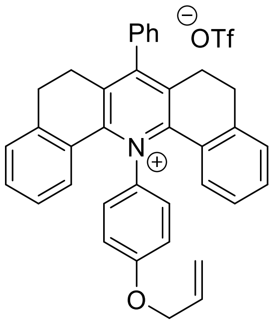

14-(4-(Allyloxy)phenyl)-7-phenyl-5,6,8,9-tetrahydrodibenzo[*c*,*h*]acridin-14-ium trifluoromethanesulfonate, 19

Prepared via general procedure **II** starting from **7** (0.77 g, 1.5 mmol) and 4-(allyloxy)aniline (prepared from *N*-(4-(allyloxy)phenyl)acetamide,[7] 0.34 g, 1.5 equiv.) to give **19** (0.92 g, 96%) as an off-white powder.

^{1}H NMR (400 MHz, CDCl$_3$): δ 7.58–7.48 (m, 3H), 7.45–7.39 (m, 2H), 7.36 (d, *J* = 8.9 Hz, 2H), 7.29–7.24 (m, 2H), 7.22 (t, *J* = 7.3 Hz, 2H), 6.94–6.86 (m, 4H), 6.62 (d, *J* = 8.1 Hz, 2H), 6.04 (ddt, *J* = 17.4, 10.5, 5.2 Hz, 1H), 5.43 (dq, *J* = 17.2, 1.6 Hz, 1H), 5.34 (dd, *J* = 10.5, 1.6 Hz, 1H), 4.58 (dt, *J* = 5.2, 1.6 Hz, 2H), 2.96–2.87 (m, 4H), 2.74–2.67 (m, 4H).

^{13}C NMR (101 MHz, DMSO-*d*$_6$): δ 159.6, 154.1, 148.0, 142.0, 138.1, 134.1, 133.4, 132.8, 130.6, 130.5, 129.7, 129.3, 129.1, 128.2, 127.9, 127.1, 125.9, 120.6 (q, *J* = 322.4 Hz, OTf), 117.8, 115.7, 68.6, 28.0, 27.3.

^{19}F NMR (470 MHz, DMSO-*d*$_6$): δ -77.7.

HRMS (ESI): calc'd for $C_{36}H_{30}NO^+$ [M-OTf]$^+$ 492.232189; found 492.232200.

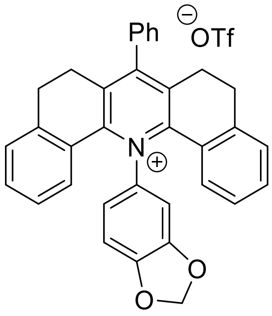

14-(Benzo[*d*][1,3]dioxol-5-yl)-7-phenyl-5,6,8,9-tetrahydrodibenzo[*c*,*h*]acridin-14-ium trifluoromethanesulfonate, 20

Prepared via general procedure **II** starting from **7** (0.77 g, 1.5 mmol) and 3,4-(Methylenedioxy)aniline (0.31 g, 1.5 equiv.) to give **20** (0.57 g, 89%) as a pale yellow powder.

^{1}H NMR (400 MHz, DMSO-*d*$_6$): δ 7.68–7.58 (m, 3H), 7.53–7.48 (m, 2H), 7.45 (d, *J* = 7.4 Hz, 2H), 7.37 (t, *J* = 7.4 Hz, 2H), 7.26 (d, *J* = 1.9 Hz, 1H), 7.15–7.06 (m, 3H), 7.03 (d, *J* = 8.3 Hz, 1H), 6.71 (d, *J* = 8.2 Hz, 2H), 6.18 (s, 2H), 2.95–2.84 (m, 4H), 2.77–2.67 (m, 4H).

^{13}C NMR (101 MHz, DMSO-*d*$_6$): δ 154.2, 149.2, 148.2, 148.1, 142.0, 138.2, 134.1, 134.0, 130.7, 129.8, 129.3, 128.9, 128.2, 128.0, 127.1, 126.1, 123.7, 120.7 (q, *J* = 322.2 Hz, OTf), 110.2, 108.7, 102.6, 28.0, 27.2.

^{19}F NMR (470 MHz, DMSO-*d*$_6$): δ -77.7.

HRMS (ESI): calc'd for $C_{34}H_{26}NO_2^+$ [M-OTf]$^+$ 480.195804; found 480.195780.

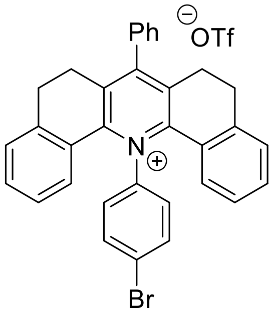

14-(4-Bromophenyl)-7-phenyl-5,6,8,9-tetrahydrodibenzo[*c*,*h*]acridin-14-ium trifluoromethanesulfonate, 21

Prepared via general procedure **II** starting from **7** (0.77 g, 1.5 mmol) and 4-bromoaniline (0.38 g, 1.5 equiv.) to give **21** (0.74 g, 74%) as a pale yellow powder. Sodium pivalate was used for this condensation.

^{1}H NMR (400 MHz, DMSO-*d*$_6$): δ 7.80–7.75 (m, 2H), 7.69–7.58 (m, 5H), 7.53–7.49 (m, 2H), 7.47 (d, *J* = 7.4 Hz, 2H), 7.37 (td, *J* = 7.5, 1.2 Hz, 2H), 7.04 (td, *J* = 7.8, 1.4 Hz, 2H), 6.51 (d, *J* = 8.0 Hz, 2H), 2.95–2.87 (m, 4H), 2.78–2.70 (m, 4H).

^{13}C NMR (101 MHz, DMSO-*d*$_6$): δ 154.3, 147.8, 142.2, 139.7, 138.3, 133.9, 133.0, 131.5, 130.8, 129.9, 129.3, 129.2, 128.2, 128.0, 126.8, 126.0, 124.3, 120.7 (q, *J* = 322.2 Hz, OTf), 27.9, 27.2.

^{19}F NMR (470 MHz, DMSO-*d*$_6$): δ -77.7.

HRMS (ESI): calc'd for $C_{33}H_{25}BrN^+$ [M-OTf]$^+$ 514.116499; found 514.116640.

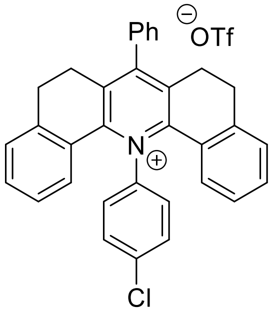

14-(4-Chlorophenyl)-7-phenyl-5,6,8,9-tetrahydrodibenzo[*c*,*h*]acridin-14-ium trifluoromethanesulfonate, 22

Prepared via general procedure **II** starting from **7** (0.51 g, 1.0 mmol) and 4-chloroaniline (0.19 g, 1.5 equiv.) to give **22** (0.563 g, 91%) as a pale yellow solid. Sodium pivalate (0.124 g, 1.5 mmol) was used for this condensation.

^{1}H NMR (400 MHz, DMSO-*d*$_6$): δ 7.70–7.59 (m, 7H), 7.53–7.48 (m, 2H), 7.46 (d, *J* = 7.3 Hz, 2H), 7.36 (td, *J* = 7.5, 1.2 Hz, 2H), 7.03 (td, *J* = 7.8, 1.4 Hz, 2H), 6.50 (d, *J* = 8.0 Hz, 2H), 2.95–2.86 (m, 4H), 2.78–2.70 (m, 4H).

^{13}C NMR (101 MHz, DMSO-*d*$_6$): δ 154.9, 148.4, 142.7, 139.8, 138.8, 136.2, 134.5, 131.8, 131.3, 130.5, 130.3, 129.8, 129.8, 128.7, 128.5, 127.3, 126.5, 121.1 (q, *J* = 322.4 Hz, OTf), 28.4, 27.7.

^{19}F NMR (470 MHz, DMSO-*d*$_6$): δ -77.7.

HRMS (ESI): calc'd for $C_{33}H_{25}ClN^+$ [M-OTf]$^+$ 470.167002; found 470.166980.

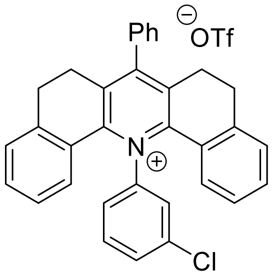

14-(3-Chlorophenyl)-7-phenyl-5,6,8,9-tetrahydrodibenzo[*c*,*h*]acridin-14-ium trifluoromethanesulfonate, 23

Prepared via general procedure **II** starting from **7** (0.77 g, 1.5 mmol) and 3-chloroaniline (0.29 g, 1.5 equiv.) to give **23** (0.85 g, 91%) as a pale yellow powder. Sodium pivalate was used for this condensation.

^{1}H NMR (400 MHz, DMSO-d_6): δ 7.84 (t, *J* = 1.8 Hz, 1H), 7.73 (d, *J* = 8.0 Hz, 1H), 7.69–7.59 (m, 4H), 7.56 (t, *J* = 8.1 Hz, 1H), 7.53–7.44 (m, 4H), 7.37 (t, *J* = 7.3 Hz, 2H), 7.02 (td, *J* = 7.8, 1.3 Hz, 2H), 6.49 (d, *J* = 8.0 Hz, 2H), 2.96–2.87 (m, 4H), 2.79–2.69 (m, 4H).

^{13}C NMR (101 MHz, DMSO-d_6): δ 154.5, 147.9, 142.2, 141.4, 138.3, 134.1, 134.0, 131.5, 131.2, 130.8, 129.8, 129.4, 129.3, 129.2, 128.5, 128.2, 128.2, 128.1, 126.7, 126.0, 120.64 (q, *J* = 322.3 Hz, OTf), 27.9, 27.2.

^{19}F NMR (470 MHz, DMSO-d_6): δ -77.7.

HRMS (ESI): calc'd for $C_{33}H_{25}ClN^+$ [M-OTf]$^+$ 470.167002; found 470.166980.

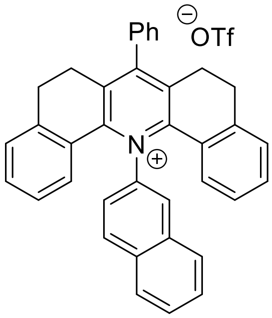

14-(Naphthalen-2-yl)-7-phenyl-5,6,8,9-tetrahydrodibenzo[*c*,*h*]acridin-14-ium trifluoromethanesulfonate, 24

Prepared via general procedure **II** starting from **7** (0.51 g, 1.0 mmol) and naphthalen-2-amine (0.22 g, 1.5 equiv.) to give **24** (0.57 g, 89%) as a pale yellow powder. Sodium pivalate was used for this condensation.

^{1}H NMR (400 MHz, DMSO-d_6): δ 8.24 (s, 1H), 8.07 (t, *J* = 8.2 Hz, 2H), 7.84 (d, *J* = 8.2 Hz, 1H), 7.74–7.57 (m, 6H), 7.54 (d, *J* = 6.4 Hz, 2H), 7.44 (d, *J* = 7.5 Hz, 2H), 7.24 (t, *J* = 7.5 Hz, 2H), 6.74 (t, *J* = 7.6 Hz, 2H), 6.43 (d, *J* = 8.1 Hz, 2H), 2.99–2.91 (m, 4H), 2.80–2.72 (m, 4H).

^{13}C NMR (101 MHz, DMSO-d_6): δ 154.3, 148.1, 142.1, 138.4, 137.8, 134.0, 133.0, 132.2, 130.6, 130.0, 129.8, 129.3, 129.3, 129.1, 128.8, 128.6, 128.5, 128.3, 128.2, 128.0, 128.0, 127.8, 127.0, 126.1, 125.8, 120.7 (q, *J* = 322.6 Hz, OTf), 27.9, 27.3.

^{19}F NMR (470 MHz, DMSO-d_6): δ -77.7.

HRMS (ESI): calc'd for $C_{37}H_{28}N^+$ [M-OTf]$^+$ 486.221624; found 486.221580.

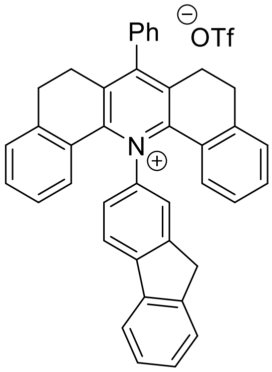

14-(9*H*-Fluoren-2-yl)-7-phenyl-5,6,8,9-tetrahydrodibenzo[*c*,*h*]acridin-14-ium trifluoromethanesulfonate, 25

Prepared via general procedure **II** starting from **7** (0.77 g, 1.5 mmol) and 2-fluorenamine (0.41 g, 1.5 equiv.) to give **25** (0.89 g, 88%) as a pale brown powder. Sodium pivalate was used for this condensation.

^{1}H NMR (400 MHz, DMSO-*d*$_6$): δ 8.03 (t, *J* = 7.0 Hz, 2H), 7.81 (s, 1H), 7.69–7.59 (m, 5H), 7.56–7.50 (m, 2H), 7.50–7.39 (m, 4H), 7.28 (t, *J* = 7.3 Hz, 2H), 6.88 (t, *J* = 7.5 Hz, 2H), 6.58 (d, *J* = 8.1 Hz, 2H), 3.88 (s, 2H), 2.98–2.89 (m, 4H), 2.80–2.70 (m, 4H).

^{13}C NMR (101 MHz, DMSO-*d*$_6$): δ 154.2, 148.0, 144.4, 143.9, 143.4, 142.0, 139.4, 139.0, 138.3, 134.1, 130.6, 129.7, 129.3, 129.2, 128.3, 128.2, 128.0, 127.9, 127.1, 126.1, 125.9, 125.4, 121.1, 120.7 (q, *J* = 322.4 Hz, OTf), 36.6, 28.0, 27.3.

^{19}F NMR (470 MHz, DMSO-*d*$_6$): δ -77.7.

HRMS (ESI): calc'd for $C_{40}H_{30}N^+$ [M-OTf]$^+$ 524.237274; found 524.237260.

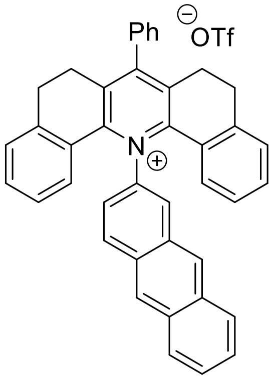

14-(Anthracen-2-yl)-7-phenyl-5,6,8,9-tetrahydrodibenzo[*c*,*h*]acridin-14-ium trifluoromethanesulfonate, 26

Prepared via general procedure **II** starting from **7** (0.77 g, 1.5 mmol) and anthracen-2-amine (0.43 g, 1.5 equiv.) to give **26** (0.95 g, 92%) as a yellow green powder. Sodium pivalate was used for this condensation.

^{1}H NMR (400 MHz, DMSO-*d*$_6$): δ 8.73 (s, 1H), 8.57 (s, 1H), 8.43 (s, 1H), 8.25 (d, *J* = 9.1 Hz, 1H), 8.16 (d, *J* = 7.9 Hz, 1H), 8.11 (d, *J* = 7.9 Hz, 1H), 7.71–7.57 (m, 6H), 7.57–7.51 (m, 2H), 7.45 (d, *J* = 7.5 Hz, 2H), 7.22 (t, *J* = 7.4 Hz, 2H), 6.71 (t, *J* = 7.6 Hz, 2H), 6.58 (d, *J* = 8.1 Hz, 2H), 3.02–2.92 (m, 4H), 2.83–2.73 (m, 4H).

^{13}C NMR (101 MHz, DMSO-*d*$_6$): δ 154.3, 148.1, 142.1, 138.4, 137.4, 134.1, 132.5, 131.9, 130.7, 130.5, 130.2, 129.8, 129.6, 129.5, 129.3, 129.3, 129.2, 128.3, 128.2, 128.2, 128.1, 128.0, 127.9, 127.0, 126.9, 126.9, 126.7, 125.8, 125.4, 120.7 (q, *J* = 322.4 Hz, OTf), 27.9, 27.3.

^{19}F NMR (470 MHz, DMSO-*d*$_6$): δ -77.7.

HRMS (ESI): calc'd for $C_{41}H_{30}N^+$ [M-OTf]$^+$ 536.237274; found 536.236940.

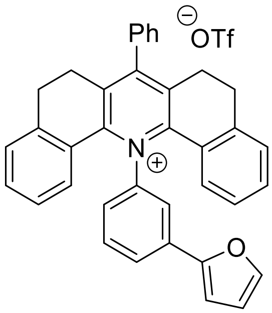

14-(3-(Furan-2-yl)phenyl)-7-phenyl-5,6,8,9-tetrahydrodibenzo[*c*,*h*]acridin-14-ium trifluoromethanesulfonate, 27

Prepared via general procedure **II** starting from **7** (1.28 g, 2.5 mmol) and 3-(furan-2-yl)aniline[8] (0.60 g, 3.75 mmol) to give **27** (1.46 g, 90%) as a green solid. Sodium pivalate (0.31 g, 2.5 mmol) was used for this condensation.

^{1}H NMR (400 MHz, DMSO-*d*$_6$): δ 8.09 (s, 1H), 7.92–7.83 (m, 2H), 7.72 (s, 1H), 7.70–7.59 (m, 3H), 7.58–7.47 (m, 4H), 7.45 (d, *J* = 7.4 Hz, 2H), 7.31 (t, *J* = 7.4 Hz, 2H), 6.96 (t, *J* = 7.6 Hz, 2H), 6.75 (s, 1H), 6.60 (d, *J* = 8.1 Hz, 2H), 3.00–2.86 (m, 4H), 2.75 (t, *J* = 6.6 Hz, 4H).

^{13}C NMR (101 MHz, DMSO-*d*$_6$): δ 154.4, 147.9, 144.7, 142.1, 141.0, 140.2, 138.3, 134.0, 133.9, 130.7, 130.3, 129.8, 129.3, 129.2, 128.2, 128.0, 127.8, 127.6, 126.9, 126.2, 125.9, 124.3, 120.7 (q, *J* = 322.4 Hz, OTf), 108.4, 27.9, 27.3.

^{19}F NMR (470 MHz, DMSO-*d*$_6$): δ -77.8.

HRMS (ESI): calc'd for $C_{37}H_{28}NO^+$ [M-OTf]$^+$ 502.216539; found 502.216450.

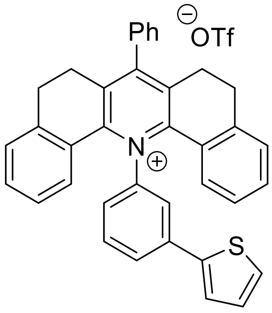

7-Phenyl-14-(3-(thiophen-2-yl)phenyl)-5,6,8,9-tetrahydrodibenzo[*c*,*h*]acridin-14-ium trifluoromethanesulfonate, 28

Prepared via general procedure **II** starting from **7** (1.12 g, 2.2 mmol) and 3-(thiophen-2-yl)aniline[8] (0.58 g, 3.3 mmol) to give **28** (1.25 g, 85%) as a green solid. Sodium pivalate (0.27 g, 2.2 mmol) was used for this condensation.

^{1}H NMR (400 MHz, DMSO-*d*$_6$): δ 7.97–7.93 (m, 1H), 7.89 (dt, *J* = 6.9, 1.9 Hz, 1H), 7.69–7.60 (m, 3H), 7.58 (dd, *J* = 5.1, 1.0 Hz, 1H), 7.56–7.48 (m, 4H), 7.45 (d, *J* = 7.4 Hz, 2H), 7.39 (dd, *J* = 3.7, 1.2 Hz, 1H), 7.32 (td, *J* = 7.5, 1.2 Hz, 2H), 7.12 (dd, *J* = 5.1, 3.6 Hz, 1H), 6.97 (td, *J* = 7.8, 1.3 Hz, 2H), 6.61 (d, *J* = 8.0 Hz, 2H), 3.00–2.87 (m, 4H), 2.75 (t, *J* = 6.7 Hz, 4H).

^{13}C NMR (101 MHz, DMSO-*d*$_6$): δ 154.3, 147.9, 142.1, 141.1, 141.1, 138.2, 135.4, 134.0, 130.7, 130.6, 129.8, 129.3, 129.3, 128.7, 128.2, 128.2, 128.0, 128.0, 127.5, 127.2, 126.9, 126.1, 125.9, 125.1, 120.7 (q, *J* = 322.3 Hz, OTf), 27.9, 27.3.

^{19}F NMR (470 MHz, DMSO-*d*$_6$): δ -77.7.

HRMS (ESI): calc'd for $C_{37}H_{28}NS^{+}$ [M-OTf]$^{+}$ 518.193696; found 518.193690.

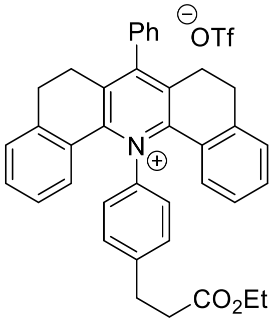

14-(4-(3-Ethoxy-3-oxopropyl)phenyl)-7-phenyl-5,6,8,9-tetrahydrodibenzo[*c*,*h*]acridin-14-ium trifluoromethanesulfonate, 29

Prepared via general procedure **II** starting from **7** (0.77 g, 1.5 mmol) and ethyl 3-(4-aminophenyl)propanoate (0.43 g, 1.5 equiv.) to give **29** (0.84 g, 82%) as an off-white powder.

^{1}H NMR (400 MHz, DMSO-*d*$_6$): δ 7.68–7.58 (m, 3H), 7.54–7.47 (m, 4H), 7.44 (d, *J* = 7.3 Hz, 2H), 7.37 (d, *J* = 8.4 Hz, 2H), 7.33 (td, *J* = 7.5, 1.2 Hz, 2H), 6.92 (td, *J* = 7.8, 1.4 Hz, 2H), 6.46 (d, *J* = 8.0 Hz, 2H), 4.06 (q, *J* = 7.1 Hz, 2H), 2.98–2.85 (m, 6H), 2.76–2.70 (m, 4H), 2.67 (t, *J* = 7.3 Hz, 2H), 1.17 (t, *J* = 7.1 Hz, 3H).

^{13}C NMR (101 MHz, DMSO-*d*$_6$): δ 171.7, 154.2, 147.9, 144.2, 142.0, 138.6, 138.2, 134.1, 130.6, 129.9, 129.8, 129.3, 129.2, 129.2, 128.2, 127.9, 127.0, 125.8, 120.7 (q, *J* = 322.4 Hz, OTf), 59.9, 35.1, 29.9, 27.9, 27.3, 14.1.

^{19}F NMR (470 MHz, DMSO-*d*$_6$): δ -77.8.

HRMS (ESI): calc'd for $C_{38}H_{34}NO_2^+$ [M-OTf]$^+$ 536.258404; found 536.258440.

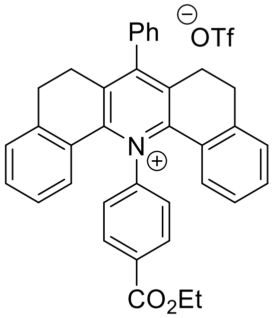

14-(4-(Ethoxycarbonyl)phenyl)-7-phenyl-5,6,8,9-tetrahydrodibenzo[*c*,*h*]acridin-14-ium trifluoromethanesulfonate, 30

Prepared via general procedure **II** starting from **7** (0.77 g, 1.5 mmol) and ethyl 4-aminobenzoate (0.37 g, 1.5 equiv.) to give **30** (0.82 g, 83%) as a pale yellow powder. Sodium pivalate was used for this condensation. The crude product was purified by flash chromatography [DCM/Acetone (5/1 to 2/1, *v*/*v*)].

^{1}H NMR (300 MHz, DMSO-*d*$_6$): δ 8.11–8.03 (m, 2H), 7.84–7.77 (m, 2H), 7.70–7.58 (m, 3H), 7.55–7.49 (m, 2H), 7.46 (d, *J* = 7.3 Hz, 2H), 7.34 (td, *J* = 7.5, 1.1 Hz, 2H), 6.96 (td, *J* = 7.8, 1.4 Hz, 2H), 6.46 (d, *J* = 7.9 Hz, 2H), 4.36 (q, *J* = 7.1 Hz, 2H), 2.97–2.88 (m, 4H), 2.79–2.70 (m, 4H), 1.35 (t, *J* = 7.1 Hz, 3H).

^{13}C NMR (101 MHz, DMSO-*d*$_6$): δ 164.5, 154.5, 147.8, 144.0, 142.2, 138.4, 134.0, 131.7, 130.8, 130.6, 130.2, 129.8, 129.3 (2C), 128.2, 128.0, 126.7, 126.0, 120.6 (q, *J* = 322.5 Hz), 61.5, 27.9, 27.2, 14.0.

^{19}F NMR (470 MHz, DMSO-*d*$_6$): δ -77.7.

HRMS (ESI): calc'd for $C_{36}H_{30}NO_2^+$ [M-OTf]$^+$ 508.227104; found 508.227190.

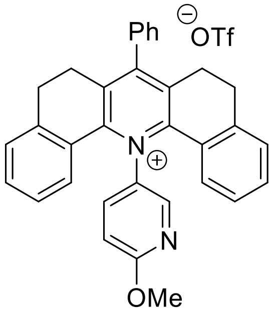

14-(6-Methoxypyridin-3-yl)-7-phenyl-5,6,8,9-tetrahydrodibenzo[*c*,*h*]acridin-14-ium trifluoromethanesulfonate, 31

Prepared via general procedure **II** starting from **7** (0.77 g, 1.5 mmol) and 6-methoxypyridin-3-amine (0.28 g, 1.5 equiv.) to give **31** (0.75 g, 81%) as an off-white powder. Sodium pivalate was used for this condensation.

^{1}H NMR (400 MHz, DMSO-*d*$_6$): δ 8.36 (d, *J* = 2.7 Hz, 1H), 7.91 (dd, *J* = 8.9, 2.8 Hz, 1H), 7.68–7.60 (m, 3H), 7.54–7.49 (m, 2H), 7.48 (d, *J* = 7.5 Hz, 2H), 7.37 (td, *J* = 7.5, 1.2 Hz, 2H), 7.08 (td, *J* = 7.8, 1.4 Hz, 2H), 7.01 (d, *J* = 8.9 Hz, 1H), 6.53 (d, *J* = 8.0 Hz, 2H), 3.91 (s, 3H), 2.91 (t, *J* = 6.3 Hz, 4H), 2.79–2.71 (m, 4H).

^{13}C NMR (101 MHz, DMSO-*d*$_6$): δ 163.9, 154.4, 148.2, 147.0, 142.4, 139.8, 138.3, 133.9, 132.0, 130.8, 129.9, 129.6, 129.3, 128.2, 128.2, 128.1, 126.7, 126.1, 120.6 (q, *J* = 322.3 Hz, OTf), 111.6, 54.1, 27.9, 27.3.

^{19}F NMR (470 MHz, DMSO-*d*$_6$): δ -77.7.

HRMS (ESI): calc'd for $C_{33}H_{27}N_2O^+$ [M-OTf]$^+$ 467.211788; found 467.211500.

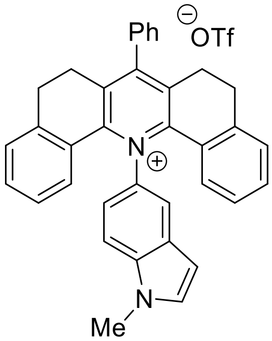

14-(1-Methyl-1*H*-indol-5-yl)-7-phenyl-5,6,8,9-tetrahydrodibenzo[*c*,*h*]acridin-14-ium trifluoromethanesulfonate, 32

Prepared via general procedure **II** starting from **7** (0.77 g, 1.5 mmol) and 1-methyl-1*H*-indol-5-amine (prepared from 5-nitro-1*H*-indole in two steps[9], 0.33 g, 1.5 equiv.) to give **32** (0.82 g, 86%) as a pale yellow powder. Sodium pivalate was used for this condensation.

^{1}H NMR (400 MHz, DMSO-*d*$_6$): δ 7.80 (d, *J* = 2.0 Hz, 1H), 7.68–7.59 (m, 3H), 7.57 (d, *J* = 8.7 Hz, 1H), 7.55–7.49 (m, 3H), 7.41 (d, *J* = 7.4 Hz, 2H), 7.31 (dd, *J* = 8.7, 2.0 Hz, 1H), 7.24 (td, *J* = 7.5, 1.2 Hz, 2H), 6.78 (t, *J* = 7.4 Hz, 2H), 6.49 (d, *J* = 2.9 Hz, 1H), 6.43 (d, *J* = 8.1 Hz, 2H), 3.85 (s, 3H), 2.97–2.88 (m, 4H), 2.78–2.68 (m, 4H).

^{13}C NMR (101 MHz, DMSO-*d*$_6$): δ 154.0, 148.3, 141.8, 138.1, 136.5, 134.2, 132.7, 132.5, 130.3, 129.7, 129.3, 128.9, 128.2, 127.8, 127.7, 127.5, 125.8, 121.5, 121.4, 120.7 (q, *J* = 321.8 Hz, OTf), 110.9, 101.9, 32.8, 28.1, 27.3.

^{19}F NMR (470 MHz, DMSO-*d*$_6$): δ -77.7.

HRMS (ESI): calc'd for $C_{36}H_{29}N_2^+$ [M-OTf]$^+$ 489.232522; found 489.232200.

2.3 Preparation of *N,N*-diisopropylacetamide (solvent)

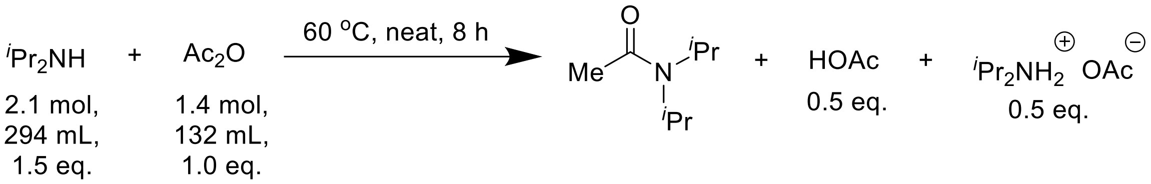

Modification of the synthetic procedure of *N,N*-diisopropylacetamide[10] has been made to be applied for large scale preparation: to a 1L double-necked flask equipped with dropping funnel and bubbler, diisopropylamine (294 mL, 2.1 mol, 1.5 equiv.) was added. With water bath cooling, acetic anhydride was added via dropping funnel. The reaction released large amount of heat and the ammonium salt precipitated soon as colorless needle crystal. The reaction mixture was heated to 60 °C for 8 h. During the addition of acetic anhydride, the mixture became pale yellow and clear.

Work-up: After the reaction mixture cooled down, water (200 mL) was added to the mixture to dissolve the ammonium salt. The aqueous layer was extracted with Et_2O (450 mL). The organic layer was washed by water (200 mL), added into 1L one-necked flask and neutralized with saturated Na_2CO_3 solution under stirring. CO_2 was released and a large amount of white solid crushed out from the aqueous phase. Crude ^{1}H NMR showed that ammonium salt and HOAc were completely removed. The organic layer was dried by Na_2SO_4 and the Et_2O was evaporated *in vacuo*. CaH_2 was added to the crude product portion by portion until no H_2 was released (*Caution: certain amount of water remains in crude product due to its hydrophilicity.CaH$_2$ should be added cautiously!*). Vapor distillation (35-38 mmbar, oil bath temperature: 130 °C, boiling point: ca. 98 °C) gave pure product (180 mL, 78%) as colorless liquid. The product was kept in glove box for storage.

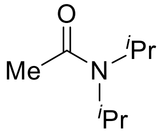

***N*,*N*-diisopropylacetamide**

^{1}H NMR (300 MHz, $CDCl_3$): δ 3.62–3.44 (m, 1H), 3.62–3.44 (m, 1H), 2.06 (s, 3H), 1.36 (d, J = 6.8 Hz, 6H), 1.20 (d, J = 6.7 Hz, 6H).

^{13}C NMR (101 MHz, $CDCl_3$): δ 169.6, 49.4 (br), 45.6, 24.1, 21.1, 20.8.

3. Optimization Studies

General Procedure for Optimization

A dry 10 mL-reaction vial equipped with a stir bar was charged with promoter, and then transferred to an argon-filled glove box. Aryl pyridinium salt (0.1 mmol), boron sources (1.5-3.0 equiv) and dry solvent (0.5 mL) were added successively. The vial was sealed and taken out of the glovebox. The resulting mixture was stirred at 120 °C (temperature of oil bath) for 24 h. After this time, the vial was cooled to room temperature, then Pinacol (3.0-6.0 equiv) and dry Et_3N (0.5 mL) were added and stirred at r.t. for 2 h. After completion of the reaction, the solution was diluted with ethyl acetate (~10 mL). The yield was confirmed by GC-FID using dodecane as an internal standard.

Promoter Screening

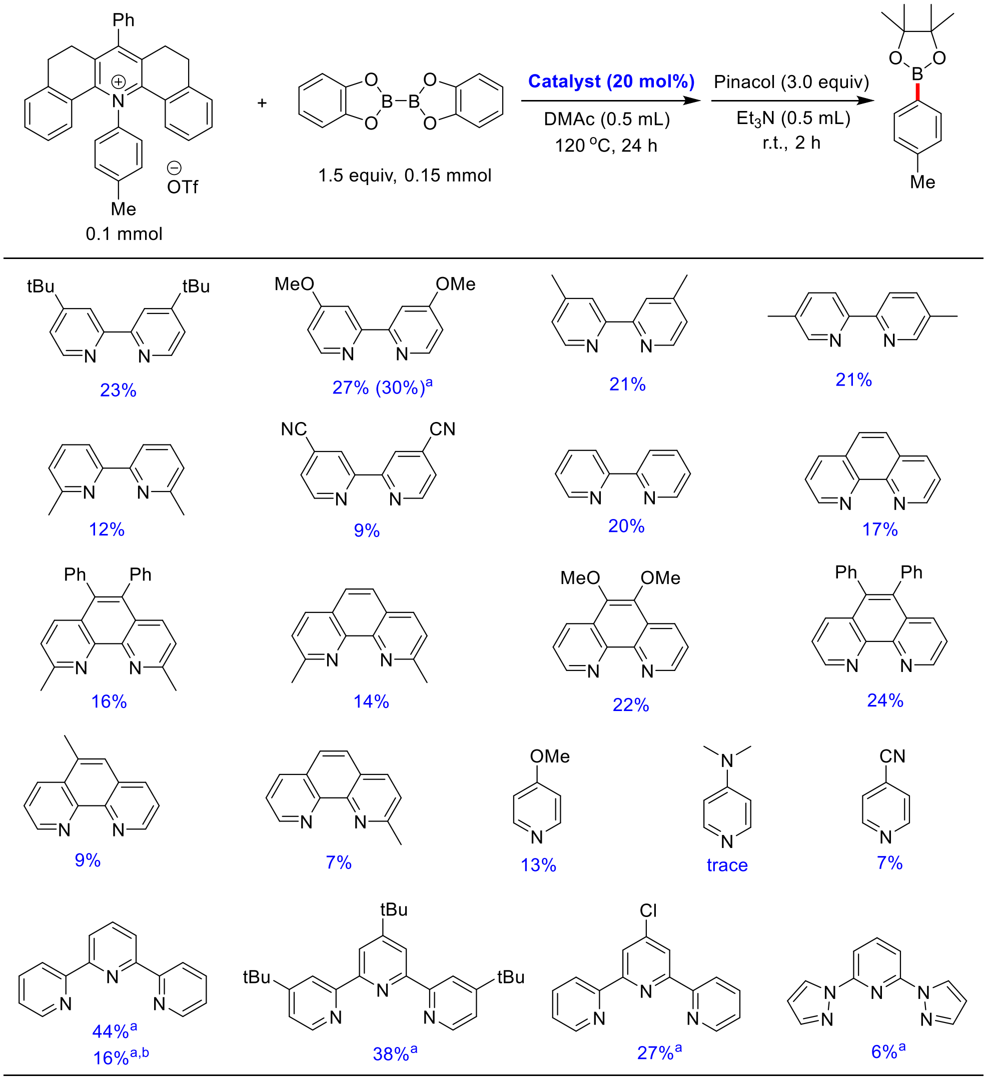

[a]B_2Cat_2 (2.5 equiv) and Pinacol (5.0 equiv) were used. [b]10 mol% catalyst loading. No desired product was detected in absence of catalyst.

Evaluation of Boron Sources

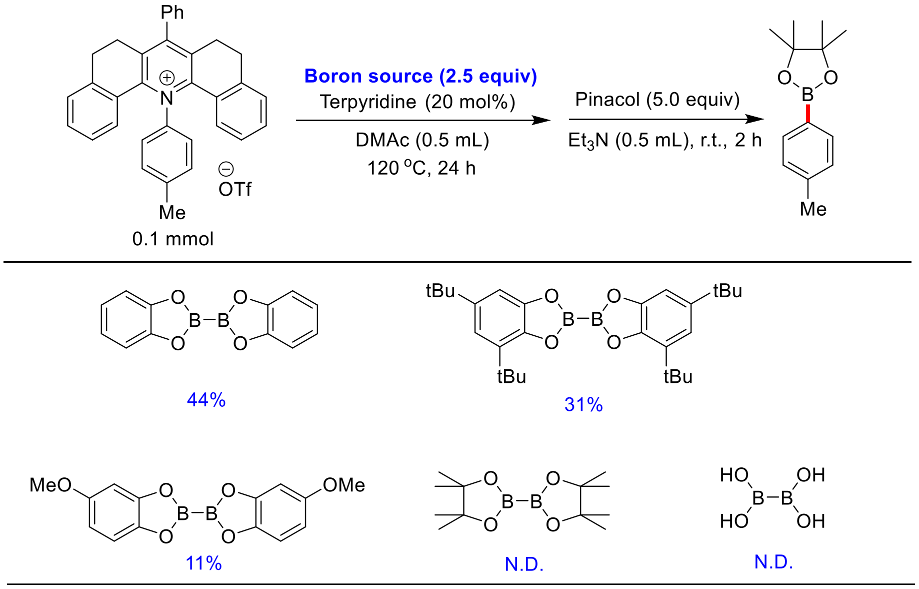

N.D. = Not detected

Solvent Screening

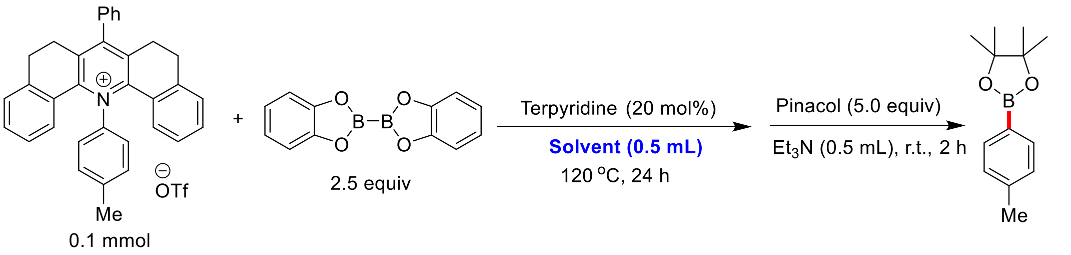

Dioxane	DMSO	DMF	DMAc	NMP	DMPU	DiPrAc
7%	N.D.	22%, 37%[a,b]	44%, 49%[a,b]	28%	17%	68%, 82%[a,b]

[a]3.0 equiv B_2Cat_2 and 6.0 equiv pinacol were used. [b]130 °C N.D. = Not detected

DMSO = Dimethyl sulfoxide DMF = *N*,*N*-Dimethylformamide DMAc = *N*,*N*-Dimethylacetamide

NMP = 1-Methyl-2-pyrrolidinone DMPU = *N*,*N'*-dimethylpropylene urea DiPrAc = *N*,*N*-diisopropylacetamide

Evaluation of Structures of Pyridinium Salts

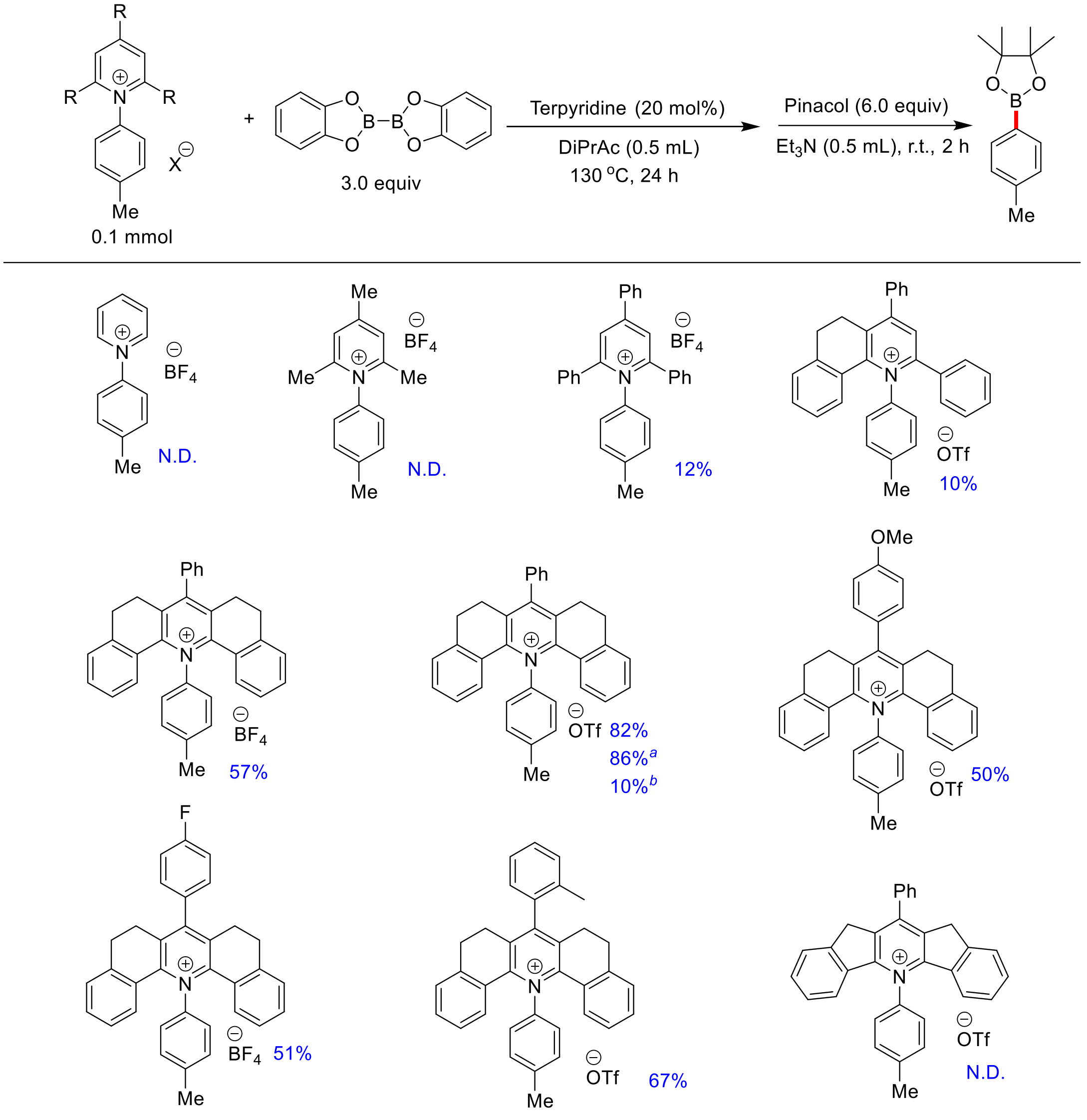

[a]The reaction was performed at 0.25 mmol scale at 130 °C for 12 h and quenched by methyliminodiacetic acid (6.0 equiv) at 90 °C for 4 h. Yield of isolated product and product is MeC_6H_5BMIDA. [b]Without catalyst
N.D. = Not detected DiPrAc = *N*,*N*-diisopropylacetamide

4. General Procedure for C–N Borylation of Aromatic Amines

A dry 10 mL-reaction vial equipped with a stir bar was charged with terpyridine (11.7 mg, 0.05 mmol), and then transferred to an argon-filled glove box. Aryl pyridinium salt (0.25 mmol), bis(catecholato)diboron (178.5 mg, 0.75 mmol) and DiPrAc (0.2 M, 1.25 mL) were added successively. The vial was sealed and taken out of the glovebox. The resulting mixture was stirred at 130 °C (temperature of oil bath) for 12 h. After this time, the vial was cooled to room temperature. Then methyliminodiacetic acid (220.7 mg, 1.5 mmol) and dry DMF (1.0 mL) were added under argon and the reaction mixture was heated to 90 °C for 4 h, after which solvent DiPrAc and DMF were removed under high vacuum. The resulting crude mixture was purified by column chromatography on silica gel using an elution gradient from MTBE (100%) to MTBE/Acetone (4/1, v/v) to afford the desired borylation product.

5. Characterization Data

***Note**: The signal of the α-B-carbon was not observed due to quadrupolar broadening.*[11]

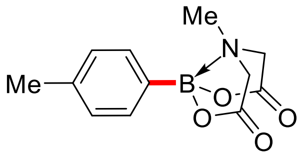

6-Methyl-2-(*p*-tolyl)-1,3,6,2-dioxazaborocane-4,8-dione, 6

Following the general procedure, purification via column chromatography on silica gel [MTBE (100%) to MTBE/Acetone (4/1, *v*/*v*)] afforded **6** as a pale yellow solid (53.1 mg, 86% yield).

^{1}H NMR (400 MHz, Acetone-*d$_6$*): δ 7.42 (d, *J* = 8.0 Hz, 2H), 7.18 (d, *J* = 7.6 Hz, 2H), 4.32 (d, *J* = 16.8 Hz, 2H), 4.11 (d, *J* = 17.2 Hz, 2H), 2.70 (s, 3H), 2.32 (s, 3H).

^{13}C NMR (101 MHz, Acetone-*d$_6$*): δ 169.3, 139.4, 133.3, 129.3, 62.6, 48.2, 21.4. The signal of the α-B-carbon was not observed.

^{11}B NMR (128 MHz, Acetone-*d$_6$*): δ 11.8.

HRMS (ESI): calc'd for $C_{12}H_{14}BN NaO_4^+$ $[M+Na]^+$ 270.090808; found 270.090670.

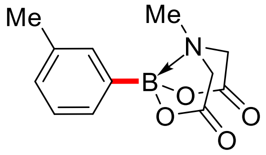

6-Methyl-2-(*m*-tolyl)-1,3,6,2-dioxazaborocane-4,8-dione, 33

Following the general procedure, purification via column chromatography on silica gel [MTBE (100%) to MTBE/Acetone (4/1, *v*/*v*)] afforded **33** as a pale yellow solid (51.3 mg, 83% yield).

^{1}H NMR (400 MHz, Acetone-*d$_6$*): δ 7.37 (s, 1H), 7.32 (d, *J* = 7.2 Hz, 1H), 7.25 (t, *J* = 7.2 Hz, 1H), 7.20–7.17 (m, 1H), 4.33 (d, *J* = 16.8 Hz, 2H), 4.12 (d, *J* = 17.2 Hz, 2H), 2.71 (s, 3H), 2.32 (s, 3H).

^{13}C NMR (101 MHz, Acetone-*d$_6$*): δ 169.3, 137.7, 134.0, 130.5, 130.4, 128.5, 62.7, 48.2, 21.5. The signal of the α-B-carbon was not observed.

^{11}B NMR (128 MHz, Acetone-*d$_6$*): δ 11.6.

HRMS (ESI): calc'd for $C_{12}H_{15}BNO_4^+$ $[M+H]^+$ 248.108864; found 248.108820.

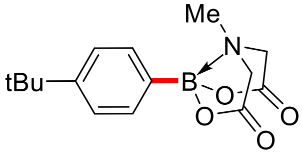

2-(4-(*tert*-Butyl)phenyl)-6-methyl-1,3,6,2-dioxazaborocane-4,8-dione, 34

Following the general procedure, purification via column chromatography on silica gel [MTBE (100%) to MTBE/Acetone (4/1, *v*/*v*)] afforded **34** as a pale yellow solid (53.5 mg, 74% yield).

^{1}H NMR (400 MHz, Acetone-*d_6*): δ 7.48–7.46 (m, 2H), 7.43–7.40 (m, 2H), 4.32 (d, *J* = 16.8 Hz, 2H), 4.12 (d, *J* = 17.2 Hz, 2H), 2.72 (s, 3H), 1.31 (s, 9H).

^{13}C NMR (101 MHz, Acetone-*d_6*): δ 169.3, 152.5, 133.2, 125.4, 62.6, 48.1, 35.1, 31.6. The signal of the α-B-carbon was not observed.

^{11}B NMR (128 MHz, Acetone-*d_6*): δ 11.7.

HRMS (ESI): calc'd for $C_{15}H_{21}BNO_4^+$ $[M+H]^+$ 290.155814; found 290.155570.

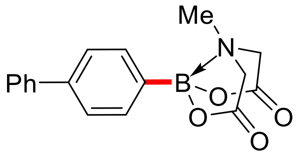

2-([1,1'-Biphenyl]-4-yl)-6-methyl-1,3,6,2-dioxazaborocane-4,8-dione, 35

Following the general procedure, purification via column chromatography on silica gel [MTBE (100%) to MTBE/Acetone (4/1, *v*/*v*)] afforded **35** as a pale yellow solid (63.4 mg, 82% yield).

^{1}H NMR (400 MHz, Acetone-*d_6*): δ 7.69–7.62 (m, 6H), 7.49–7.44 (m, 2H), 7.38–7.34 (m, 1H), 4.37 (d, *J* = 17.2 Hz, 2H), 4.18 (d, *J* = 17.2 Hz, 2H), 2.79 (s, 3H).

^{13}C NMR (101 MHz, Acetone-*d_6*): δ 169.3, 142.3, 141.7, 134.0, 129.7, 128.2, 127.7, 127.1, 62.8, 48.3. The signal of the α-B-carbon was not observed.

^{11}B NMR (128 MHz, Acetone-*d_6*): δ 11.7.

HRMS (ESI): calc'd for $C_{17}H_{17}BNO_4^+$ $[M+H]^+$ 310.124514; found 310.124380.

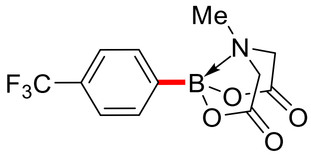

6-Methyl-2-(4-(trifluoromethyl)phenyl)-1,3,6,2-dioxazaborocane-4,8-dione, 36

Following the general procedure, bis(catecholato)diboron (4.0 equiv) was used and the borylation reaction was performed at 130 °C for 24 h. Purification via column chromatography on silica gel [MTBE (100%) to MTBE/Acetone (4/1, v/v)] afforded **36** as a pale yellow solid (46.5 mg, 62% yield).

^{1}H NMR (400 MHz, Acetone-*d$_6$*): δ 7.79 (d, *J* = 8.0 Hz, 2H), 7.72 (d, *J* = 8.0 Hz, 2H), 4.43 (d, *J* = 17.2 Hz, 2H), 4.23 (d, *J* = 16.8 Hz, 2H), 2.81 (s, 3H).

^{13}C NMR (101 MHz, Acetone-*d$_6$*): δ 169.1, 134.2, 131.3 (d, *J* = 31.8 Hz), 125.5 (d, *J* = 272.8 Hz), 125.1 (q, *J* = 4.3 Hz), 62.9, 48.4. The signal of the α-B-carbon was not observed.

^{11}B NMR (128 MHz, Acetone-*d$_6$*): δ 11.1.

^{19}F NMR (282 MHz, Acetone-*d$_6$*) : δ -63.2.

HRMS (ESI): calc'd for $C_{12}H_{11}BF_3NNaO_4^+$ $[M+Na]^+$ 324.062543; found 324.062220.

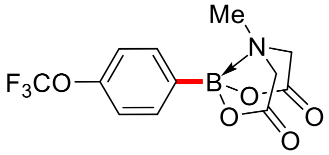

6-Methyl-2-(4-(trifluoromethoxy)phenyl)-1,3,6,2-dioxazaborocane-4,8-dione, 37

Following the general procedure, bis(catecholato)diboron (4.0 equiv) was used and the borylation reaction was performed at 130 °C for 24 h. Purification via column chromatography on silica gel [MTBE (100%) to MTBE/Acetone (4/1, *v*/*v*)] afforded **37** as a pale yellow solid (71.8 mg, 91% yield).

^{1}H NMR (400 MHz, Acetone-*d$_6$*): δ 7.68–7.66 (m, 2H), 7.33–7.30 (m, 2H), 4.39 (d, *J* = 17.2 Hz, 2H), 4.19 (d, *J* = 16.8 Hz, 2H), 2.78 (s, 3H).

^{13}C NMR (101 MHz, Acetone-*d$_6$*): δ 169.2, 150.8 (q, *J* = 1.6 Hz), 135.4, 121.4 (q, *J* = 256.3 Hz), 120.9, 62.8, 48.3. The signal of the α-B-carbon was not observed.

^{11}B NMR (128 MHz, Acetone-*d$_6$*): δ 11.3.

^{19}F NMR (471 MHz, Acetone-*d$_6$*) : δ -58.4.

HRMS (ESI): calc'd for $C_{12}H_{12}BF_3NO_5^+$ $[M+H]^+$ 318.075514; found 318.075740.

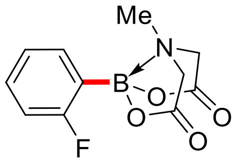

2-(2-Fluorophenyl)-6-methyl-1,3,6,2-dioxazaborocane-4,8-dione, 38

Following the general procedure, purification via column chromatography on silica gel [MTBE (100%) to MTBE/Acetone (4/1, *v*/*v*)] afforded **38** as a pale yellow solid (45.2 mg, 72% yield).

^{1}H NMR (400 MHz, Acetone-*d$_6$*): δ 7.61 (td, *J* = 7.2 Hz, 1.6 Hz, 1H), 7.48–7.42 (m, 1H), 7.21 (tt, *J* = 7.2 Hz, 0.8 Hz, 1H), 7.10–7.05 (m, 1H), 4.42 (dd, *J* = 16.8 Hz, 1.6 Hz, 2H), 4.17 (d, *J* = 17.2 Hz, 2H), 2.87 (s, 3H).

^{13}C NMR (101 MHz, Acetone-*d$_6$*): δ 168.9, 167.0 (d, *J* = 241.3 Hz), 135.9 (d, *J* = 9.3 Hz), 132.5 (d, *J* = 8.8 Hz), 125.0 (d, *J* = 2.9 Hz), 115.7 (d, *J* = 25.1 Hz), 63.5 (d, *J* = 2.7 Hz), 48.2. The signal of the α-B-carbon was not observed.

^{11}B NMR (128 MHz, Acetone-*d$_6$*): δ 11.3.

^{19}F NMR (471 MHz, Acetone-*d^6*) : δ (-106.74)–(-106.69) (m, 1F).

HRMS (ESI): calc'd for $C_{11}H_{11}BFNNaO_4^+$ [M+Na]$^+$ 274.065736; found 274.065780.

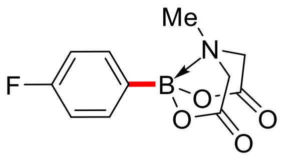

2-(4-Fluorophenyl)-6-methyl-1,3,6,2-dioxazaborocane-4,8-dione, 39

Following the general procedure, purification via column chromatography on silica gel [MTBE (100%) to MTBE/Acetone (4/1, *v*/*v*)] afforded **39** as a pale yellow solid (53.2 mg, 85% yield).

^{1}H NMR (400 MHz, Acetone-*d$_6$*): δ 7.62–7.57 (m, 2H), 7.17–7.11 (m, 2H), 4.37 (d, *J* = 17.2 Hz, 2H), 4.17 (d, *J* = 17.2 Hz, 2H), 2.77 (s, 3H).

^{13}C NMR (101 MHz, Acetone-*d$_6$*): δ 169.2, 164.6 (d, *J* = 246.3 Hz), 135.6 (d, *J* = 7.7 Hz), 115.4 (d, *J* = 20.1 Hz), 62.8, 48.3. The signal of the α-B-carbon was not observed.

^{11}B NMR (128 MHz, Acetone-*d$_6$*): δ 11.4.

^{19}F NMR (282 MHz, Acetone-*d$_6$*) : δ -114.3.

HRMS (ESI): calc'd for $C_{11}H_{12}BFNO_4^+$ [M+H]$^+$ 252.083792; found 252.086471.

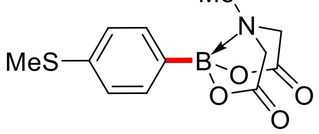

6-Methyl-2-(4-(methylthio)phenyl)-1,3,6,2-dioxazaborocane-4,8-dione, 40

Following the general procedure, purification via column chromatography on silica gel [MTBE (100%) to MTBE/Acetone (4/1, *v*/*v*)] afforded **40** as a pale yellow solid (52.3 mg, 75% yield).

^{1}H NMR (400 MHz, Acetone-d_6): δ 7.46 (d, J = 8.4 Hz, 2H), 7.28–7.25 (m, 2H), 4.33 (d, J = 16.8 Hz, 2H), 4.13 (d, J = 17.2 Hz, 2H), 2.74 (s, 3H), 2.49 (s, 3H).

^{13}C NMR (101 MHz, Acetone-d_6): δ 169.3, 140.7, 133.9, 126.1, 62.7, 48.2, 15.0. The signal of the α-B-carbon was not observed.

^{11}B NMR (128 MHz, Acetone-d_6): δ 11.6.

HRMS (ESI): calc'd for $C_{12}H_{14}BNNaO_4S^+$ $[M+Na]^+$ 302.062880; found 302.062910.

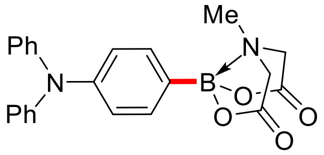

2-(4-(Diphenylamino)phenyl)-6-methyl-1,3,6,2-dioxazaborocane-4,8-dione, 41

Following the general procedure, purification via column chromatography on silica gel [MTBE (100%) to MTBE/Acetone (4/1, *v*/*v*)] afforded **41** as a pale yellow solid (69.6 mg, 70% yield).

^{1}H NMR (400 MHz, Acetone-d_6): δ 7.45–7.42 (m, 2H), 7.31–7.26 (m, 4H), 7.07–7.01 (m, 8H), 4.33 (d, J = 16.8 Hz, 2H), 4.23 (d, J = 16.8 Hz, 2H), 2.79 (s, 3H).

^{13}C NMR (101 MHz, Acetone-d_6): δ 169.3, 149.5, 148.6, 134.4, 130.2, 125.2, 123.9, 123.5, 62.6, 48.2. The signal of the α-B-carbon was not observed.

^{11}B NMR (128 MHz, Acetone-d_6): δ 11.6.

HRMS (ESI): calc'd for $C_{12}H_{11}BF_3NNaO_4^+$ $[M+H]^+$ 401.166712; found 401.166860.

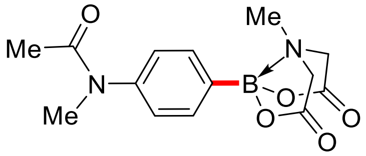

***N*-Methyl-*N*-(4-(6-methyl-4,8-dioxo-1,3,6,2-dioxazaborocan-2-yl)phenyl)acetamide, 42**

Following the general procedure, purification via column chromatography on silica gel [MTBE (100%) to MTBE/Acetone (4/1, *v*/*v*)] afforded **42** as a pale yellow solid (40.3 mg, 53% yield).

^{1}H NMR (400 MHz, Acetone-d_6): δ 7.62 (d, J = 8.0 Hz, 2H), 7.33–7.30 (m, 2H), 4.38 (d, J = 16.8 Hz, 2H), 4.18 (d, J = 17.2 Hz, 2H), 3.20 (s, 3H), 2.79 (s, 3H), 1.78 (s, 3H).

^{13}C NMR (101 MHz, Acetone-d_6): δ 169.7, 169.2, 146.6, 134.7, 127.3, 62.8, 48.3, 36.9, 22.4. The signal of the α-B-carbon was not observed.

^{11}B NMR (128 MHz, Acetone-d_6): δ 11.5.

HRMS (ESI): calc'd for $C_{14}H_{18}BN_2O_5^+$ $[M+H]^+$ 305.130327; found 305.130160.

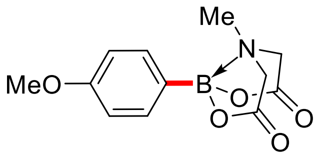

2-(4-Methoxyphenyl)-6-methyl-1,3,6,2-dioxazaborocane-4,8-dione, 43

Following the general procedure, purification via column chromatography on silica gel [MTBE (100%) to MTBE/Acetone (4/1, *v*/*v*)] afforded **43** as a pale yellow solid (54.3 mg, 83% yield).

^{1}H NMR (400 MHz, DMSO-*d$_6$*): δ 7.35 (d, *J* = 8.8 Hz, 2H), 6.92 (d, *J* = 8.4 Hz, 2H), 4.30 (d, *J* = 17.2 Hz, 2H), 4.07 (d, *J* = 17.2 Hz, 2H), 3.76 (s, 3H), 2.48 (s, 3H).

^{13}C NMR (101 MHz, DMSO-*d$_6$*): δ 169.4, 159.9, 133.7, 113.3, 61.6, 54.8, 47.5. The signal of the α-B-carbon was not observed.

^{11}B NMR (128 MHz, DMSO-*d$_6$*): δ 12.0.

HRMS (ESI): calc'd for $C_{12}H_{15}BNO_5^+$ $[M+H]^+$ 264.103779; found 264.103420.

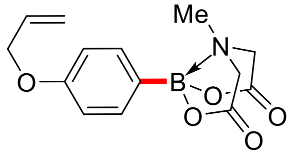

2-(4-(Allyloxy)phenyl)-6-methyl-1,3,6,2-dioxazaborocane-4,8-dione, 44

Following the general procedure, purification via column chromatography on silica gel [MTBE (100%) to MTBE/Acetone (4/1, *v*/*v*)] afforded **44** as a pale yellow solid (47.0 mg, 65% yield).

^{1}H NMR (400 MHz, Acetone-*d$_6$*): δ 7.47–7.43 (m, 2H), 6.97–6.93 (m, 2H), 6.13–6.03 (m, 1H), 5.45–5.39 (m, 1H), 5.26–5.22 (m, 1H), 4.58 (dt, *J* = 5.2 Hz, 1.6 Hz, 2H), 4.31 (d, *J* = 17.2 Hz, 2H), 4.10 (d, *J* = 16.8 Hz, 2H), 2.72 (s, 3H).

^{13}C NMR (101 MHz, Acetone-*d$_6$*): δ 169.3, 160.5, 134.7, 134.7, 117.3, 114.9, 69.0, 62.6, 48.1. The signal of the α-B-carbon was not observed.

^{11}B NMR (128 MHz, Acetone-*d$_6$*): δ 11.7.

HRMS (ESI): calc'd for $C_{14}H_{17}BNO_5^+$ $[M+H]^+$ 290.119429; found 290.119470.

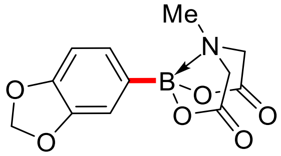

2-(Benzo[*d*][1,3]dioxol-5-yl)-6-methyl-1,3,6,2-dioxazaborocane-4,8-dione, 45

Following the general procedure, purification via column chromatography on silica gel [MTBE (100%) to MTBE/Acetone (4/1, *v*/*v*)] afforded **45** as a pale yellow solid (48.2 mg, 70% yield).

^{1}H NMR (400 MHz, Acetone-*d*$_6$): δ 7.02 (dd, *J* = 7.6 Hz, 1.6 Hz, 1H), 6.98 (d, *J* = 1.2 Hz, 1H), 6.86 (d, *J* = 7.6 Hz, 1H), 5.97 (s, 2H), 4.31 (d, *J* = 17.2 Hz, 2H), 4.12 (d, *J* = 16.8 Hz, 2H), 2.76 (s, 3H).

^{13}C NMR (101 MHz, Acetone-*d*$_6$): δ 169.2, 149.4, 148.4, 127.3, 112.8, 109.0, 101.5, 62.7, 48.1. The signal of the α-B-carbon was not observed.

^{11}B NMR (128 MHz, Acetone-*d*$_6$): δ 11.5.

HRMS (ESI): calc'd for $C_{12}H_{12}BNNaO_6^+$ $[M+Na]^+$ 300.064988; found 300.064970.

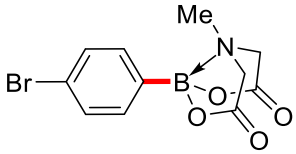

2-(4-Bromophenyl)-6-methyl-1,3,6,2-dioxazaborocane-4,8-dione, 46

Following the general procedure, purification via column chromatography on silica gel [MTBE (100%) to MTBE/Acetone (4/1, *v*/*v*)] afforded **46** as a pale yellow solid (55.6 mg, 72% yield).

^{1}H NMR (400 MHz, Acetone-*d*$_6$): δ 7.56–7.53 (m, 2H), 7.50–7.47 (m, 2H), 4.37 (d, *J* = 17.2 Hz, 2H), 4.17 (d, *J* = 17.2 Hz, 2H), 2.77 (s, 3H).

^{13}C NMR (101 MHz, Acetone-*d*$_6$): δ 169.2, 135.5, 131.7, 124.1, 62.8, 48.3. The signal of the α-B-carbon was not observed.

^{11}B NMR (128 MHz, Acetone-*d*$_6$): δ 11.4.

HRMS (ESI): calc'd for $C_{11}H_{12}BBrNO_4^+$ $[M+H]^+$ 312.003739; found 312.003280.

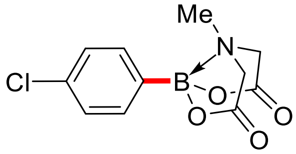

2-(4-Chlorophenyl)-6-methyl-1,3,6,2-dioxazaborocane-4,8-dione, 47

Following the general procedure, purification via column chromatography on silica gel [MTBE (100%) to MTBE/Acetone (4/1, *v*/*v*)] afforded **47** as a pale yellow solid (58.6 mg, 88% yield).

^{1}H NMR (400 MHz, Acetone-*d$_6$*): δ 7.56–7.53 (m, 2H), 7.41–7.38 (m, 2H), 4.37 (d, *J* = 17.2 Hz, 2H), 4.17 (d, *J* = 17.2 Hz, 2H), 2.77 (s, 3H).

^{13}C NMR (101 MHz, Acetone-*d$_6$*): δ 169.2, 135.6, 135.2, 128.7, 62.8, 48.3. The signal of the α-B-carbon was not observed.

^{11}B NMR (128 MHz, Acetone-*d$_6$*): δ 11.3.

HRMS (ESI): calc'd for $C_{11}H_{11}BClNNaO_4^+$ $[M+Na]^+$ 290.036186; found 290.035900.

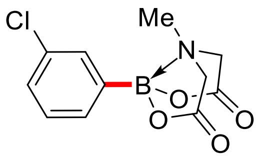

2-(3-Chlorophenyl)-6-methyl-1,3,6,2-dioxazaborocane-4,8-dione, 48

Following the general procedure, purification via column chromatography on silica gel [MTBE (100%) to MTBE/Acetone (4/1, *v*/*v*)] afforded **48** as a pale yellow solid (50.2 mg, 75% yield).

^{1}H NMR (400 MHz, Acetone-*d$_6$*): δ 7.54–7.53 (m, 1H), 7.49–7.47 (m, 1H), 7.42–7.36 (m, 2H), 4.39 (d, *J* = 17.2 Hz, 2H), 4.21 (d, *J* = 17.2 Hz, 2H), 2.81 (s, 3H).

^{13}C NMR (101 MHz, Acetone-*d$_6$*): δ 169.1, 134.6, 133.2, 131.8, 130.5, 129.8, 62.9, 48.4. The signal of the α-B-carbon was not observed.

^{11}B NMR (128 MHz, Acetone-*d$_6$*): δ 11.1.

HRMS (ESI): calc'd for $C_{11}H_{12}BClNO_4^+$ $[M+H]^+$ 268.054242; found 268.054010.

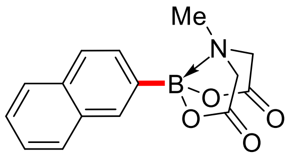

6-Methyl-2-(naphthalen-2-yl)-1,3,6,2-dioxazaborocane-4,8-dione, 49

Following the general procedure, purification via column chromatography on silica gel [MTBE (100%) to MTBE/Acetone (4/1, *v*/*v*)] afforded **49** as a pale yellow solid (57.3 mg, 81% yield).

^{1}H NMR (400 MHz, Acetone-*d$_6$*): δ 8.10 (s, 1H), 7.93–7.87 (m, 3H), 7.65 (dd, *J* = 8.0 Hz, 1.6 Hz, 1H), 7.53–7.47 (m, 2H), 4.40 (d, *J* = 17.2 Hz, 2H), 4.21 (d, *J* = 17.2 Hz, 2H), 2.76 (s, 3H).

^{13}C NMR (101 MHz, Acetone-*d$_6$*): δ 169.4, 134.9, 134.0, 133.8, 130.2, 129.1, 128.4, 127.9, 127.1, 126.6, 62.8, 48.3. The signal of the α-B-carbon was not observed.

¹¹B NMR (128 MHz, Acetone-*d*₆): δ 11.7.

HRMS (ESI): calc'd for $C_{15}H_{14}BNNaO_4^+$ [M+Na]$^+$ 306.090808; found 306.090790.

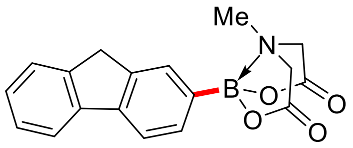

2-(9*H*-Fluoren-2-yl)-6-methyl-1,3,6,2-dioxazaborocane-4,8-dione, 50

Following the general procedure, purification via column chromatography on silica gel [MTBE (100%) to MTBE/Acetone (4/1, *v*/*v*)] afforded **50** as a pale yellow solid (65.7 mg, 82% yield).

¹H NMR (400 MHz, Acetone-*d*₆): δ 7.89–7.85 (m, 2H), 7.76 (s, 1H), 7.57 (t, *J* = 7.2 Hz, 2H), 7.38 (t, *J* = 7.2 Hz, 1H), 7.31 (td, *J* = 7.2 Hz, 1.6 Hz, 1H), 4.37 (d, *J* = 17.2 Hz, 2H), 4.17 (d, *J* = 16.8 Hz, 2H), 3.91 (s, 2H), 2.75 (s, 3H).

¹³C NMR (101 MHz, Acetone-*d*₆): δ 169.4, 144.4, 143.6, 143.3, 142.5, 131.9, 130.0, 127.7, 127.6, 126.0, 120.8, 120.1, 62.7, 48.3, 37.3. The signal of the α-B-carbon was not observed.

¹¹B NMR (128 MHz, Acetone-*d*₆): δ 11.9.

HRMS (ESI): calc'd for $C_{18}H_{16}BNNaO_4^+$ [M+Na]$^+$ 344.106458; found 344.106470.

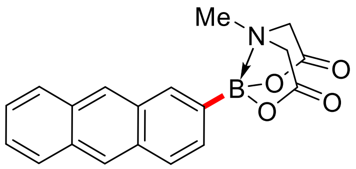

2-(Anthracen-2-yl)-6-methyl-1,3,6,2-dioxazaborocane-4,8-dione, 51

Following the general procedure, purification via column chromatography on silica gel [MTBE (100%) to MTBE/Acetone (4/1, *v*/*v*)] afforded **51** as a pale yellow solid (56.5 mg, 68% yield).

¹H NMR (400 MHz, Acetone-*d*₆): δ 8.56 (s, 1H), 8.52 (s, 1H), 8.28 (s, 1H), 8.10–8.04 (m, 3H), 7.64 (dd, *J* = 8.8 Hz, 1.6 Hz, 1H), 7.52–7.48 (m, 2H), 4.43 (d, *J* = 17.2 Hz, 2H), 4.24 (d, *J* = 17.2 Hz, 2H), 2.82 (s, 3H).

¹³C NMR (101 MHz, Acetone-*d*₆): δ 169.4, 134.4, 133.0, 133.0, 132.7, 132.4, 129.5, 129.1, 128.9, 128.1, 127.6, 126.6, 126.4, 126.2, 62.9, 48.3. The signal of the α-B-carbon was not observed.

¹¹B NMR (128 MHz, Acetone-*d*₆): δ 11.8.

HRMS (ESI): calc'd for $C_{19}H_{16}BNNaO_4^+$ [M+Na]$^+$ 356.106458; found 356.106680.

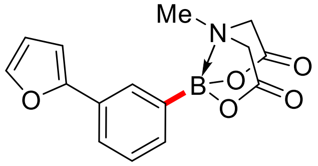

2-(3-(Furan-2-yl)phenyl)-6-methyl-1,3,6,2-dioxazaborocane-4,8-dione, 52

Following the general procedure, bis(catecholato)diboron (4.0 equiv) and $^{i}Pr_2NCOMe$ (2.0 mL) were used and the borylation reaction was performed at 130 °C for 24 h. Purification via column chromatography on silica gel [MTBE (100%) to MTBE/Acetone (4/1, *v*/*v*)] afforded **52** as a pale yellow solid (38.6 mg, 52% yield).

^{1}H NMR (400 MHz, Acetone-*d*$_6$): δ 8.02–8.01 (m, 1H), 7.78 (s, 1H), 7.63–7.60 (m, 2H), 7.45–7.37 (m, 2H), 6.89 (dd, *J* = 2.0 Hz, 0.8 Hz, 1H), 4.36 (d, *J* = 17.2 Hz, 2H), 4.18 (d, *J* = 17.2 Hz, 2H), 2.79 (s, 3H).

^{13}C NMR (101 MHz, Acetone-*d*$_6$): δ 169.3, 144.8, 139.9, 132.7, 132.0, 130.7, 129.1, 127.6, 127.4, 109.7, 62.8, 48.3. The signal of the α-B-carbon was not observed.

^{11}B NMR (128 MHz, Acetone-*d*$_6$): δ 11.6.

HRMS (ESI): calc'd for $C_{15}H_{15}BNO_5^+$ $[M+H]^+$ 300.103779; found 300.103540.

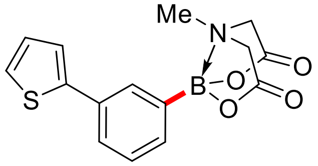

6-Methyl-2-(3-(thiophen-2-yl)phenyl)-1,3,6,2-dioxazaborocane-4,8-dione, 53

Following the general procedure, bis(catecholato)diboron (4.0 equiv) and $^{i}Pr_2NCOMe$ (2.0 mL) were used and the borylation reaction was performed at 130 °C for 24 h. Purification via column chromatography on silica gel [MTBE (100%) to MTBE/Acetone (4/1, *v*/*v*)] afforded **53** as a pale yellow solid (49.3mg, 63% yield).

^{1}H NMR (400 MHz, Acetone-*d*$_6$): δ 7.85 (s, 1H), 7.69–7.67 (m, 1H), 7.49–7.40 (m, 4H), 7.13–7.11 (m, 1H), 4.38 (d, *J* = 17.2 Hz, 2H), 4.20 (d, *J* = 16.8 Hz, 2H), 2.81 (s, 3H).

^{13}C NMR (101 MHz, Acetone-*d*$_6$): δ 169.3, 145.3, 134.6, 132.7, 130.6, 129.3, 129.0, 127.3, 125.8, 124.2, 62.9, 48.4. The signal of the α-B-carbon was not observed.

^{11}B NMR (128 MHz, Acetone-*d*$_6$): δ 11.5.

HRMS (ESI): calc'd for $C_{15}H_{15}BNO_4S^+$ $[M+H]^+$ 316.080936; found 316.080670.

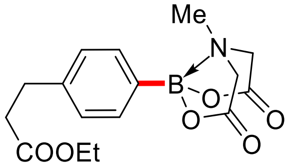

Ethyl 3-(4-(6-methyl-4,8-dioxo-1,3,6,2-dioxazaborocan-2-yl)phenyl)propanoate, 54

Following the general procedure, purification via column chromatography on silica gel [MTBE (100%) to MTBE/Acetone (3/1, *v*/*v*)] afforded **54** as a yellow solid (61.6 mg, 74% yield).

^{1}H NMR (400 MHz, DMSO-*d₆*): δ 7.34 (d, *J* = 7.6 Hz, 2H), 7.21 (d, *J* = 7.2 Hz, 2H), 4.31 (d, *J* = 17.2 Hz, 2H), 4.11–4.01 (m, 4H), 2.85 (t, *J* = 7.6 Hz, 2H), 2.61 (t, *J* = 7.6 Hz, 2H), 2.48 (s, 3H), 1.15 (t, *J* = 7.2 Hz, 3H).

^{13}C NMR (101 MHz, DMSO-d_6): δ 172.2, 169.4, 141.1, 132.4, 127.6, 61.7, 59.8, 47.5, 34.9, 30.2, 14.1. The signal of the α-B-carbon was not observed.

^{11}B NMR (128 MHz, DMSO-d_6): δ 11.7.

HRMS (ESI): calc'd for $C_{16}H_{20}BNNaO_6^+$ [M+Na]$^+$ 356.127588; found 356.127760.

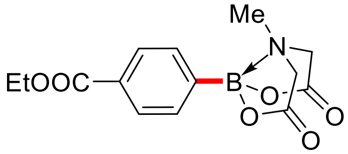

Ethyl 4-(6-methyl-4,8-dioxo-1,3,6,2-dioxazaborocan-2-yl)benzoate, 55

Following the general procedure, bis(catecholato)diboron (4.0 equiv) was used and the borylation reaction was performed at 130 °C for 24 h. Purification via column chromatography on silica gel [MTBE (100%) to MTBE/Acetone (3/1, *v*/*v*)] afforded **55** as a pale yellow solid (46.3 mg, 61% yield).

^{1}H NMR (400 MHz, Acetone-d_6): δ 8.01–7.99 (m, 2H), 7.69–7.67 (m, 2H), 4.40 (d, *J* = 16.0 Hz, 2H), 4.35 (q, *J* = 7.2 Hz, 2H), 4.19 (d, *J* = 17.2 Hz, 2H), 2.77 (s, 3H), 1.37 (t, *J* = 7.2 Hz, 3H).

^{13}C NMR (101 MHz, Acetone-d_6): δ 169.2, 166.9, 133.6, 132.0, 129.3, 62.9, 61.4, 48.4, 14.6. The signal of the α-B-carbon was not observed.

^{11}B NMR (128 MHz, Acetone-d_6): δ 11.2.

HRMS (ESI): calc'd for $C_{14}H_{17}BNO_6^+$ [M+H]$^+$ 306.114344; found 306.114170.

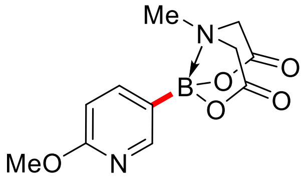

2-(6-Methoxypyridin-3-yl)-6-methyl-1,3,6,2-dioxazaborocane-4,8-dione, 56

Following the general procedure, bis(catecholato)diboron (4.0 equiv) was used and the borylation reaction was performed at 130 °C for 24 h. Purification via column chromatography on silica gel [MTBE (100%) to MTBE/Acetone (3/1, *v*/*v*)] afforded **56** as a white solid (42.0 mg, 64% yield).

^{1}H NMR (400 MHz, Acetone-*d$_6$*): δ 8.27 (dd, *J* = 2.0 Hz, 0.8 Hz, 1H), 7.77 (dd, *J* = 8.4 Hz, 2.4 Hz, 1H), 6.74 (dd, *J* = 8.4 Hz, 0.8 Hz, 1H), 4.36 (d, *J* = 17.2 Hz, 2H), 4.18 (d, *J* = 16.8 Hz, 2H), 3.89 (s, 3H), 2.82 (s, 3H).

^{13}C NMR (101 MHz, Acetone-*d$_6$*): δ 169.1, 165.8, 152.3, 143.8, 110.9, 62.7, 53.3, 48.2. The signal of the α-B-carbon was not observed.

^{11}B NMR (128 MHz, Acetone-*d$_6$*): δ 11.5.

HRMS (ESI): calc'd for $C_{11}H_{14}BN_2O_5^+$ [M+H]$^+$ 265.099027; found 265.098980.

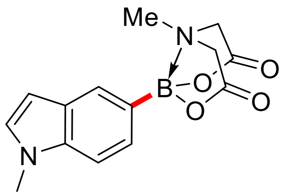

6-Methyl-2-(1-methyl-1*H*-indol-5-yl)-1,3,6,2-dioxazaborocane-4,8-dione, 57

Following the general procedure, bis(catecholato)diboron (4.0 equiv) was used and the borylation reaction was performed at 130 °C for 24 h. Purification via column chromatography on silica gel [MTBE (100%) to MTBE/Acetone (3/1, *v*/*v*)] afforded **57** as a pale yellow solid (41.3 mg, 58% yield).

^{1}H NMR (400 MHz, Acetone-*d$_6$*): δ 7.75 (s, 1H), 7.39 (dt, *J* = 8.4 Hz, 0.8 Hz, 1H), 7.33 (dd, *J* = 8.0 Hz, 1.2 Hz, 1H), 7.19 (d, *J* = 3.2 Hz, 1H), 6.44 (dd, *J* = 3.2 Hz, 0.8 Hz, 1H), 4.31 (d, *J* = 17.2 Hz, 2H), 4.10 (d, *J* = 16.8 Hz, 2H), 3.82 (s, 3H), 2.66 (s, 3H).

^{13}C NMR (101 MHz, Acetone-*d$_6$*): δ 169.5, 138.5, 129.8, 129.5, 126.2, 126.1, 109.7, 101.6, 62.6, 48.2, 32.8. The signal of the α-B-carbon was not observed.

^{11}B NMR (128 MHz, Acetone-*d$_6$*): δ 12.5.

HRMS (ESI): calc'd for $C_{14}H_{16}BN_2O_4^+$ [M+H]$^+$ 287.119762; found 287.119530.

6. Gram-scale Synthesis and One-pot Borylation

6.1 Gram-scale Synthesis

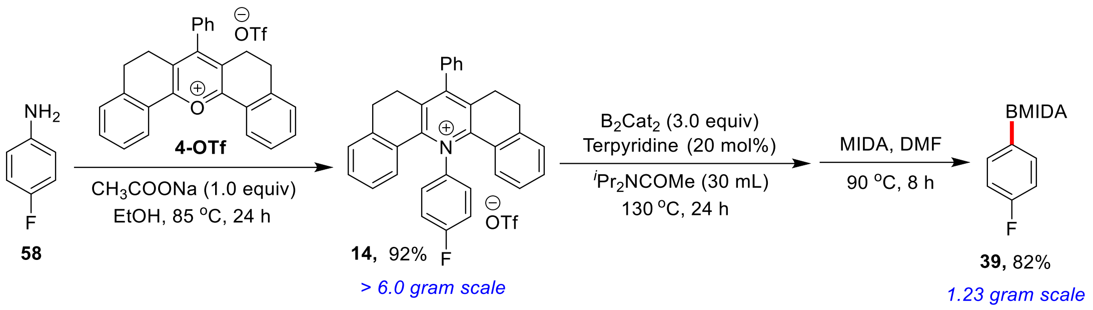

Synthesis of Pyridinium Salt 4-OTf

A 100 mL Schlenk tube equipped with a stirring bar was charged with pyrylium salt **4-OTf** (6.12 g, 12 mmol), 4-fluoroaniline **58** (1.40 g, 12.6 mmol, 1.05 equiv) and sodium acetate (0.984 g, 12 mmol). Then EtOH (50 mL) was added to the resulting mixture and the reaction was refluxed at 85 °C (temperature of oil bath) for 24 h. After that time, the Schlenk tube was cooled to room temperature. The product crushed out during the reaction or after cooling down. Upon filtration, washing with Et_2O three times (~100 mL) and drying under high vacuum successively, **14** was obtained as a pale yellow solid (6.66 g, 92%).

Borylation of Pyridinium Salt 14

A dry 100 mL Schlenk tube equipped with a stir bar was charged with terpyridine (280 mg, 1.2 mmol), and then transferred to an argon-filled glove box. Pyridinium salt **14** (3.62 g, 6.0 mmol), bis(catecholato)diboron (4.3 g, 18.0 mmol) and $^{i}Pr_2NCOMe$ (30.0 mL) were added successively. The Schlenk tube was sealed and taken out of the glovebox. The resulting mixture was stirred at 130 °C (temperature of oil bath) for 24 h. After this time, the Schlenk tube was cooled to room temperature. Then methyliminodiacetic acid (5.3 g, 36.0 mmol) and dry DMF (25.0 mL) were added under argon and the reaction mixture was heated to 90 °C for 8 h, after which solvent $^{i}Pr_2NCOMe$ and DMF were removed under high vacuum. The resulting crude mixture was purified by column chromatography on silica gel using an elution gradient from MTBE (100%) to MTBE/Acetone (4/1, *v*/*v*) to afford the desired borylation product **39** as a pale yellow solid (1.23 g, 82%).

6.2 One-pot Procedure for Borylation of 4-Fluoroaniline

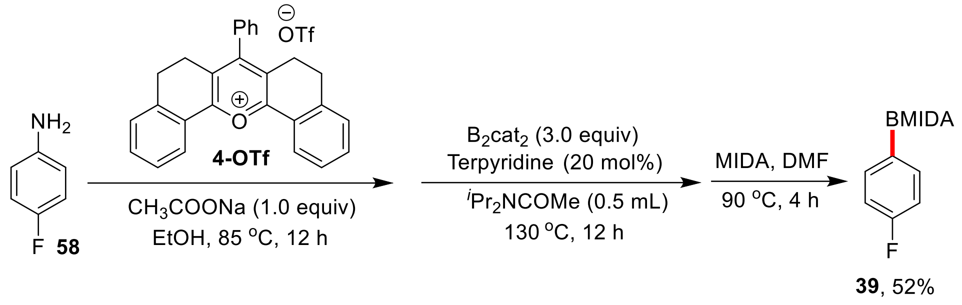

A dry 10 mL-reaction vial equipped with a stirring bar was charged with pyrylium salt **4-OTf** (127.5 mg, 0.25 mmol), 4-fluoroaniline **58** (29.2 mg, 0.263 mmol, 1.05 equiv) and sodium acetate (20.5 mg, 0.25 mmol). Then EtOH (1.0 mL) was added to the resulting mixture and the reaction was refluxed at 85 ^{o}C (temperature of oil bath) for 12 h. After that time, the vial was cooled to room temperature. Evaporation of volatiles afforded a crude mixture for next step without further purification. After terpyridine (11.7 mg, 0.05 mmol) was added to resulting mixture, then the vial was transferred to an argon-filled glove box. Bis(catecholato)diboron (178.5 mg, 0.75 mmol) and $^{i}Pr_2$NCOMe (0.2 M, 1.25 mL) were added successively. The vial was sealed and taken out of the glovebox. The resulting mixture was stirred at 130 °C (temperature of oil bath) for 12 h. After this time, the vial was cooled to room temperature. Then methyliminodiacetic acid (257.3 mg, 1.75 mmol) and dry DMF (1.0 mL) were added under argon and the reaction mixture was heated to 90 °C for 4 h, after which solvent $^{i}Pr_2$NCOMe and DMF were removed under high vacuum. The resulting crude mixture was purified by column chromatography on silica gel using an elution gradient from MTBE (100%) to MTBE/Acetone (4/1, *v*/*v*) to afford the desired borylation product **39** as a pale yellow solid (32.5 mg, 52%).

7. Mechanistic Studies

7.1 Hydrogen Atom Abstraction of Radical Intermediates

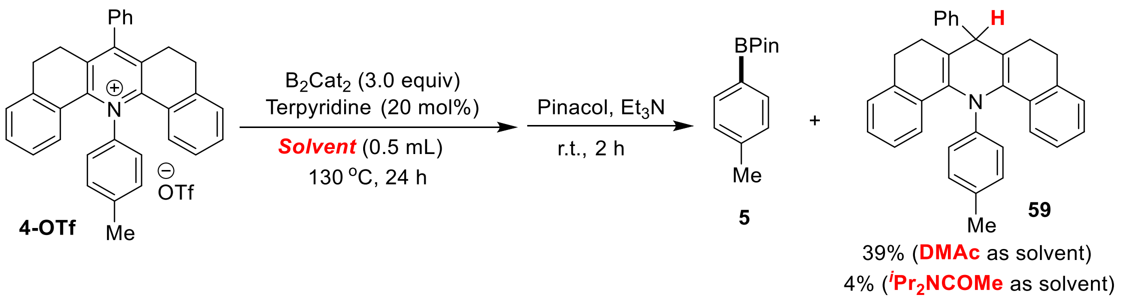

A dry 10 mL-reaction vial equipped with a stir bar was charged with terpyridine (4.7 mg, 0.02 mmol), and then transferred to an argon-filled glove box. Pyridinium salt **4-OTf** (60.1 mg, 0.1 mmol) and bis(catecholato)diboron (71.4 mg, 0.3 mmol) and DMAc (0.5 mL) or ^{i}Pr$_2$NCOMe (0.5 mL) were added successively. The vial was sealed and taken out of the glovebox. The resulting mixture was stirred at 130 °C (temperature of oil bath) for 24 h. After this time, the vial was cooled to room temperature. Then pinacol (70.8 g, 0.6 mmol) and dry Et$_3$N (0.5 mL) were added under argon and the reaction mixture was stirred at room temperature for 2 h. The resulting crude mixture was purified by column chromatography on silica gel using an elution gradient from Hexane (100%) to Hexane/Dichloromethane (3/1, *v*/*v*) to afford the side product **59** as a white solid (17.5 mg, 39%, DMAc as solvent). In the case of using ^{i}Pr$_2$NCOMe as solvent, side product **59** was isolated in 2 mg (4%) yield. Characterization of **59** as below:

^{1}H NMR (400 MHz, CDCl$_3$): δ 7.69 (d, *J* = 7.2 Hz, 2H), 7.25–7.11 (m, 11H), 6.84 (d, *J* = 8.0 Hz, 2H), 6.70–6.66 (m, 2H), 3.69 (s, 1H), 2.86–2.72 (m, 4H), 2.40–2.21 (m, 4H), 2.19 (s, 3H).

^{13}C NMR (101 MHz, CDCl$_3$): δ 145.7, 142.2, 137.9, 134.9, 134.1, 132.9, 129.2, 128.9, 128.7, 128.6, 127.5, 126.9, 126.9, 126.8, 123.8, 117.7, 52.1, 28.7, 26.4, 20.5.

HRMS (ESI): calc'd for $C_{34}H_{30}N^{+}$ $[M+H]^{+}$ 452.237274; found 452.237060.

The structure of 59 was further confirmed by its preparation using an alternative route as shown below.

7.2 Exploring the origin of byproduct 59

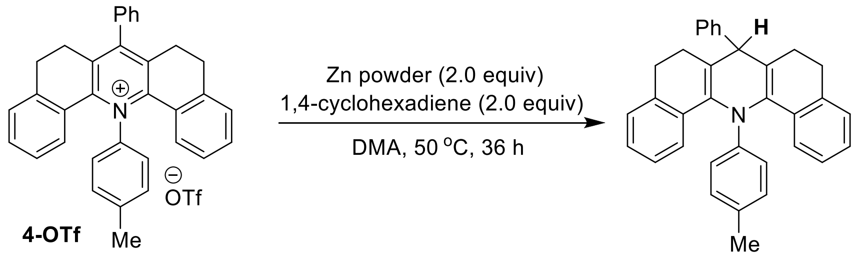

59, 77%

59 was prepared by the following method: to a 10 mL Schlenk flask pyridinium salt **4-OTf** (1.2 g, 2.0 mmol), zinc powder (SET reagent, 262 mg, 2.0 equiv.) and anhydrous DMAc (5.0 mL) were added in glove box, and the reaction turned deep yellow immediately. Cyclohexa-1,4-diene (HAT reagent, 320 mg, 2.0 equiv.) was added via Hamilton syringe out of glove box. The reaction was stirred at 50 ºC in dark for 36 h until it turned yellow muddy. The pure compound was isolated by flash chromatography [*n*-hexane/DCM (3/1, *v*/*v*)] as white solid (706 mg, 78%). Its NMR data are identical with that described above.

7.3 Trapping of Aryl Radical

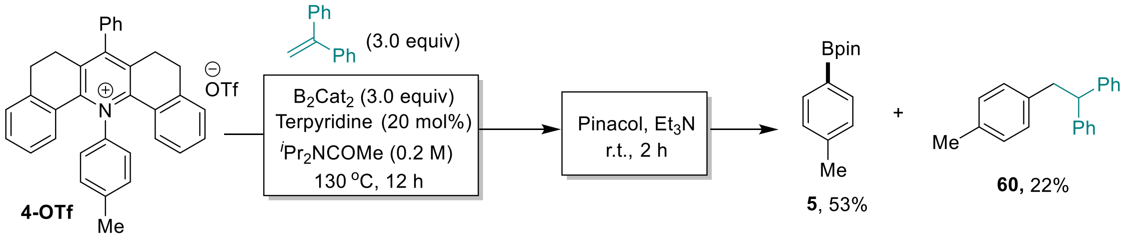

A dry 10 mL-reaction vial equipped with a stir bar was charged with terpyridine (11.7 mg, 0.05 mmol), and then transferred to an argon-filled glove box. Pyridinium salt **4-OTf** (150.0 mg, 0.25 mmol) and bis(catecholato)diboron (178.5 mg, 0.75 mmol) and ^{i}Pr$_2$NCOMe (0.2M, 1.25 mL) were added successively. The vial was sealed and taken out of the glovebox, and 1,1'-diphenylethylene (132 µL, 0.75 mmol) was added to the vial via a microsyringe under an argon atmosphere. The resulting mixture was stirred at 130 °C (temperature of oil bath) for 12 h. After this time, the vial was cooled to room temperature. Then pinacol (177.0 g, 1.50 mmol) and dry Et_3N (1.25 mL) were added under argon and the reaction mixture was stirred at room temperature for 2 h. *n*-Dodecane (57 µL, 0.25 mmol) was added as internal standard. And then the reaction mixture was submitted to GC-FID for the analysis of borylation product **5 (**53%). The crude mixture was purified by column chromatography on silica gel using *n*-hexane (100%) to afford a mixture–containing side product **60** (22%), 1,1'-diphenylethylene and *n*-dodecane (dibromomethane was used as internal standard). **60** was further confirmed by GC-MS, where certain amount of (2-(*p*-tolyl)ethene-1,1-diyl)dibenzene was also detected.

7.4 Cyclic Voltammetry Measurement

Cyclic voltammetry (CV) was performed with a Metrohm Autolab PGSTAT101 potentiostat and processed using Metrohm Autolab Nova software 2.1. A cylindrical three-electrode cell was equipped with a glassy carbon working electrode (⌀ 3 mm), a platinum wire as the counter electrode and $Ag/AgNO_3$ (0.01 M) electrode as the reference electrode. The scan rate for a typical experiment was 100 $mV\cdot s^{-1}$. The scan direction was negative. The solution of pyrdinium salt (1.0×10^{-3} M) and *n*-Bu_4NPF_6 (0.2 M) in dry DMF was deaerated by Ar gas bubbling before the measurement, and the cyclic voltammetry was carried out under an Ar gas atmosphere at room temperature. CV of pyridinium salts were first measured, and then CV of ferrocene was measured for each pyridinium salts.

For 7-phenyl-14-(*p*-tolyl)-5,6,8,9-tetrahydrodibenzo[*c*,*h*]acridin-14-ium trifluoromethanesulfonate (**4-OTf**):

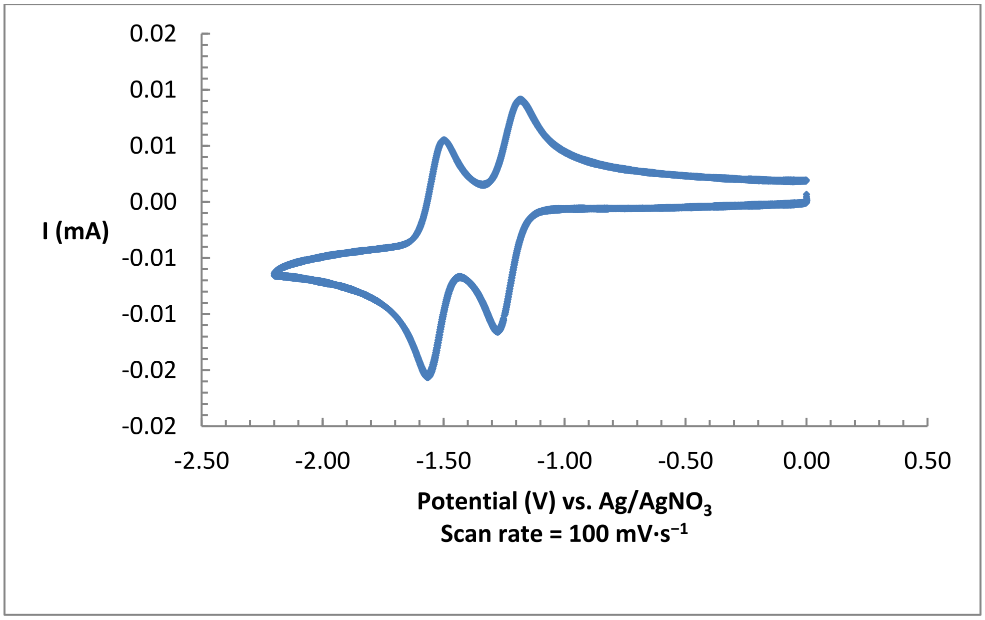

The reversible peaks at -1.27 V and -1.19 V correspond to the first reduction of pyridinium salt **4-OTf**. The reversible peaks at -1.56 V and -1.51 V correspond to the second reduction of pyridinium salt **4-OTf**. The measurements with scan rates 200, 400 and 800 $mV\cdot s^{-1}$ gave the same values. The $E_{1/2}$ value of the ferrocene–ferrocenium (Fc/Fc^+) in DMF was +0.074 V vs. $Ag/AgNO_3$ in this measurement.

E_{red1}(**4-OTf**) = - 1.23 V, E_{red2}(**4-OTf**) = - 1.54 V;

E_{red1}(**4-OTf**) = - 1.30 V vs. Fc/Fc^+, E_{red2}(**4-OTf**) = - 1.61 V vs. Fc/Fc^+;

For 2,4,6-triphenyl-1-(*p*-tolyl)pyridin-1-ium tetrafluoroborate (**3**):

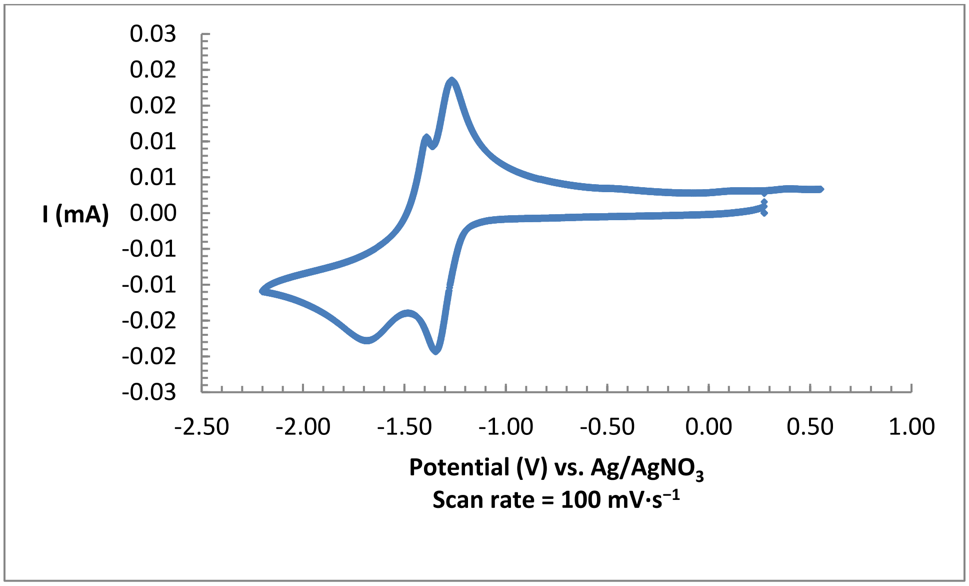

The reversible peaks at -1.33 V and -1.28 V correspond to the first reduction of pyridinium salt **3**. The semi-reversible peaks at -1.65 V and -1.40 V correspond to the second reduction of pyridinium salt **3**. The measurements with scan rates 200, 400 and 800 $mV\cdot s^{-1}$ gave the same values. The $E_{1/2}$ value of the ferrocene–ferrocenium (Fc/Fc^{+}) in DMF was +0.090 V vs. $Ag/AgNO_3$ in this measurement.

E_{red1}(**3**) = - 1.30 V, E_{red2}(**3**) = - 1.52 V;

E_{red1}(**3**) = - 1.39 V vs. Fc/Fc^{+}, E_{red2}(**3**) = - 1.61 V vs. Fc/Fc^{+}.

7.5 EPR Measurements

EPR spectra were obtained using a commercial X-band spectrometer (MS5000, Magnettech GmbH) at 293 K. The EPR spectra were recorded using 10 mW microwave power, 12 mT field sweep centered at 338 mT, a modulation amplitude of 0.1 mT, a sweep time 30 s and a microwave frequency of 9.47 GHz.

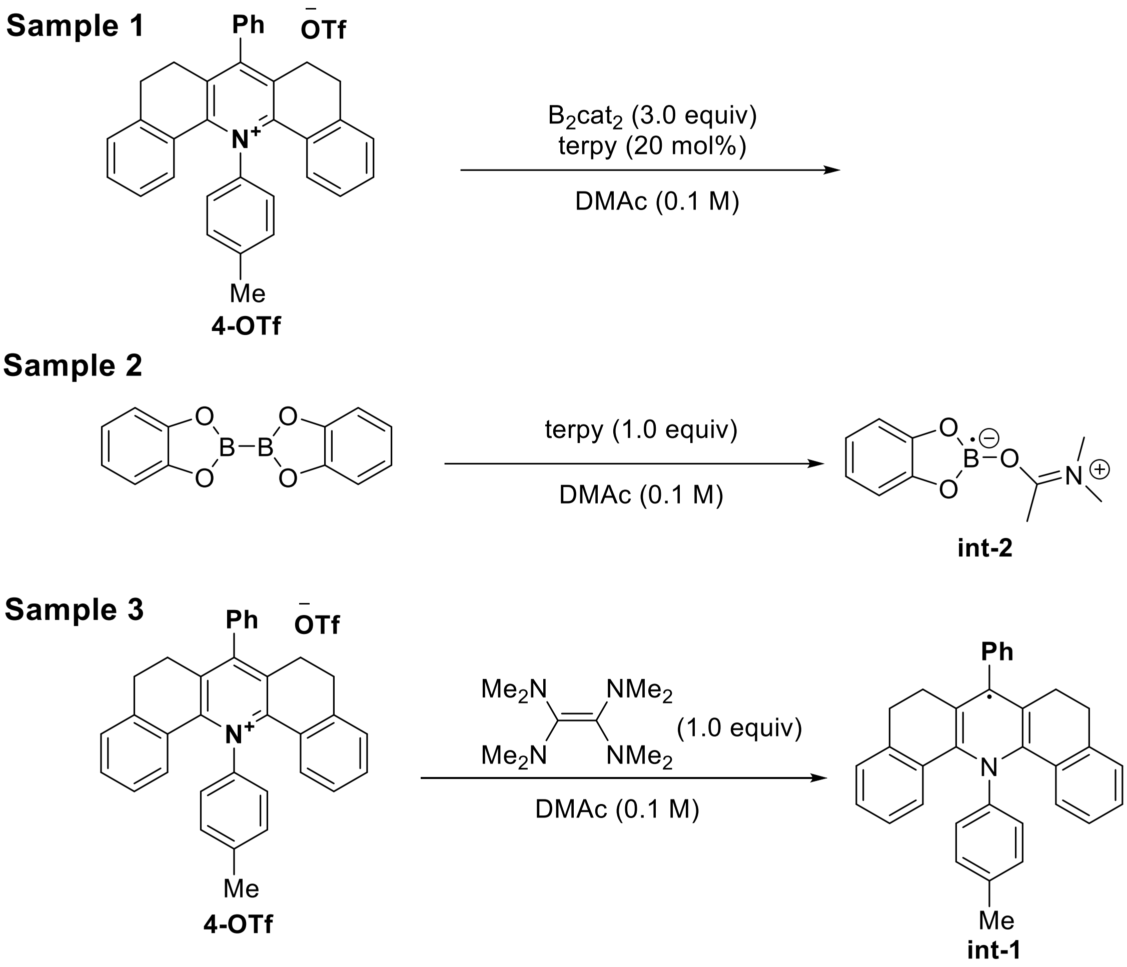

In a glovebox, each of the samples were prepared in a 10 ml vial and stirred for 20 min. The mixtures were transferred into 1.6 mm quartz capillaries, which were sealed from the top by wax. These capillaries were inserted into a 4 mm tube and were sealed using rubber septa, and brought out of the glovebox. The samples were kept in liquid nitrogen until EPR measurements.

For EPR measurements, DMAc was chosen as the solvent because of the sluggish solubility of **4-OTf** in $^{i}Pr_2NC(O)Me$ at ambient temperature. In sample 1, **4-OTf** (30 mg, 0.05 mmol), B_2cat_2 (36 mg, 0.15 mmol, 3.0 equiv.), terpyridine (2.3 mg, 0.01 mmol, 20 mol%) and dry DMAc (0.5 ml, 0.1 M) were used. In sample 2,

B_2cat_2 (12 mg, 0.05 mmol), terpyridine (12 mg, 0.05 mmol, 1.0 equiv.) and dry DMAc (0.5 ml, 0.1 M) were used. In sample 3, **4-OTf** (30 mg, 0.05 mmol), TDAE (12 µl, 0.05 mmol, 1.0 equiv.) and dry DMAc (0.5 ml, 0.1 M) were used.

7.6 X-ray Crystallography

The single crystal of compound **3·acetone** was obtained by heating the mixture of **3** and acetone to reflux and cooling down slowly to room temperature. The single crystal of compound **4-BF$_4$·H$_2$O** was obtained by vapor diffusion between *n*-pentane and the chloroform solution of **4-BF$_4$**.

CCDC number 1957258 and **1957259** (for **3·acetone** and **4-BF$_4$·H$_2$O**) contain the supplementary crystallographic data for this paper. These data can be obtained free of charge from The Cambridge Crystallographic Data Centre via www.ccdc.cam.ac.uk/data_request/cif.

7.6.1 X-ray structure of compound 3·acetone

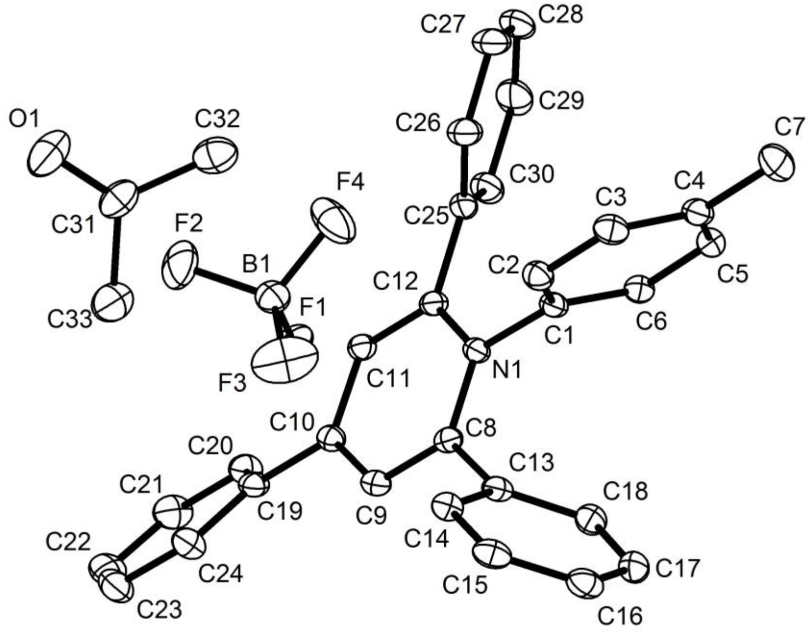

Crystal data and structure refinement of 3·acetone.

Identification code	3-acetone (12581)	
Empirical formula	$C_{33} H_{30} B F_4 N O$	
Color	colourless	
Formula weight	543.39 g·mol^{-1}	
Temperature	100(2) K	
Wavelength	0.71073 Å	
Crystal system	monoclinic	
Space group	*P* 2$_1$, (no. 4)	
Unit cell dimensions	a = 11.0265(7) Å	α= 90°.

b = 8.1991(5) Å	β= 93.158(5)°.	
c = 15.6689(15) Å	γ = 90°.	
Volume	1414.43(18) $Å^3$	
Z	2	
Density (calculated)	1.276 $Mg·m^{-3}$	
Absorption coefficient	0.094 mm^{-1}	
F(000)	568 e	
Crystal size	0.18 x 0.10 x 0.06 mm^3	
θ range for data collection	2.604 to 33.119°.	
Index ranges	-16 ≤ h ≤ 16, -12≤ k ≤ 12, -24≤ l ≤ 24	
Reflections collected	32316	
Independent reflections	10684 [R_{int} = 0.0455]	
Reflections with I>2σ(I)	7627	
Completeness to θ = 25.242°	99.8 %	
Absorption correction	Gaussian	
Max. and min. transmission	0.99497 and 0.98808	
Refinement method	Full-matrix least-squares on F^2	
Data / restraints / parameters	10684 / 1 / 364	
Goodness-of-fit on F^2	1.020	
Final R indices [I>2σ(I)]	R_1 = 0.0471	wR^2 = 0.0929
R indices (all data)	R_1 = 0.0842	wR^2 = 0.1038
Absolute structure parameter	0.5	
Extinction coefficient	0	
Largest diff. peak and hole	0.314 and -0.225 $e·Å^{-3}$	

7.6.2 X-ray structure of compound 4-BF_4·H_2O

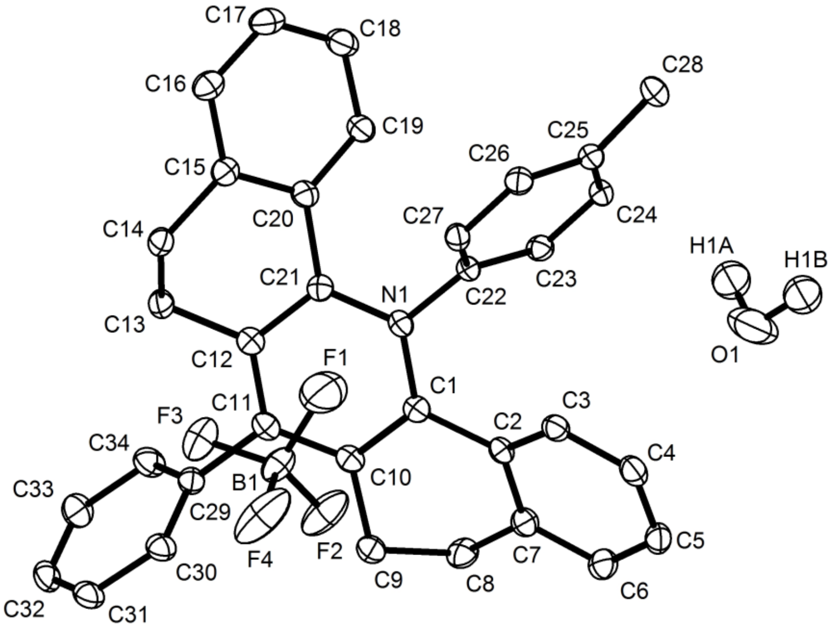

Crystal data and structure refinement of 4-$BF_4 \cdot H_2O$.

Identification code	4-water (12630)	
Empirical formula	$C_{34} H_{28.50} B F_4 N O_{0.25}$	
Color	yellow	
Formula weight	541.89 g·mol^{-1}	
Temperature	100(2) K	
Wavelength	1.54178 Å	
Crystal system	monoclinic	
Space group	*P* 2_1, (no. 4)	
Unit cell dimensions	a = 8.9212(13) Å	α= 90°.
	b = 13.881(2) Å	β= 100.437(7)°.
	c = 10.8608(16) Å	γ = 90°.
Volume	1322.7(3) Å^3	
Z	2	
Density (calculated)	1.361 Mg·m^{-3}	
Absorption coefficient	0.810 mm^{-1}	
F(000)	565 e	
Crystal size	0.497 x 0.247 x 0.070 mm^3	
θ range for data collection	4.139 to 72.373°.	
Index ranges	-10 ≤ h ≤ 10, -17≤ k ≤ 17, -13≤ l ≤ 13	
Reflections collected	45707	
Independent reflections	4871 [R_{int} = 0.0448]	

Reflections with I>2σ(I)	4635	
Completeness to θ = 67.679°	99.4 %	
Absorption correction	Gaussian	
Max. and min. transmission	0.94684 and 0.78717	
Refinement method	Full-matrix least-squares on F^2	
Data / restraints / parameters	4871 / 1 / 371	
Goodness-of-fit on F^2	1.083	
Final R indices [I>2σ(I)]	R_1 = 0.0329	wR^2 = 0.0799
R indices (all data)	R_1 = 0.0366	wR^2 = 0.0821
Absolute structure parameter	0.01(5)	
Extinction coefficient	0	
Largest diff. peak and hole	0.287 and -0.260 e·Å^{-3}	

8. References

[1] K. T. Potts, P. Ralli, G. Theodoridis, P. Winslow, *Org. Synth.* **1986**, *64*, 189.

[2] F. J. Lawlor, N. C. Norman, N. L. Pickett, E. G. Robins, P. Nguyen, G. Lesley, T. B. Marder, J. A. Ashmore, J. C. Green, *Inorg. Chem.* **1998**, *37*, 5282-5288.

[3] a) *Pyrylium salts: Syntheses, Reactions and Physical Properties*. ed.; A. T. Balaban, A. Dinculescu, G. N. Dorofeenko, G. W. Fischer, A. V. Koblik, V. V. Mezheritskii, W. Schroth, Academic Press: **1982**; Vol. 2; b) T. S. Balaban, A. T. Balaban, Pyrylium Salts. In *Science of Synthesis*, Thieme-Verlag: Stuttgart, **2004**; Vol. 15, pp 11-200.

[4] A. R. Katritzky, J. M. Lloyd, R. C. Patel, *J. Chem. Soc., Perkin Trans. 1* **1982**, 117-123.

[5] D. Moser, Y. Duan, F. Wang, Y. Ma, M. J. O'Neill, J. Cornella, *Angew. Chem. Int. Ed.* **2018**, *57*, 11035-11039.

[6] a) L. Dong, G. Li, A.-D. Yu, Z. Bo, C.-L. Liu, W.-C. Chen, *Chem. Asia. J.* **2014**, *9*, 3403-3407; b) W.-Y. Lee, T. Kurosawa, S.-T. Lin, T. Higashihara, M. Ueda, W.-C. Chen, *Chem. Mater.* **2011**, *23*, 4487-4497.

[7] a) S. Krompiec, N. Kuźnik, R. Penczek, J. Rzepa, J. Mrowiec-Białoń, *J. Mol. Catal. A: Chem.* **2004**, *219*, 29-40; b) B. Schmidt, F. Wolf, *J. Org. Chem.* **2017**, *82*, 4386-4395.

[8] O. Moradei, I. Paquin, S. Leit, S. Frechette, A. Vaisburg, J. M. Besterman, P. Tessier, T. C. Mallais, Preparation of amide derivatives as inhibitors of histone deacetylase. WO2005030704A1, **2005**.

[9] a) F. Coullet, S. Morel, G. Boyer, J. P. Galy, *Synth. Commun.* **1998**, *28*, 147-157; b) M. G. Ferlin, G. Chiarelotto, S. Dall'Acqua, E. Maciocco, M. P. Mascia, M. G. Pisu, G. Biggio, *Biorg. Med. Chem.* **2005**, *13*, 3531-3541.

[10] R. Das, D. Chakraborty, *Synthesis* **2011**, *2011*, 1621-1625.

[11] a) L. Candish, M. Teders, F. Glorius, *J. Am. Chem. Soc.* **2017**, *139*, 7440-7443; b) Y. Cheng, C. Mück-Lichtenfeld, A. Studer, **2018**, *57*, 16832-16836; c) D. Mazzarella, G. Magagnano, B. Schweitzer-Chaput, P. Melchiorre, *ACS Catal.* **2019**, 9, 5876-5880; d) L. Zhang, L. Jiao, *J. Am. Chem. Soc.* **2019**, *141*, 9124-9128.

9. NMR spectra

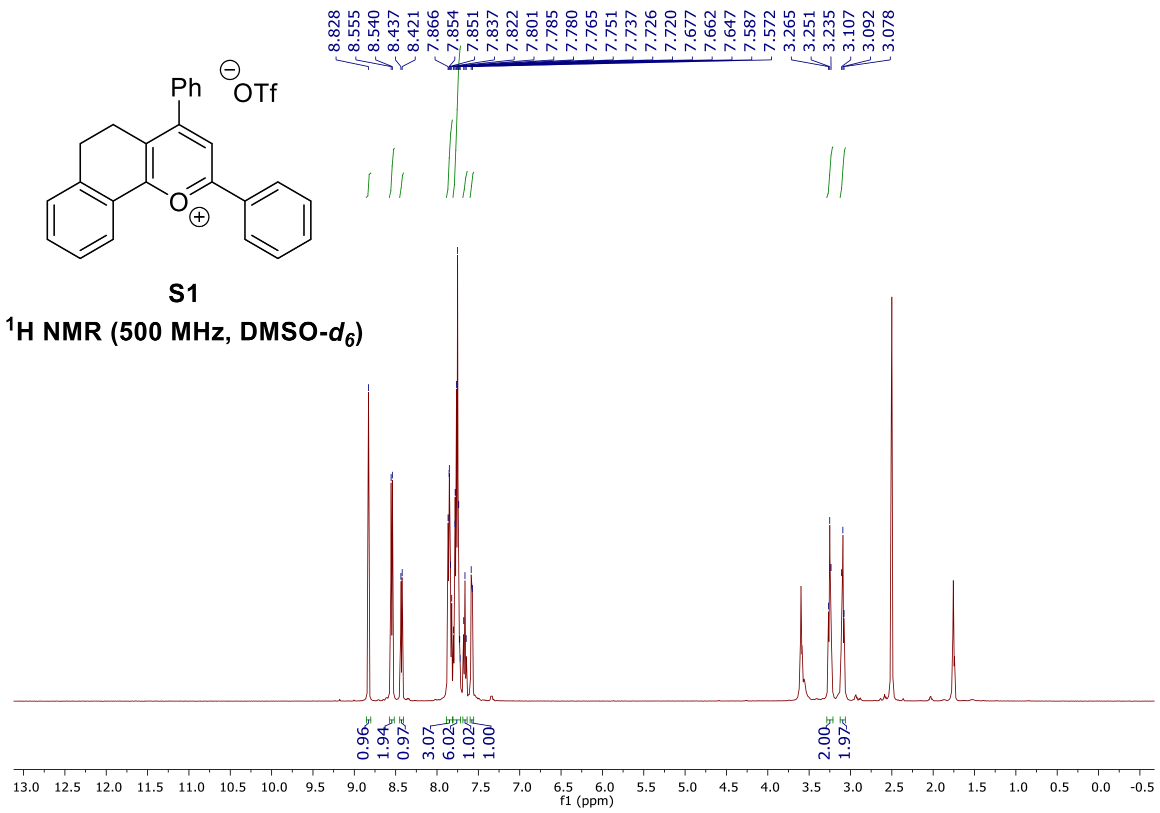

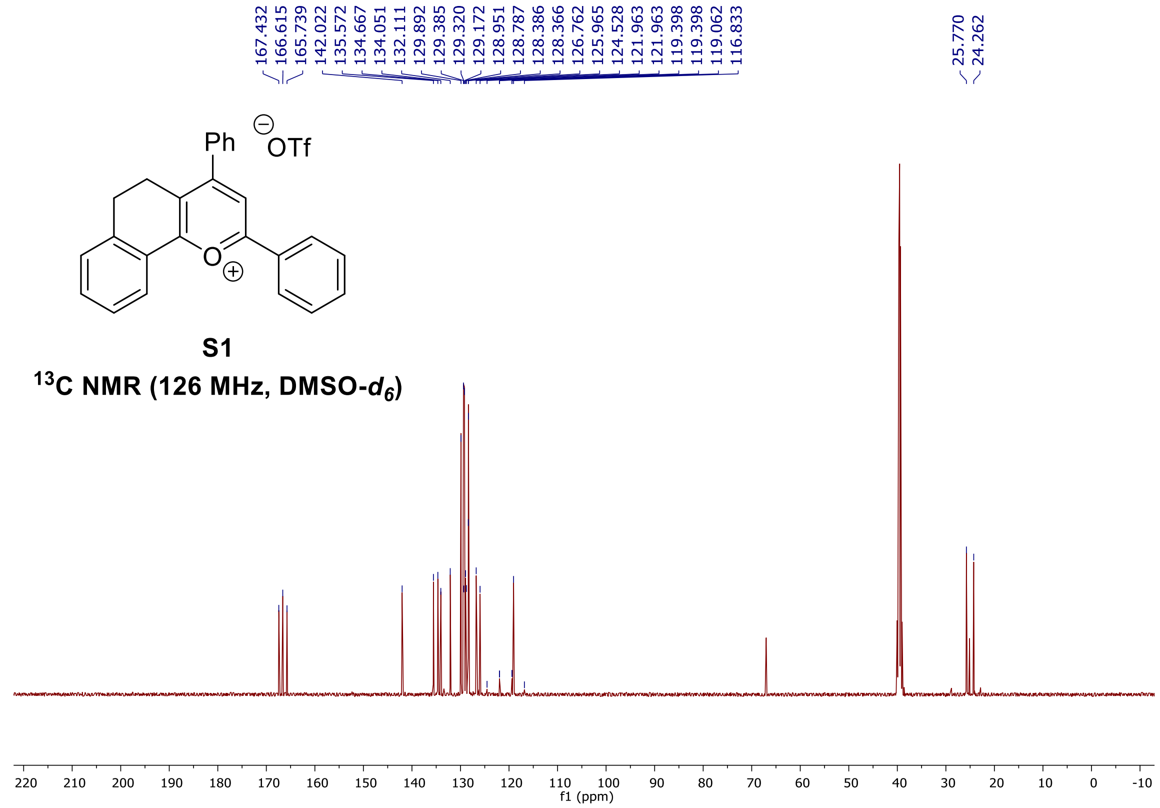

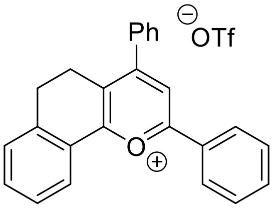

S1

^{19}F NMR (470 MHz, DMSO-*d*$_6$)

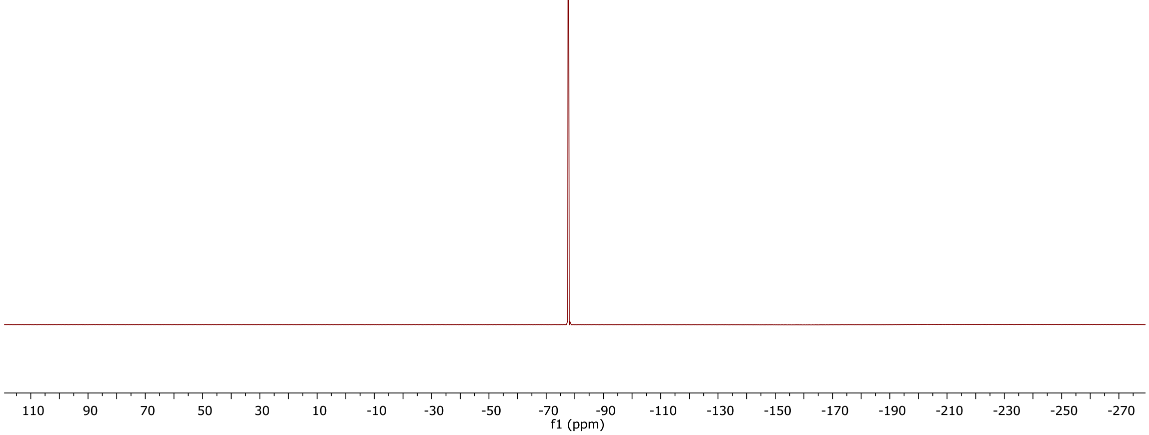

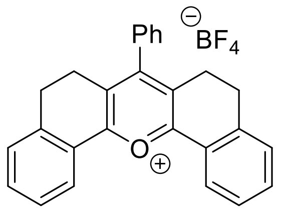

S2

^{1}H NMR (400 MHz, DMSO-d_6)

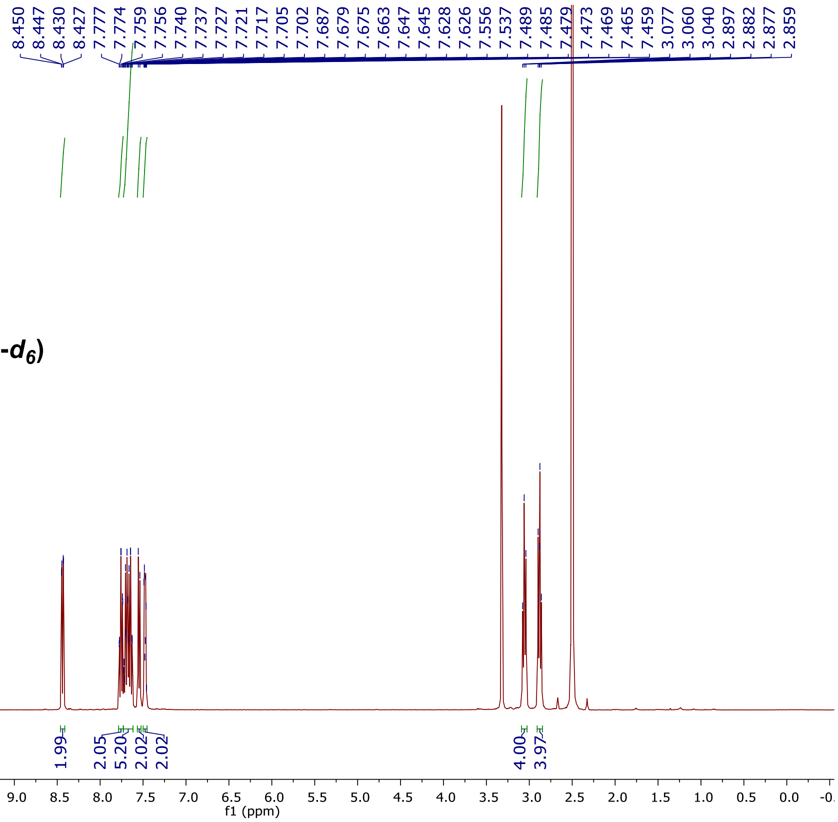

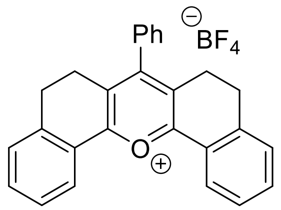

S2

^{13}C NMR (101 MHz, DMSO-d_6)

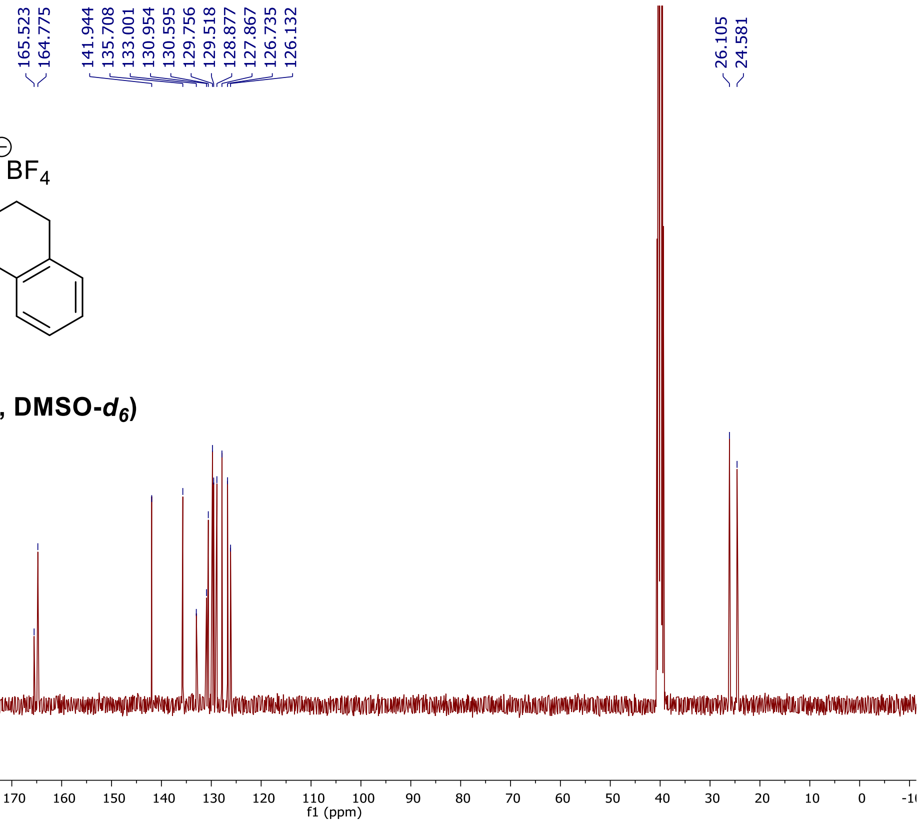

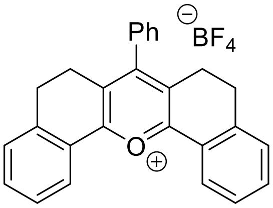

S2

^{11}B NMR (128 MHz, DMSO-*d_6*)

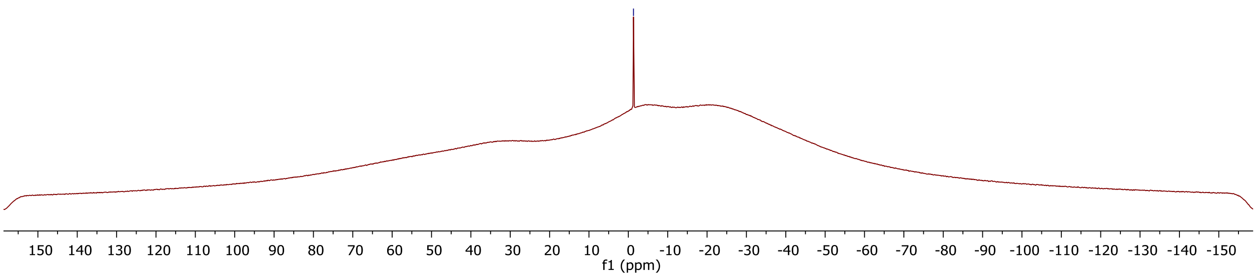

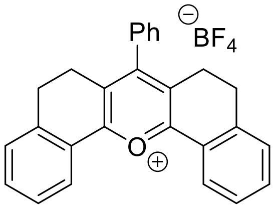

S2

^{19}F NMR (471 MHz, DMSO-*d_6*)

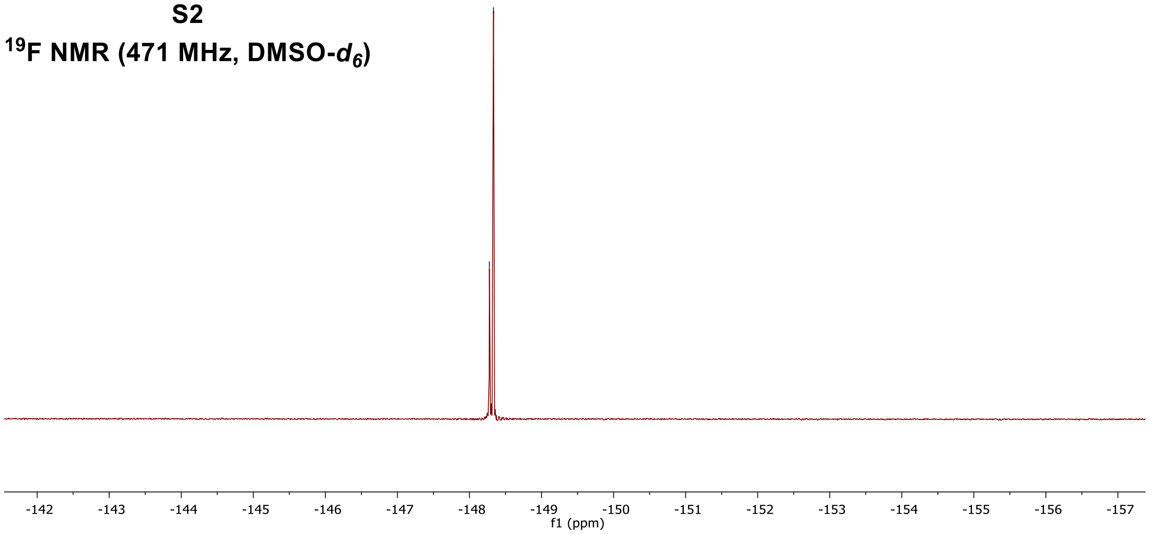

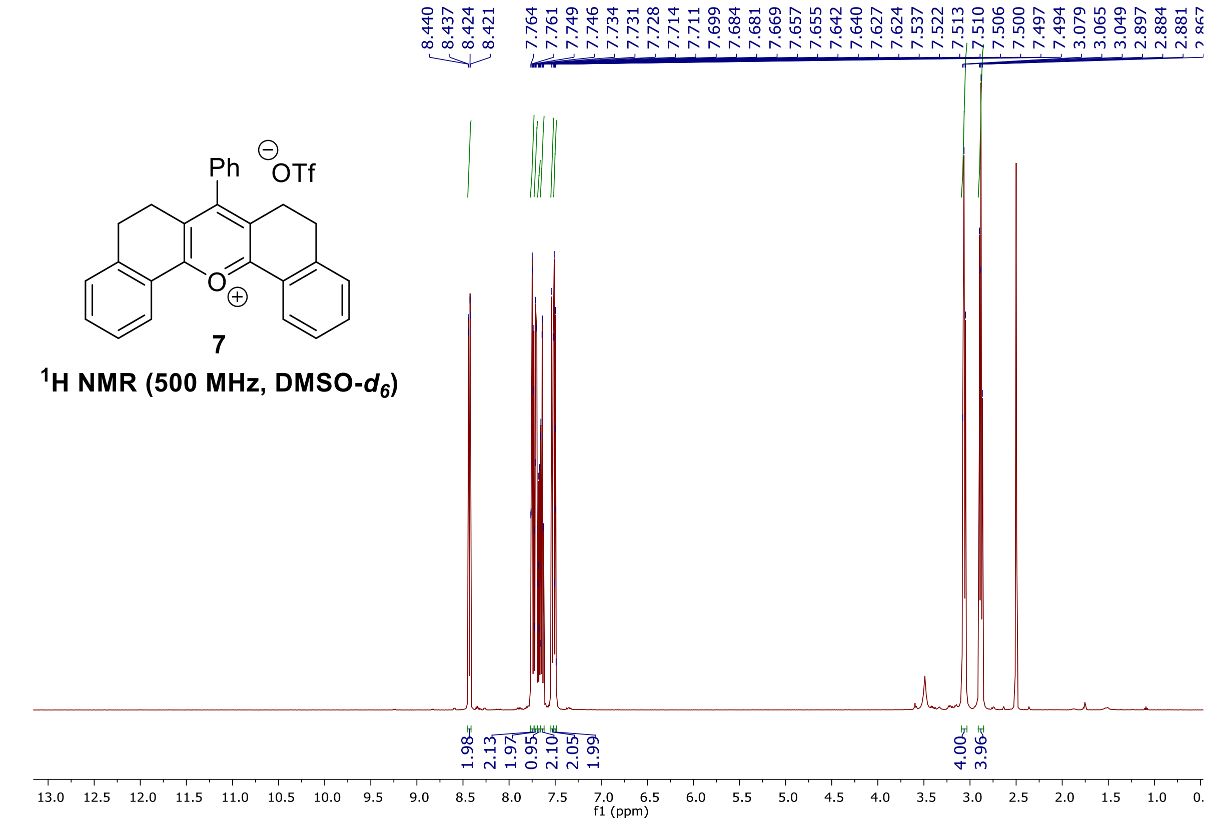
8.440
8.437
8.424
8.421
7.764
7.761
7.749
7.746
7.734
7.731
7.728
7.714
7.711
7.699
7.684
7.681
7.669
7.657
7.655
7.642
7.640
7.627
7.624
7.537
7.522
7.513
7.510
7.506
7.500
7.497
7.494
3.079
3.065
3.049
2.897
2.884
2.881
Ph
OTf
O
7
^{1}H NMR (500 MHz, DMSO-d_6)
1.98
2.13
1.97
0.95
2.10
2.05
1.99
4.00
3.96
f1 (ppm)

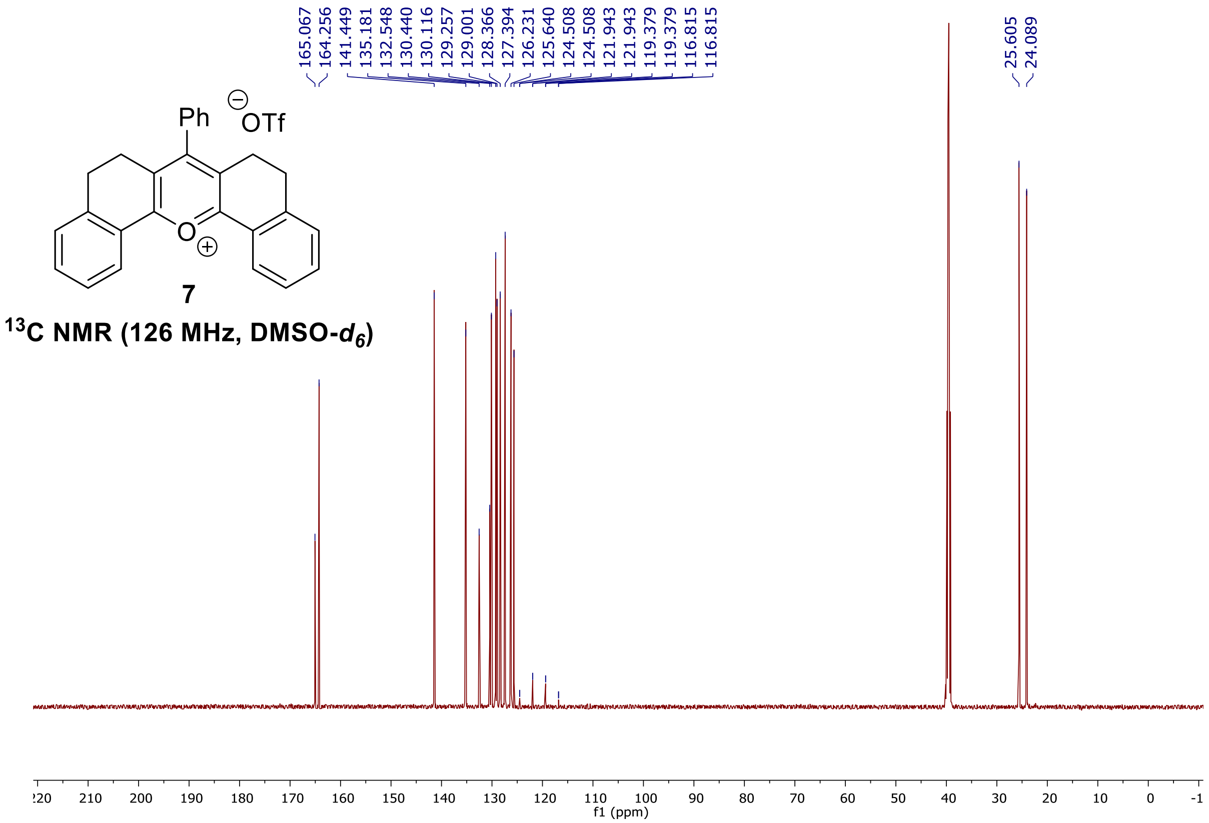
165.067
164.256
141.449
135.181
132.548
130.440
130.116
129.257
129.001
128.366
127.394
126.231
125.640
124.508
124.508
121.943
121.943
119.379
119.379
116.815
116.815
25.605
24.089
Ph
OTf
O
7
^{13}C NMR (126 MHz, DMSO-d_6)
f1 (ppm)

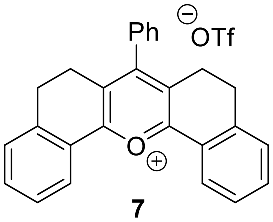

7

^{19}F NMR (470 MHz, DMSO-*d_6*)

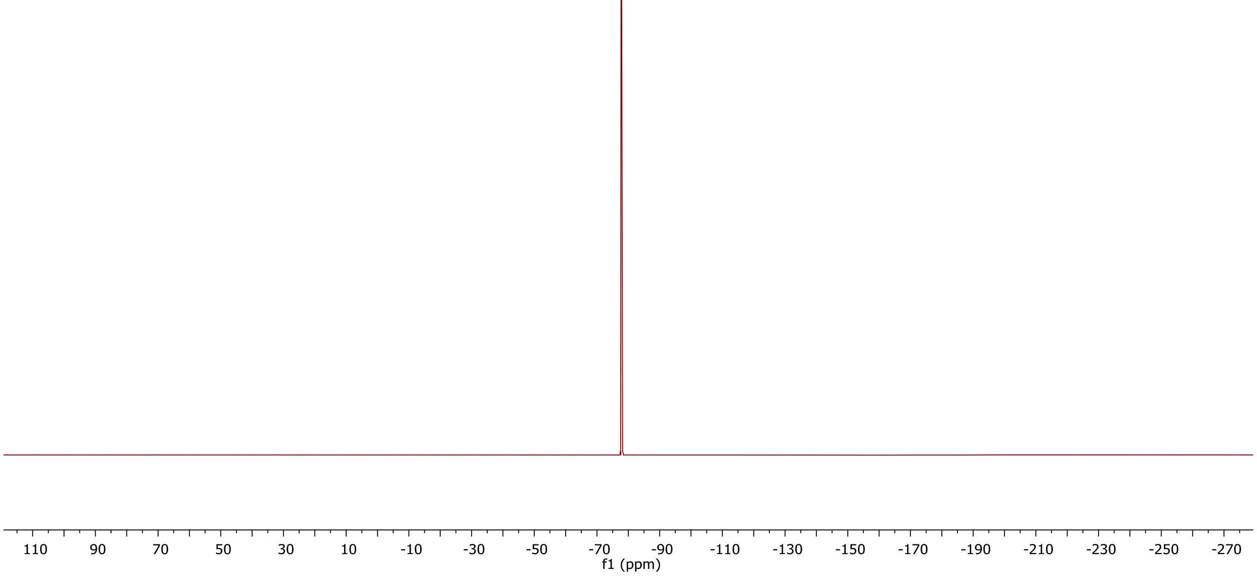

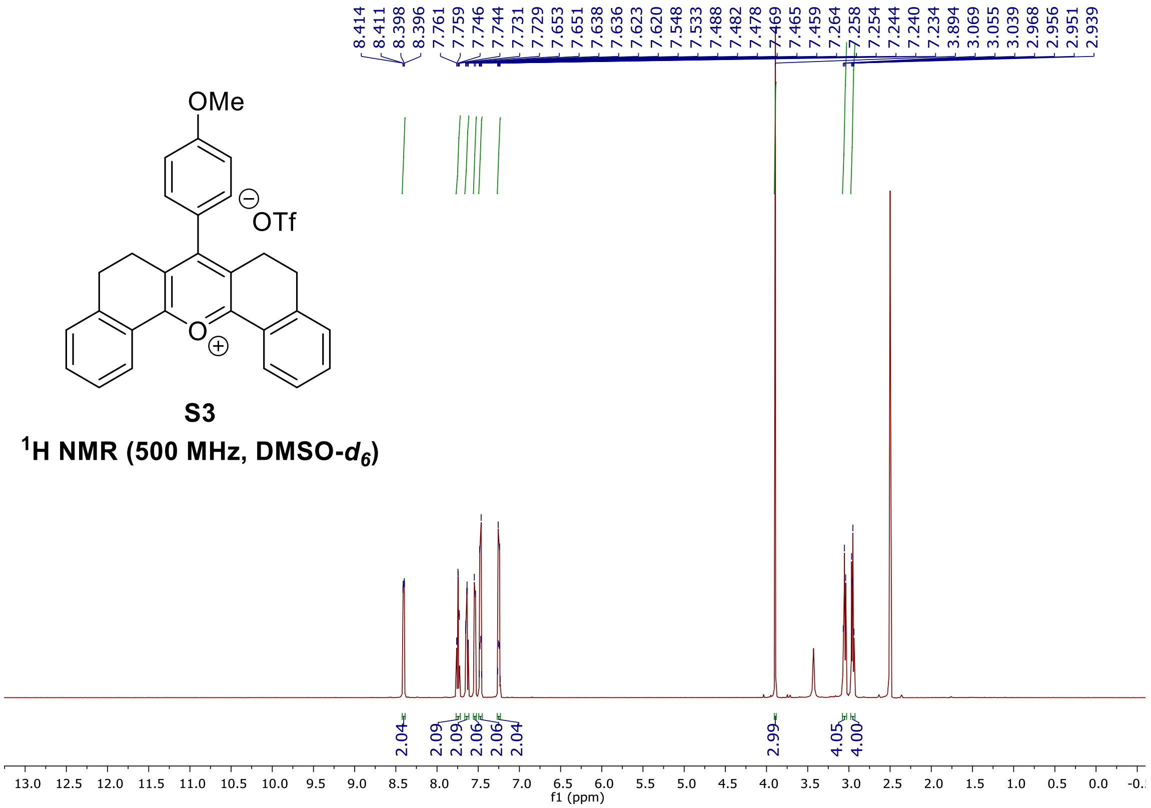

S3

^{1}H NMR (500 MHz, DMSO-d_6)

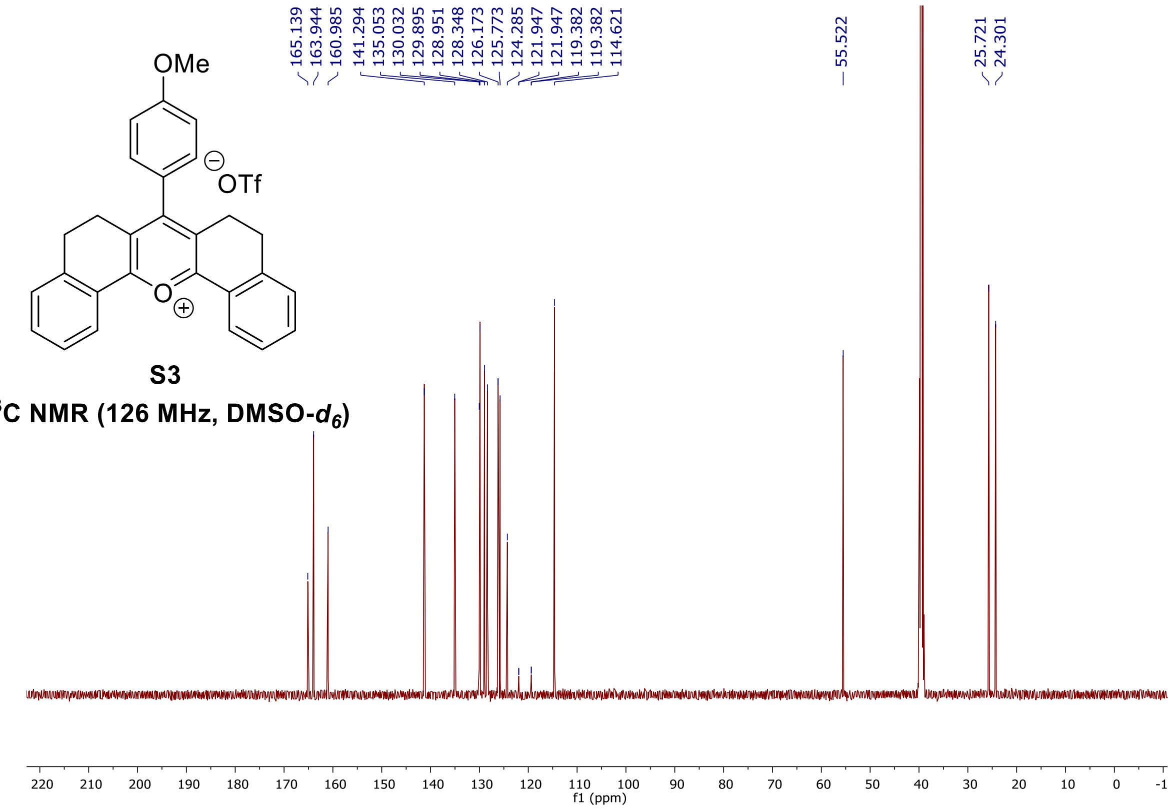

S3

^{13}C NMR (126 MHz, DMSO-d_6)

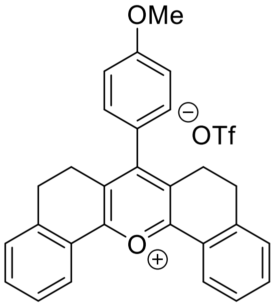

S3

^{19}F NMR (470 MHz, DMSO-*d_6*)

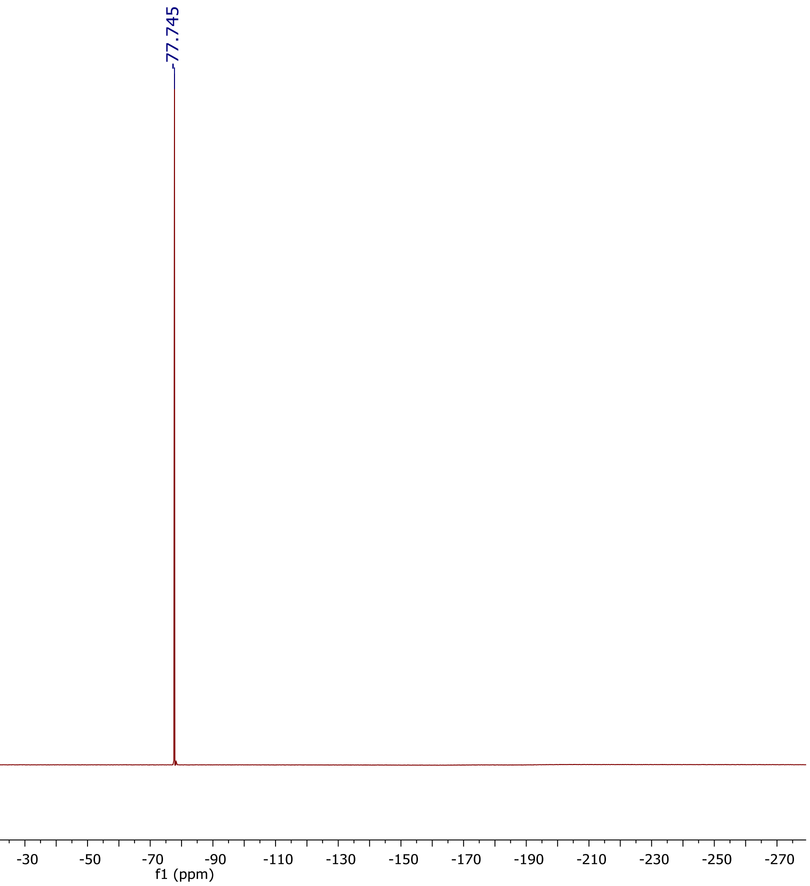

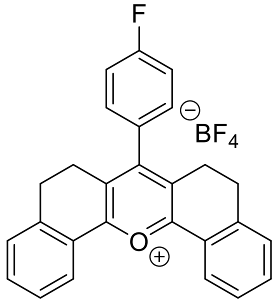

S4

^{1}H NMR (500 MHz, DMSO-d_6)

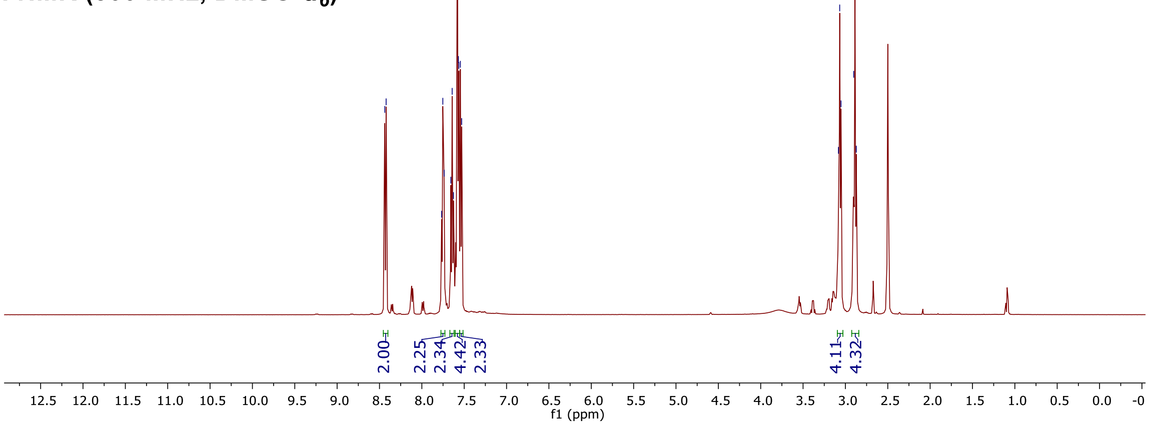

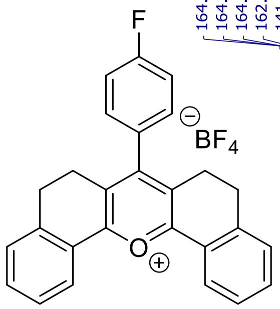

S4

^{13}C NMR (126 MHz, DMSO-d_6)

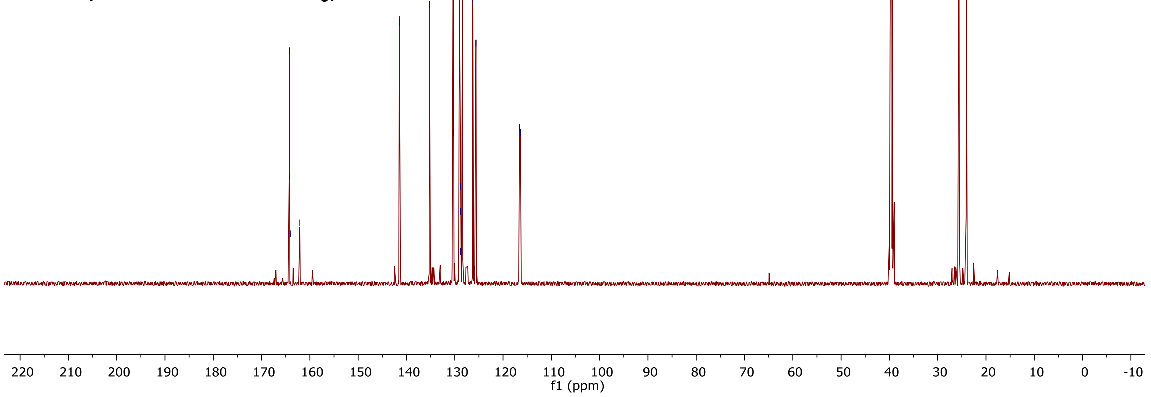

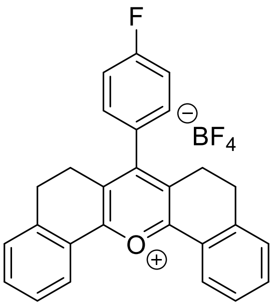

S4

^{11}B NMR (160 MHz, DMSO-d_6)

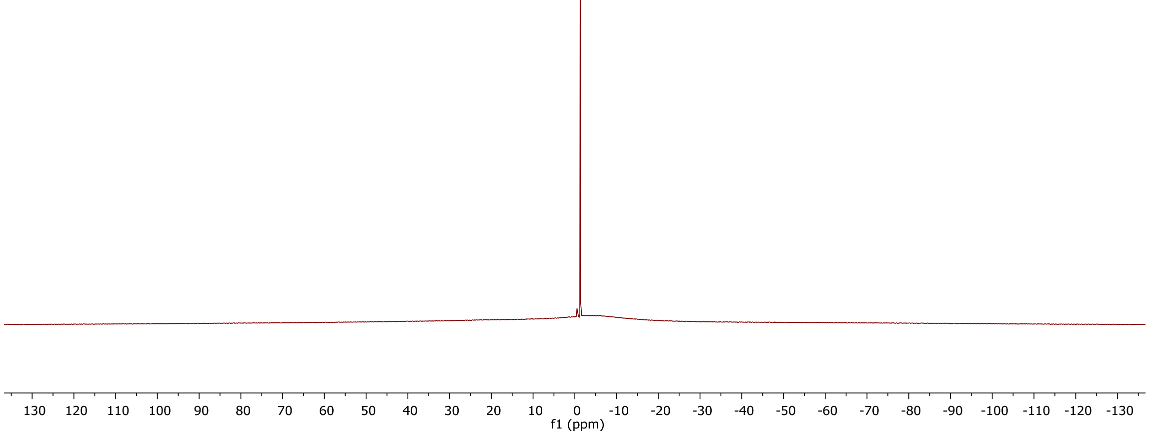

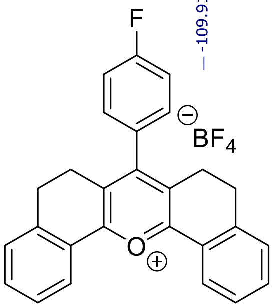

S4

^{19}F NMR (470 MHz, DMSO-d_6)

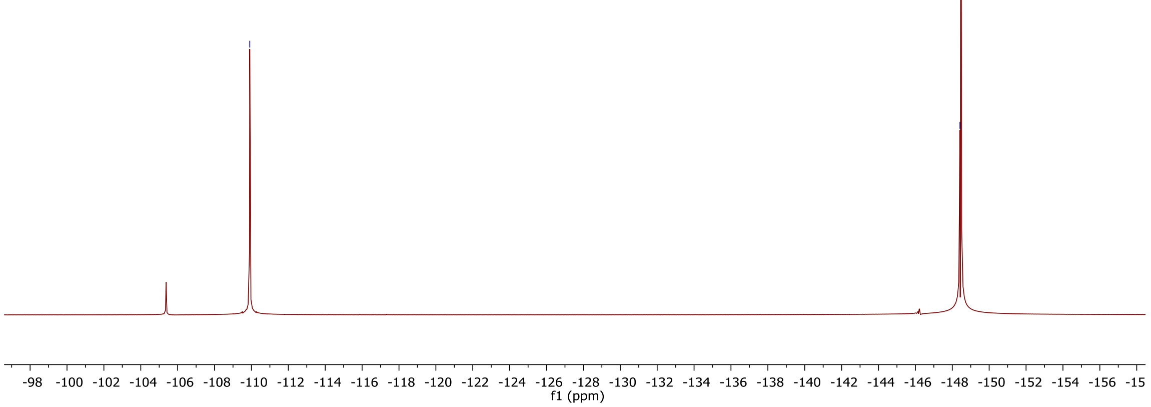

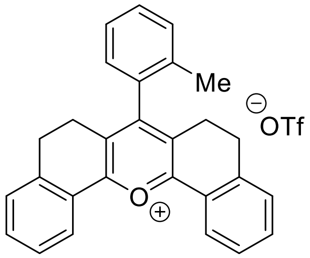

S5

^{1}H NMR (500 MHz, DMSO-d_6)

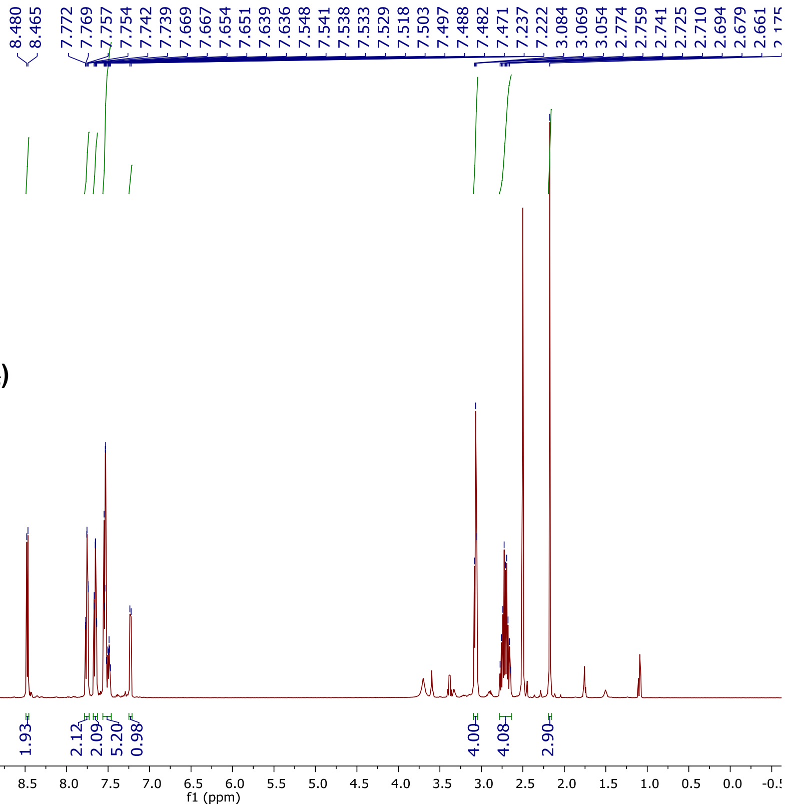

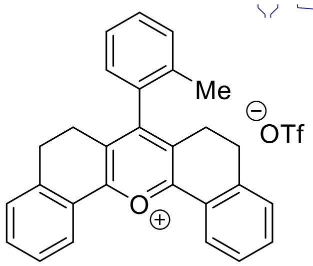

S5

^{13}C NMR (126 MHz, DMSO-d_6)

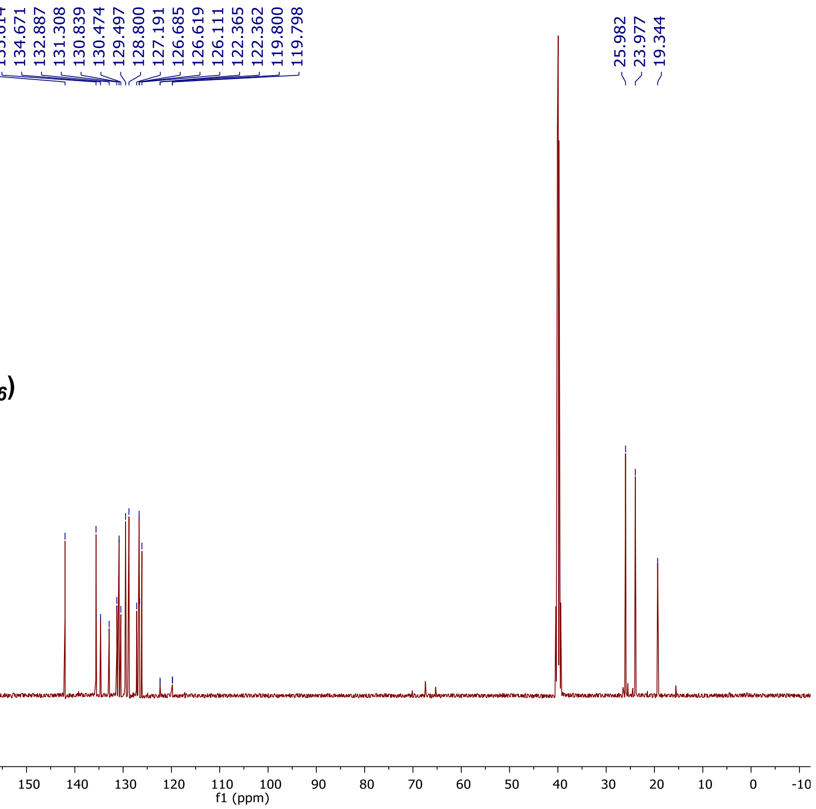

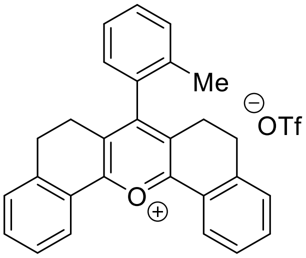

S5

^{19}F NMR (470 MHz, DMSO-d_6)

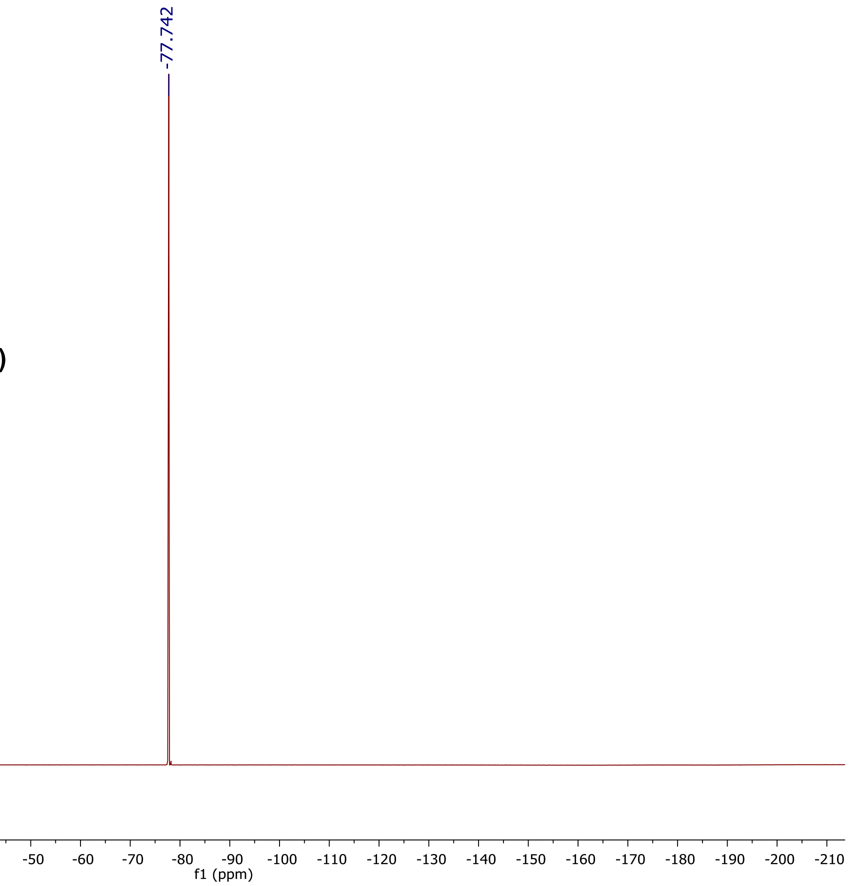

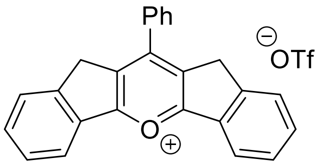

S6

^{1}H NMR (500 MHz, DMSO-d_6)

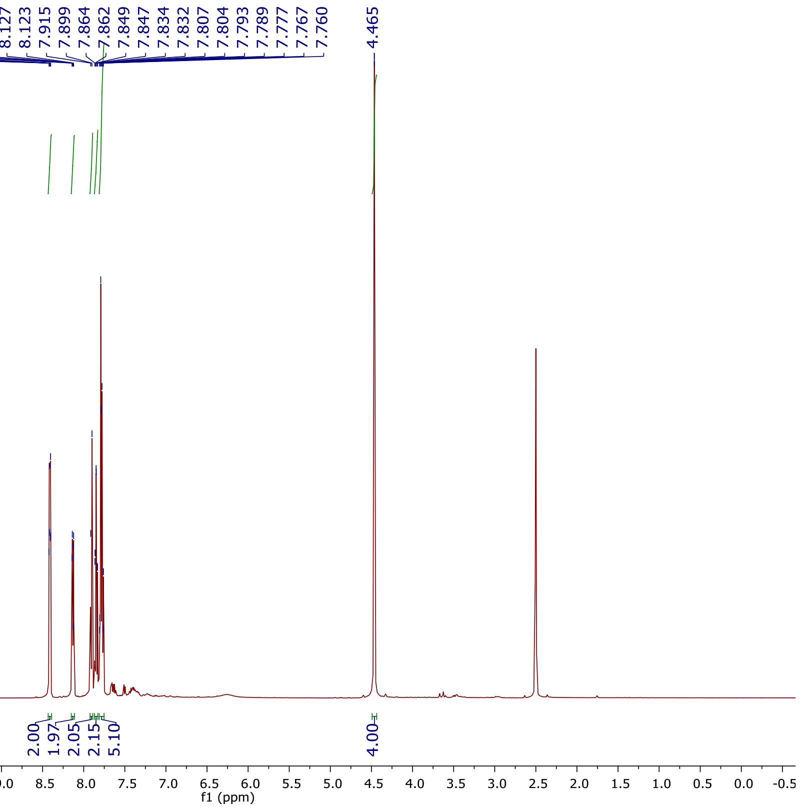

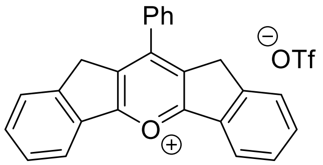

S6

^{13}C NMR (126 MHz, DMSO-d_6)

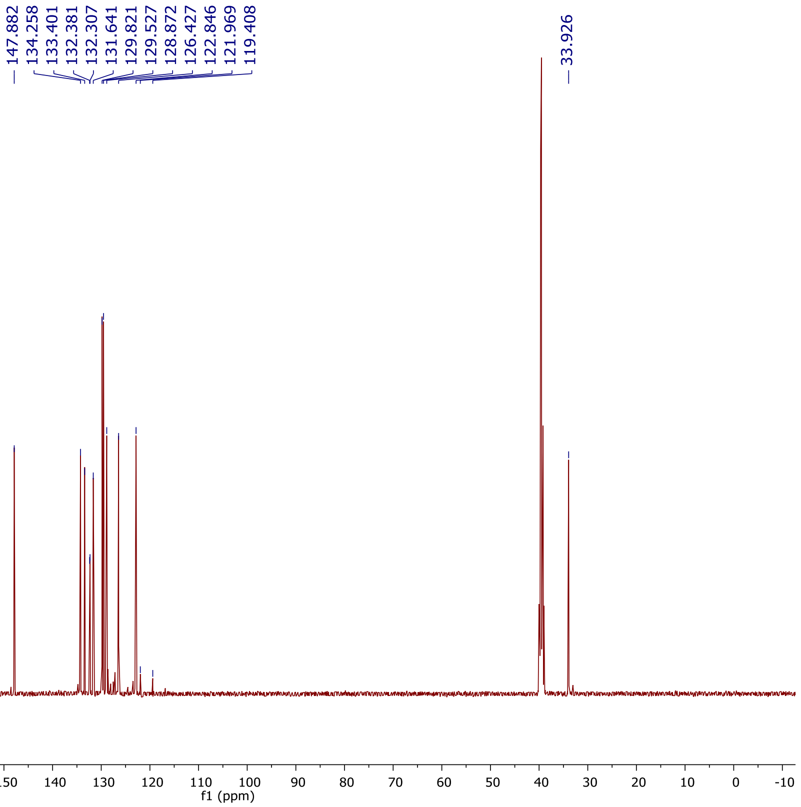

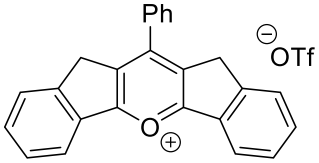

S6

^{19}F NMR (470 MHz, DMSO-d_6)

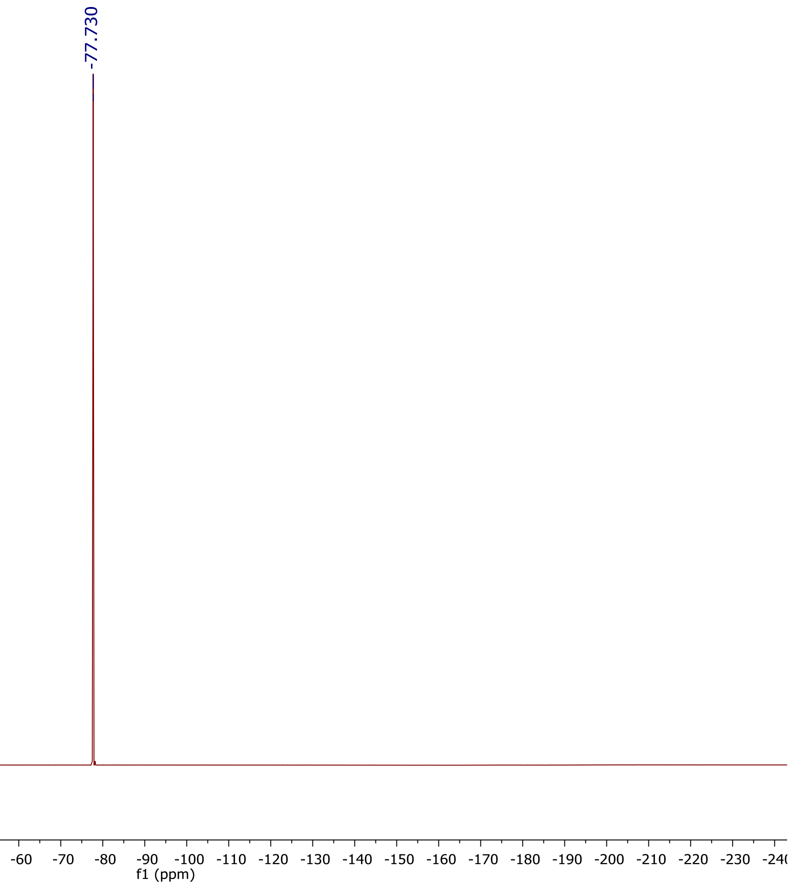

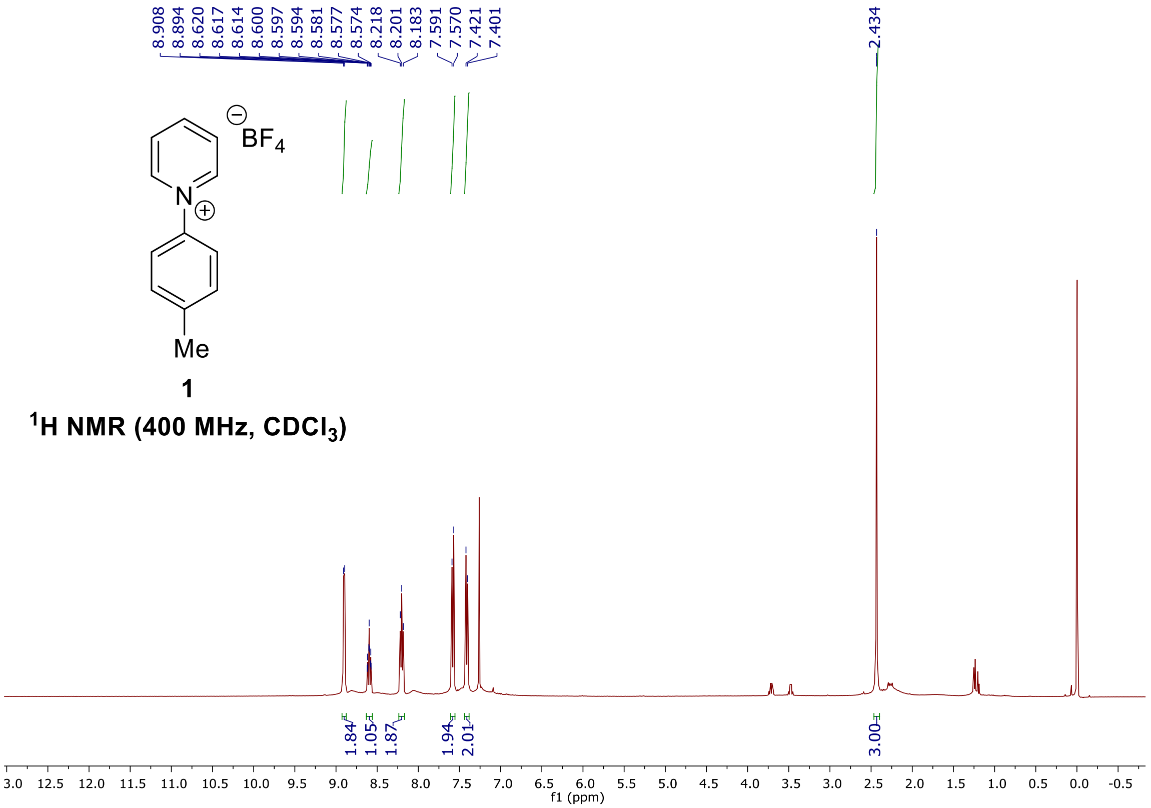

1

^{1}H NMR (400 MHz, $CDCl_3$)

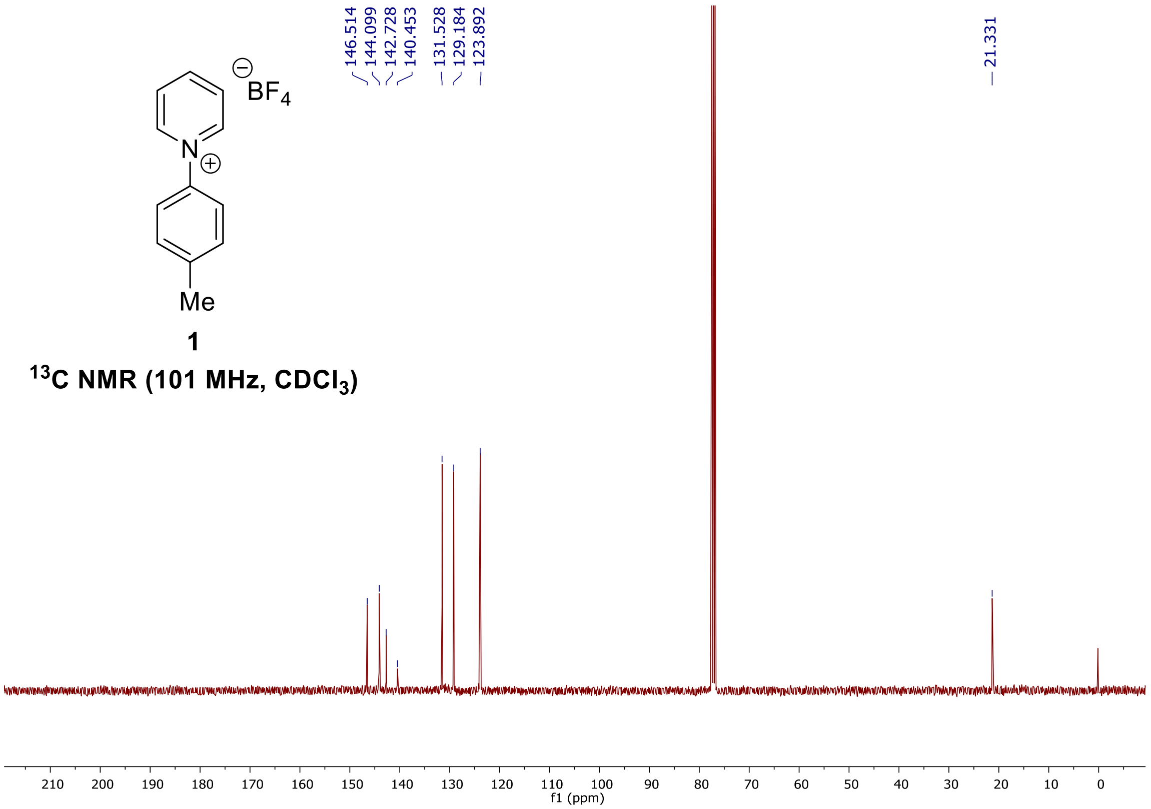

1

^{13}C NMR (101 MHz, $CDCl_3$)

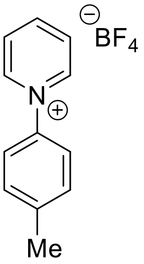

1

^{11}B NMR (160 MHz, $CDCl_3$)

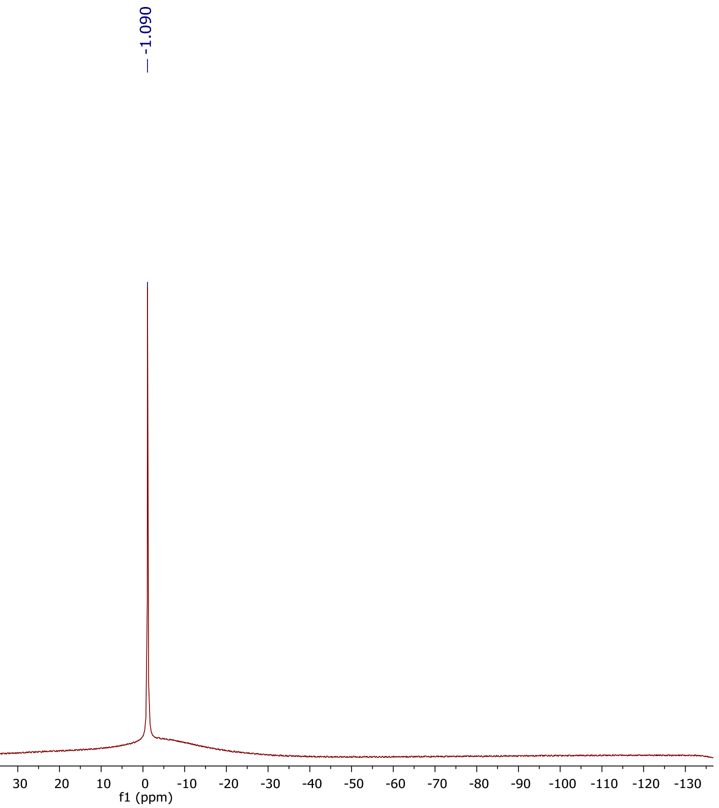

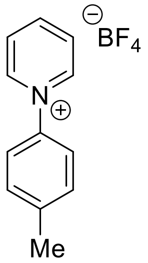

1

^{19}F NMR (470 MHz, $CDCl_3$)

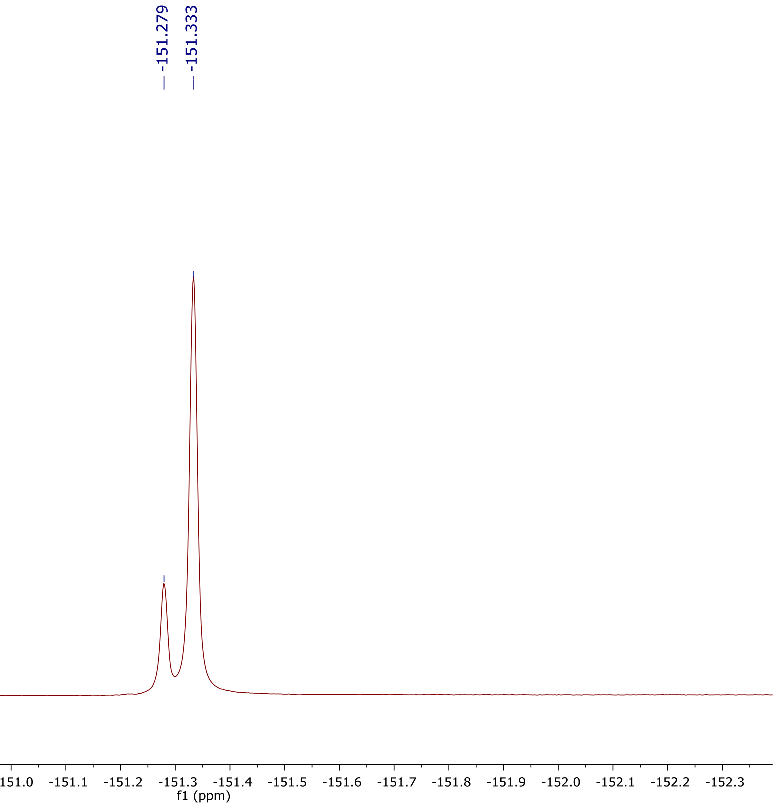

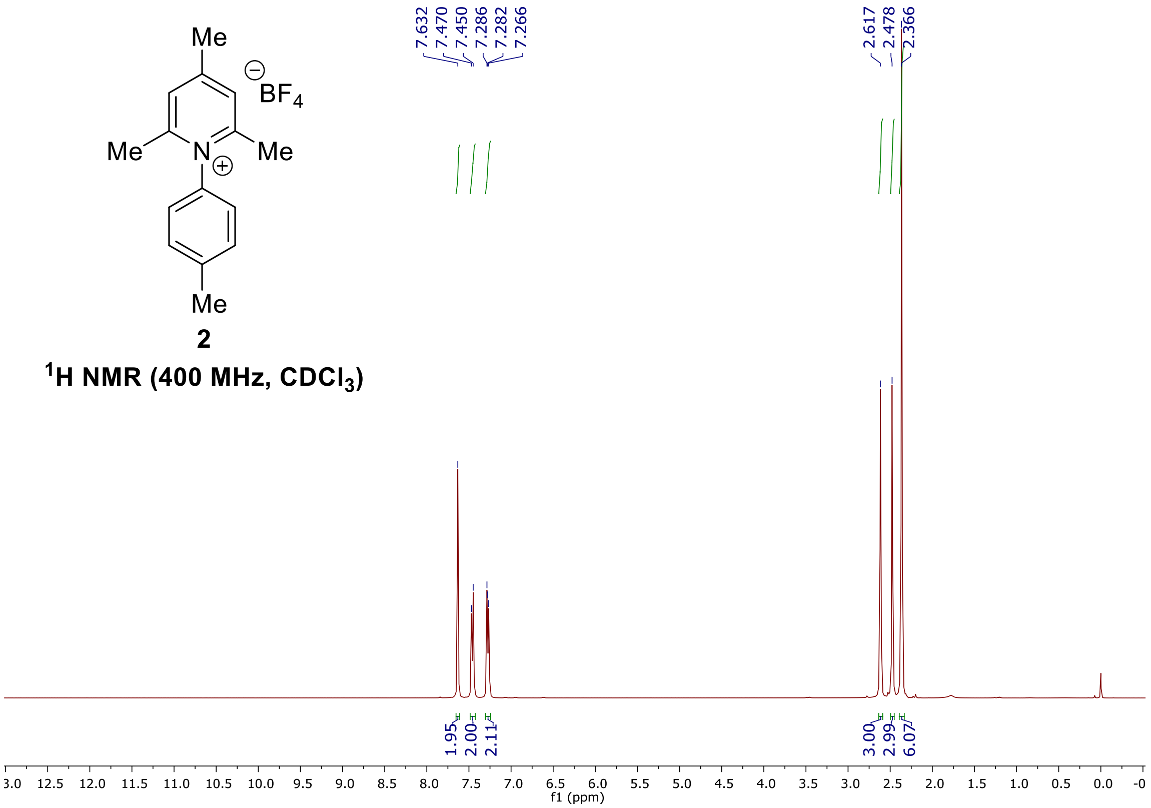

2

^{1}H NMR (400 MHz, $CDCl_3$)

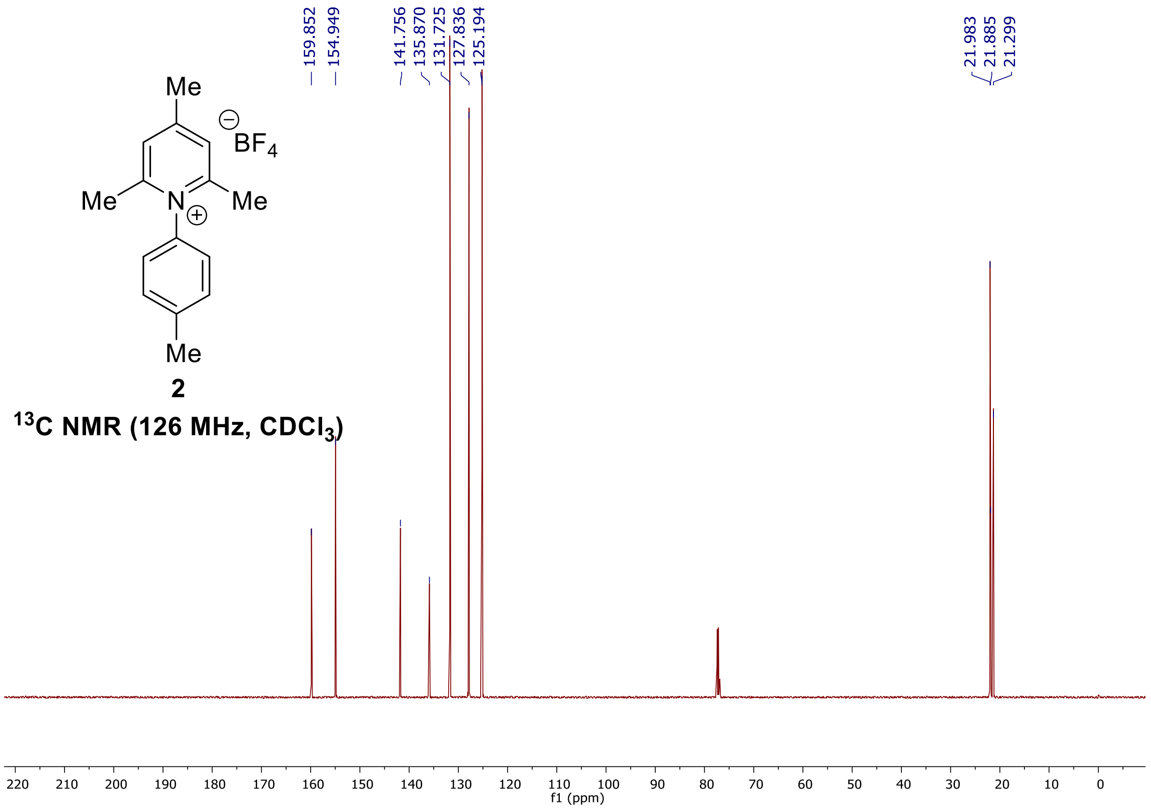

2

^{13}C NMR (126 MHz, $CDCl_3$)

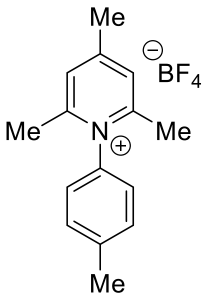

2

^{11}B NMR (160 MHz, $CDCl_3$)

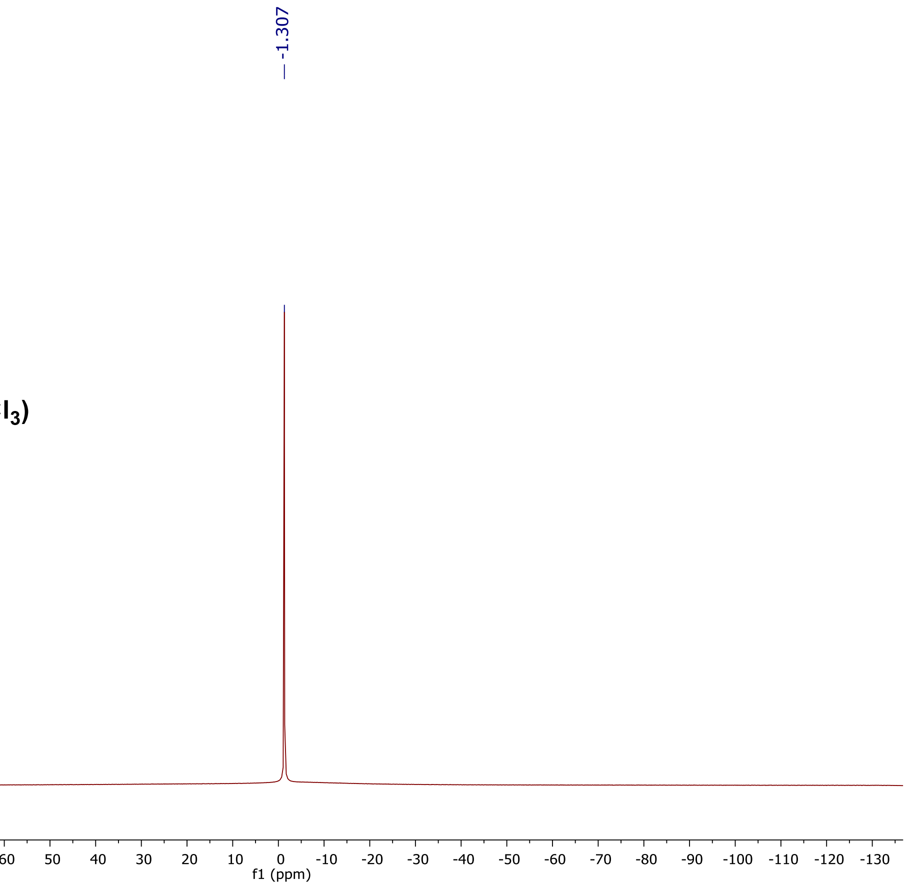

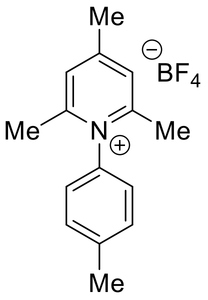

2

^{19}F NMR (470 MHz, DMSO-d_6)

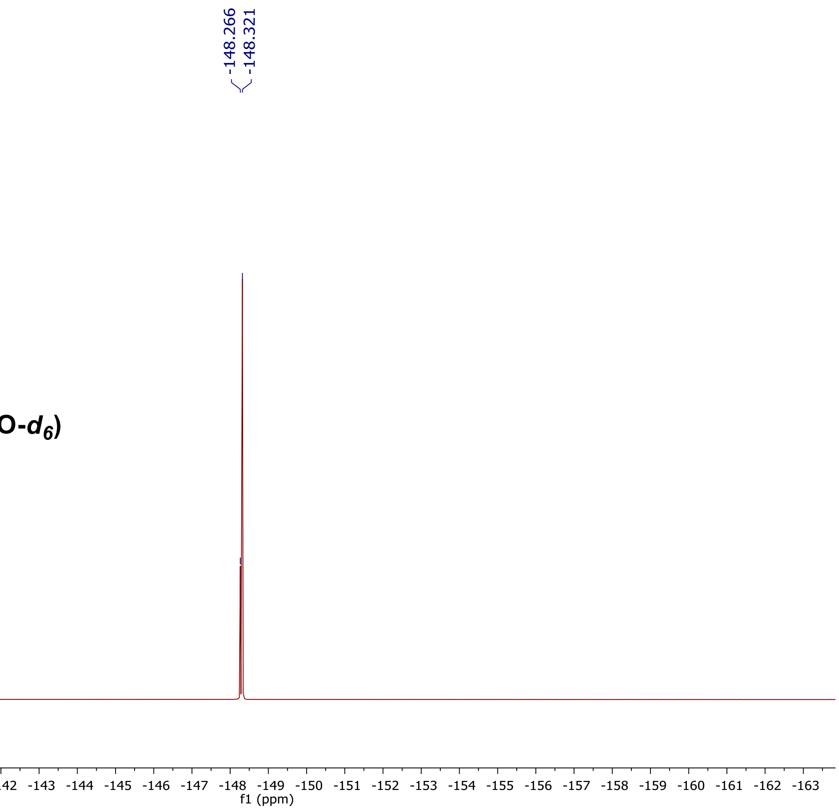

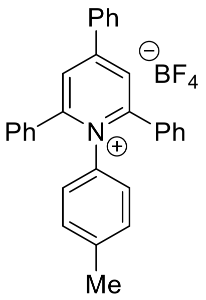

3

^{1}H NMR (500 MHz, DMSO-d_6)

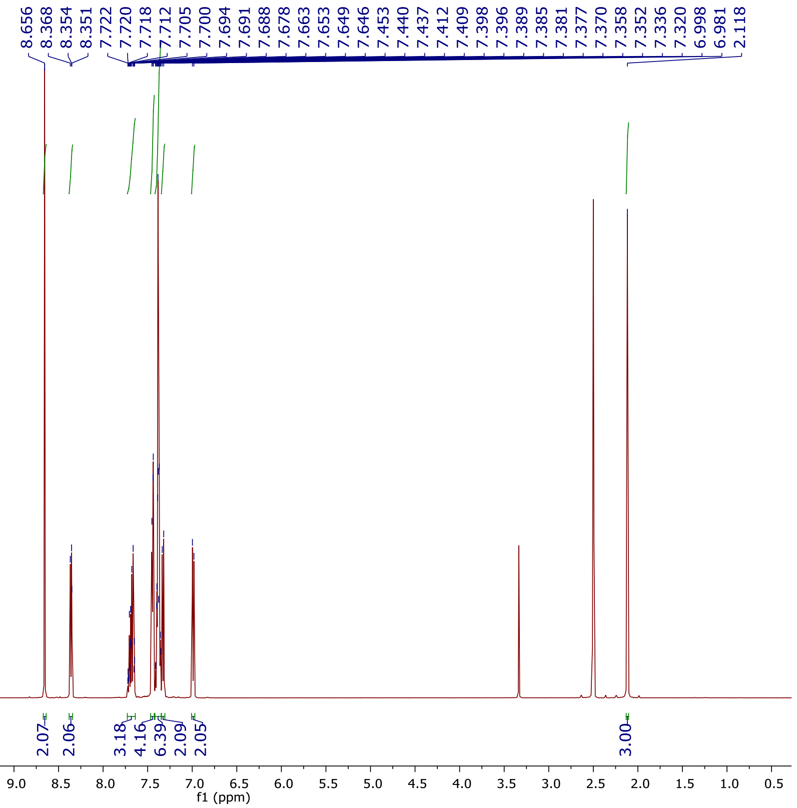

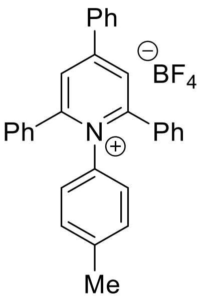

3

^{13}C NMR (126 MHz, DMSO-d_6)

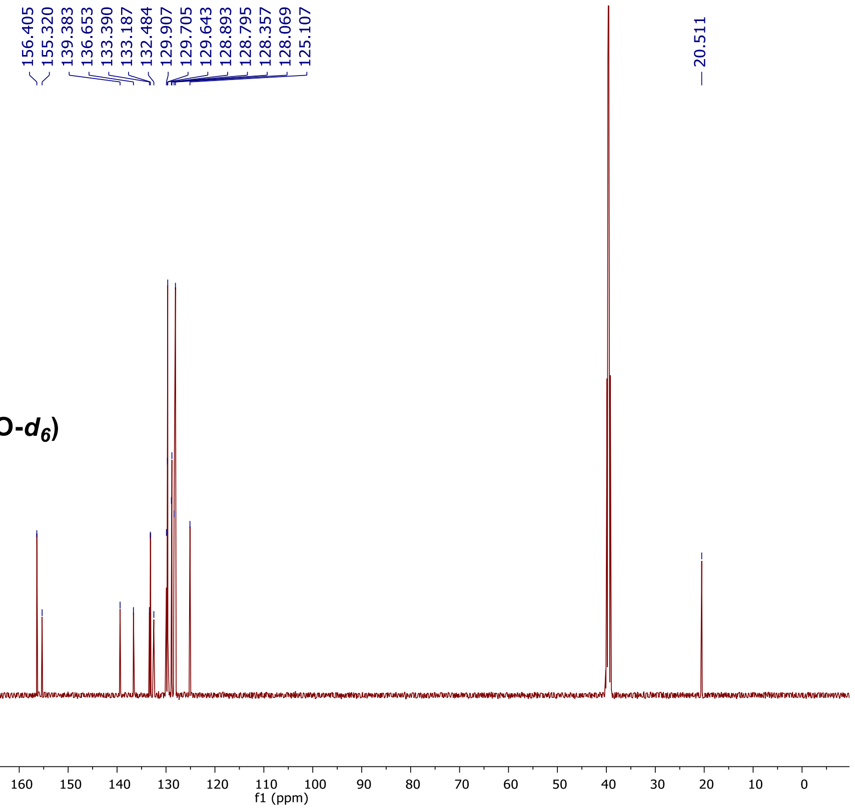

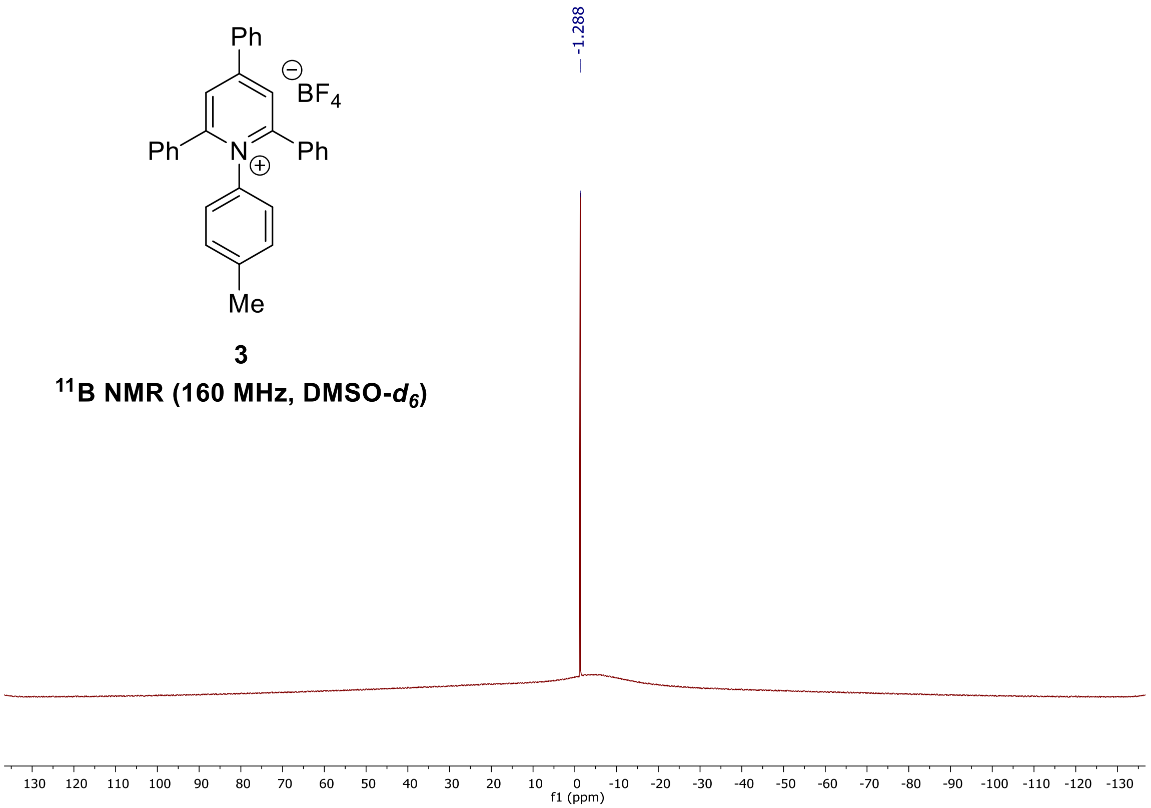

3

^{11}B NMR (160 MHz, DMSO-*d_6*)

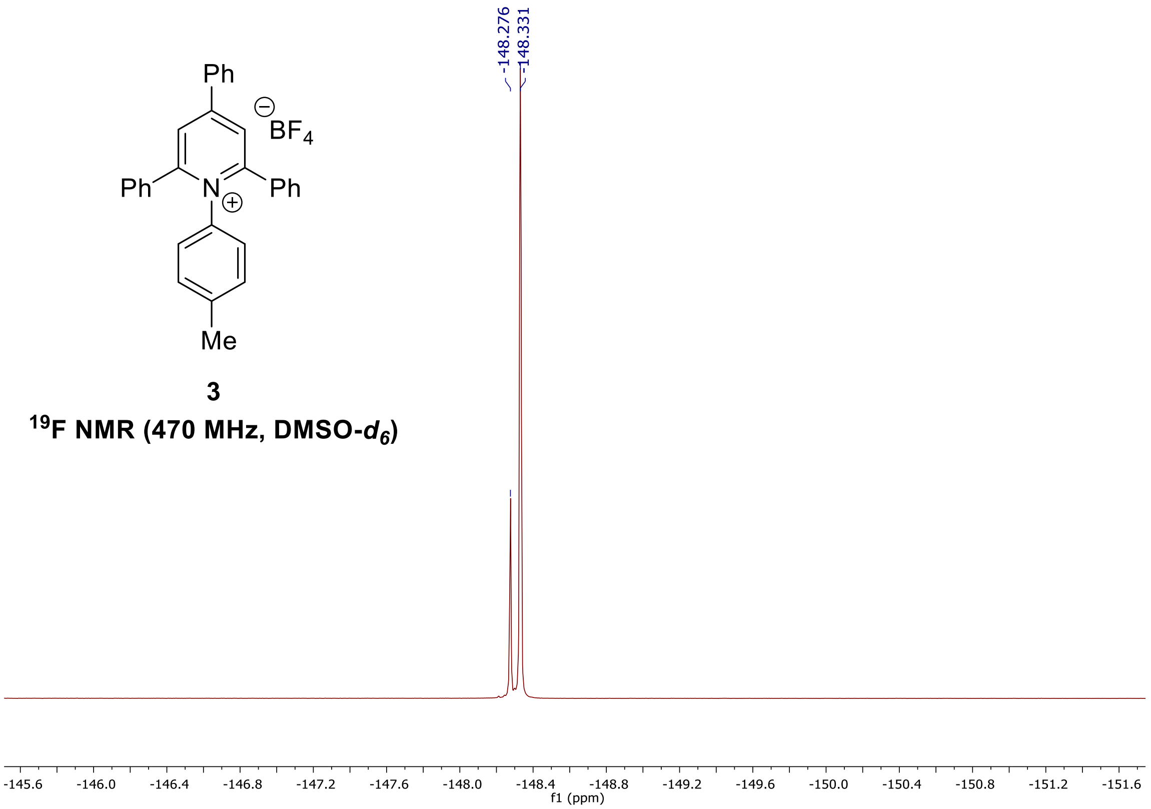

3

^{19}F NMR (470 MHz, DMSO-*d_6*)

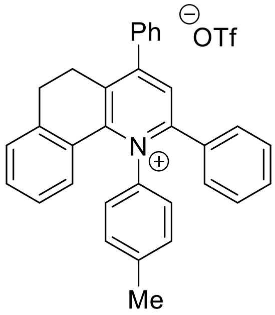

S7

^{1}H NMR (500 MHz, DMSO-d_6)

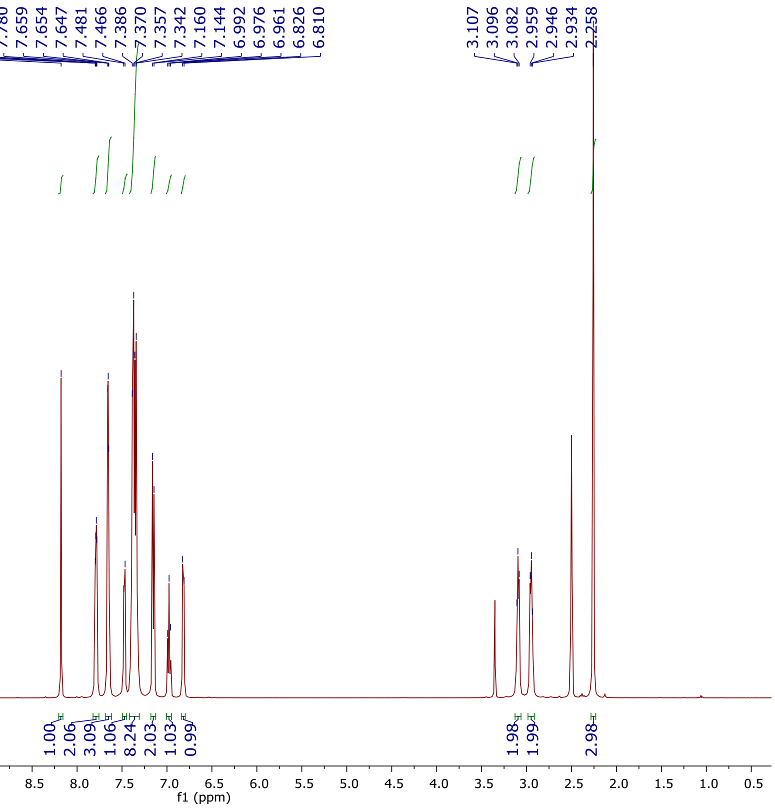

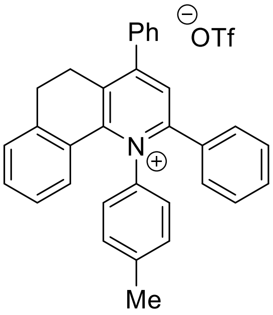

S7

^{13}C NMR (126 MHz, DMSO-d_6)

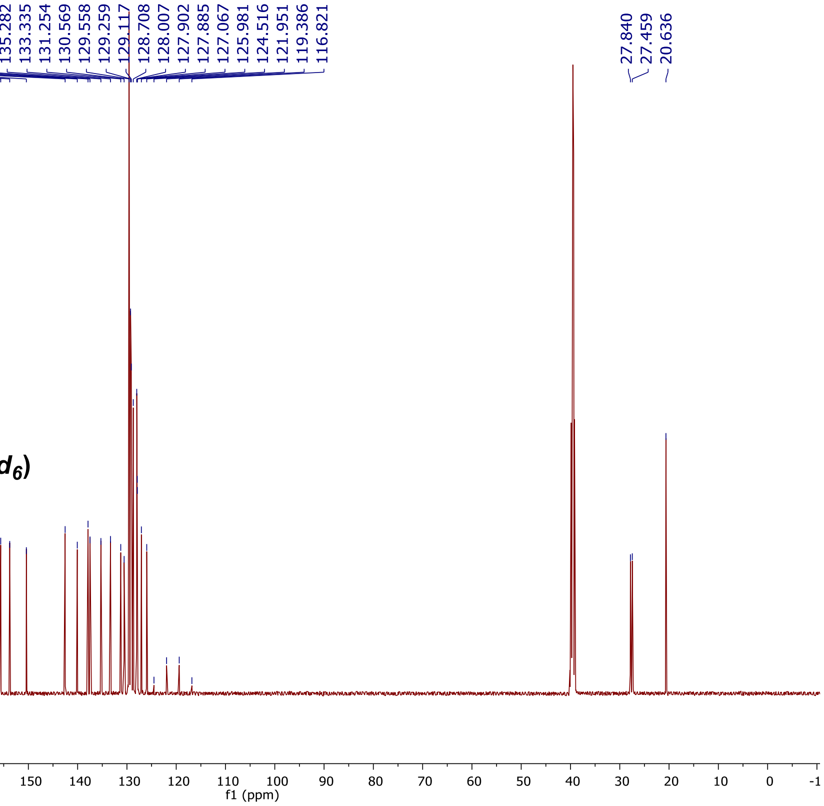

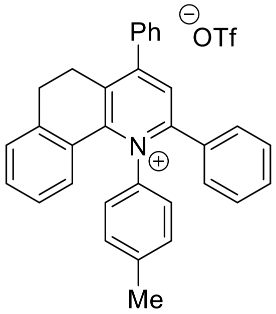

S7

^{19}F NMR (470 MHz, DMSO-d_6)

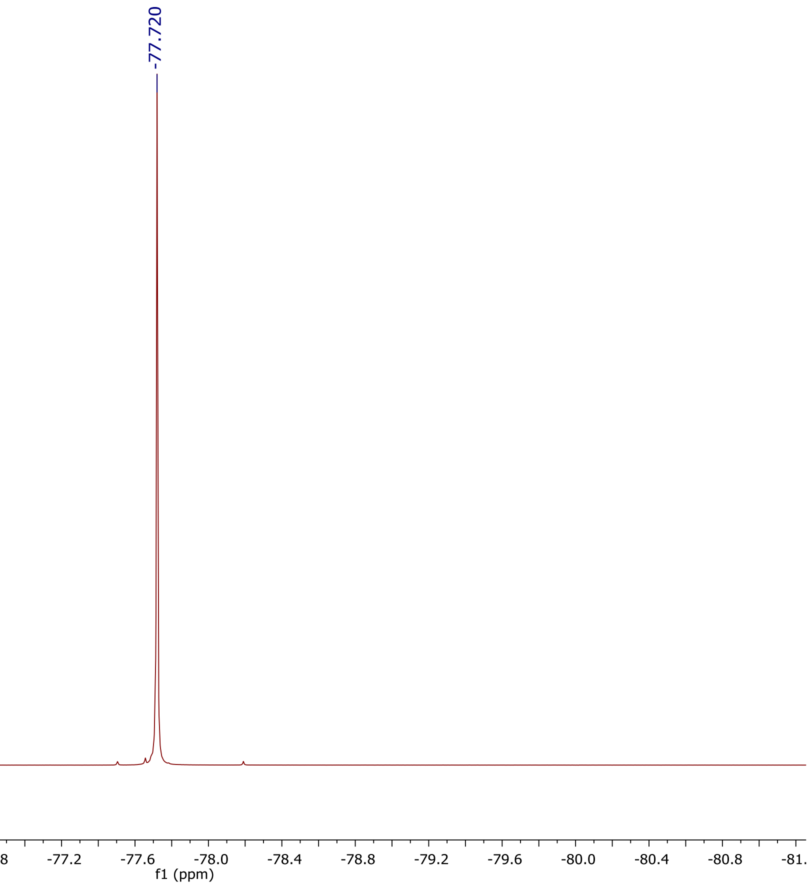

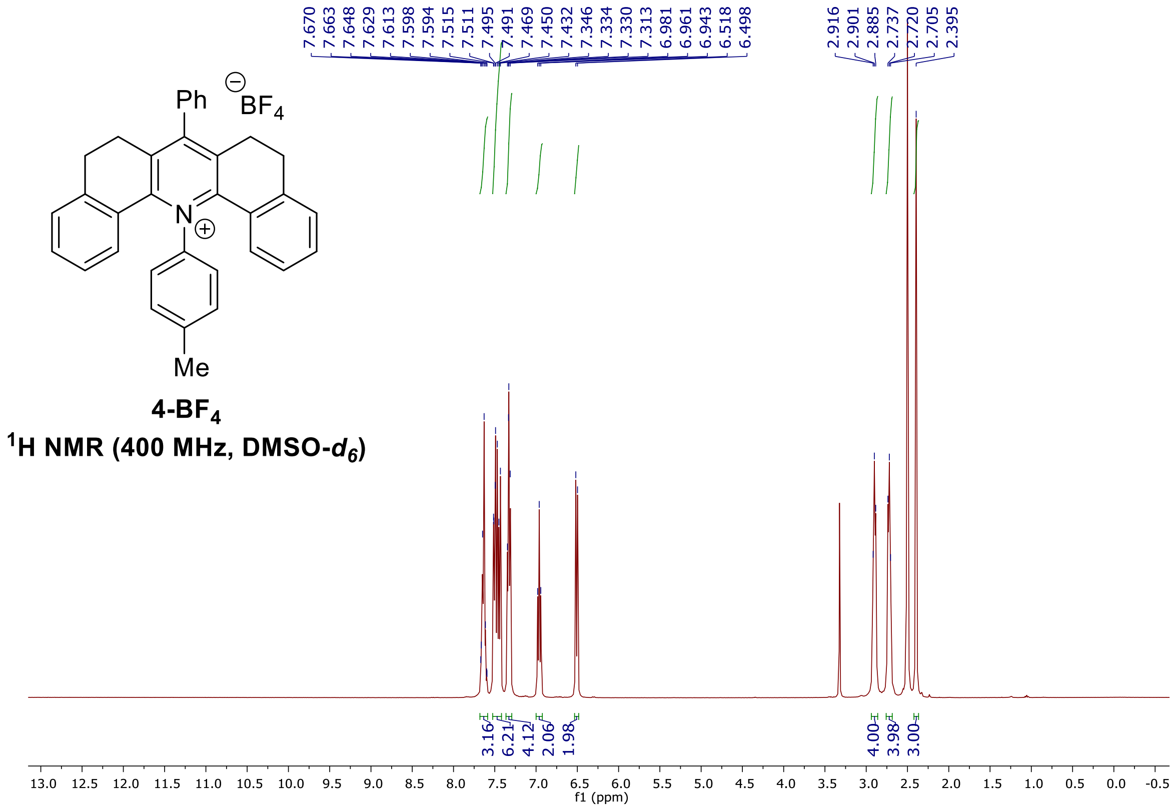

Ph
BF4
N
Me
4-BF4
1H NMR (400 MHz, DMSO-d6)
7.670 7.663 7.648 7.629 7.613 7.598 7.594 7.515 7.511 7.495 7.491 7.469 7.450 7.432 7.346 7.334 7.330 7.313 6.981 6.961 6.943 6.518 6.498
2.916 2.901 2.885 2.737 2.720 2.705 2.395
3.16 6.21 4.12 2.06 1.98 4.00 3.98 3.00
13.0 12.5 12.0 11.5 11.0 10.5 10.0 9.5 9.0 8.5 8.0 7.5 7.0 6.5 6.0 5.5 5.0 4.5 4.0 3.5 3.0 2.5 2.0 1.5 1.0 0.5 0.0 -0.5
f1 (ppm)

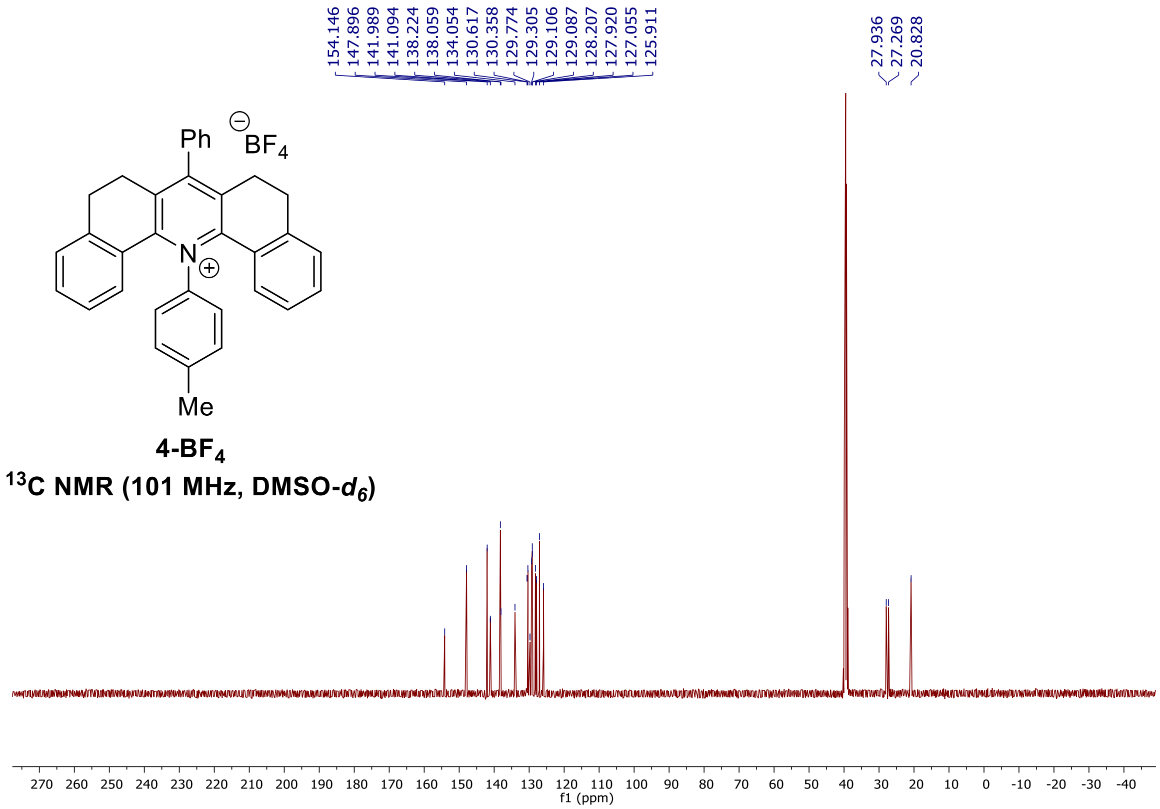

Ph
BF4
N
Me
4-BF4
13C NMR (101 MHz, DMSO-d6)
154.146 147.896 141.989 141.094 138.224 138.059 134.054 130.617 130.358 129.774 129.305 129.106 129.087 128.207 127.920 127.055 125.911
27.936 27.269 20.828
270 260 250 240 230 220 210 200 190 180 170 160 150 140 130 120 110 100 90 80 70 60 50 40 30 20 10 0 -10 -20 -30 -40
f1 (ppm)

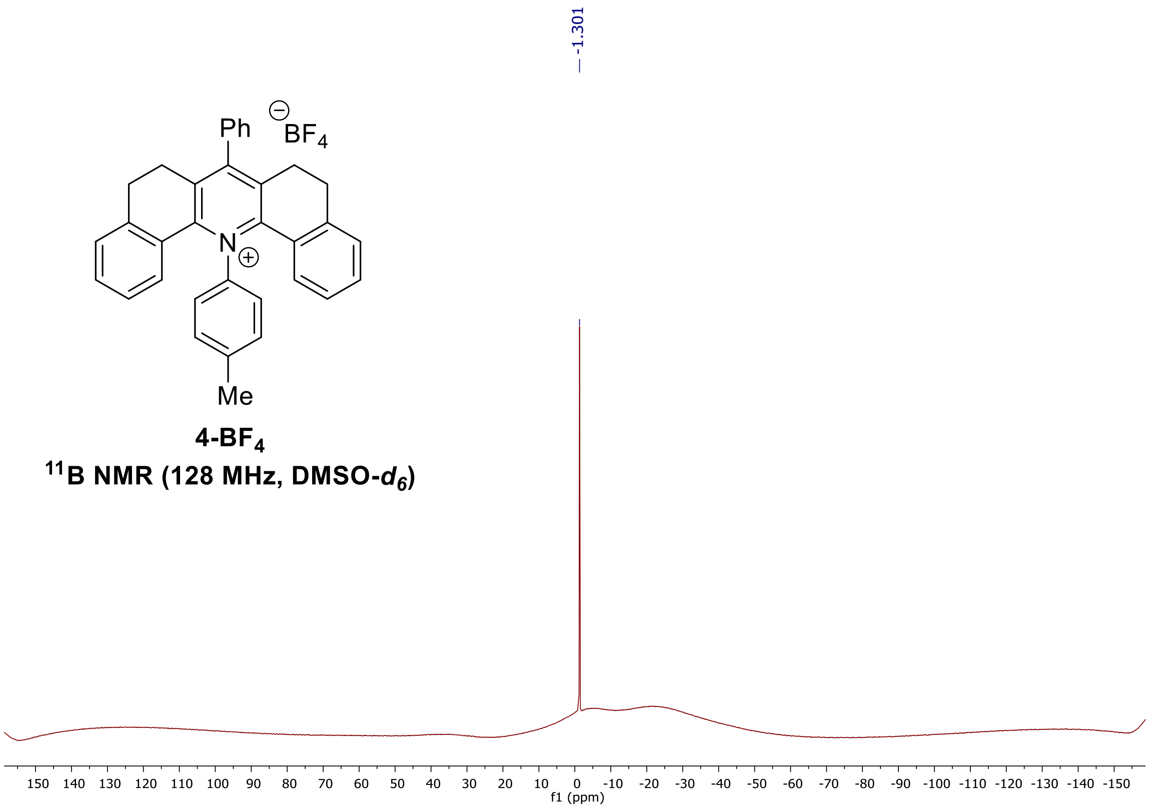

4-BF_4

^{11}B NMR (128 MHz, DMSO-*d_6*)

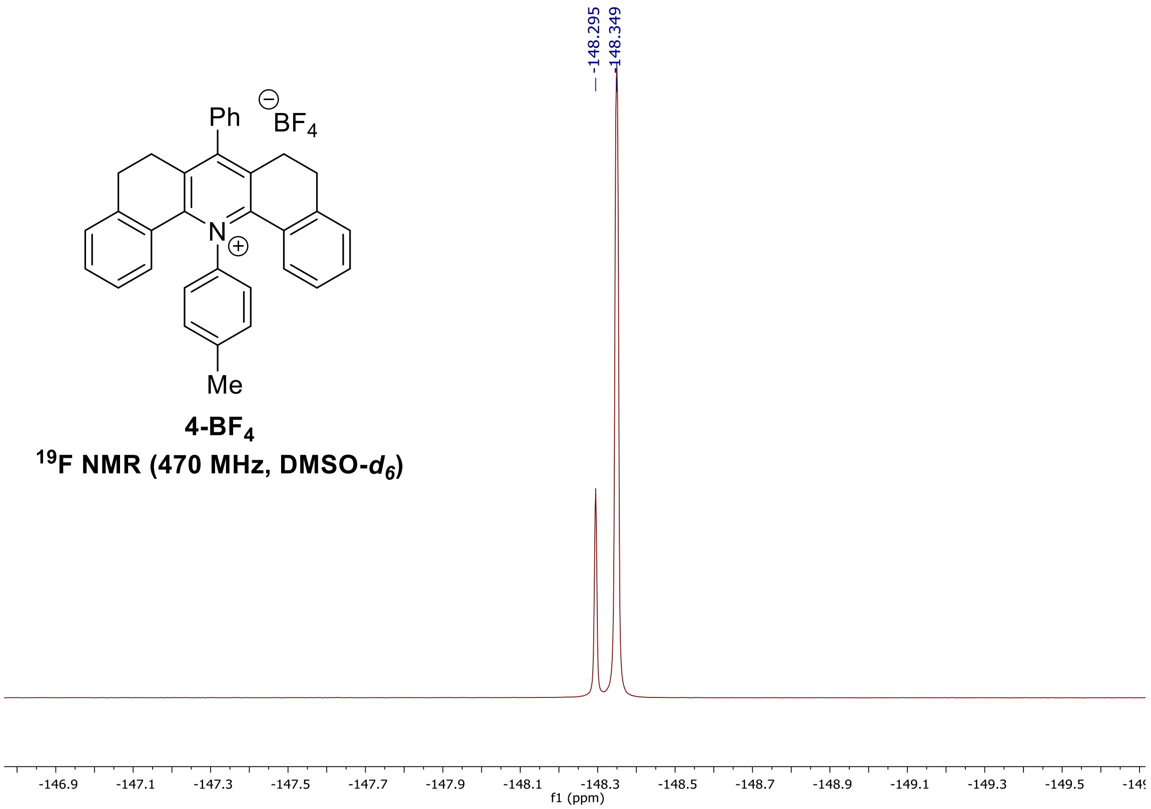

4-BF_4

^{19}F NMR (470 MHz, DMSO-*d_6*)

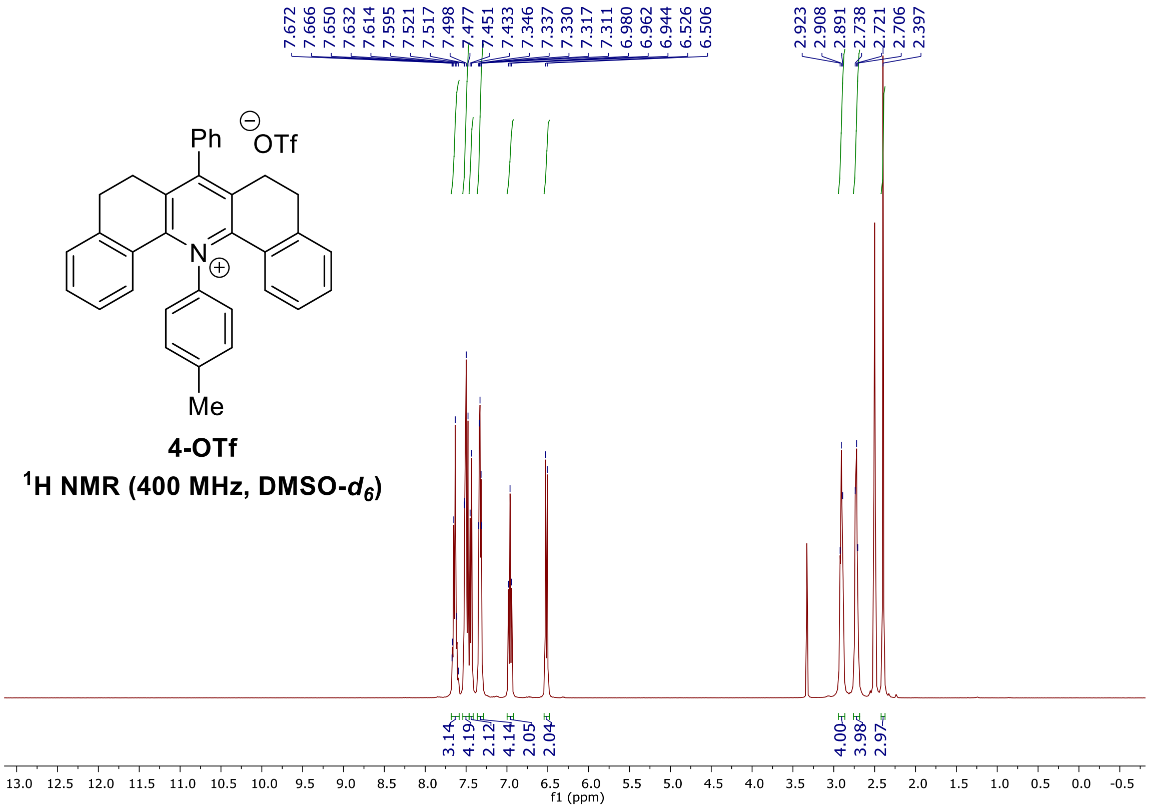

4-OTf

^{1}H NMR (400 MHz, DMSO-d_6)

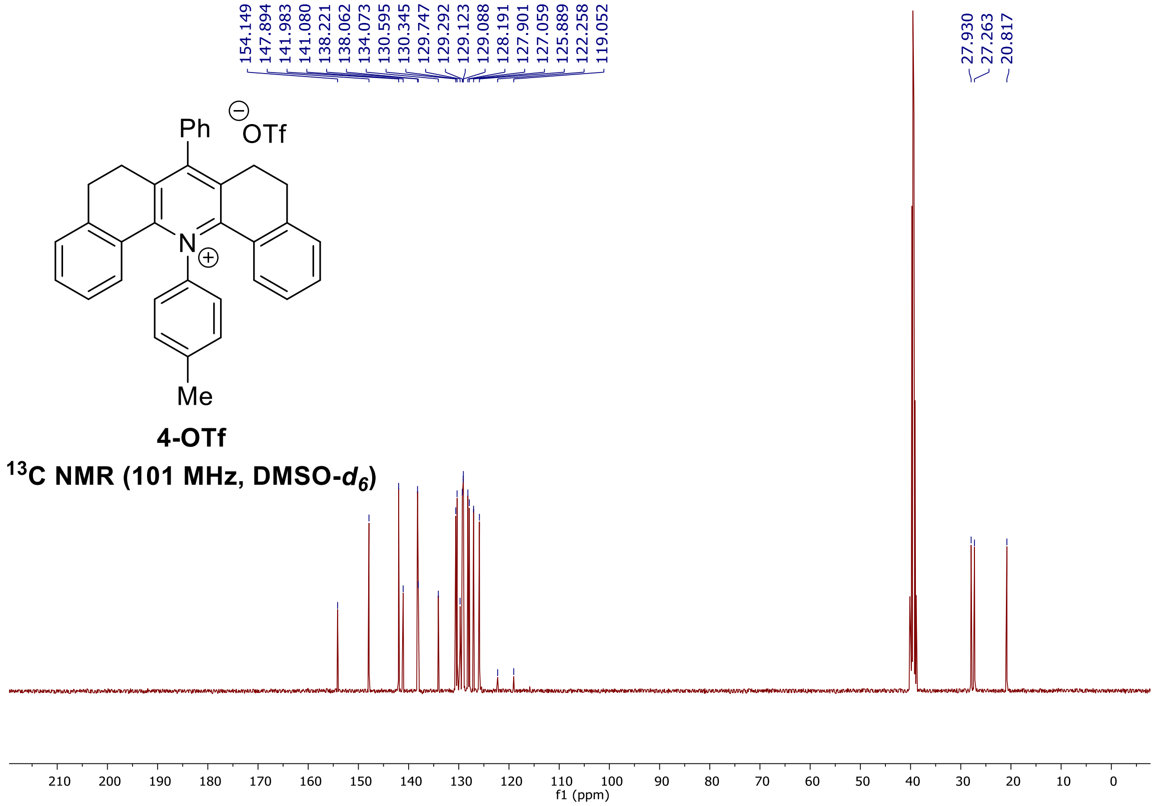

4-OTf

^{13}C NMR (101 MHz, DMSO-d_6)

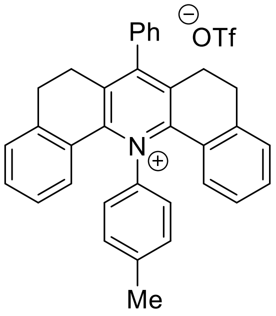

4-OTf

^{19}F NMR (470 MHz, DMSO-d_6)

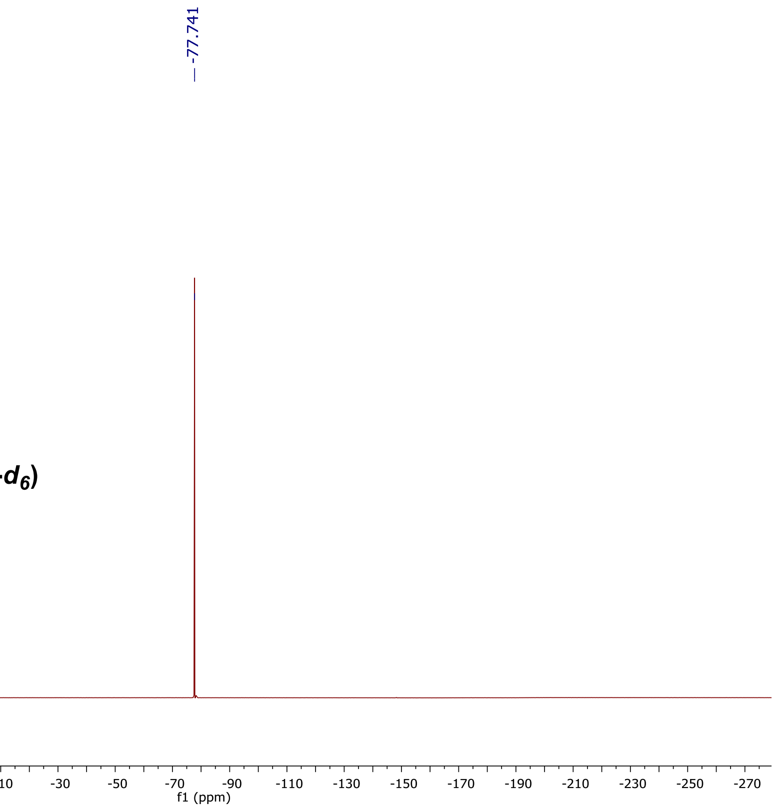

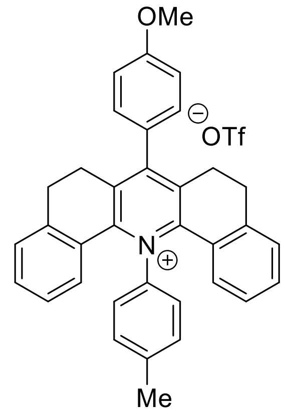

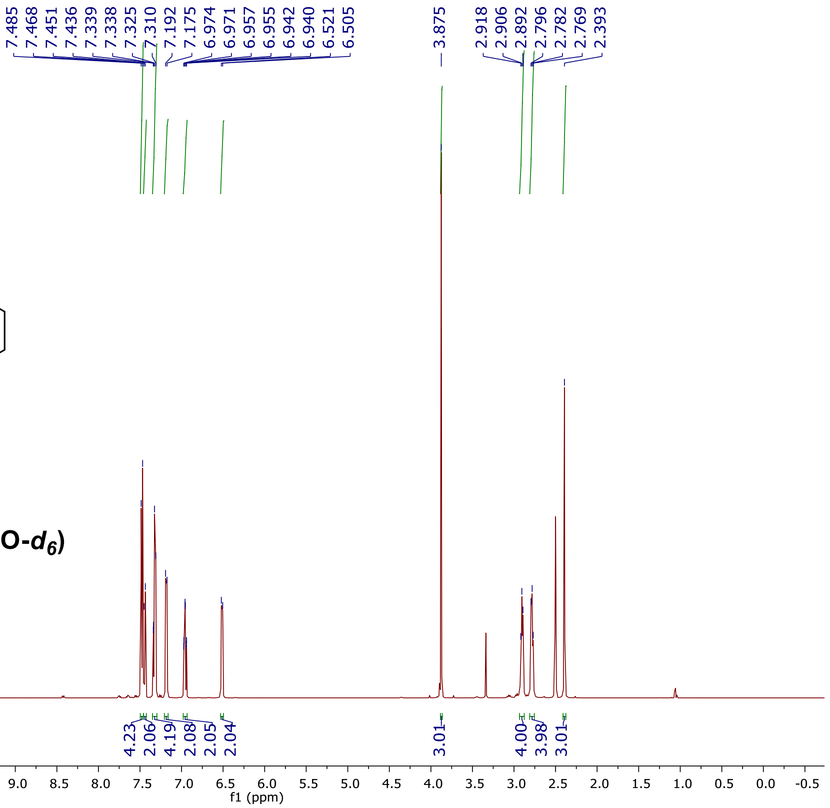

S8

^{1}H NMR (500 MHz, DMSO-*d*$_6$)

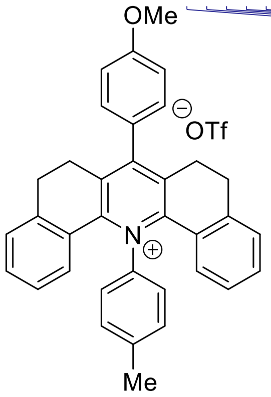

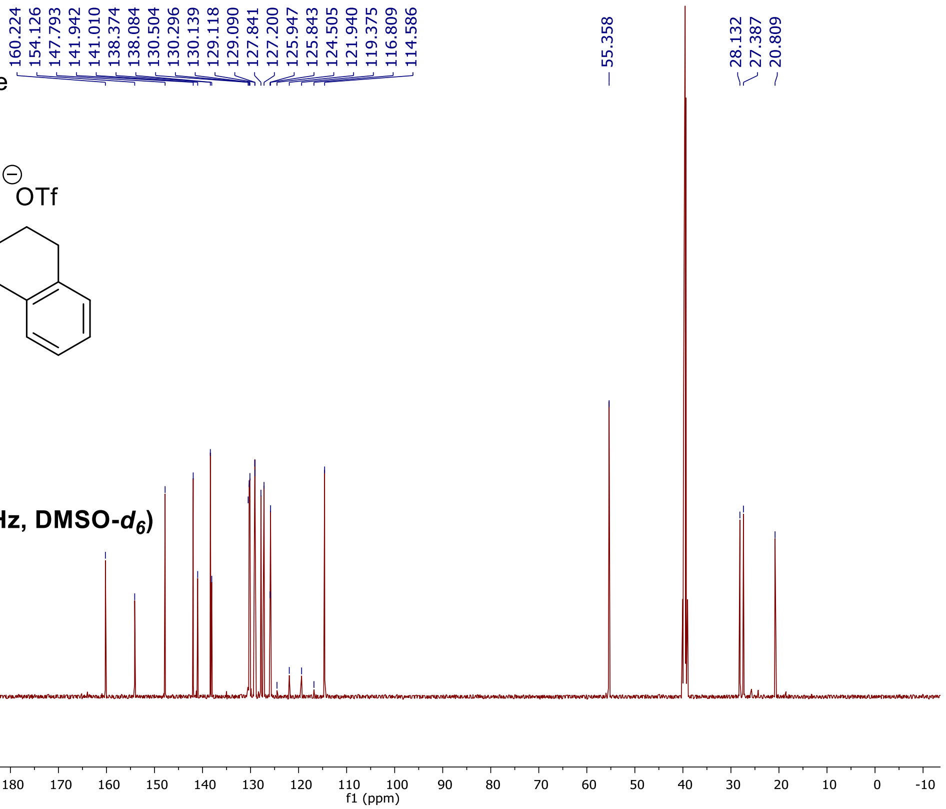

S8

^{13}C NMR (126 MHz, DMSO-*d*$_6$)

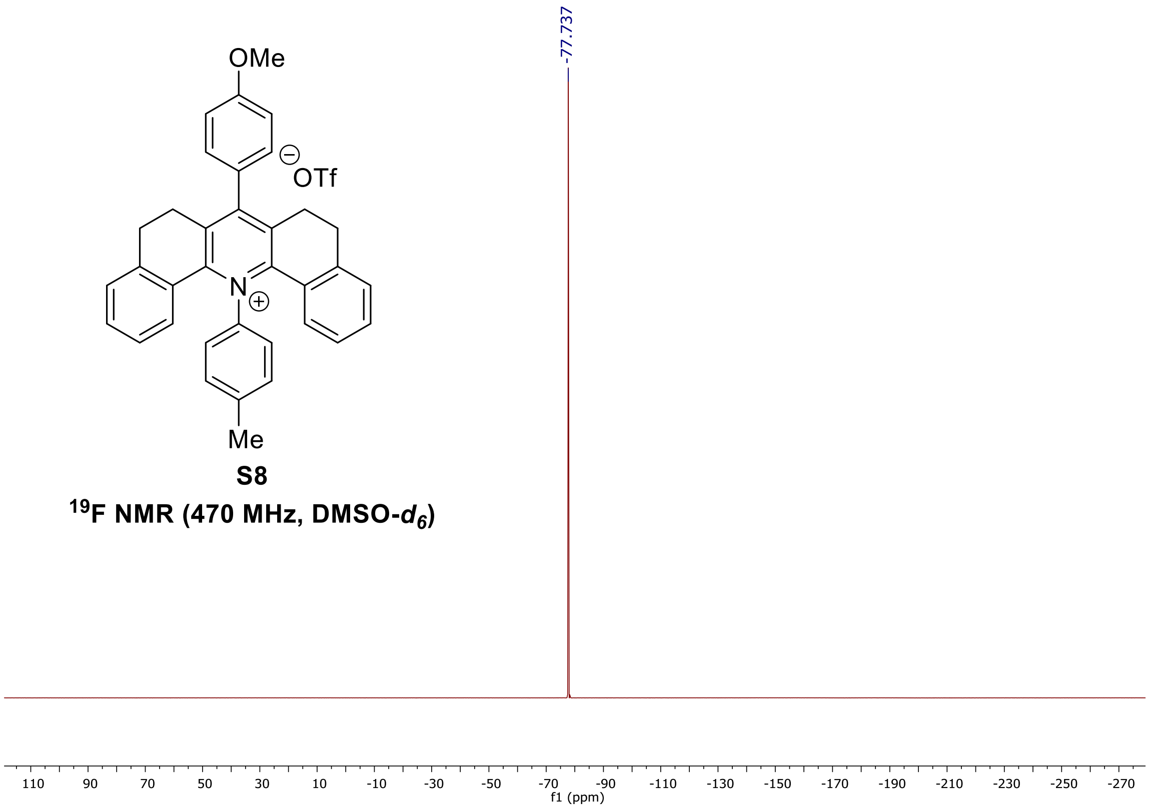

S8

^{19}F NMR (470 MHz, DMSO-d_6)

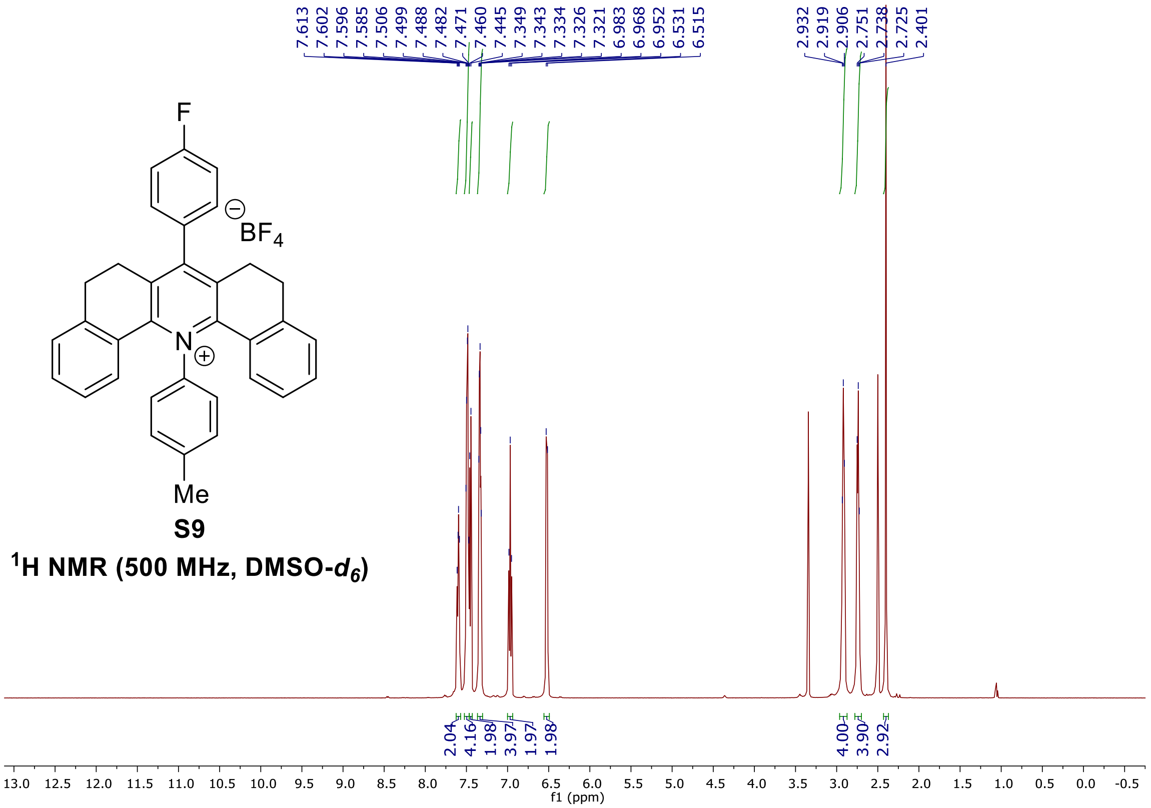

S9

^{1}H NMR (500 MHz, DMSO-d_6)

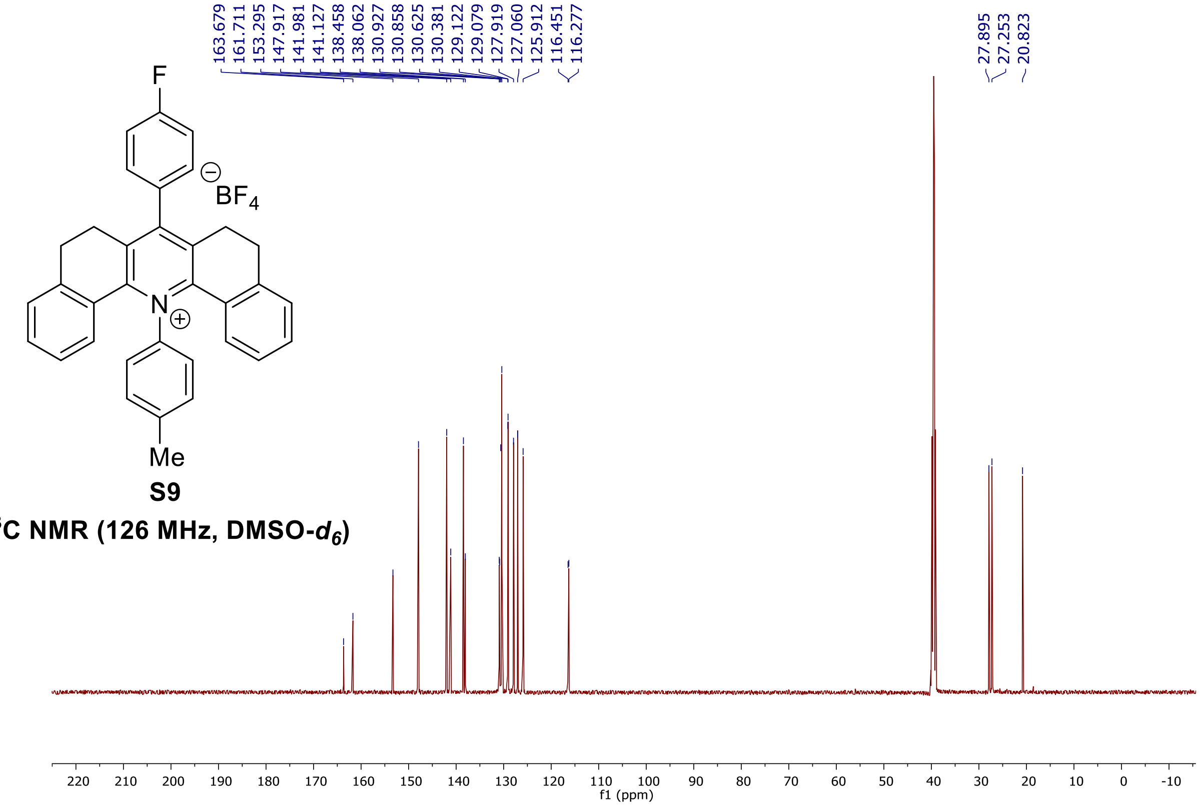

S9

^{13}C NMR (126 MHz, DMSO-d_6)

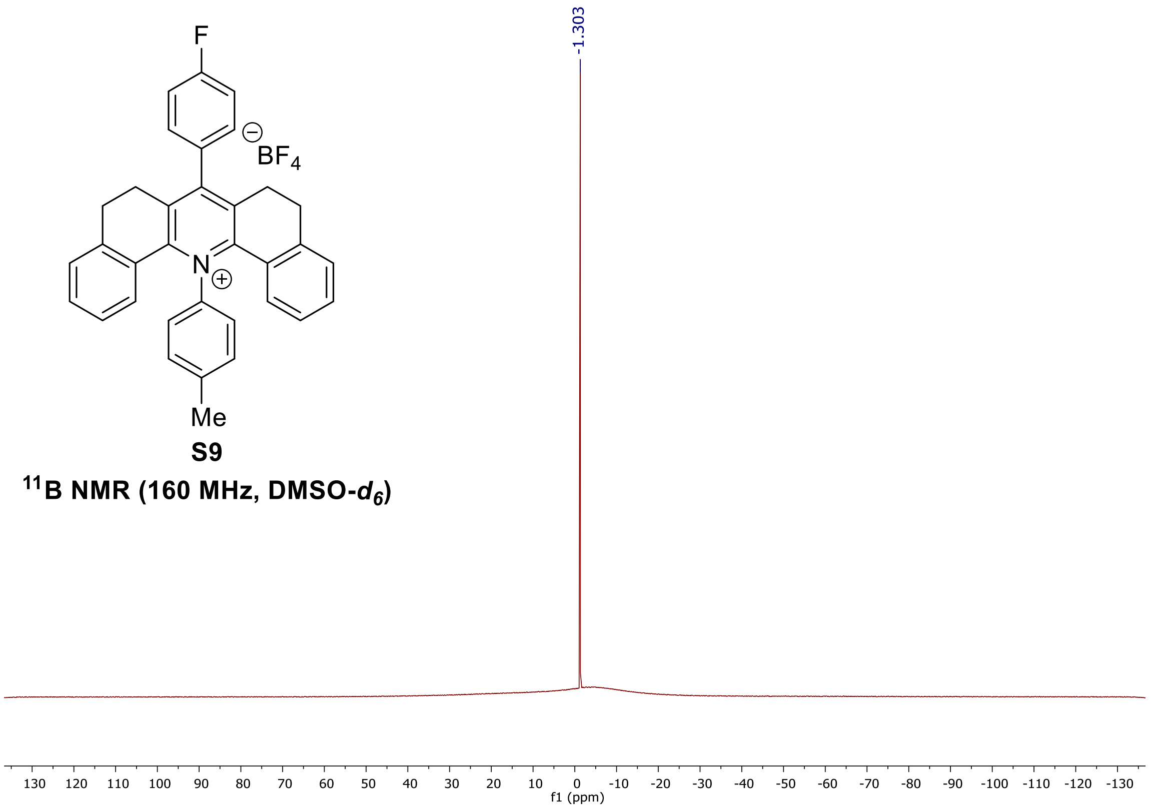

S9

^{11}B NMR (160 MHz, DMSO-d_6)

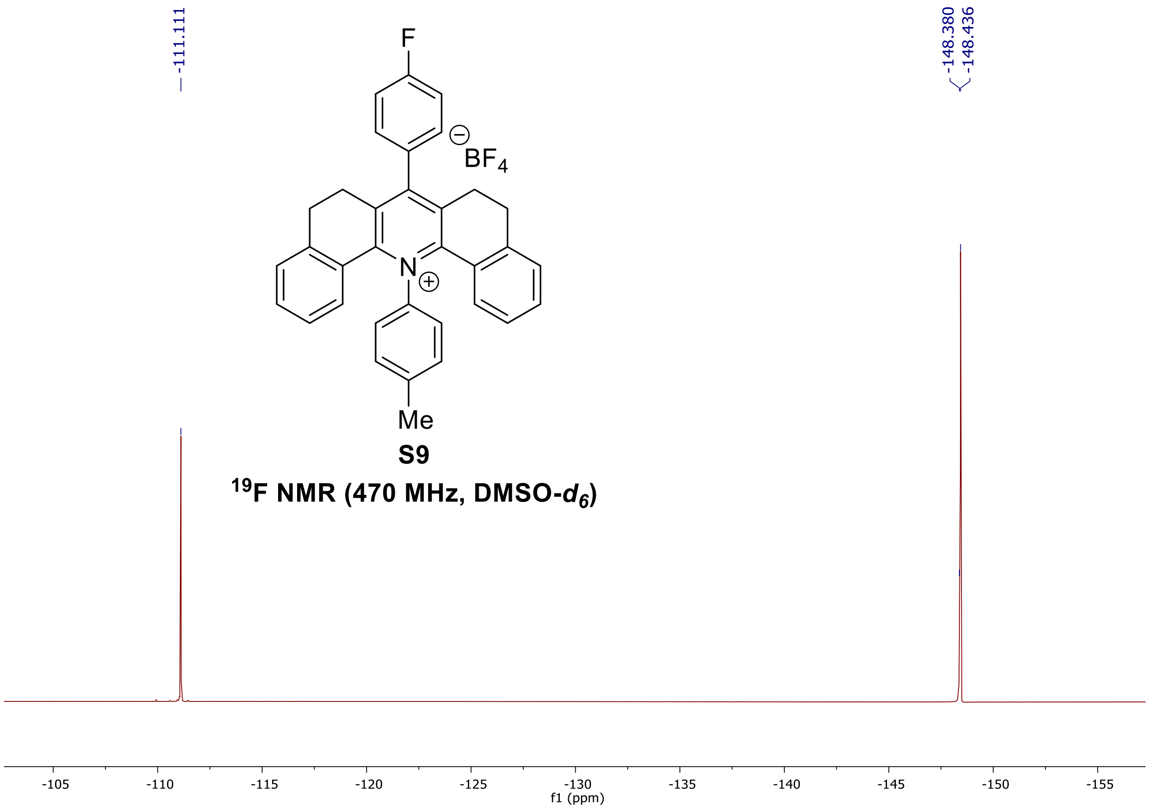

S9

^{19}F NMR (470 MHz, DMSO-d_6)

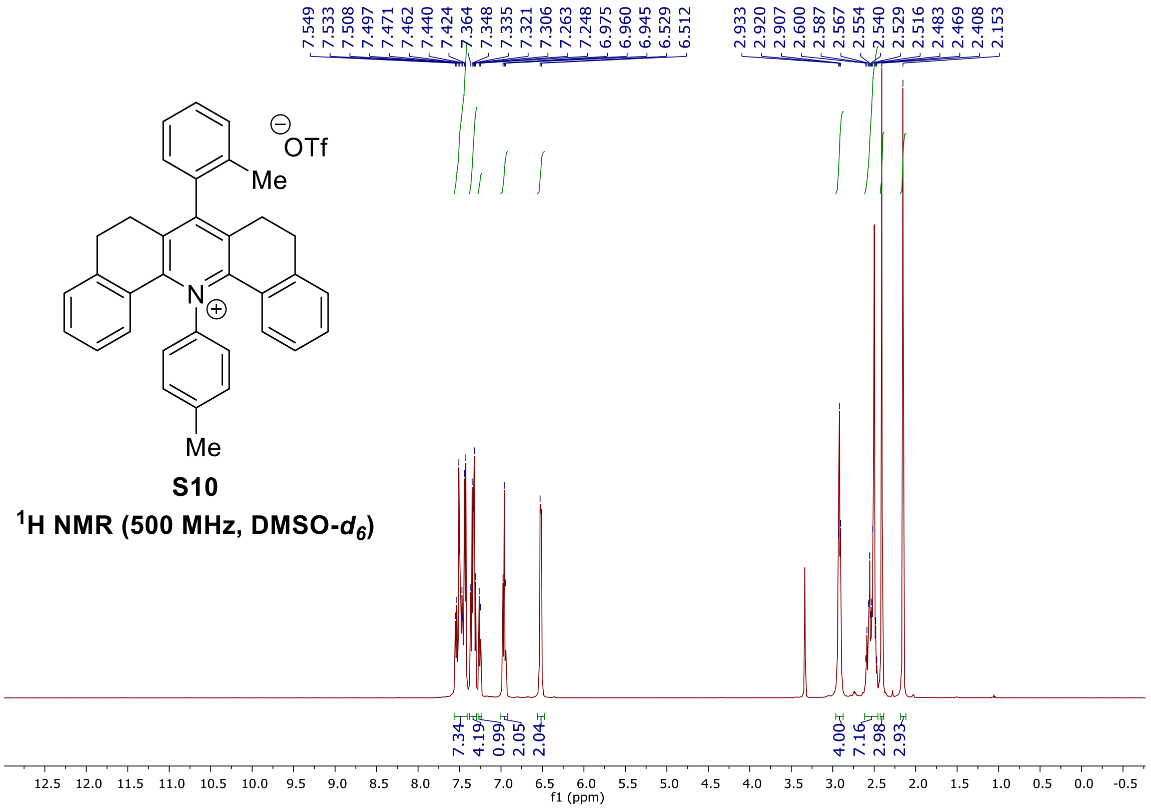

S10

^{1}H NMR (500 MHz, DMSO-*d$_6$*)

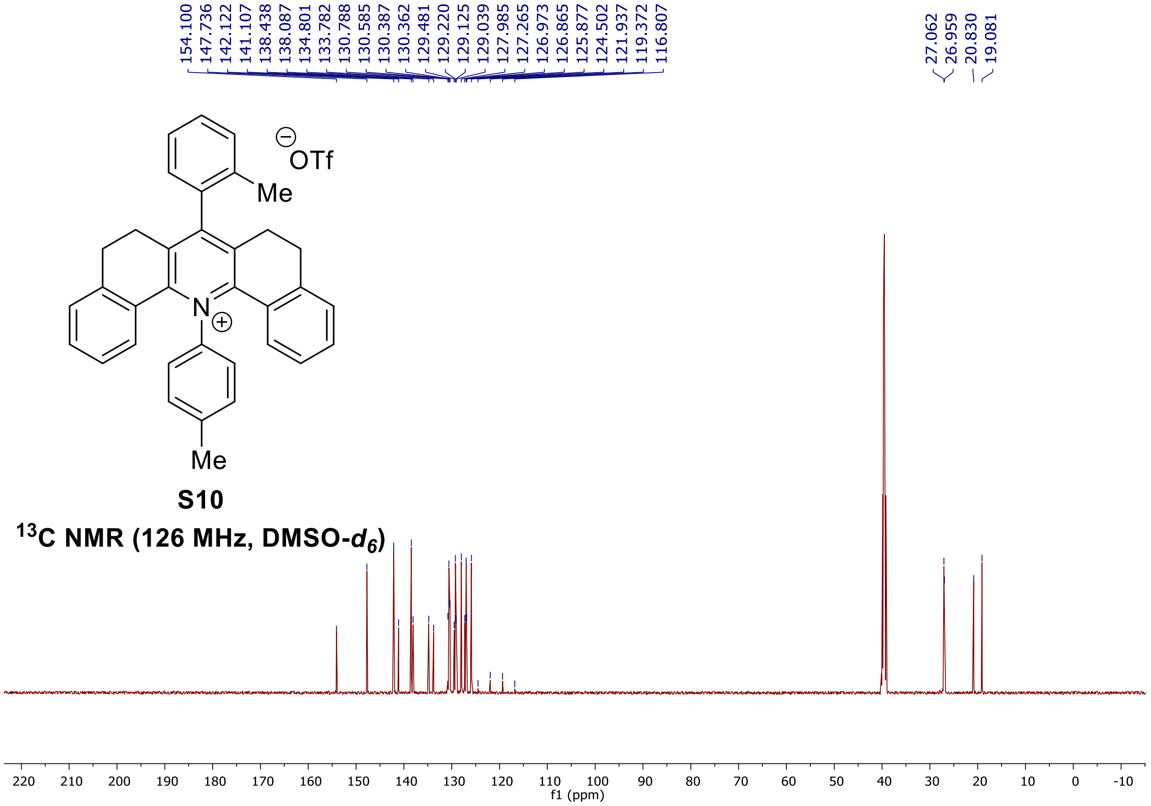

S10

^{13}C NMR (126 MHz, DMSO-*d$_6$*)

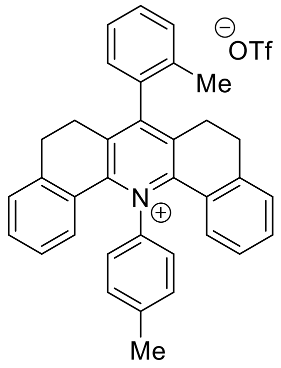

S10

^{19}F NMR (470 MHz, DMSO-*d$_6$*)

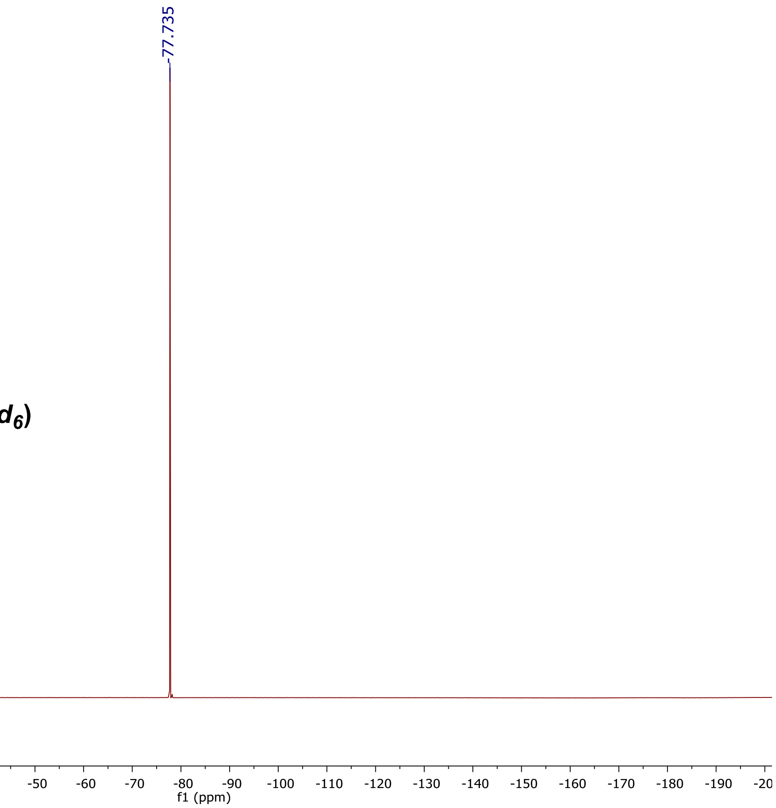

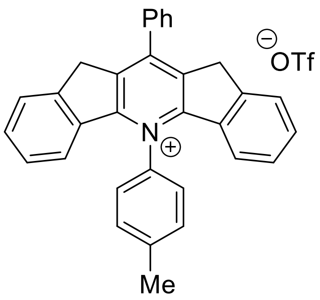

S11

^{1}H NMR (500 MHz, $CDCl_3$)

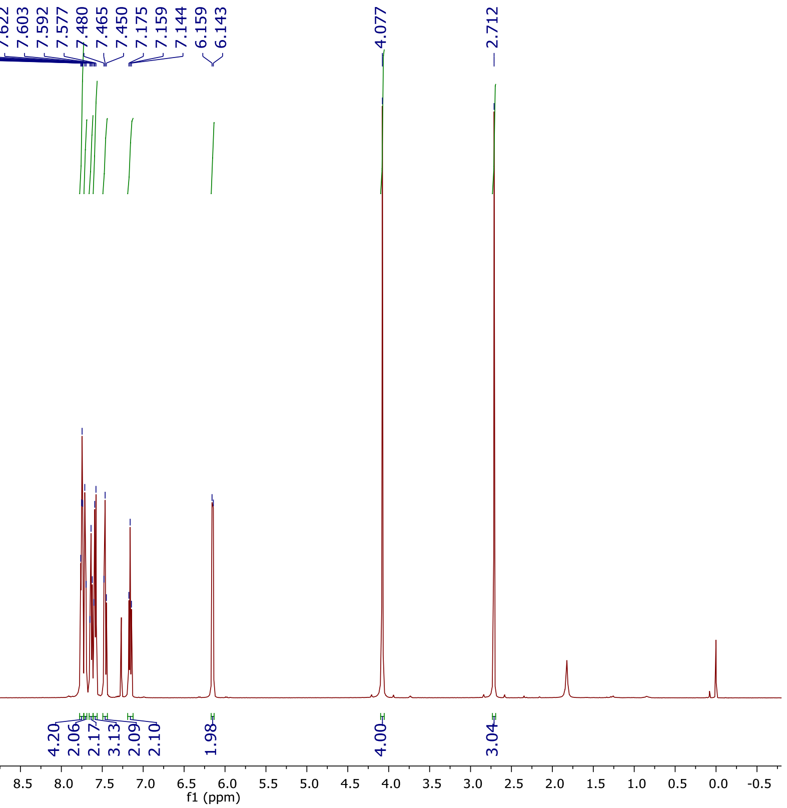

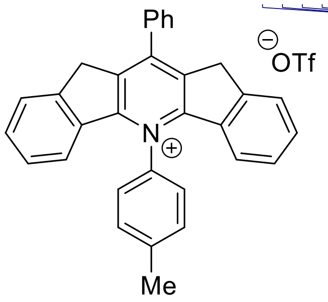

S11

^{13}C NMR (126 MHz, $CDCl_3$)

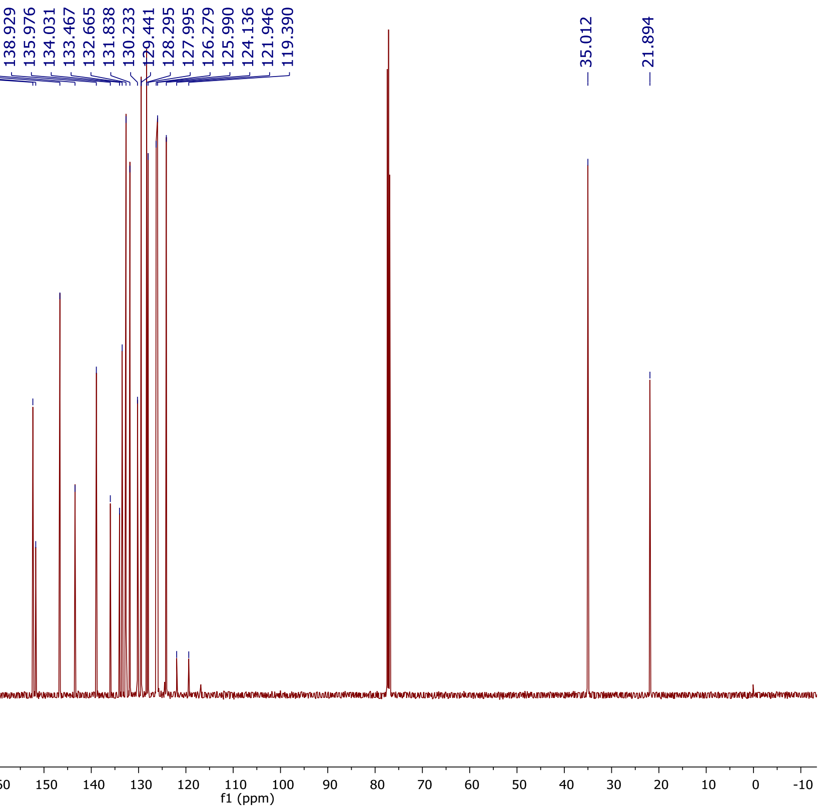

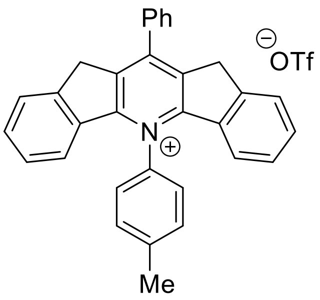

S11

^{19}F NMR (470 MHz, CDCl$_3$)

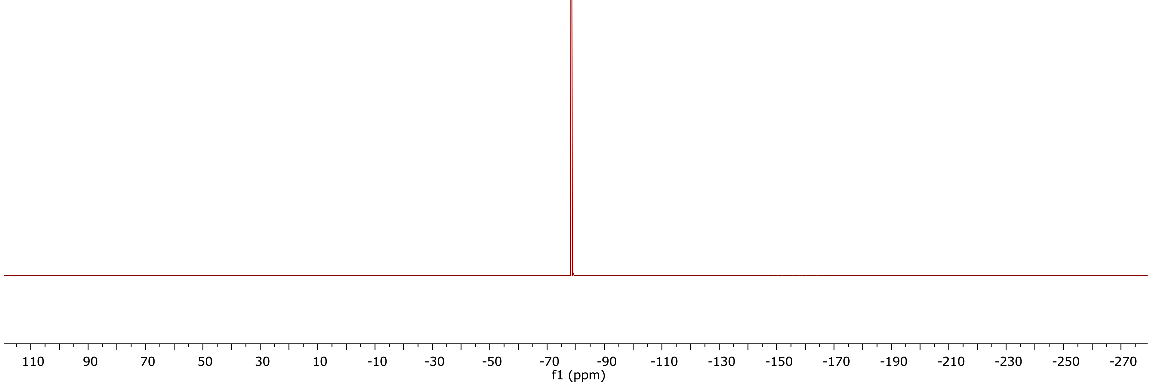

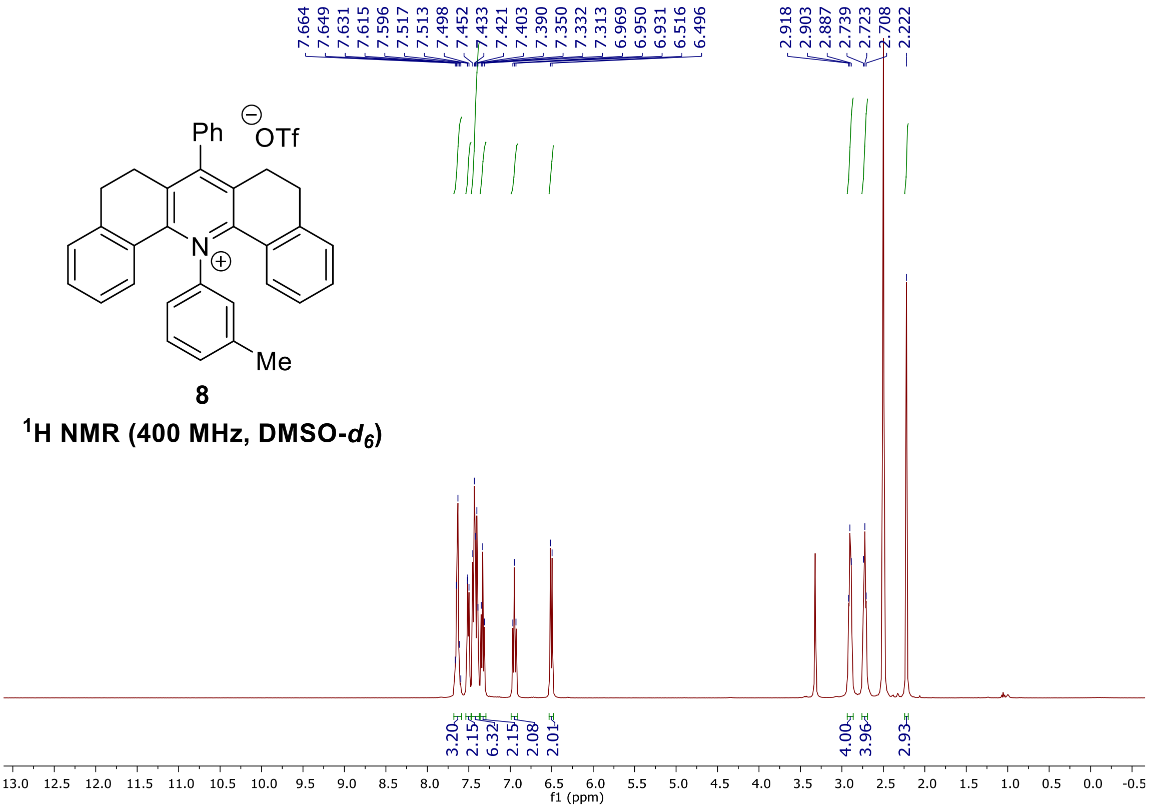

8

^{1}H NMR (400 MHz, DMSO-d_6)

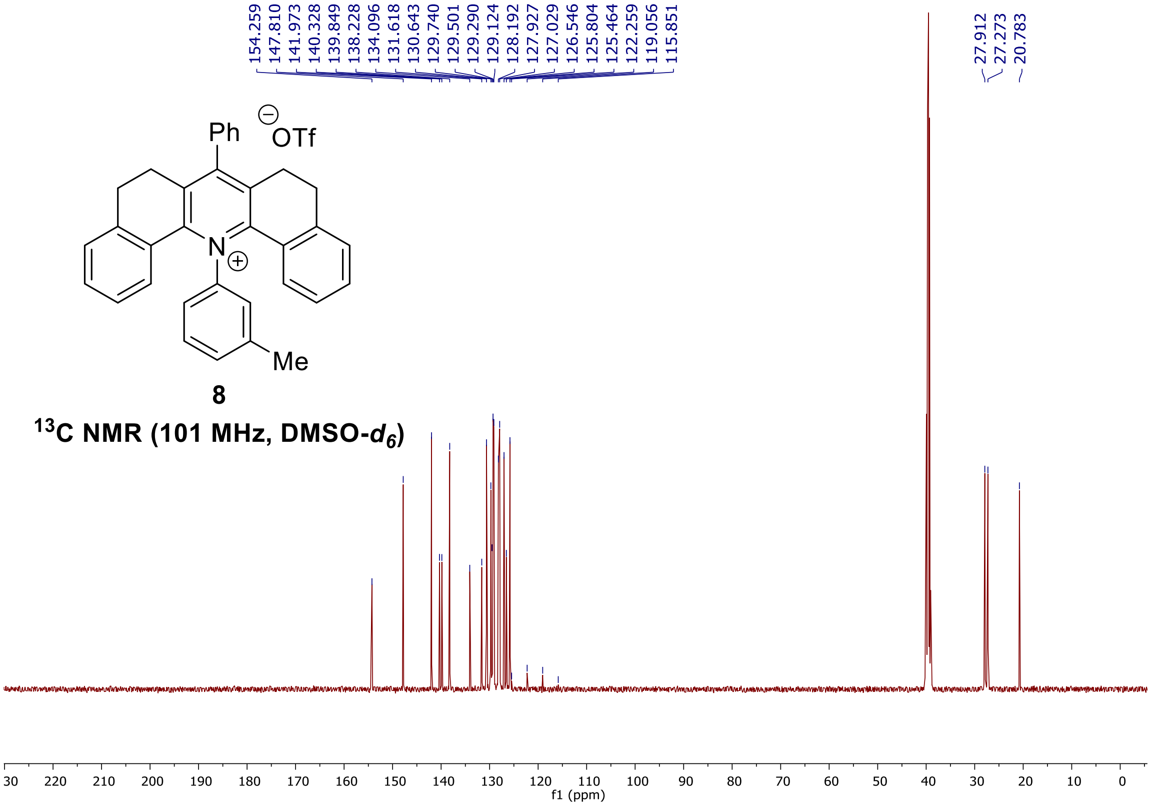

8

^{13}C NMR (101 MHz, DMSO-d_6)

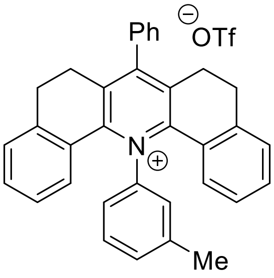

8

^{19}F NMR (470 MHz, DMSO-*d$_6$*)

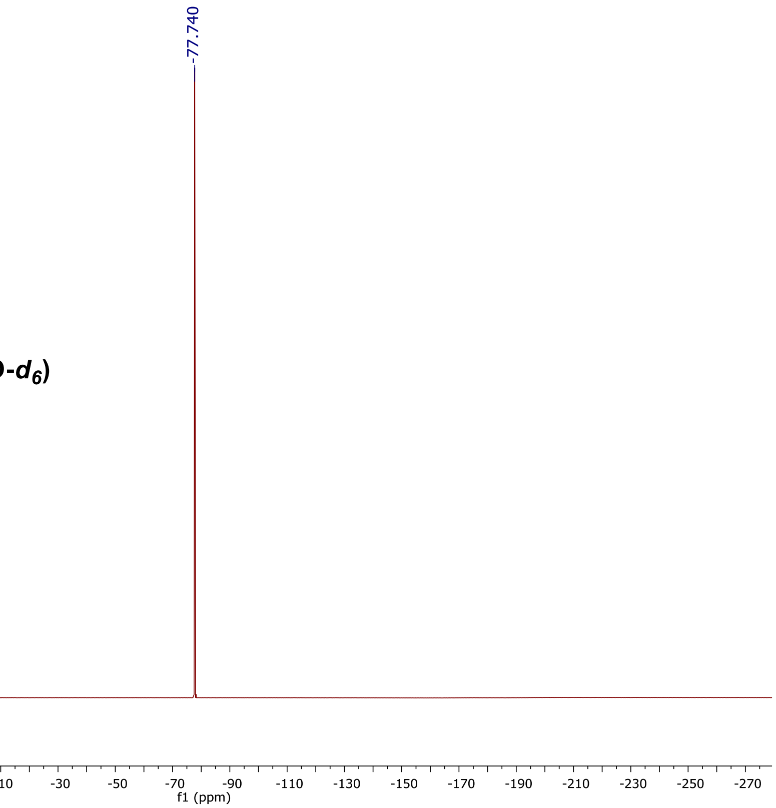

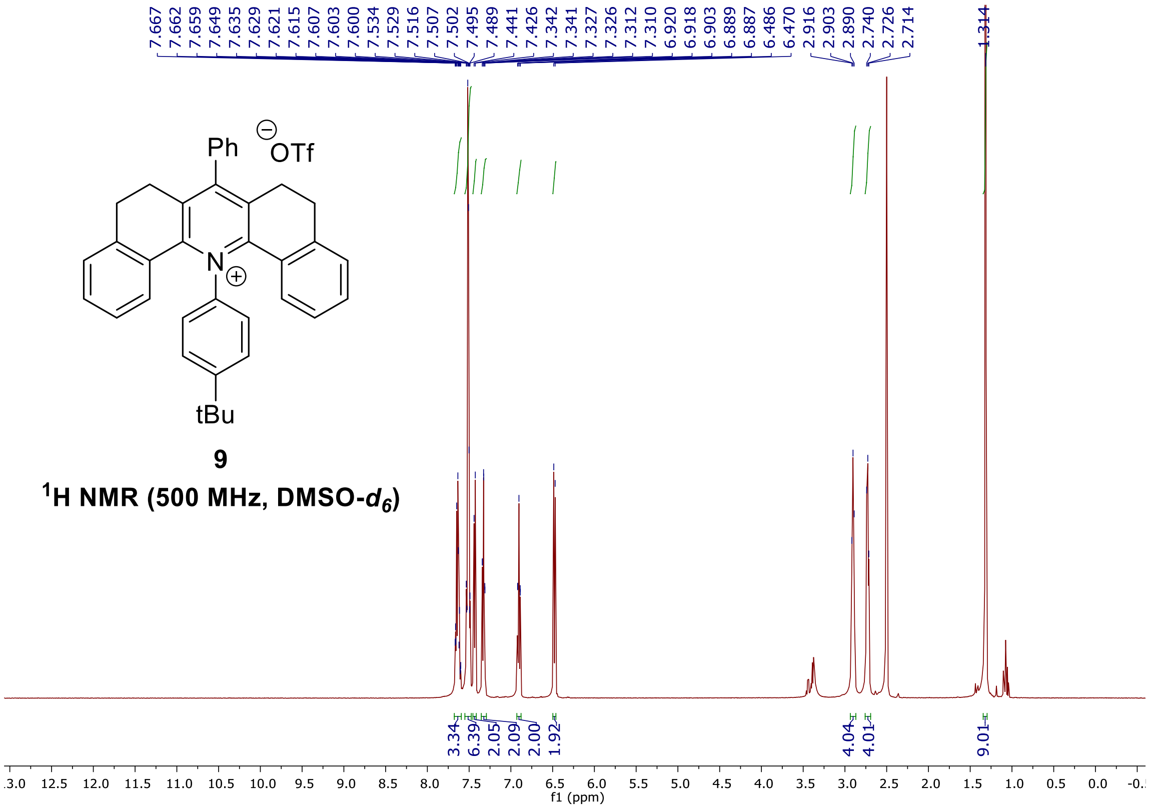
7.667 7.662 7.659 7.649 7.635 7.629 7.621 7.615 7.607 7.603 7.600 7.534 7.529 7.516 7.507 7.502 7.495 7.489 7.441 7.426 7.342 7.341 7.327 7.326 7.312 7.310 6.920 6.918 6.903 6.889 6.887 6.486 6.470 2.916 2.903 2.890 2.740 2.726 2.714 1.314
Ph
OTf
N
tBu
9
¹H NMR (500 MHz, DMSO-d6)
3.34 6.39 2.05 2.09 2.00 1.92 4.04 4.01 9.01
f1 (ppm)

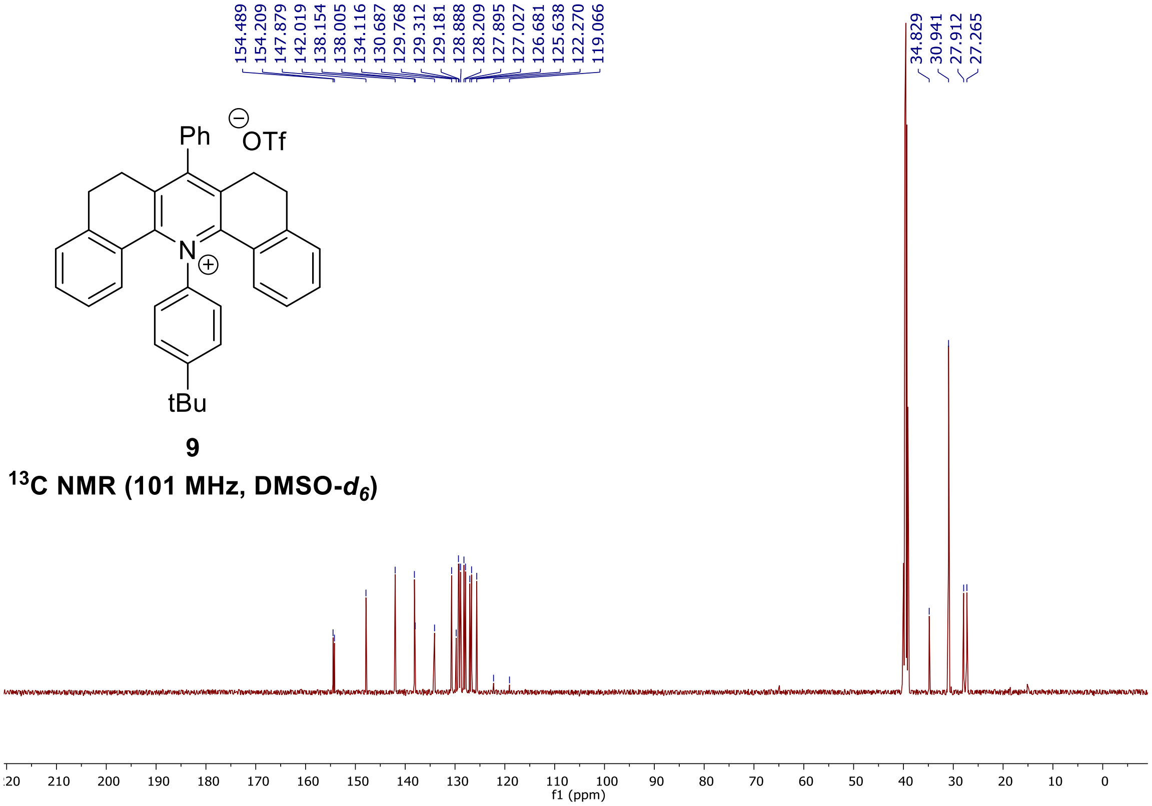
154.489 154.209 147.879 142.019 138.154 138.005 134.116 130.687 129.768 129.312 129.181 128.888 128.209 127.895 127.027 126.681 125.638 122.270 119.066
34.829 30.941 27.912 27.265
Ph
OTf
N
tBu
9
¹³C NMR (101 MHz, DMSO-d6)
f1 (ppm)

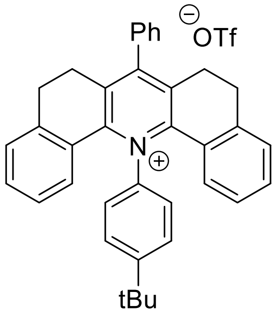

9

^{19}F NMR (470 MHz, DMSO-d_6)

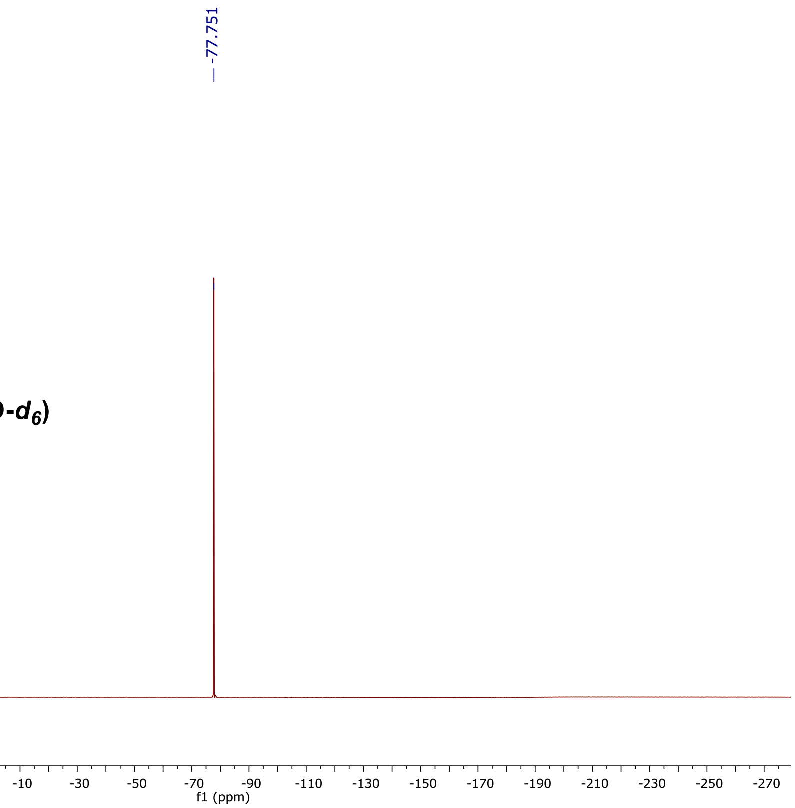

10

^{1}H NMR (400 MHz, DMSO-d_6)

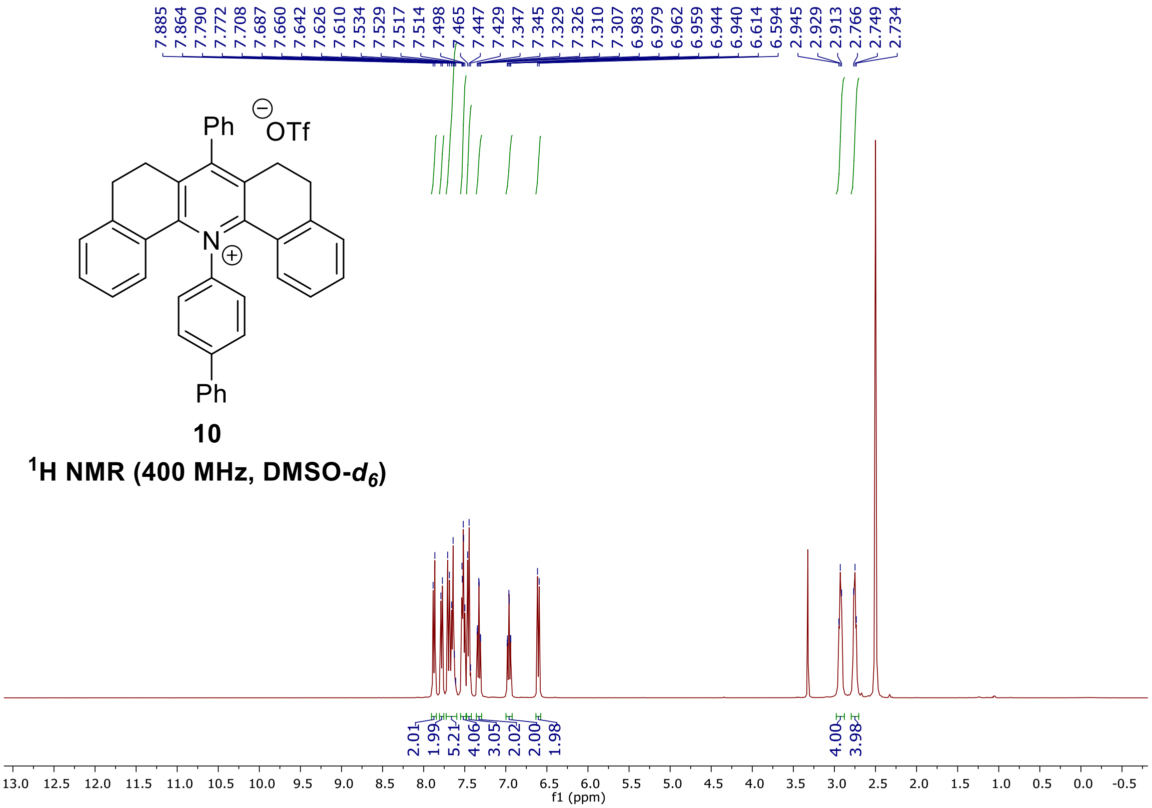

10

^{13}C NMR (101 MHz, DMSO-d_6)

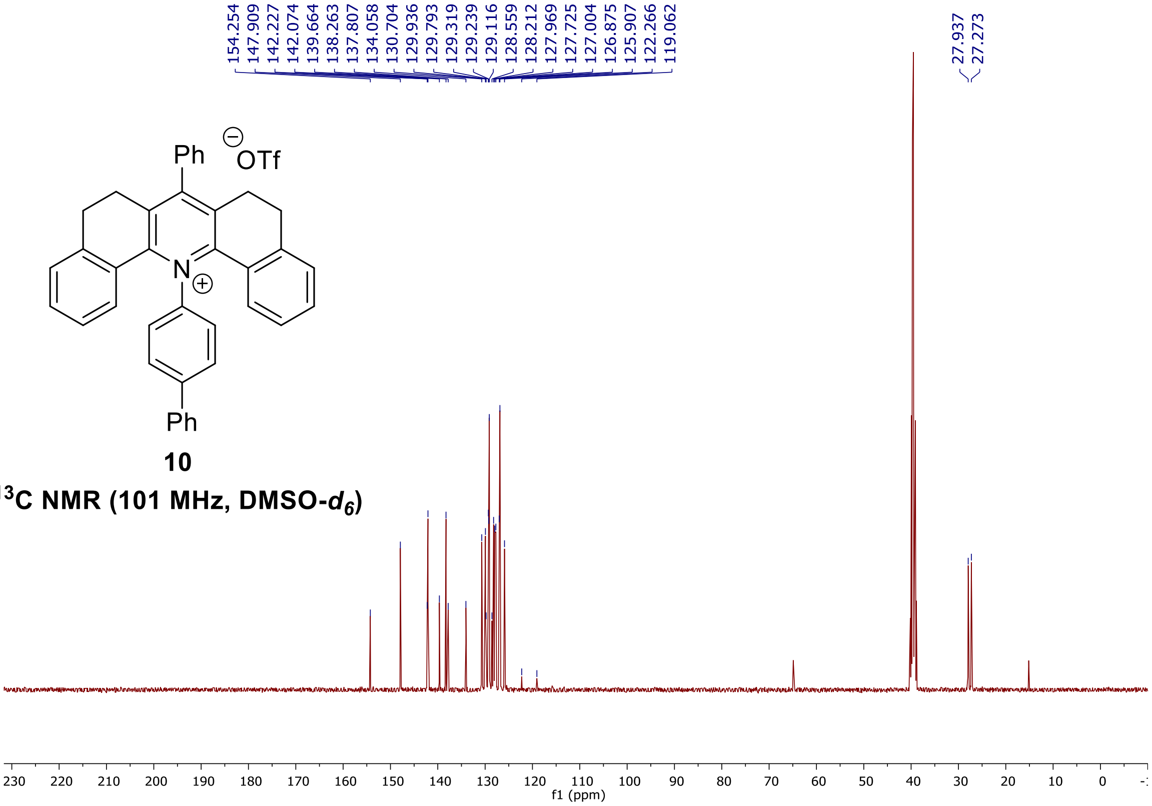

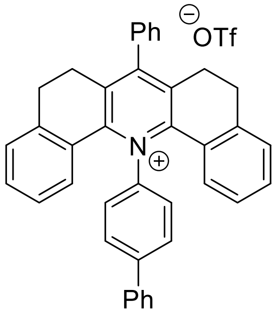

10

^{19}F NMR (470 MHz, DMSO-d_6)

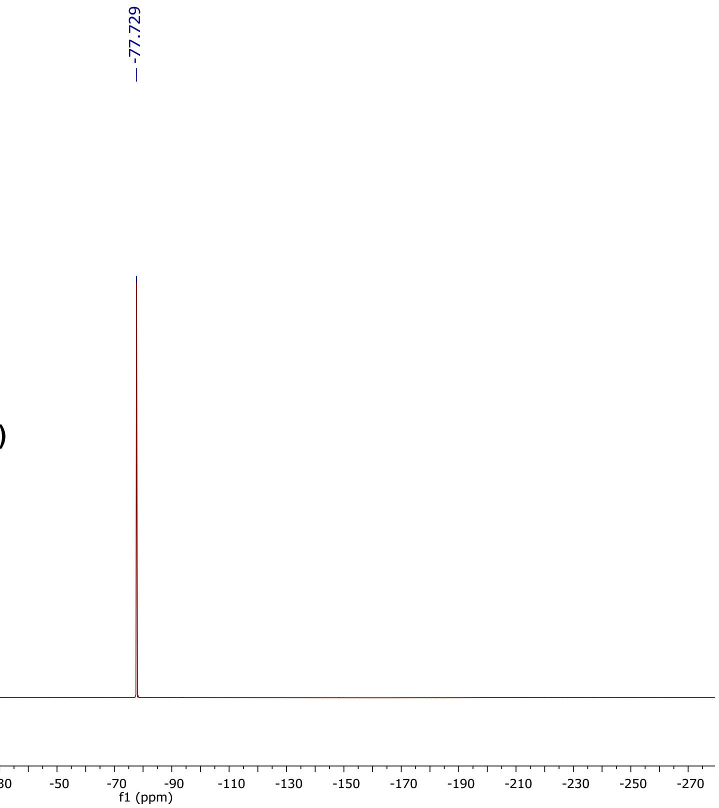

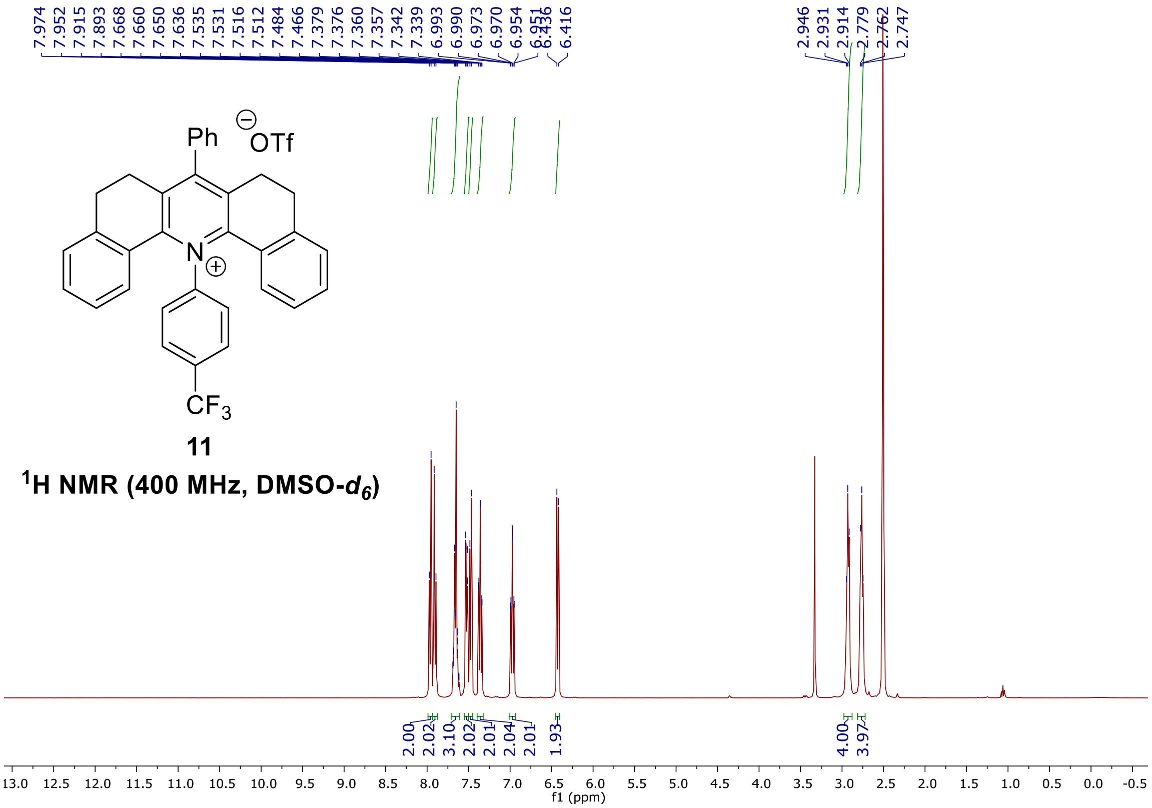
7.974 7.952 7.915 7.893 7.668 7.660 7.650 7.636 7.535 7.531 7.516 7.512 7.484 7.466 7.379 7.376 7.360 7.357 7.342 7.339 6.993 6.990 6.973 6.970 6.954 6.951 6.436 6.416
2.946 2.931 2.914 2.779 2.762 2.747
Ph
OTf
N
CF3
11
1H NMR (400 MHz, DMSO-d6)
2.00 2.02 3.10 2.02 2.01 2.04 2.01 1.93
4.00 3.97
13.0 12.5 12.0 11.5 11.0 10.5 10.0 9.5 9.0 8.5 8.0 7.5 7.0 6.5 6.0 5.5 5.0 4.5 4.0 3.5 3.0 2.5 2.0 1.5 1.0 0.5 0.0 -0.5
f1 (ppm)

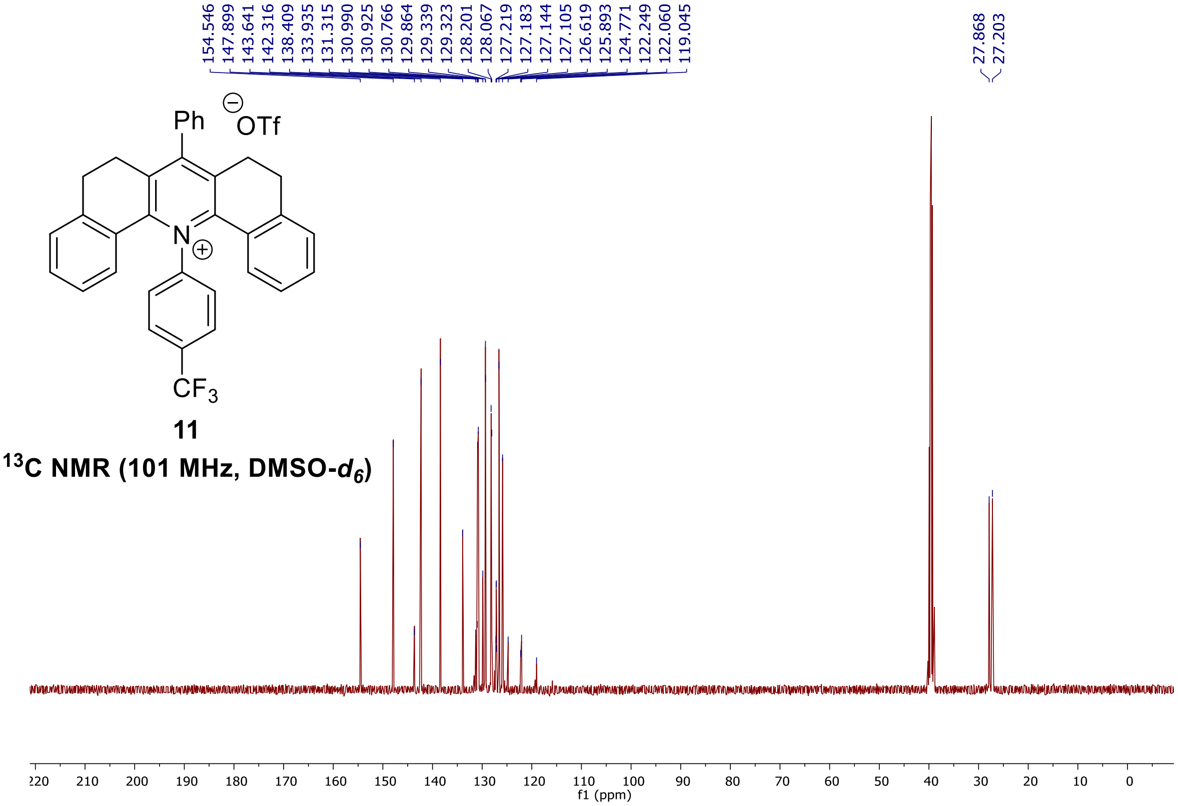
154.546 147.899 143.641 142.316 138.409 133.935 131.315 130.990 130.925 130.766 129.864 129.339 129.323 128.201 128.067 127.219 127.183 127.144 127.105 126.619 125.893 124.771 122.249 122.060 119.045
27.868 27.203
Ph
OTf
N
CF3
11
13C NMR (101 MHz, DMSO-d6)
220 210 200 190 180 170 160 150 140 130 120 110 100 90 80 70 60 50 40 30 20 10 0
f1 (ppm)

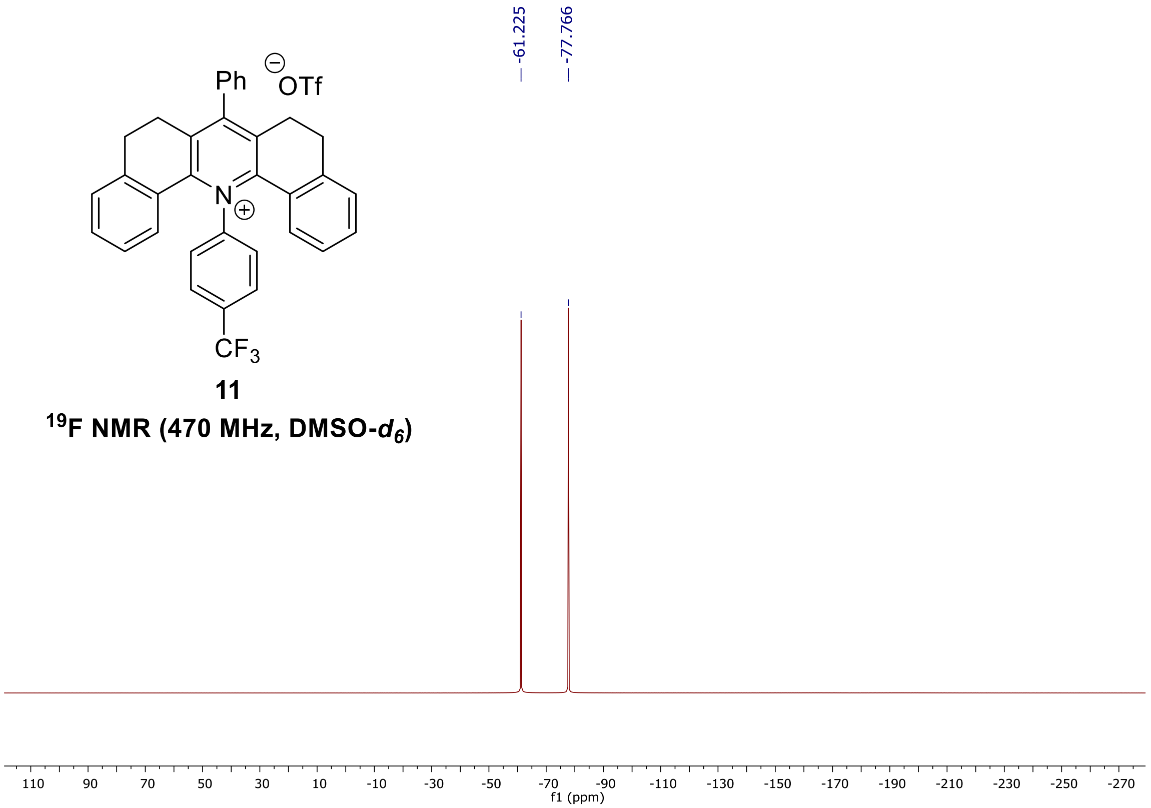

11

^{19}F NMR (470 MHz, DMSO-d_6)

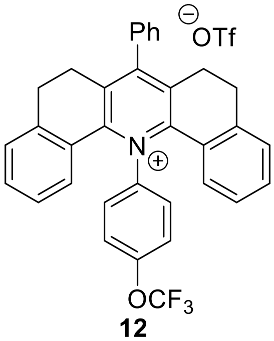

12

^{1}H NMR (400 MHz, DMSO-d_6)

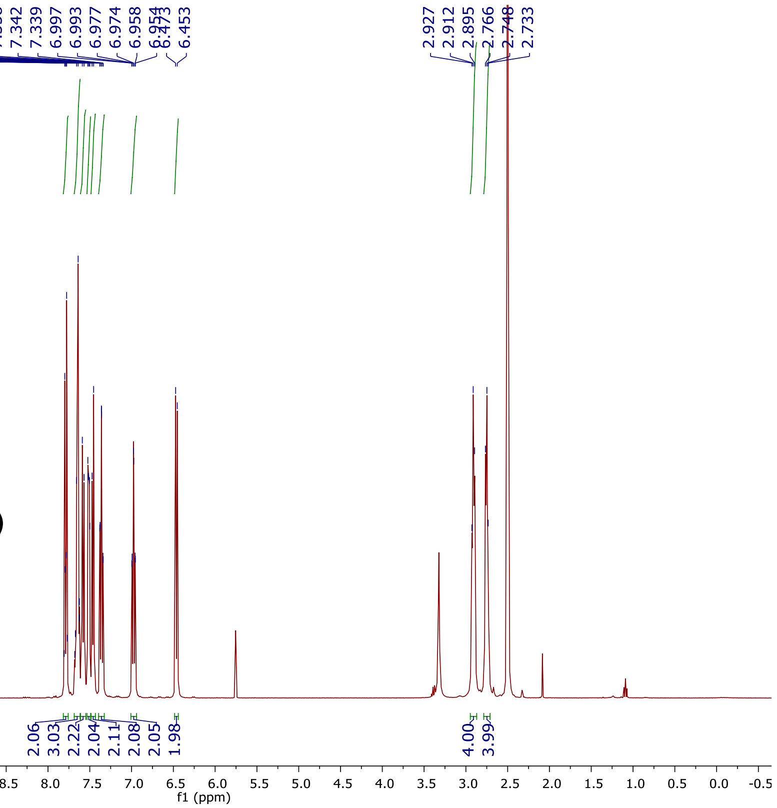

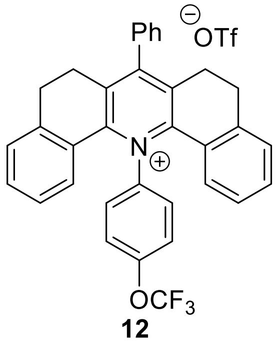

12

^{13}C NMR (101 MHz, DMSO-d_6)

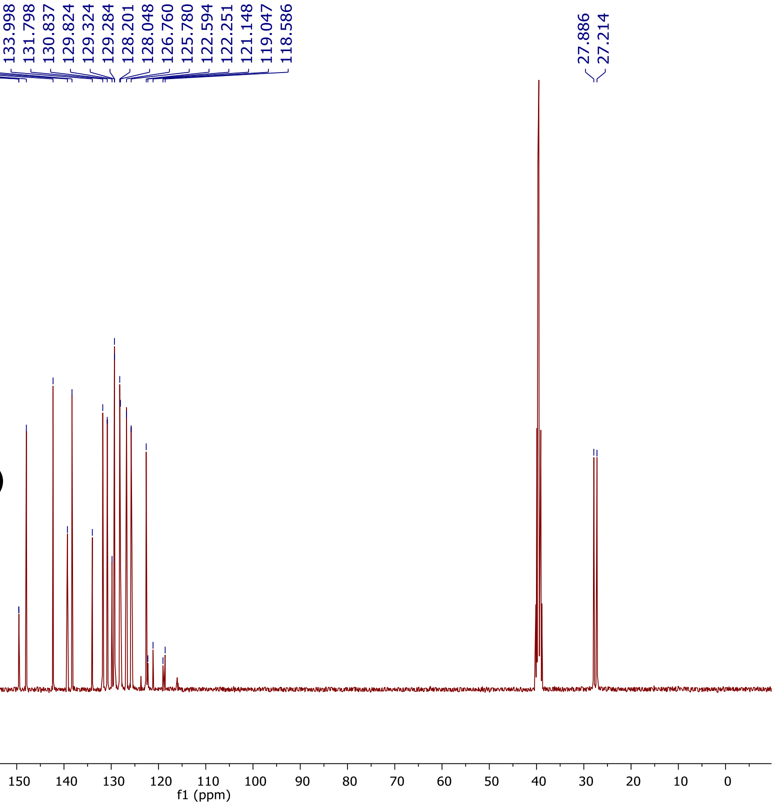

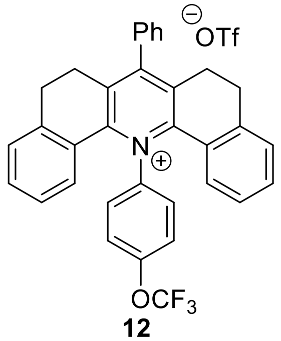

12

^{19}F NMR (470 MHz, DMSO-d_6)

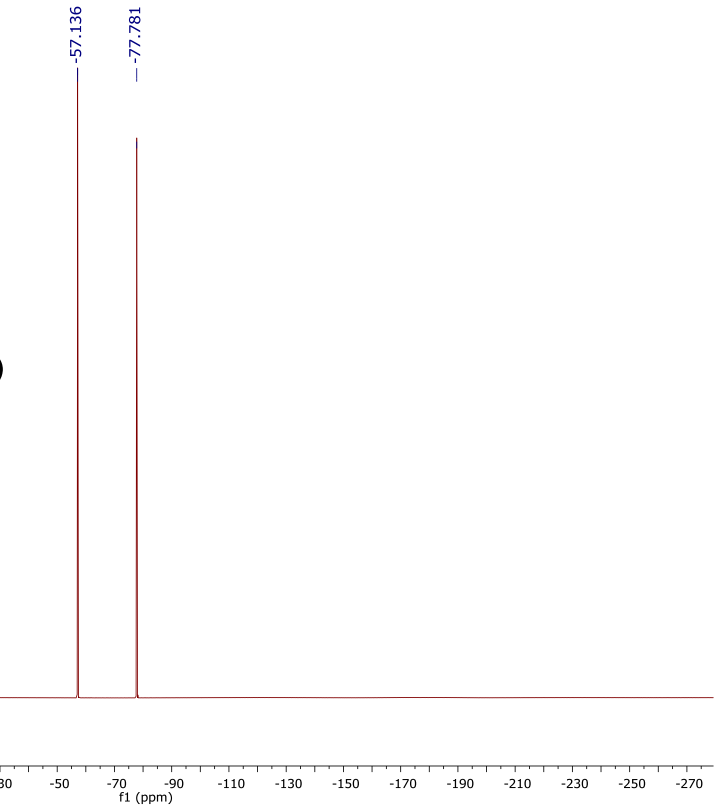

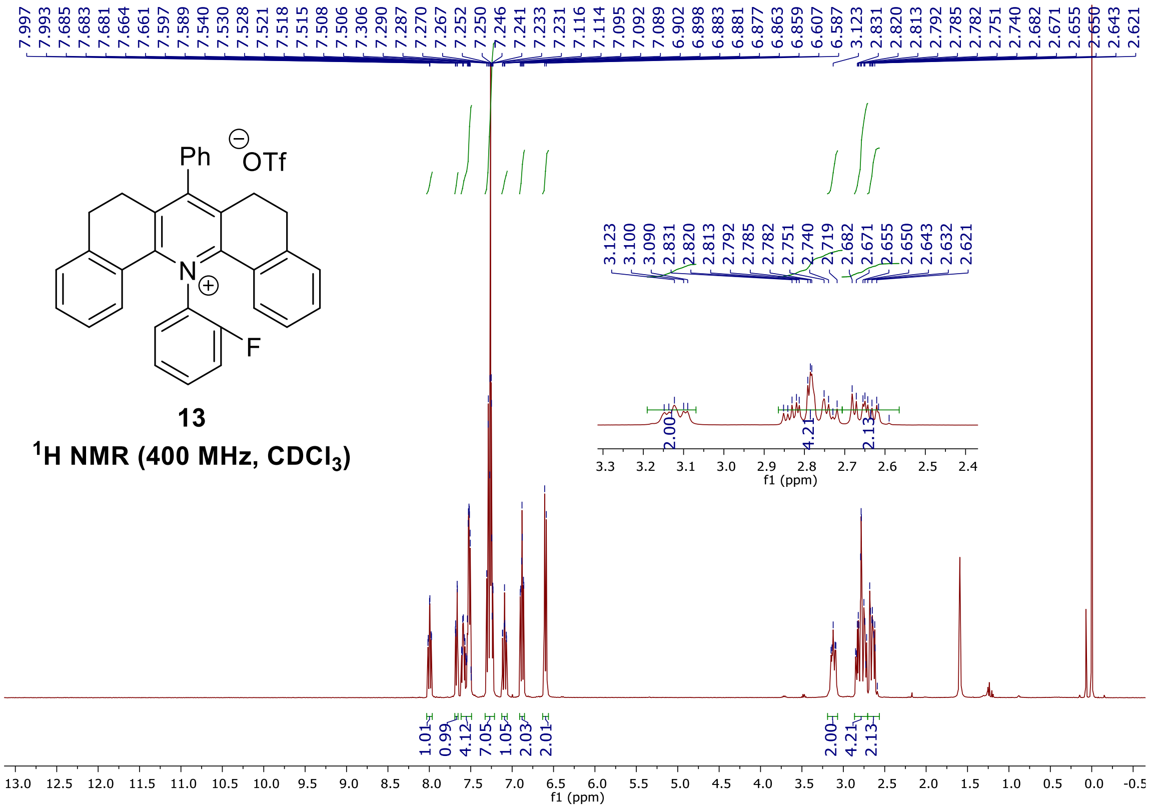
Ph
OTf
N
F
13
1H NMR (400 MHz, CDCl3)
f1 (ppm)

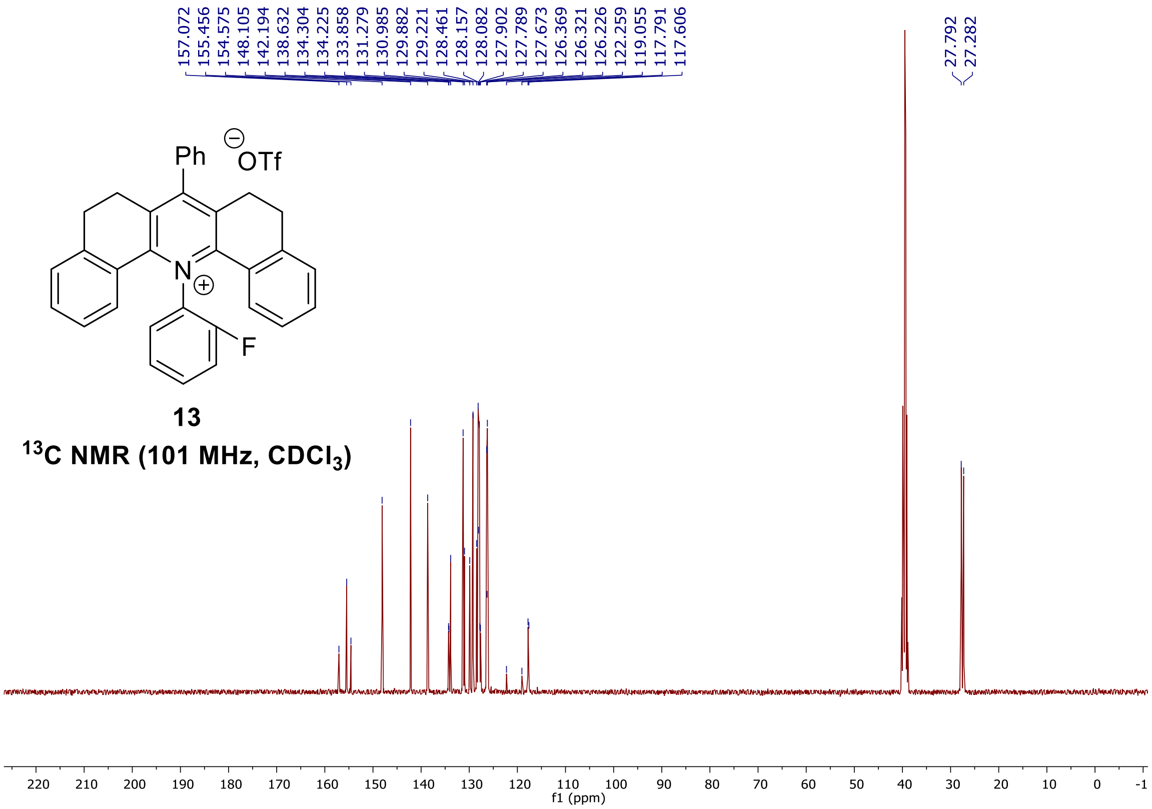
Ph
OTf
N
F
13
13C NMR (101 MHz, CDCl3)
f1 (ppm)

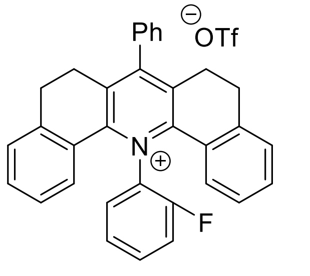

13

^{19}F NMR (470 MHz, $CDCl_3$)

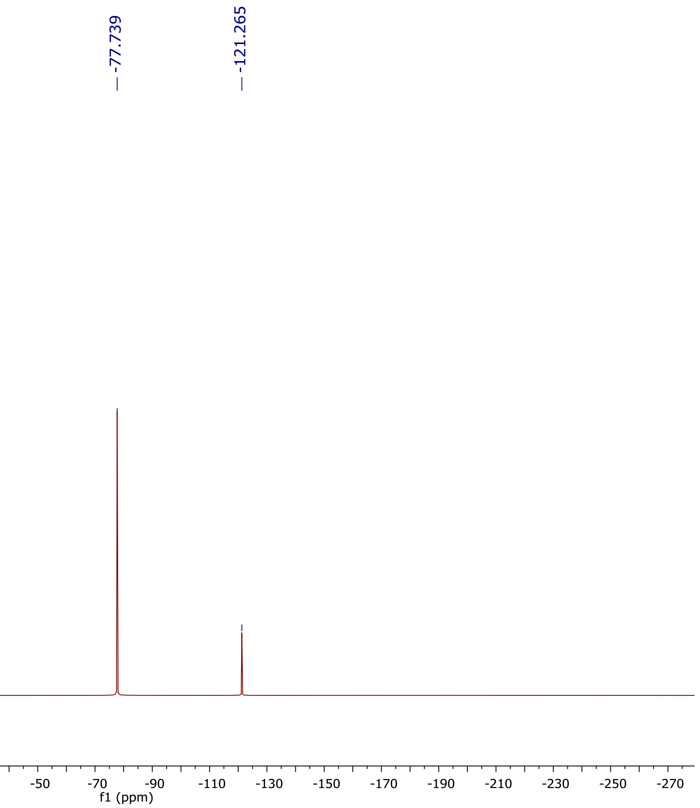

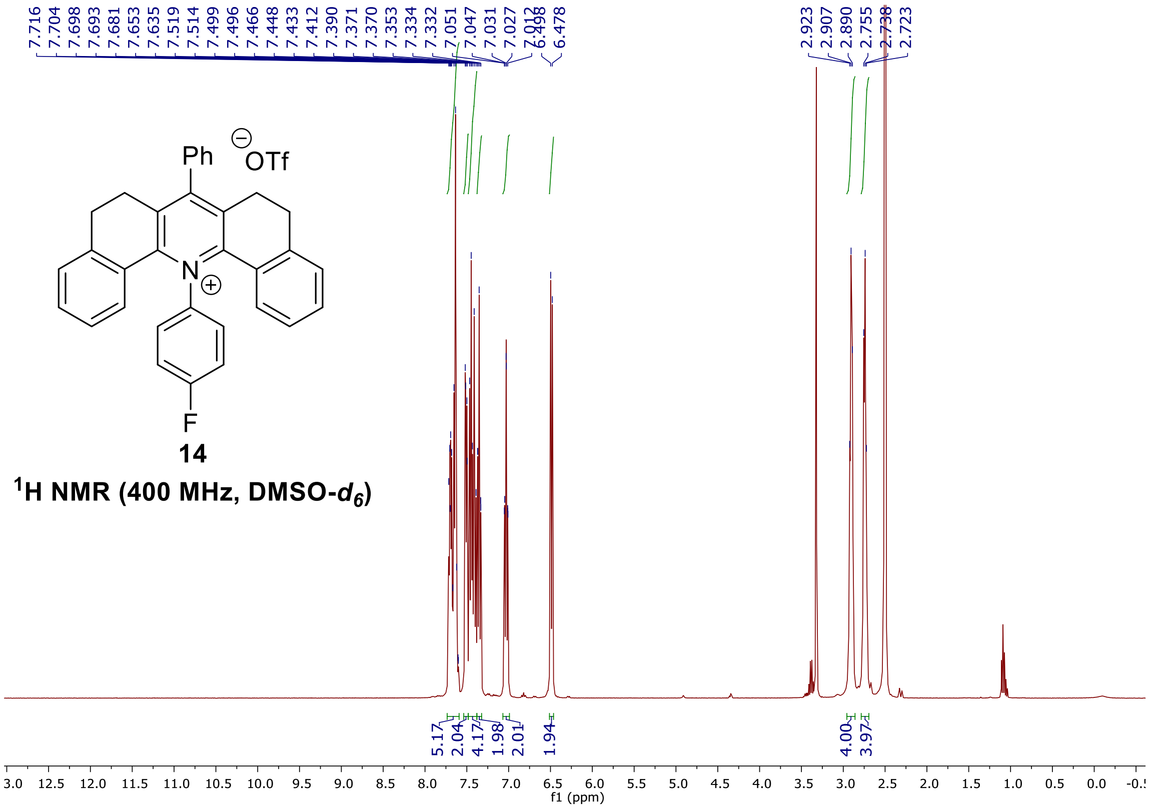

14

^{1}H NMR (400 MHz, DMSO-*d$_6$*)

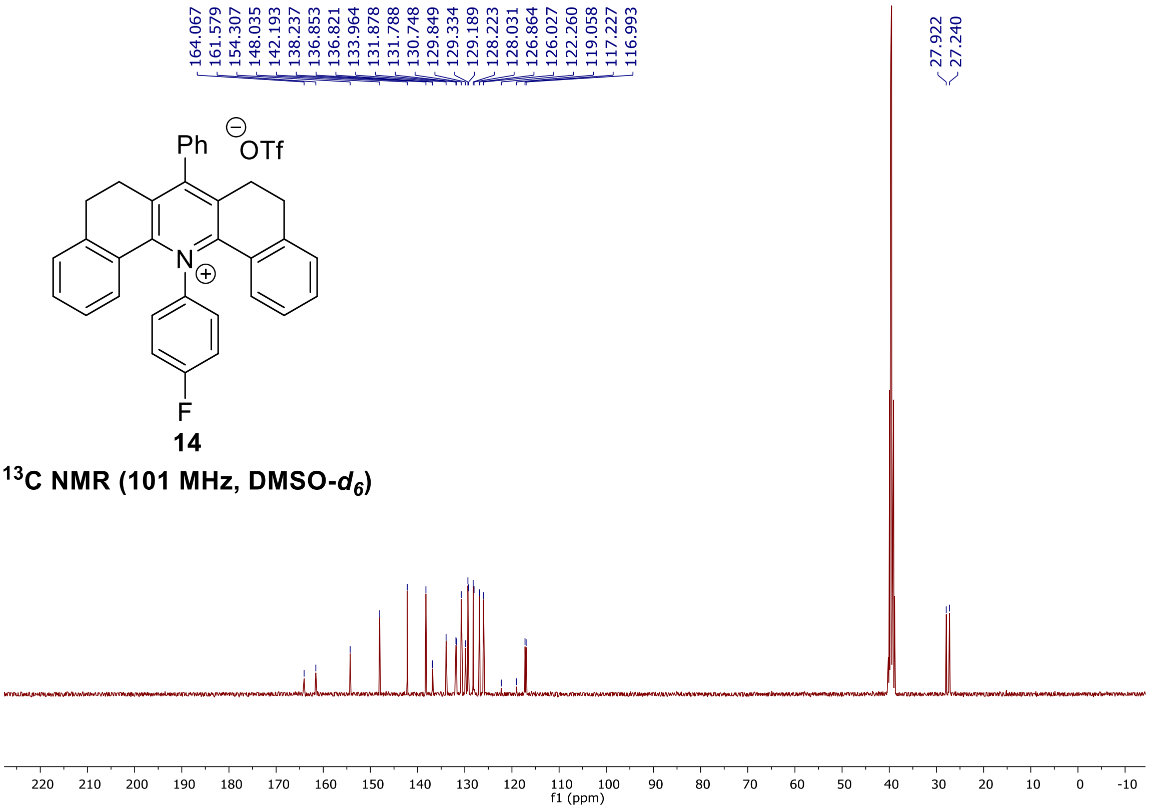

14

^{13}C NMR (101 MHz, DMSO-*d$_6$*)

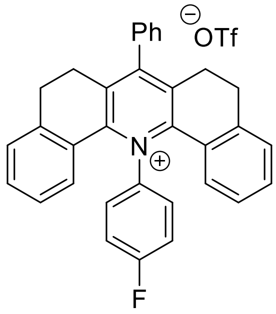

14

^{139}F NMR (470 MHz, DMSO-*d*$_6$)

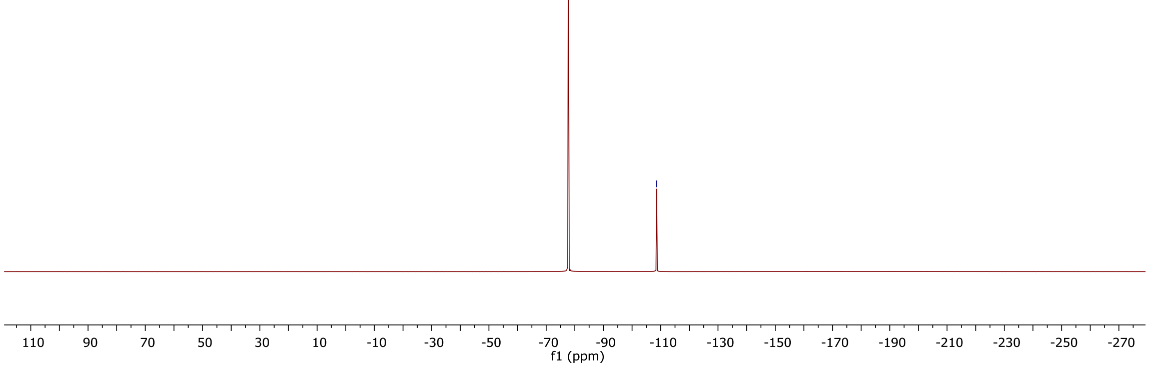

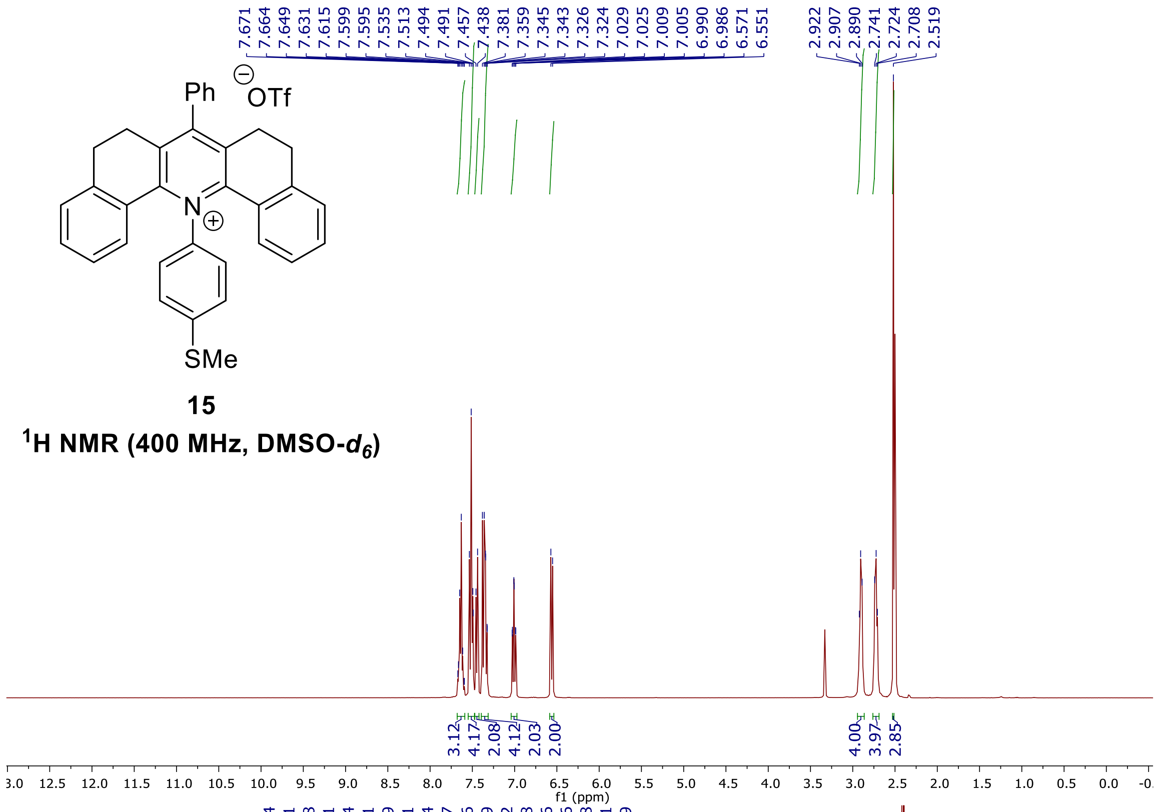
7.671 7.664 7.649 7.631 7.615 7.599 7.595 7.535 7.513 7.494 7.491 7.457 7.438 7.381 7.359 7.345 7.343 7.326 7.324 7.029 7.025 7.009 7.005 6.990 6.986 6.571 6.551
2.922 2.907 2.890 2.741 2.724 2.708 2.519
Ph
OTf
N
SMe
15
^{1}H NMR (400 MHz, DMSO-d_6)
3.12 4.17 2.08 4.12 2.03 2.00
4.00 3.97 2.85
f1 (ppm)

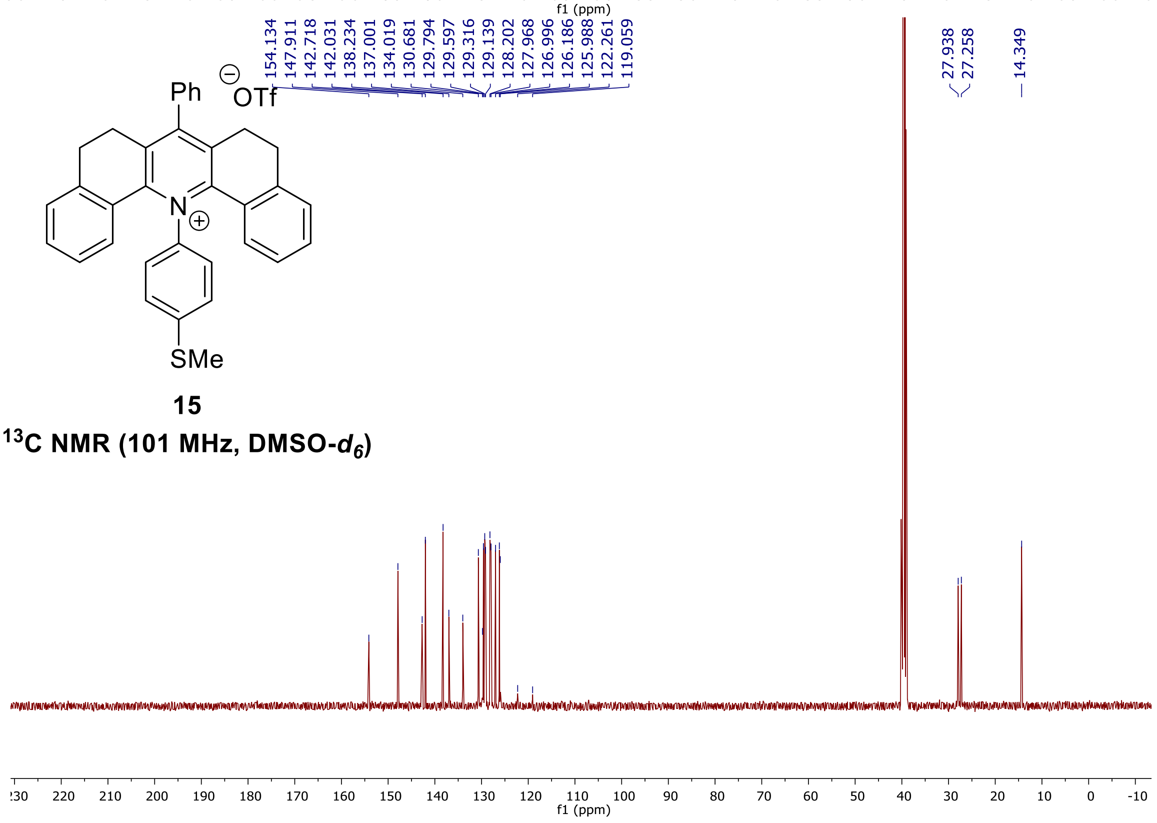
154.134 147.911 142.718 142.031 138.234 137.001 134.019 130.681 129.794 129.597 129.316 129.139 128.202 127.968 126.996 126.186 125.988 122.261 119.059
27.938 27.258
14.349
Ph
OTf
N
SMe
15
^{13}C NMR (101 MHz, DMSO-d_6)
f1 (ppm)

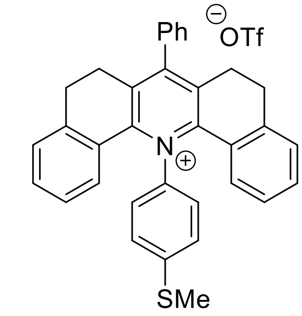

15

^{19}F NMR (470 MHz, DMSO-d_6)

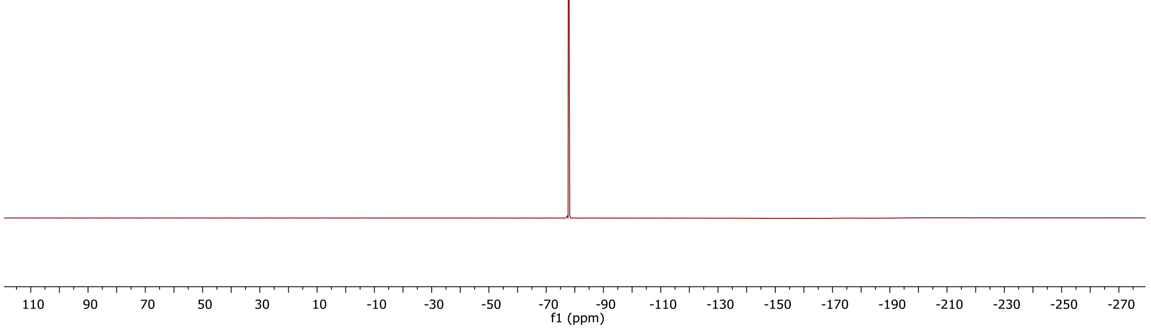

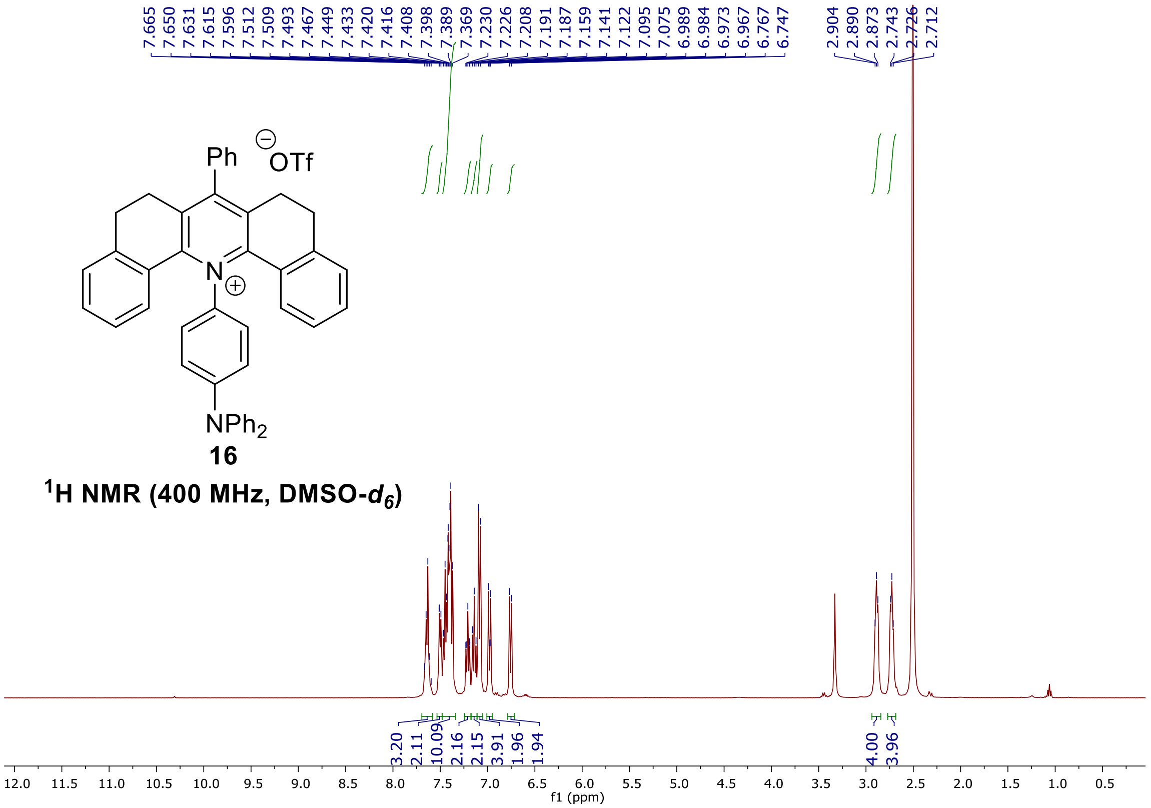
7.665
7.650
7.631
7.615
7.596
7.512
7.509
7.493
7.467
7.449
7.433
7.420
7.416
7.408
7.398
7.389
7.369
7.230
7.226
7.208
7.191
7.187
7.159
7.141
7.122
7.095
7.075
6.989
6.984
6.973
6.967
6.767
6.747
2.904
2.890
2.873
2.743
2.726
2.712
Ph
OTf
N
NPh2
16
¹H NMR (400 MHz, DMSO-d6)
3.20
2.11
10.09
2.16
2.15
3.91
1.96
1.94
4.00
3.96
f1 (ppm)

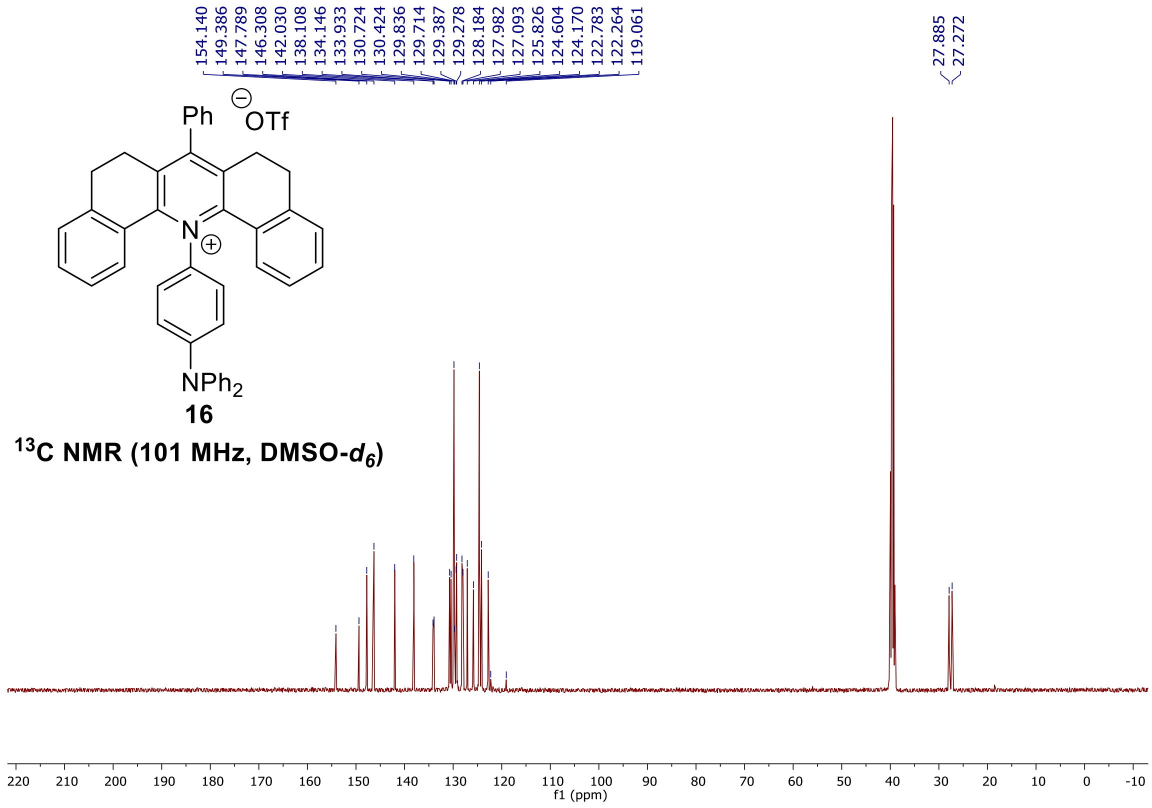
154.140
149.386
147.789
146.308
142.030
138.108
134.146
133.933
130.724
130.424
129.836
129.714
129.387
129.278
128.184
127.982
127.093
125.826
124.604
124.170
122.783
122.264
119.061
27.885
27.272
Ph
OTf
N
NPh2
16
¹³C NMR (101 MHz, DMSO-d6)
f1 (ppm)

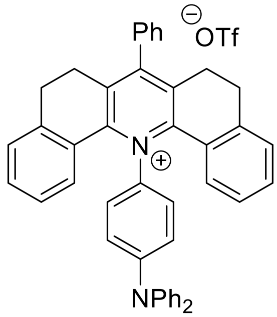

16

^{19}F NMR (470 MHz, DMSO-*d*$_6$)

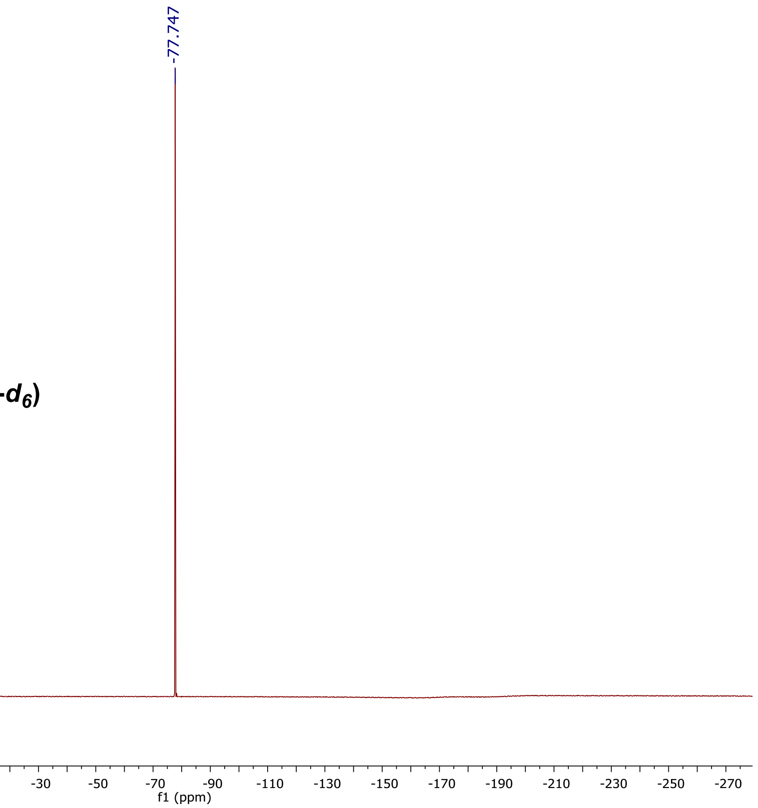

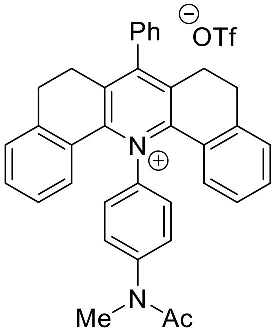

17

^{1}H NMR (300 MHz, DMSO-*d*6)

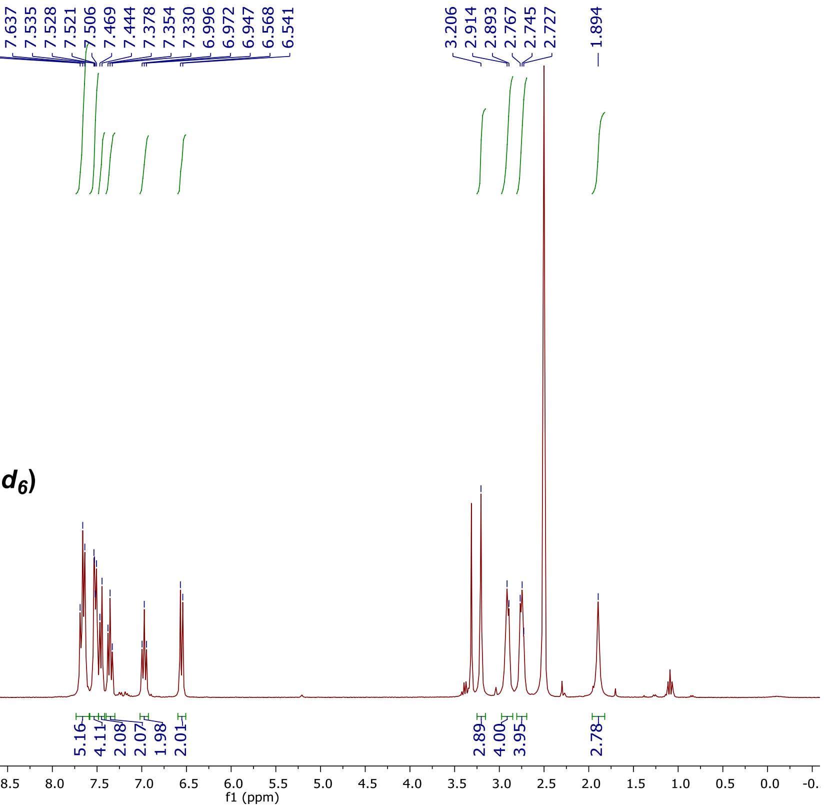

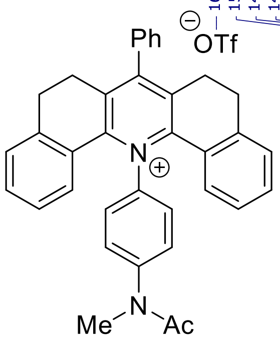

17

^{13}C NMR (101 MHz, DMSO-*d*6)

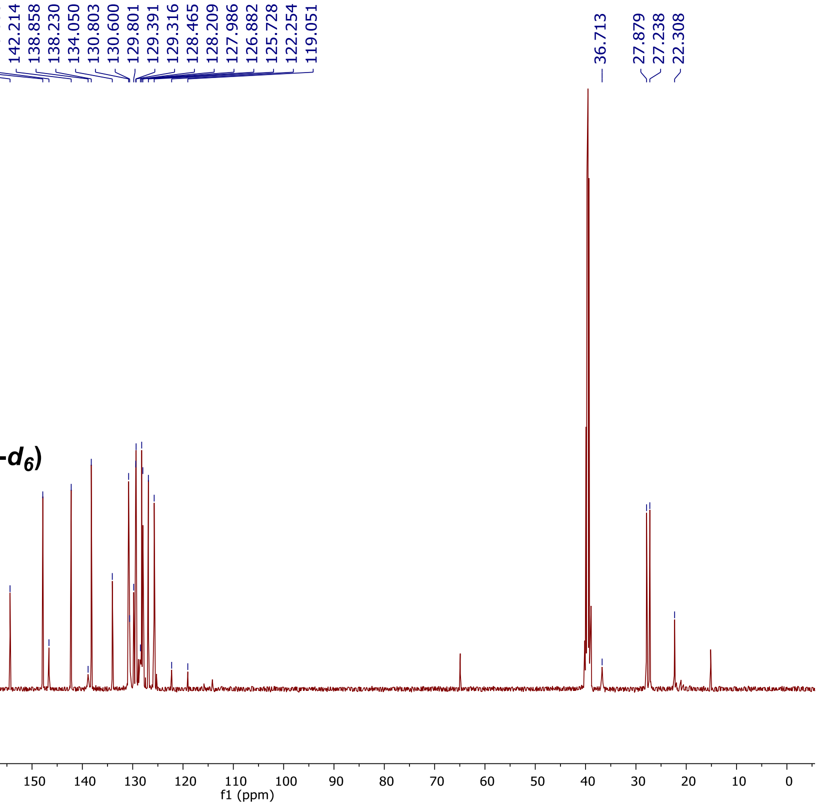

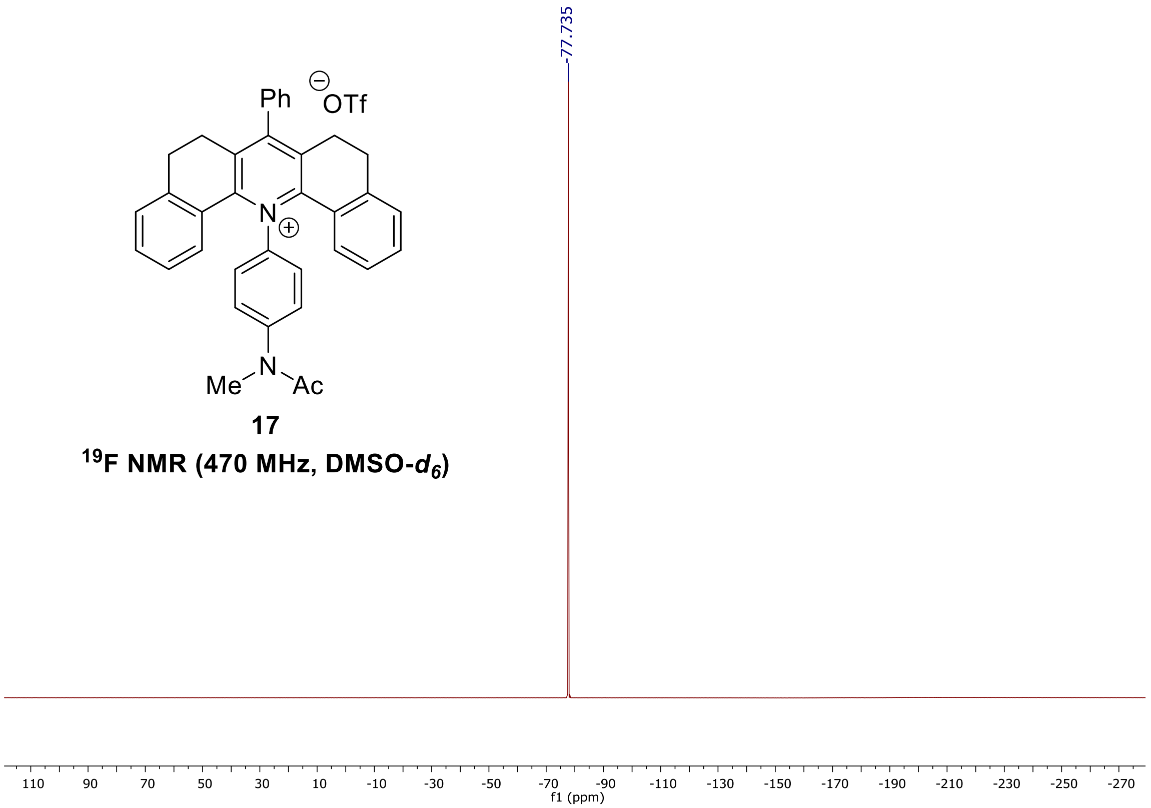

17

^{19}F NMR (470 MHz, DMSO-d_6)

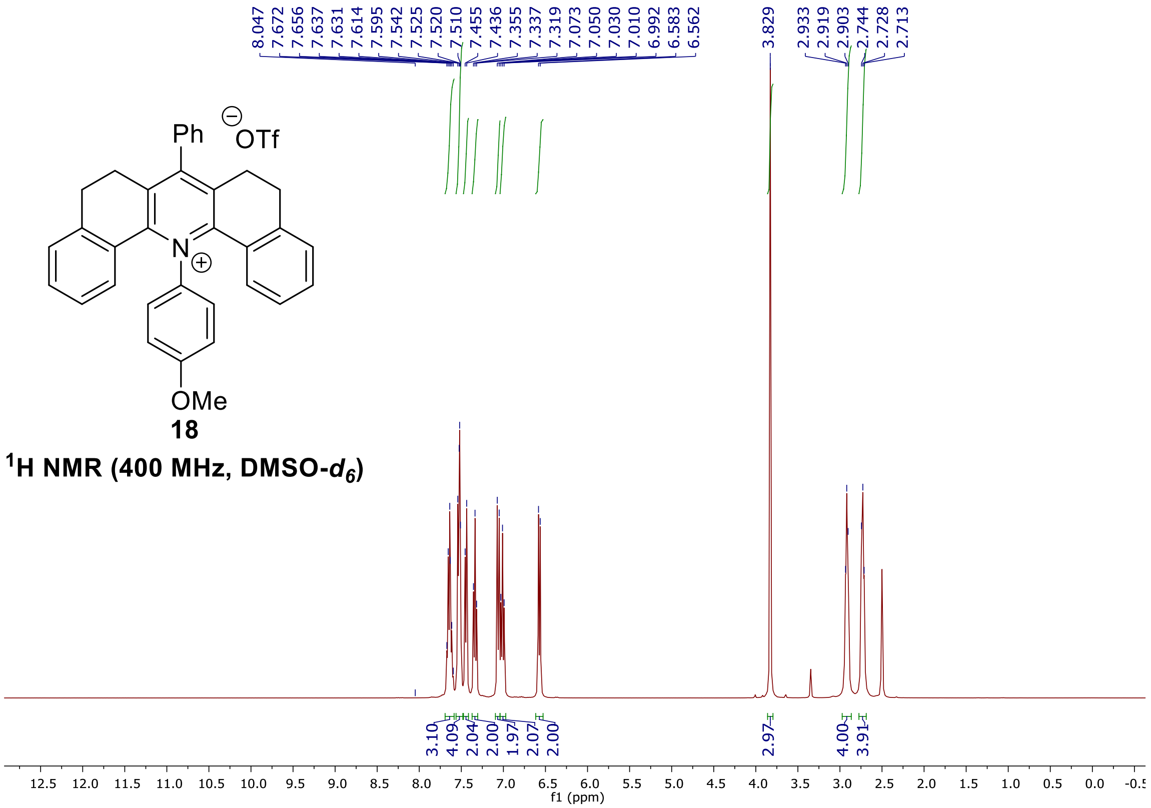

18

^{1}H NMR (400 MHz, DMSO-*d_6*)

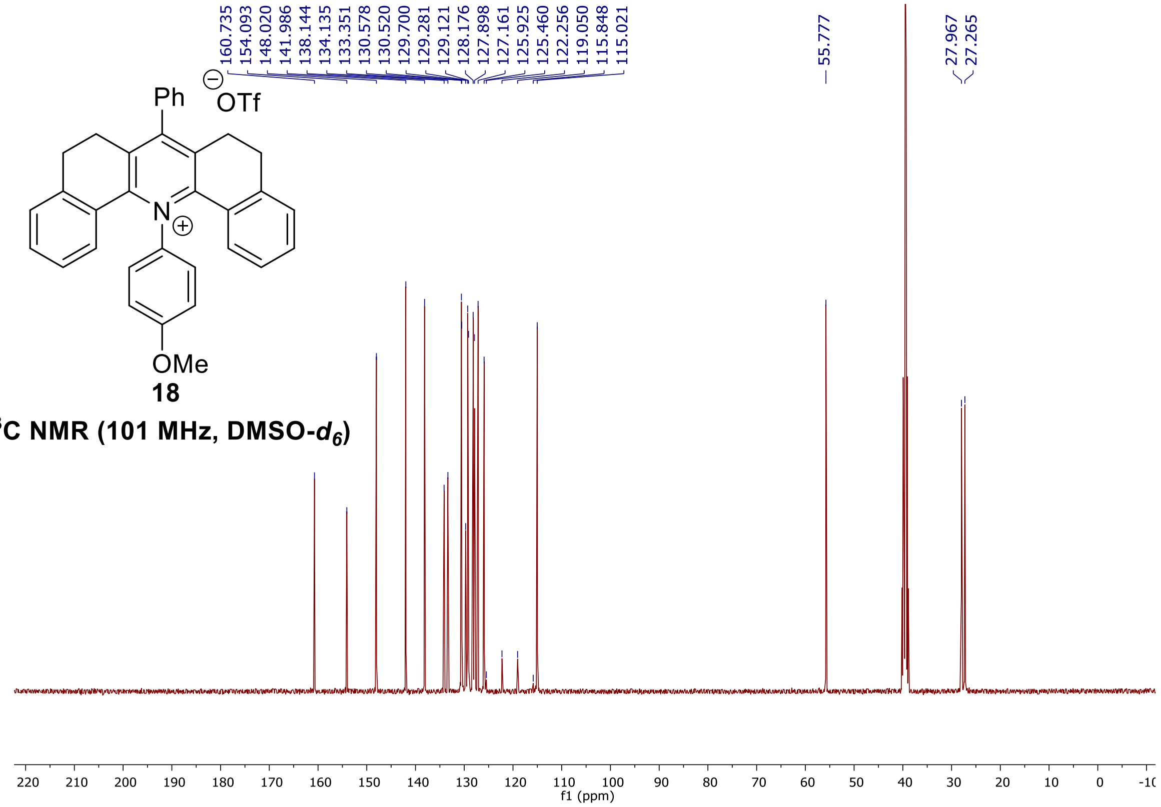

18

^{13}C NMR (101 MHz, DMSO-*d_6*)

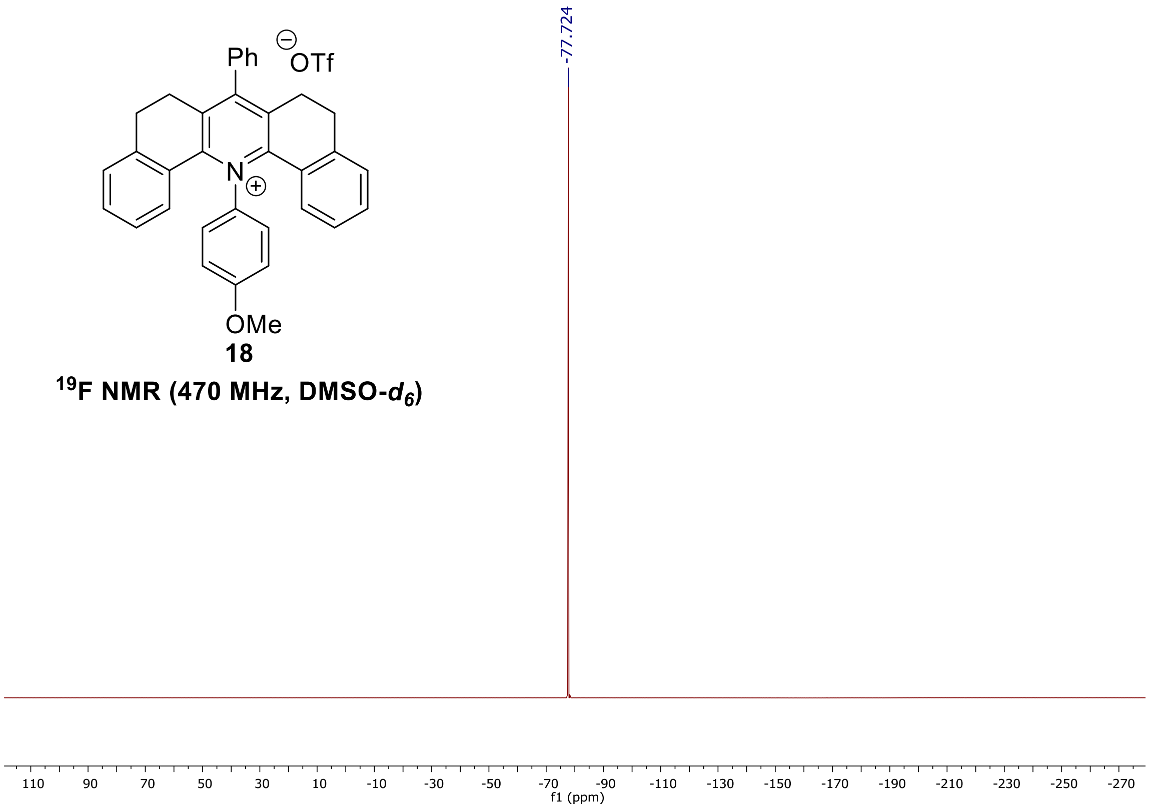

18

^{19}F NMR (470 MHz, DMSO-d_6)

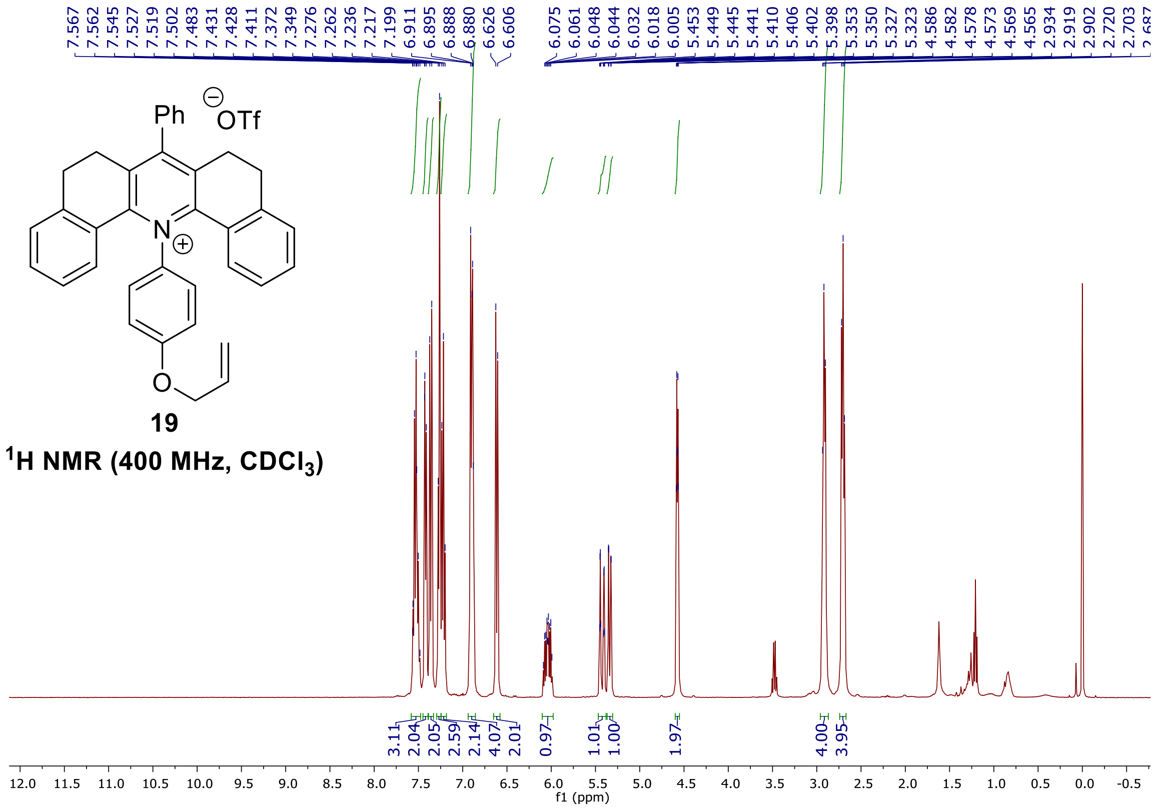

7.567 7.562 7.545 7.527 7.519 7.502 7.483 7.431 7.428 7.411 7.372 7.349 7.276 7.262 7.236 7.217 7.199 6.911 6.895 6.888 6.880 6.626 6.606 6.075 6.061 6.048 6.044 6.032 6.018 6.005 5.453 5.449 5.445 5.441 5.410 5.406 5.402 5.398 5.353 5.350 5.327 5.323 4.586 4.582 4.578 4.573 4.569 4.565 2.934 2.919 2.902 2.720 2.703
Ph
OTf
N
19
^{1}H NMR (400 MHz, CDCl$_3$)
3.11 2.04 2.05 2.59 2.14 4.07 2.01 0.97 1.01 1.00 1.97 4.00 3.95
12.0 11.5 11.0 10.5 10.0 9.5 9.0 8.5 8.0 7.5 7.0 6.5 6.0 5.5 5.0 4.5 4.0 3.5 3.0 2.5 2.0 1.5 1.0 0.5 0.0 -0.5
f1 (ppm)

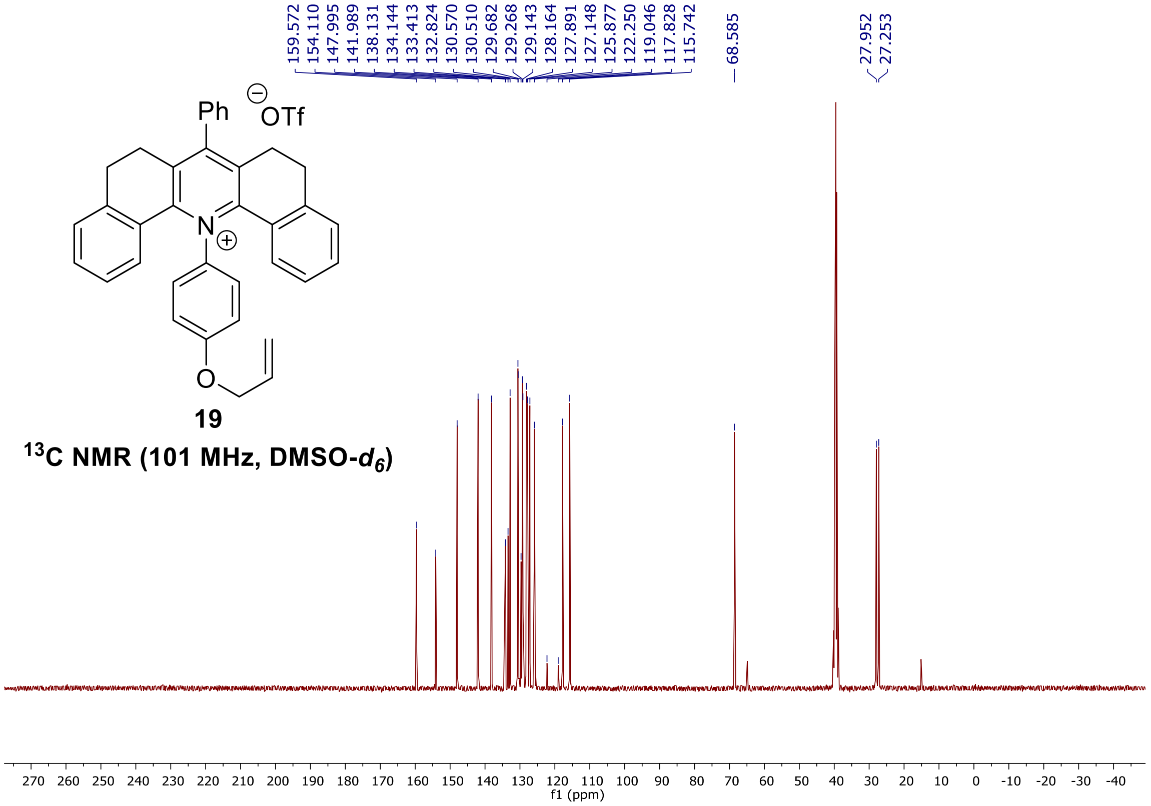

159.572 154.110 147.995 141.989 138.131 134.144 133.413 132.824 130.570 130.510 129.682 129.268 129.143 128.164 127.891 127.148 125.877 122.250 119.046 117.828 115.742
68.585
27.952 27.253
Ph
OTf
N
19
^{13}C NMR (101 MHz, DMSO-d_6)
270 260 250 240 230 220 210 200 190 180 170 160 150 140 130 120 110 100 90 80 70 60 50 40 30 20 10 0 -10 -20 -30 -40
f1 (ppm)

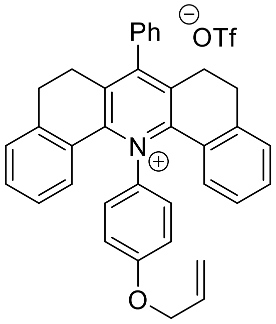

19

^{19}F NMR (470 MHz, DMSO-d_6)

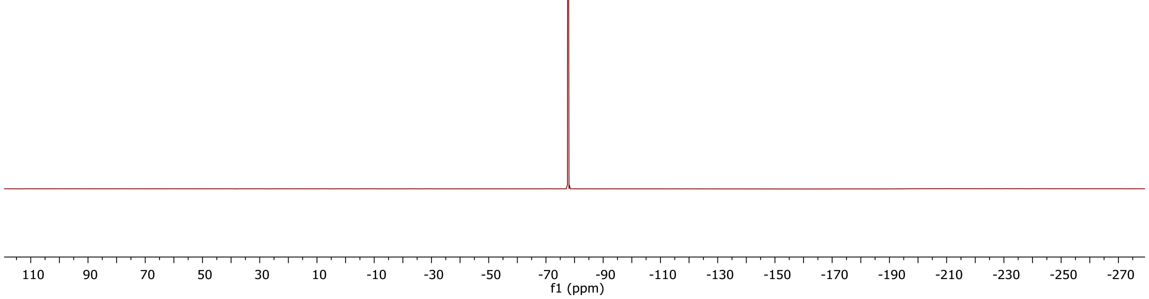

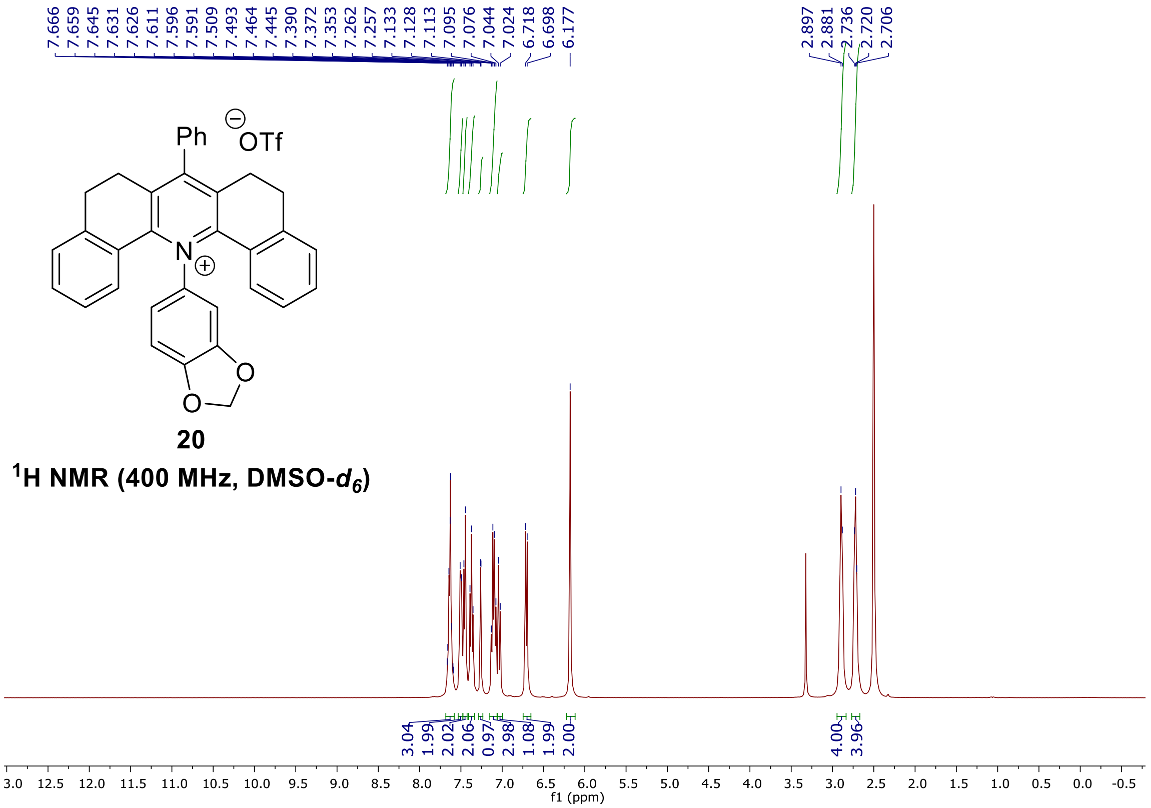

20

^{1}H NMR (400 MHz, DMSO-d_6)

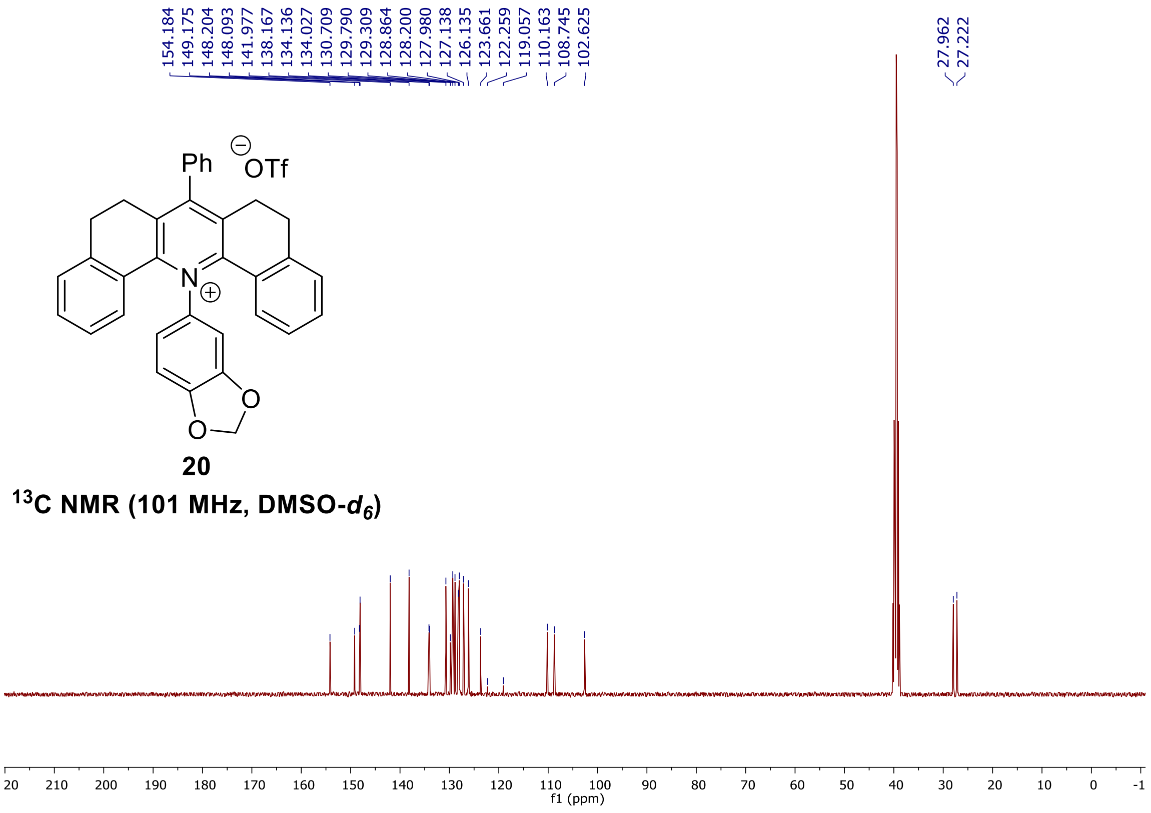

20

^{13}C NMR (101 MHz, DMSO-d_6)

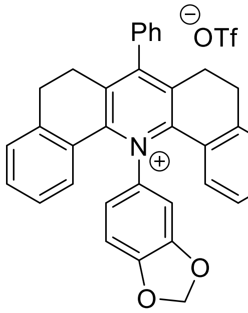

20

^{19}F NMR (470 MHz, DMSO-*d*$_6$)

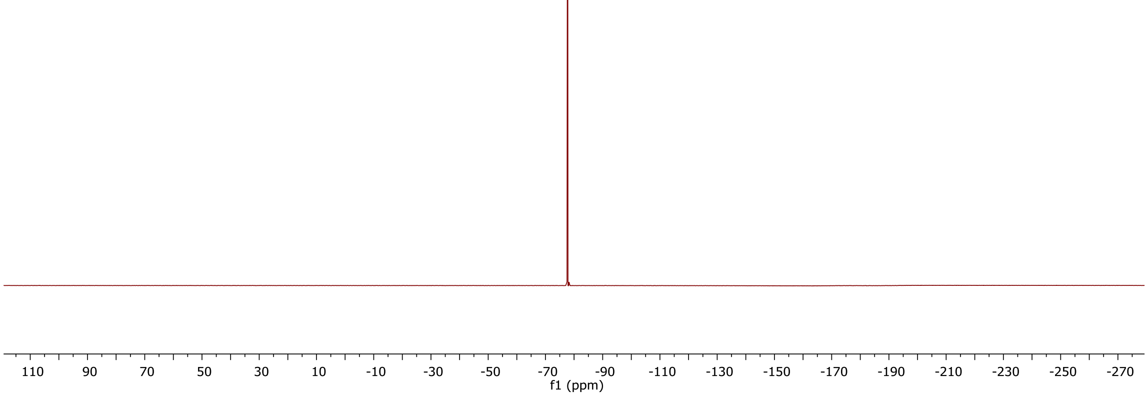

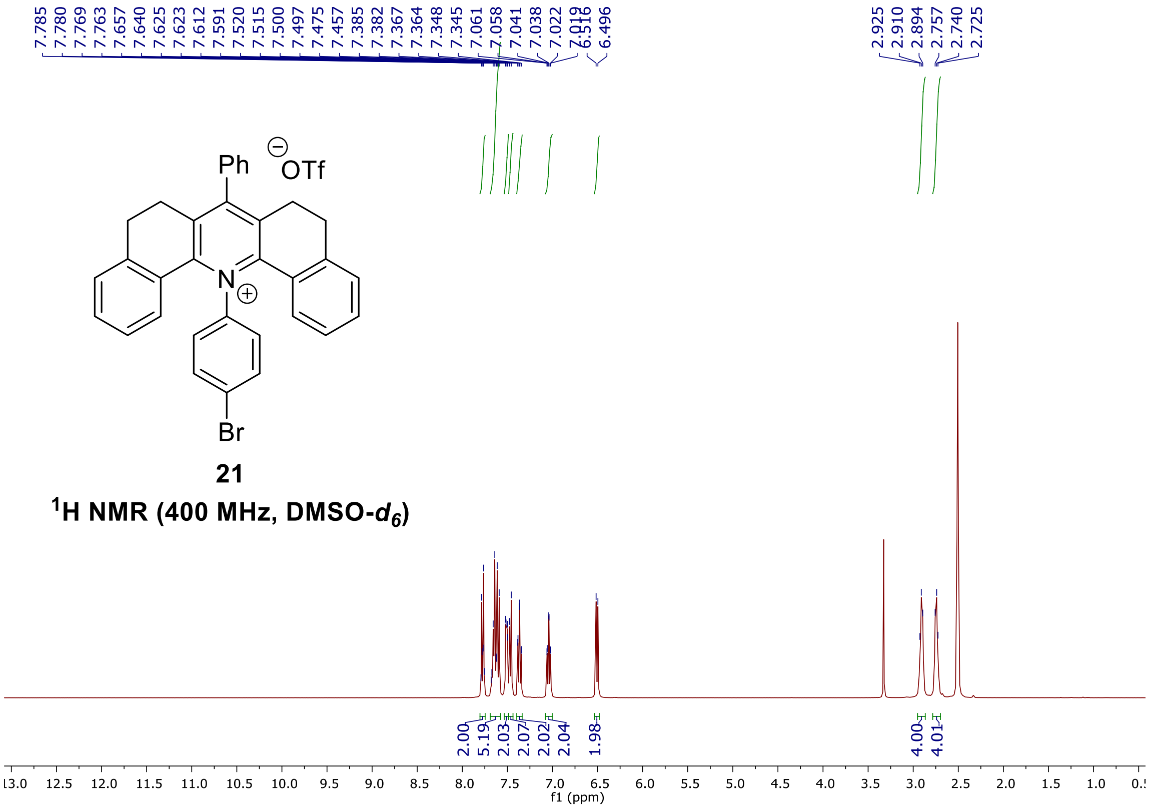
7.785
7.780
7.769
7.763
7.657
7.640
7.625
7.623
7.612
7.591
7.520
7.515
7.500
7.497
7.475
7.457
7.385
7.382
7.367
7.364
7.348
7.345
7.061
7.058
7.041
7.038
7.022
7.019
6.516
6.496
2.925
2.910
2.894
2.757
2.740
2.725
Ph
OTf
N
Br
21
^{1}H NMR (400 MHz, DMSO-d_6)
2.00
5.19
2.03
2.07
2.02
2.04
1.98
4.00
4.01
f1 (ppm)

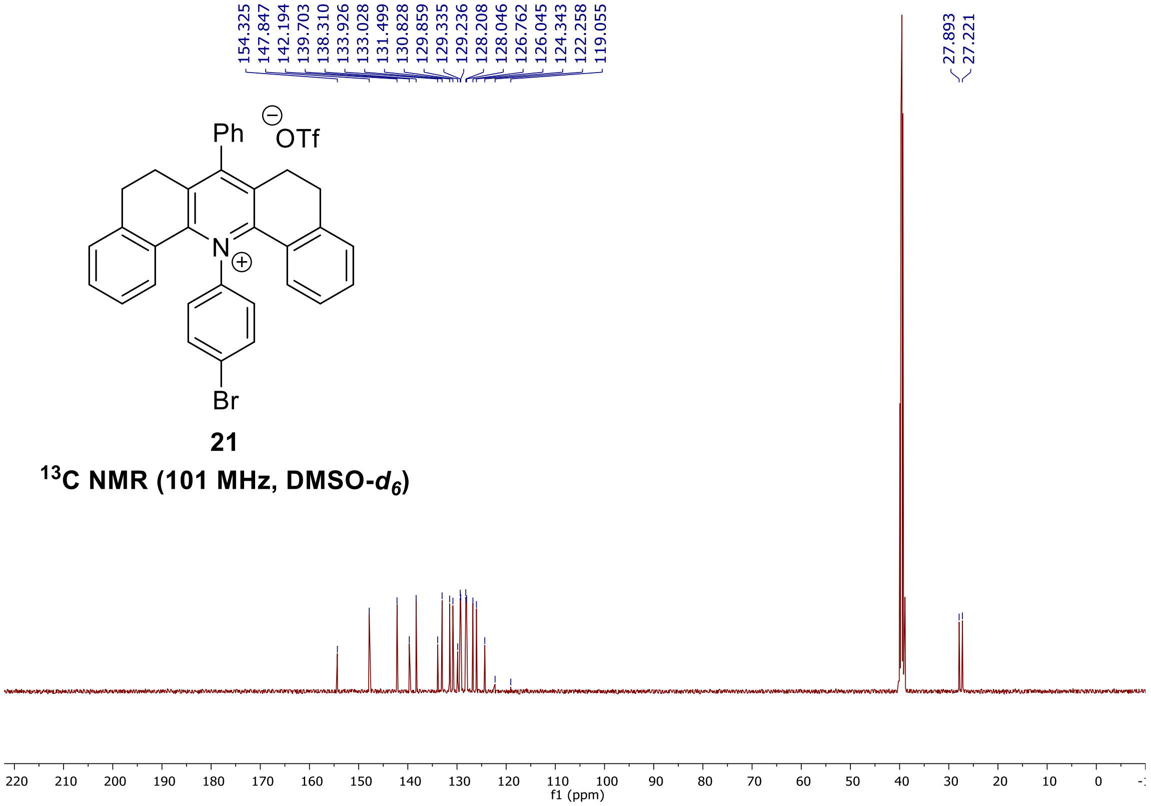
154.325
147.847
142.194
139.703
138.310
133.926
133.028
131.499
130.828
129.859
129.335
129.236
128.208
128.046
126.762
126.045
124.343
122.258
119.055
27.893
27.221
Ph
OTf
N
Br
21
^{13}C NMR (101 MHz, DMSO-d_6)
f1 (ppm)

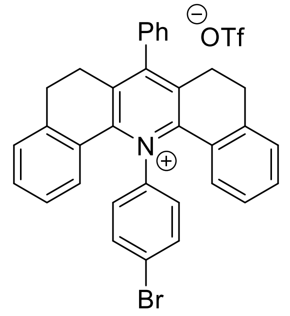

21

^{19}F NMR (470 MHz, DMSO-d_6)

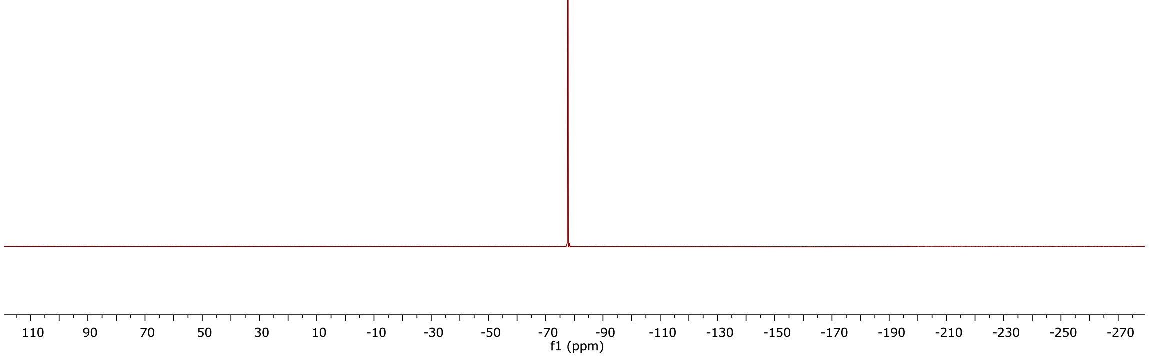

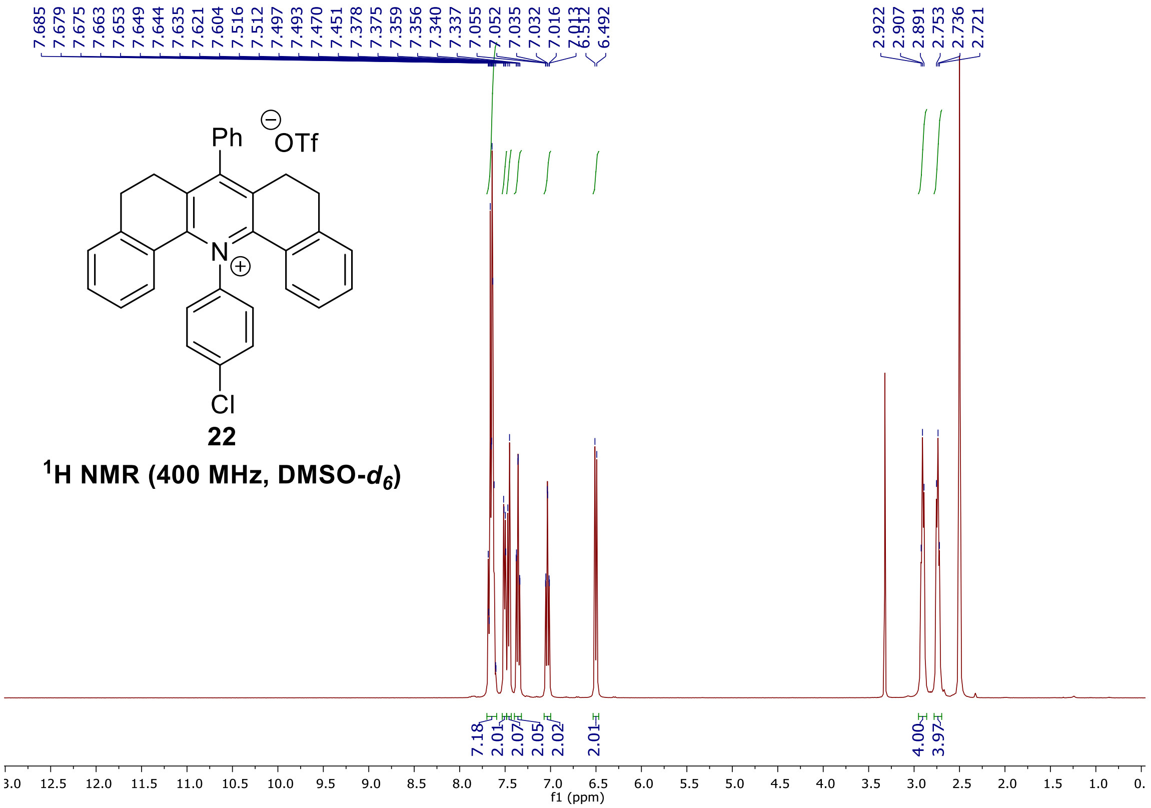

^{1}H NMR (400 MHz, DMSO-d_6)

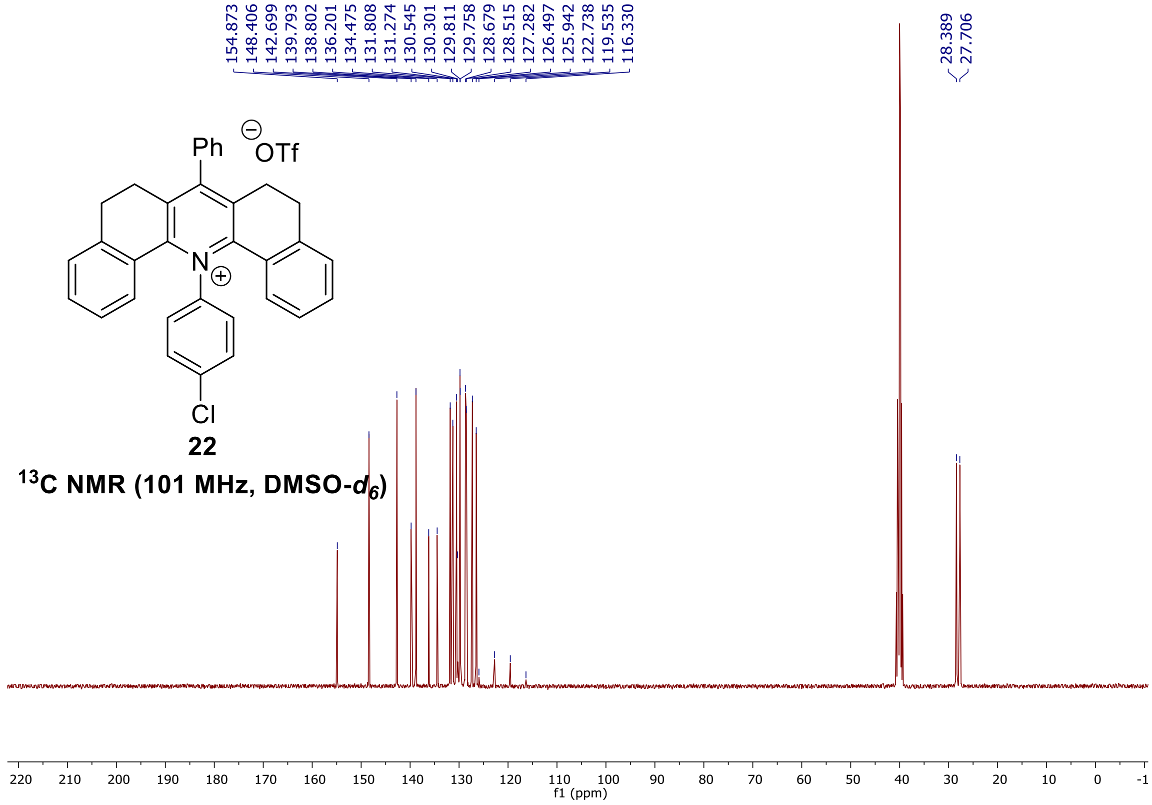

^{13}C NMR (101 MHz, DMSO-d_6)

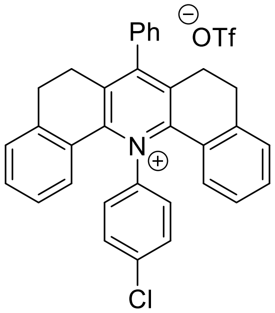

22

^{19}F NMR (470 MHz, DMSO-*d_6*)

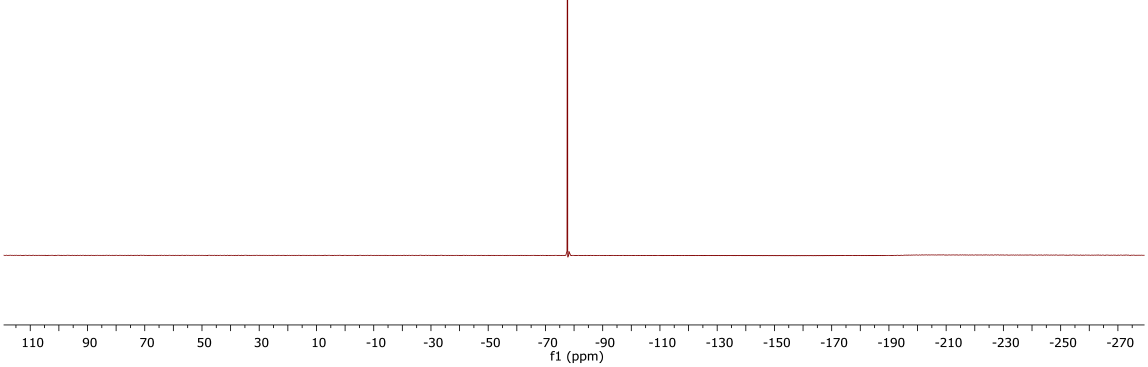

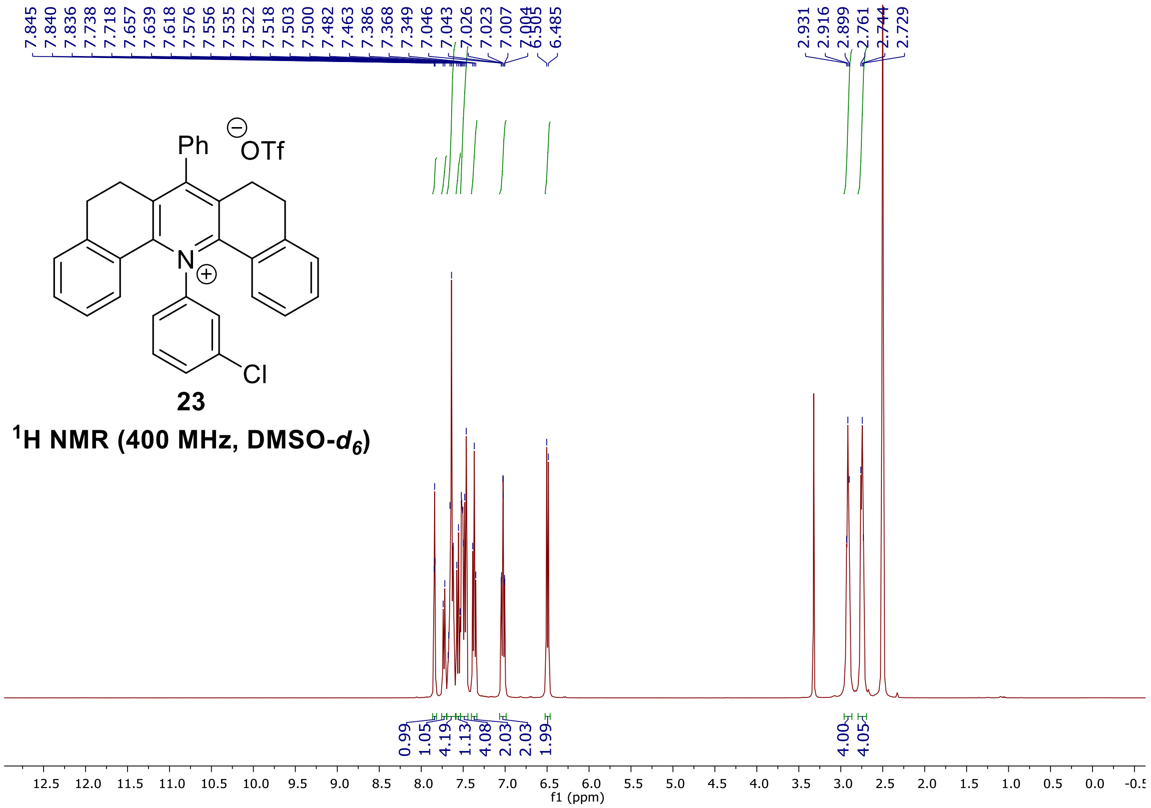
7.845 7.840 7.836 7.738 7.718 7.657 7.639 7.618 7.576 7.556 7.535 7.522 7.518 7.503 7.500 7.482 7.463 7.386 7.368 7.349 7.046 7.043 7.026 7.023 7.007 7.004 6.505 6.485
2.931 2.916 2.899 2.761 2.744 2.729
Ph
OTf
N
Cl
23
^{1}H NMR (400 MHz, DMSO-d_6)
0.99 1.05 4.19 1.13 4.08 2.03 2.03 1.99
4.00 4.05
f1 (ppm)

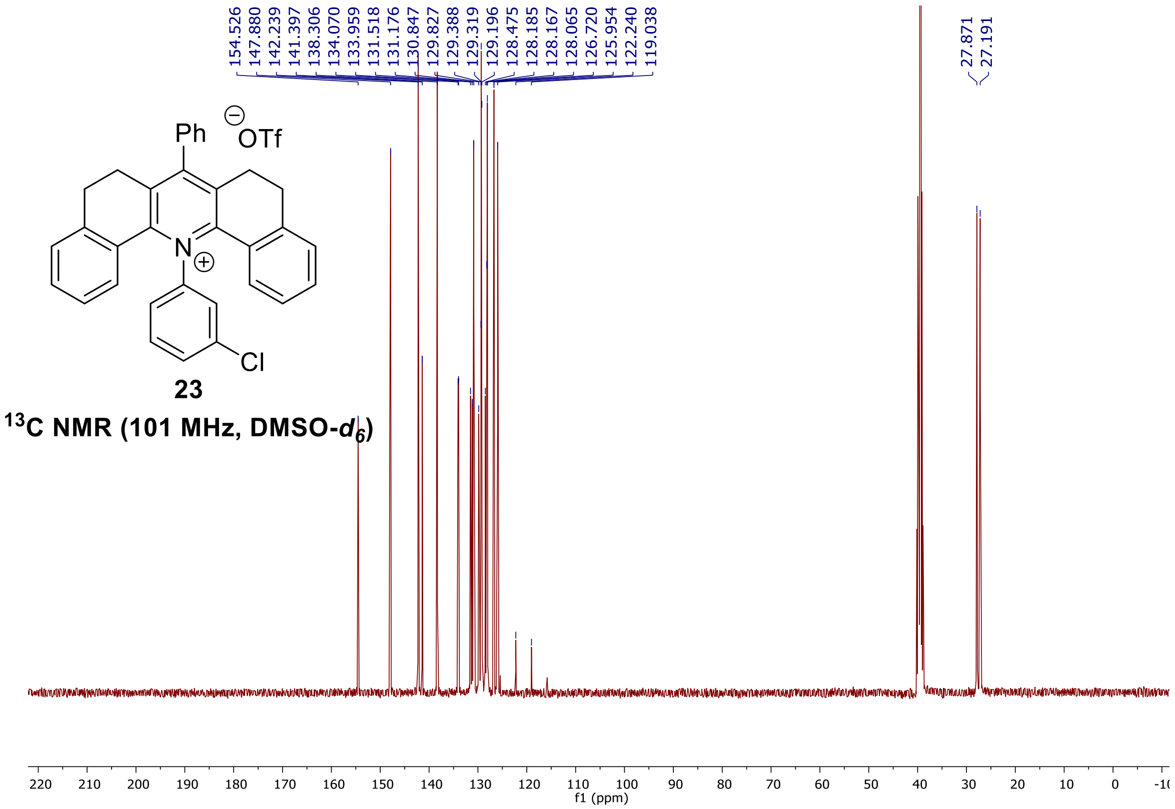
154.526 147.880 142.239 141.397 138.306 134.070 133.959 131.518 131.176 130.847 129.827 129.388 129.319 129.196 128.475 128.185 128.167 128.065 126.720 125.954 122.240 119.038
27.871 27.191
Ph
OTf
N
Cl
23
^{13}C NMR (101 MHz, DMSO-d_6)
f1 (ppm)

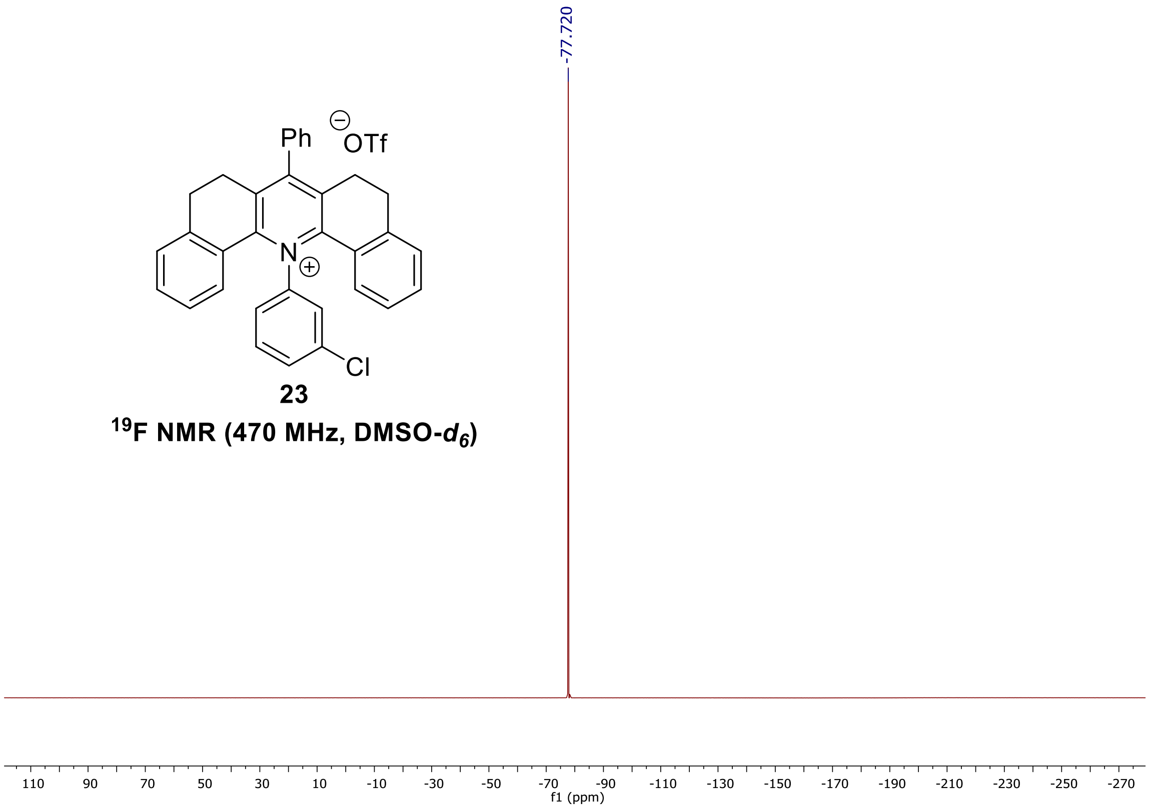

23

^{19}F NMR (470 MHz, DMSO-d_6)

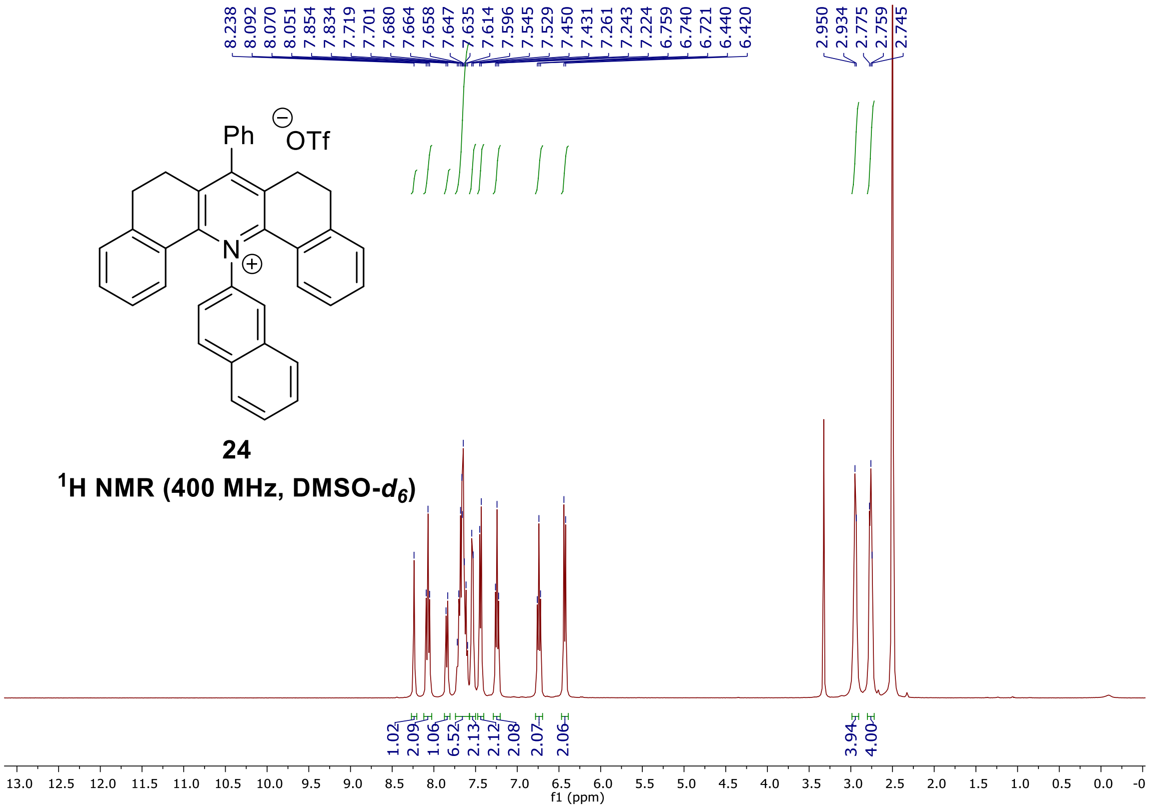

24

^{1}H NMR (400 MHz, DMSO-d_6)

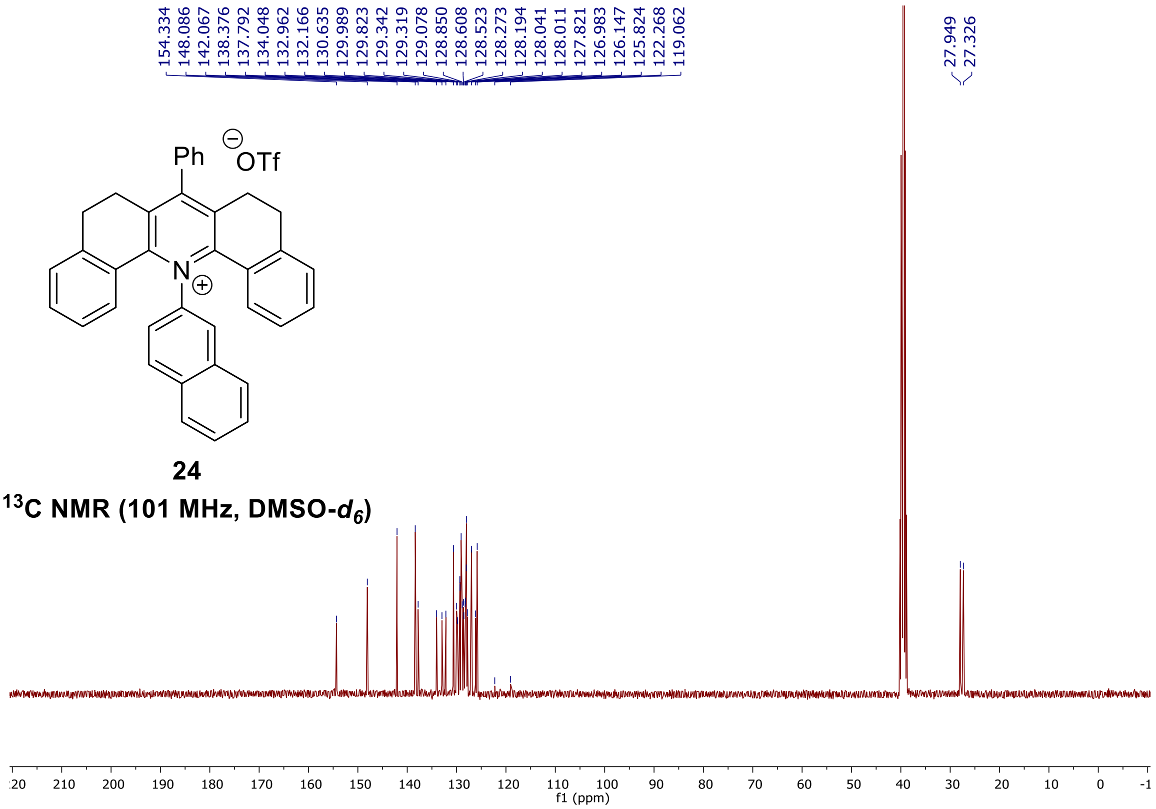

24

^{13}C NMR (101 MHz, DMSO-d_6)

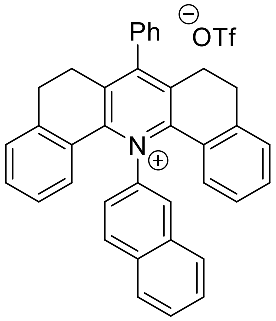

24

^{19}F NMR (470 MHz, DMSO-d_6)

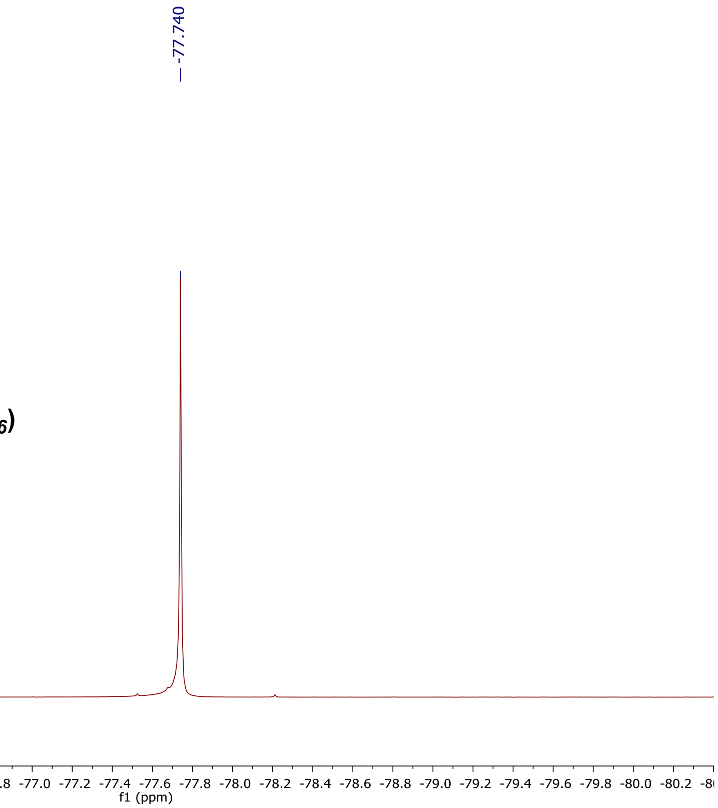

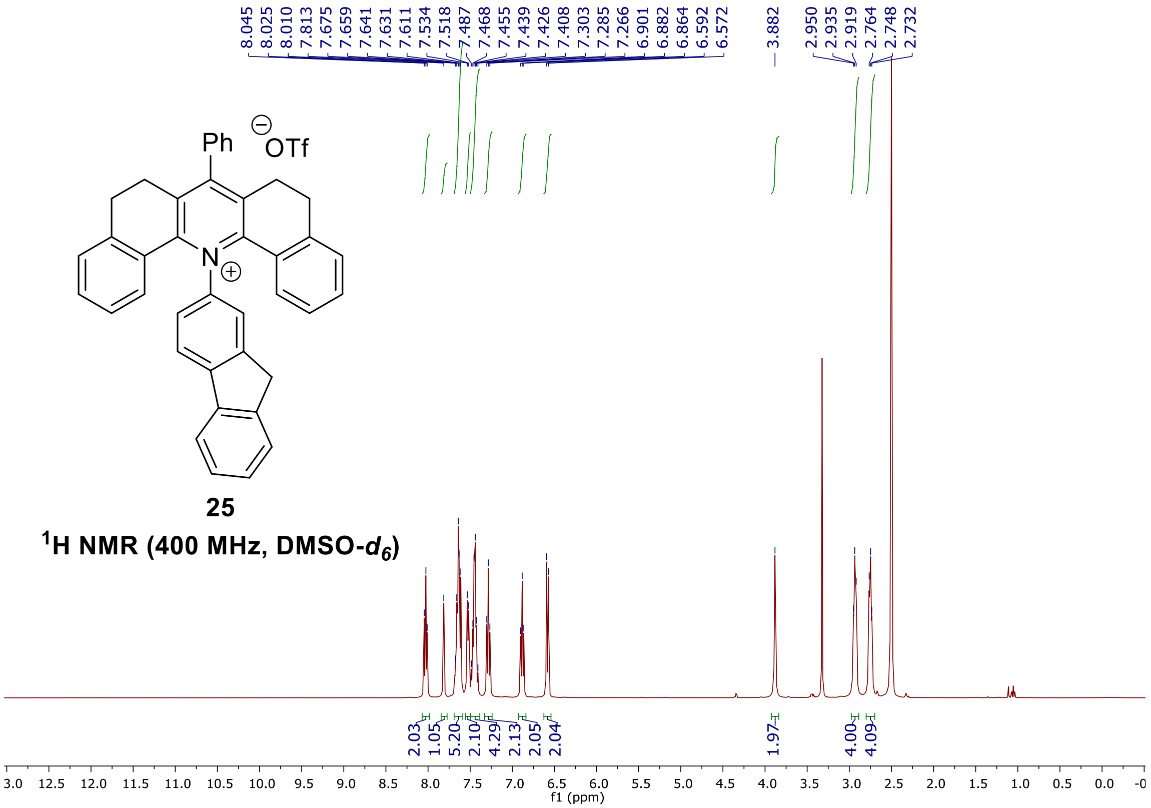

25

^{1}H NMR (400 MHz, DMSO-d_6)

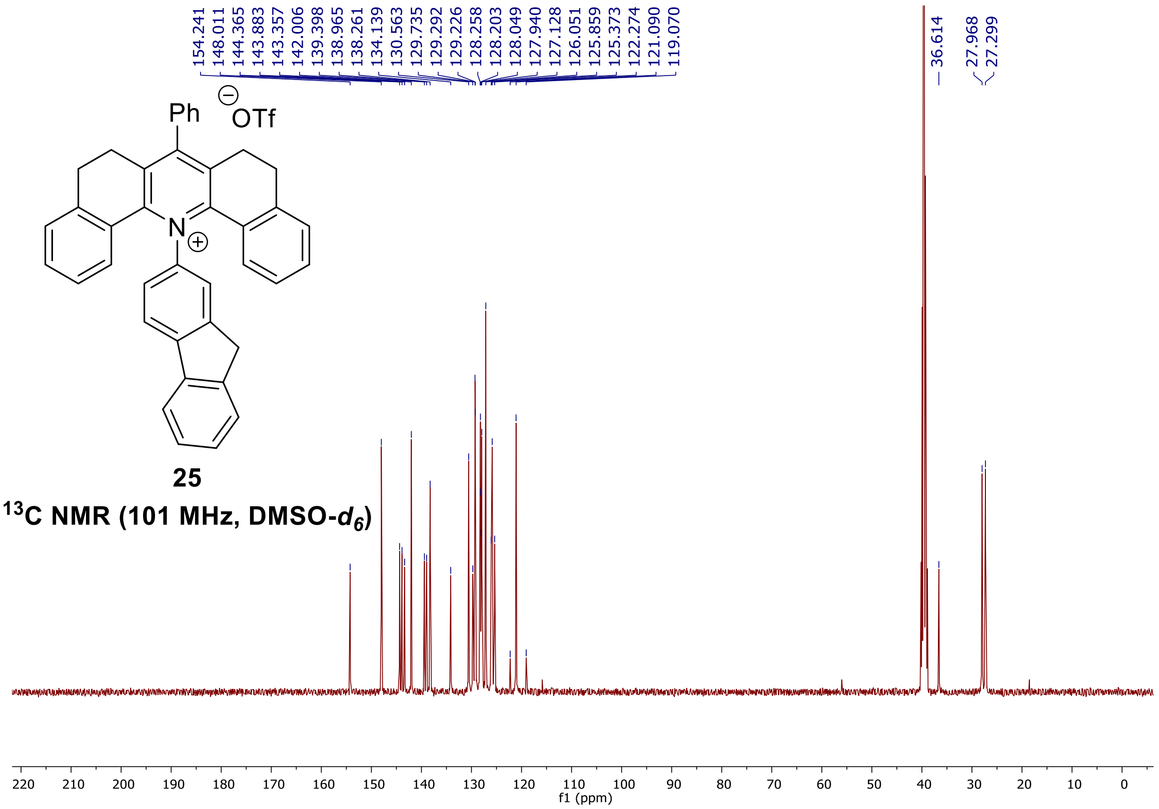

25

^{13}C NMR (101 MHz, DMSO-d_6)

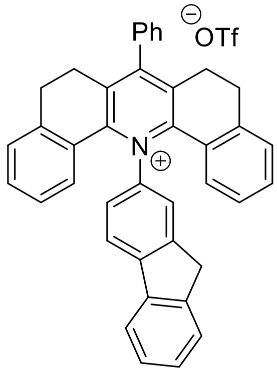

25

^{19}F NMR (470 MHz, DMSO-d_6)

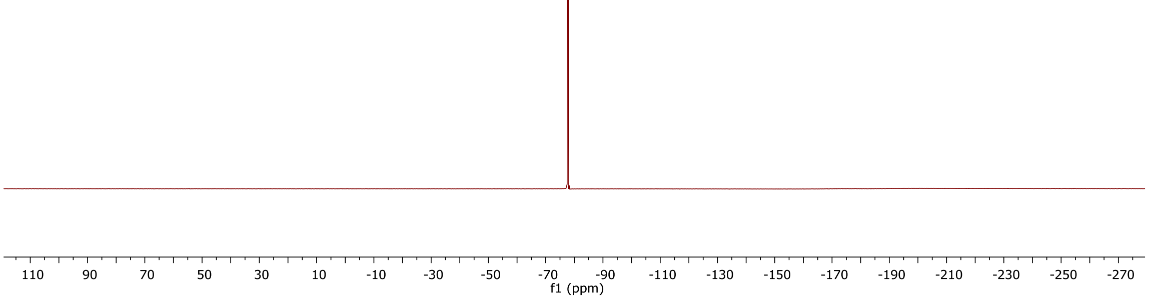

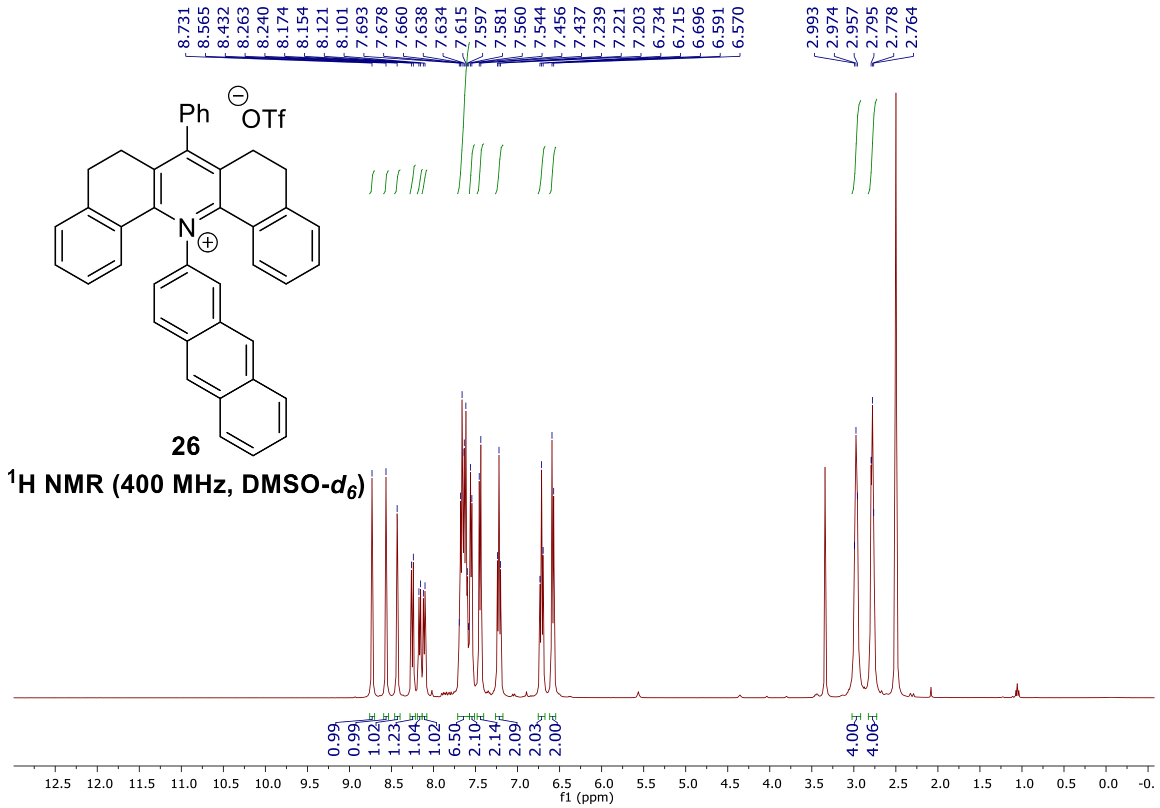
8.731 8.565 8.432 8.263 8.240 8.174 8.154 8.121 8.101 7.693 7.678 7.660 7.638 7.634 7.615 7.597 7.581 7.560 7.544 7.456 7.437 7.239 7.221 7.203 6.734 6.715 6.696 6.591 6.570
2.993 2.974 2.957 2.795 2.778 2.764
Ph
OTf
N
26
^{1}H NMR (400 MHz, DMSO-d_6)
0.99 0.99 1.02 1.23 1.04 1.02 6.50 2.10 2.14 2.09 2.03 2.00
4.00 4.06
f1 (ppm)

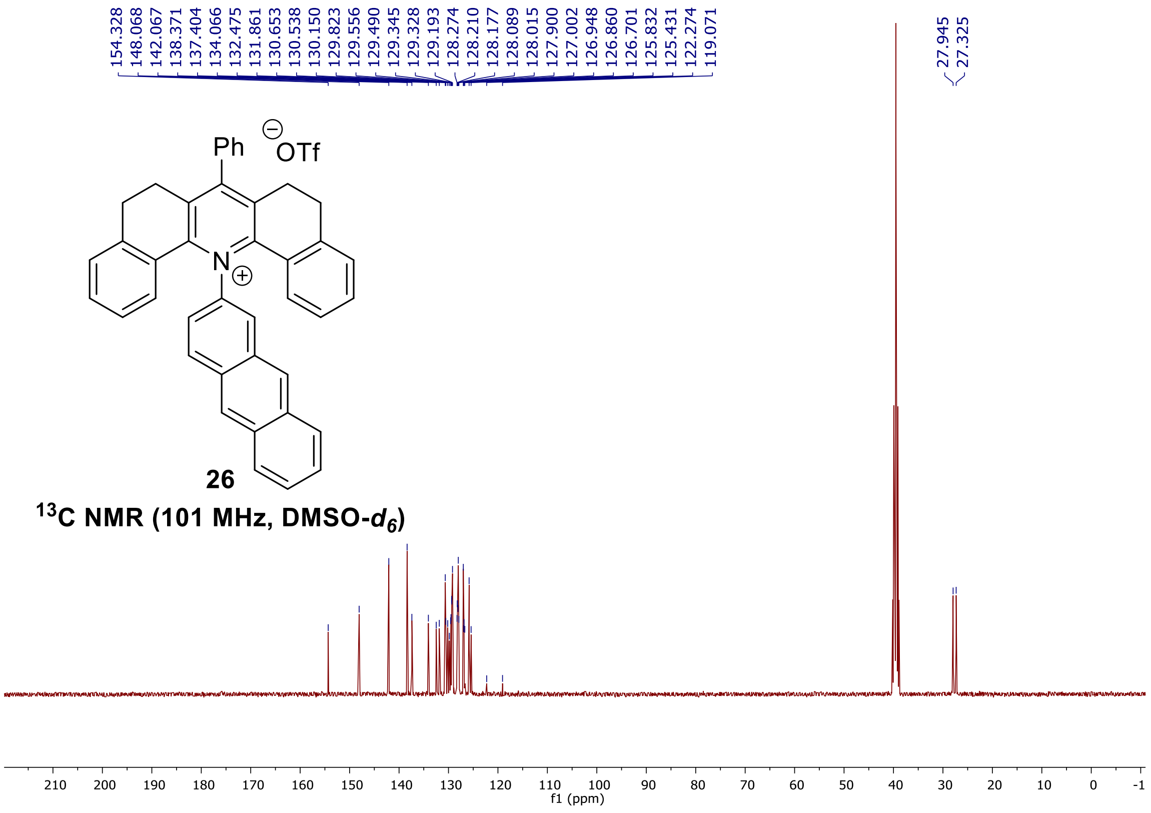
154.328 148.068 142.067 138.371 137.404 134.066 132.475 131.861 130.653 130.538 130.150 129.823 129.556 129.490 129.345 129.328 129.193 128.274 128.210 128.177 128.089 128.015 127.900 127.002 126.948 126.860 126.701 125.832 125.431 122.274 119.071
27.945 27.325
Ph
OTf
N
26
^{13}C NMR (101 MHz, DMSO-d_6)
f1 (ppm)

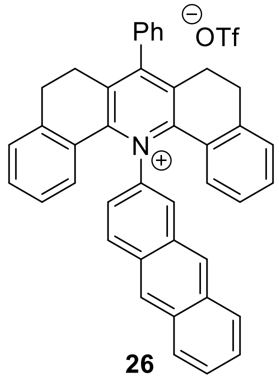

26

^{19}F NMR (470 MHz, DMSO-d_6)

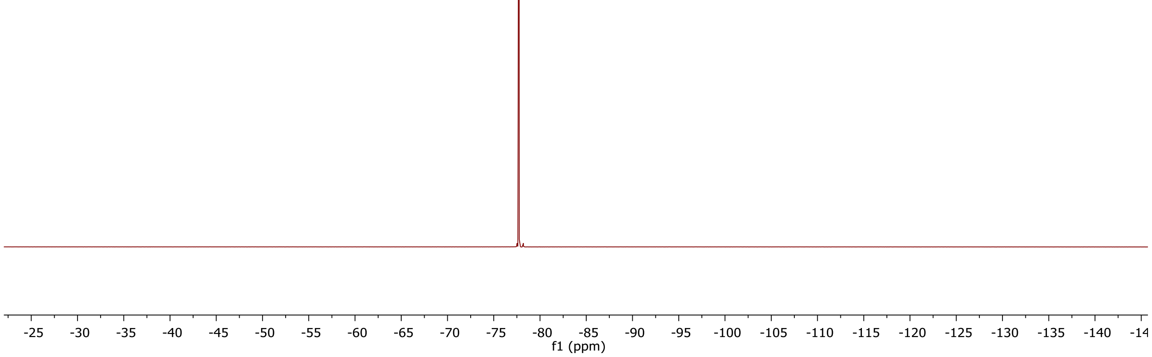

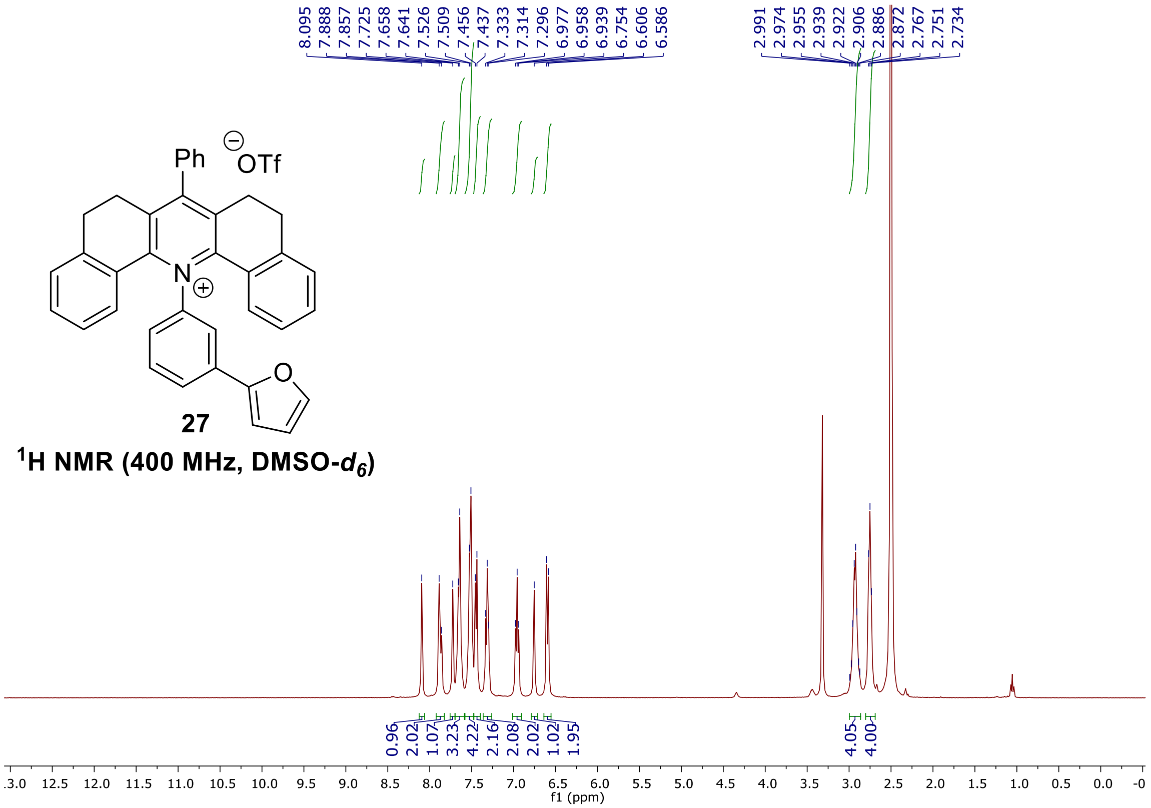
8.095 7.888 7.857 7.725 7.658 7.641 7.526 7.509 7.456 7.437 7.333 7.314 7.296 6.977 6.958 6.939 6.754 6.606 6.586
2.991 2.974 2.955 2.939 2.922 2.906 2.886 2.872 2.767 2.751 2.734
Ph
OTf
N
O
27
^{1}H NMR (400 MHz, DMSO-d_6)
0.96 2.02 1.07 3.23 4.22 2.16 2.08 2.02 1.02 1.95
4.05 4.00
f1 (ppm)

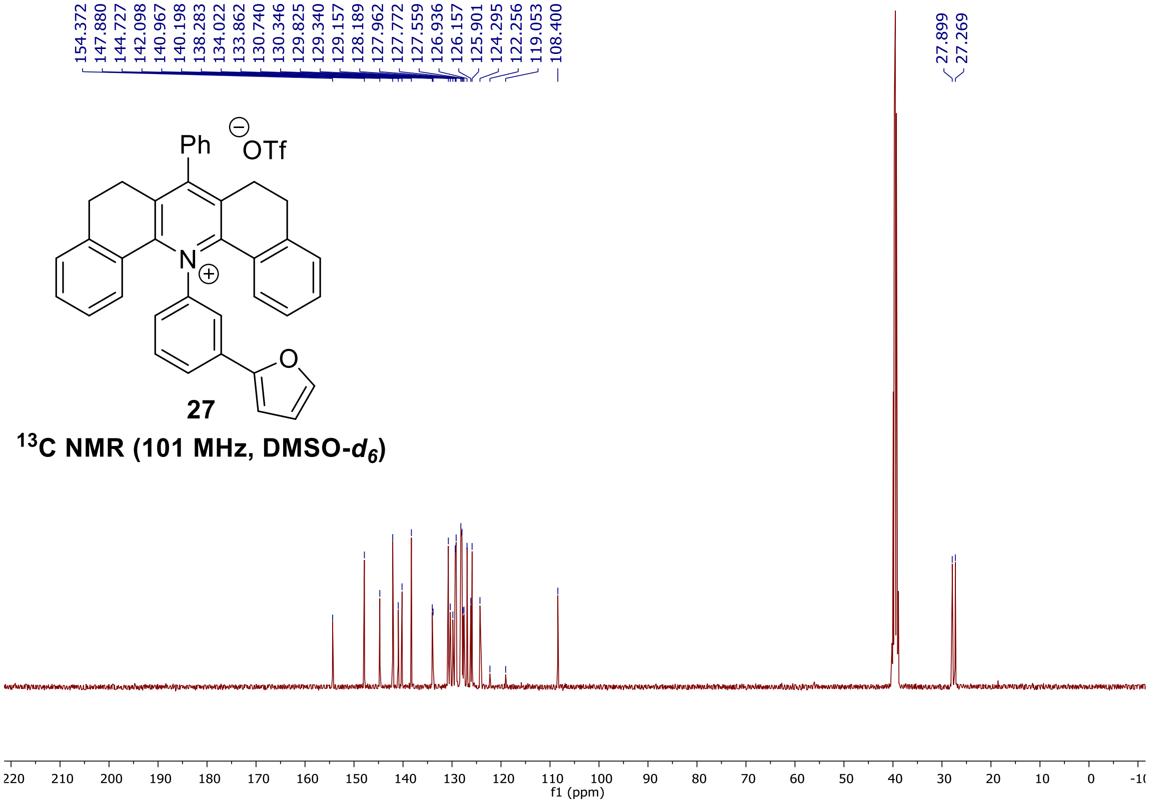
154.372 147.880 144.727 142.098 140.967 140.198 138.283 134.022 133.862 130.740 130.346 129.825 129.340 129.157 128.189 127.962 127.772 127.559 126.936 126.157 125.901 124.295 122.256 119.053 108.400
27.899 27.269
Ph
OTf
N
O
27
^{13}C NMR (101 MHz, DMSO-d_6)
f1 (ppm)

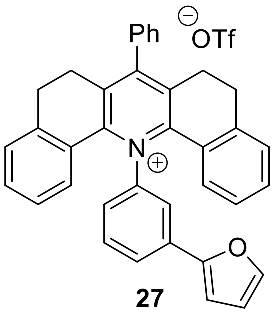

27

^{19}F NMR (470 MHz, DMSO-d_6)

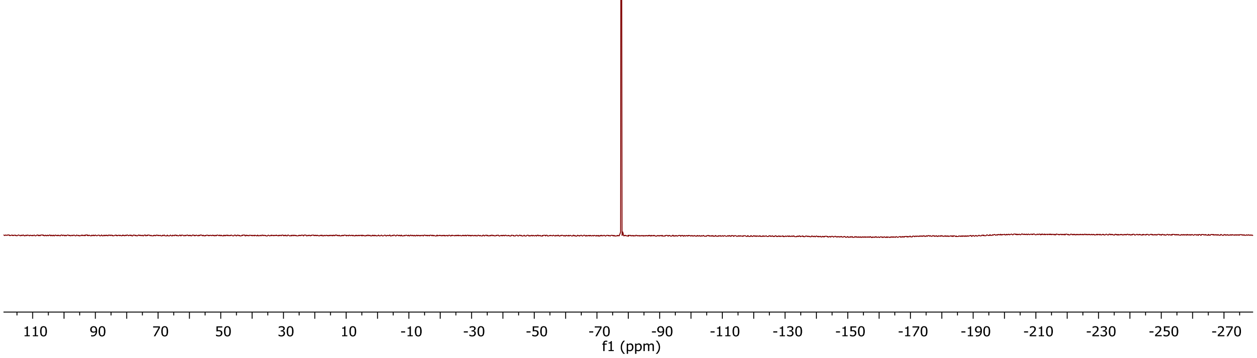

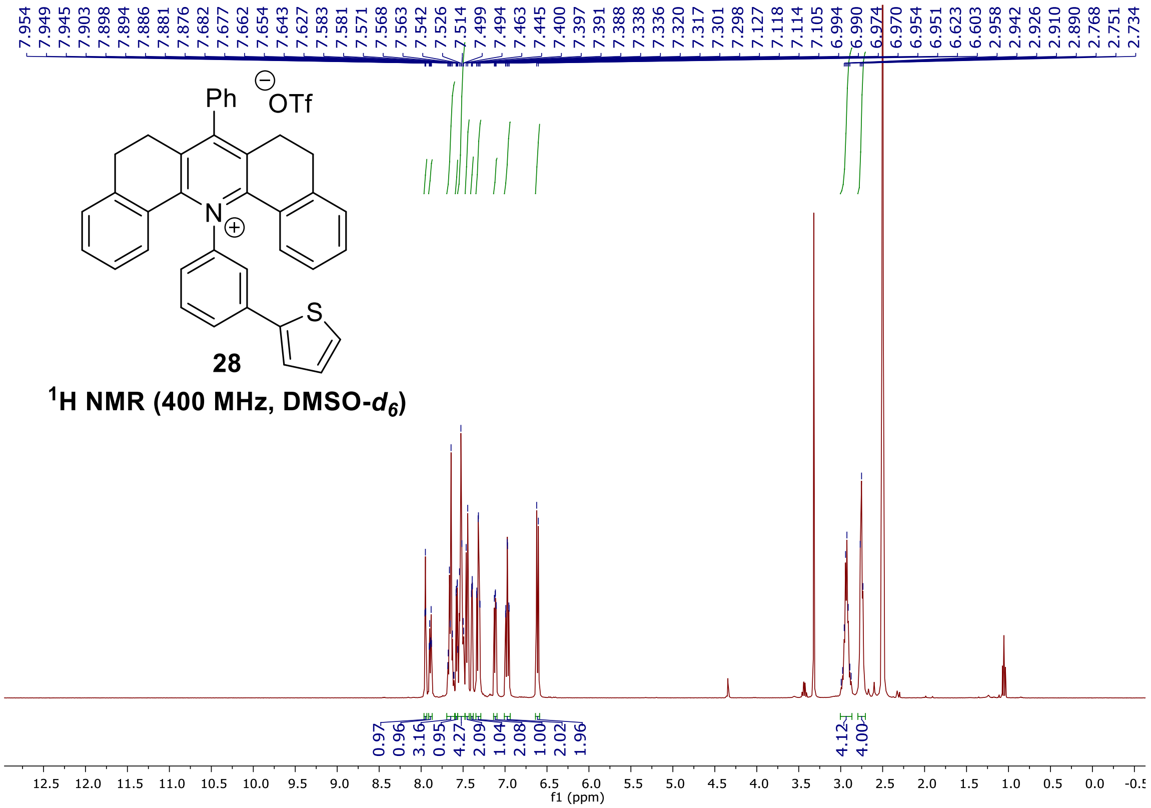
7.954 7.949 7.945 7.903 7.898 7.894 7.886 7.881 7.876 7.682 7.677 7.662 7.654 7.643 7.627 7.583 7.581 7.571 7.568 7.563 7.542 7.526 7.514 7.499 7.494 7.463 7.445 7.400 7.397 7.391 7.388 7.338 7.336 7.320 7.317 7.301 7.298 7.127 7.118 7.114 7.105 6.994 6.990 6.974 6.970 6.954 6.951 6.623 6.603 2.958 2.942 2.926 2.910 2.890 2.768 2.751 2.734
Ph
OTf
N
S
28
^{1}H NMR (400 MHz, DMSO-d_6)
0.97 0.96 3.16 0.95 4.27 2.09 1.04 2.08 1.00 2.02 1.96 4.12 4.00
f1 (ppm)

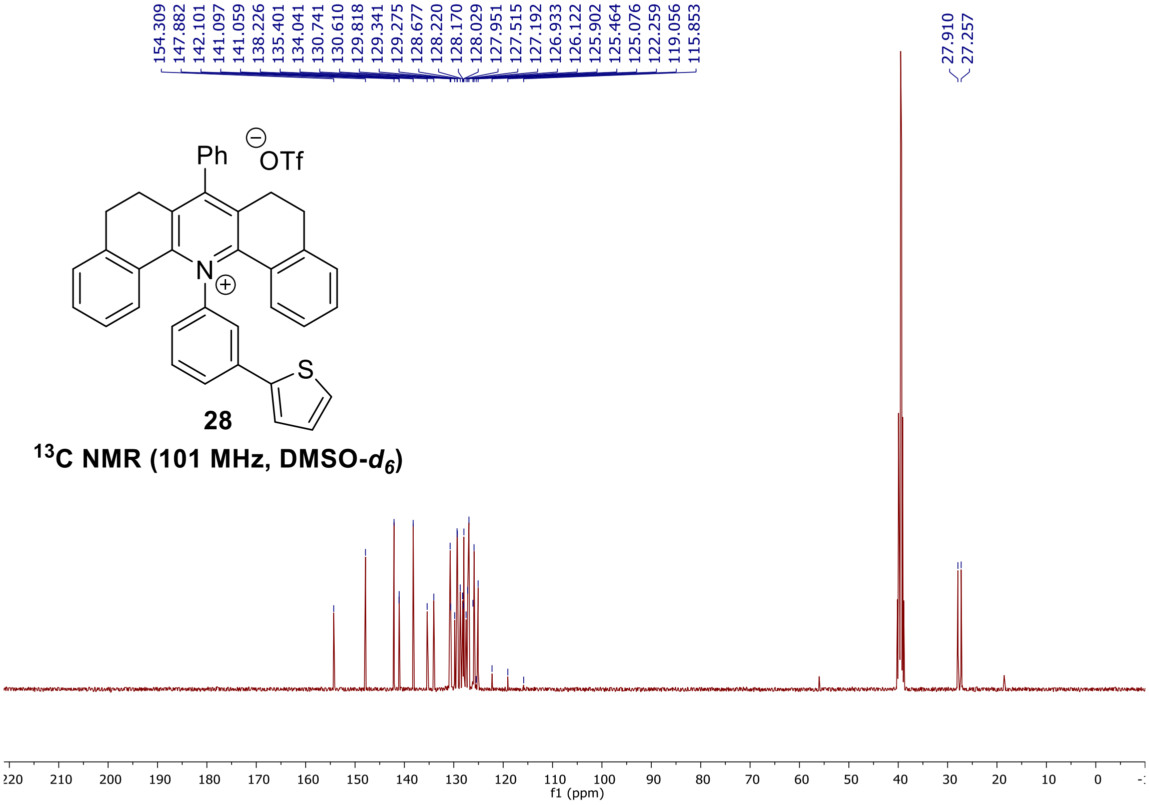
154.309 147.882 142.101 141.097 141.059 138.226 135.401 134.041 130.741 130.610 129.818 129.341 129.275 128.677 128.220 128.170 128.029 127.951 127.515 127.192 126.933 126.122 125.902 125.464 125.076 122.259 119.056 115.853
27.910 27.257
Ph
OTf
N
S
28
^{13}C NMR (101 MHz, DMSO-d_6)
f1 (ppm)

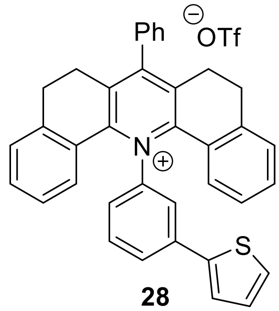

28

^{19}F NMR (470 MHz, DMSO-d_6)

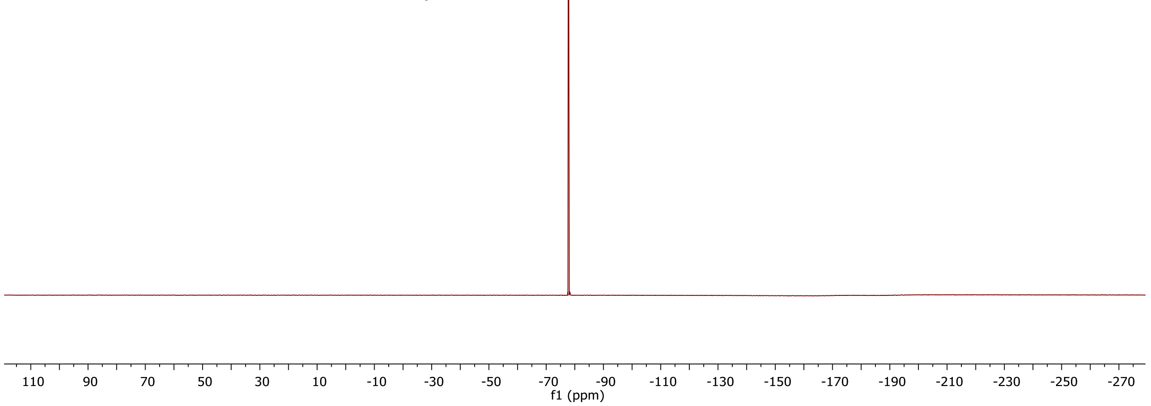

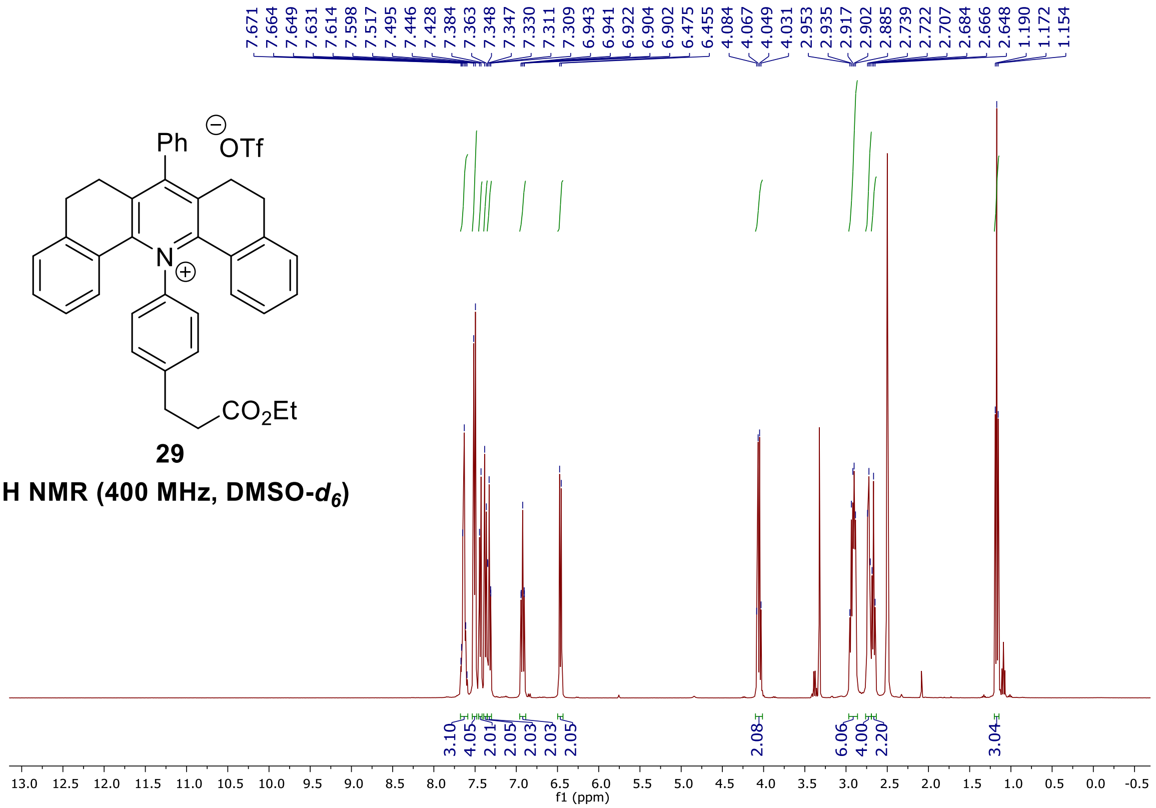

29

^{1}H NMR (400 MHz, DMSO-d_6)

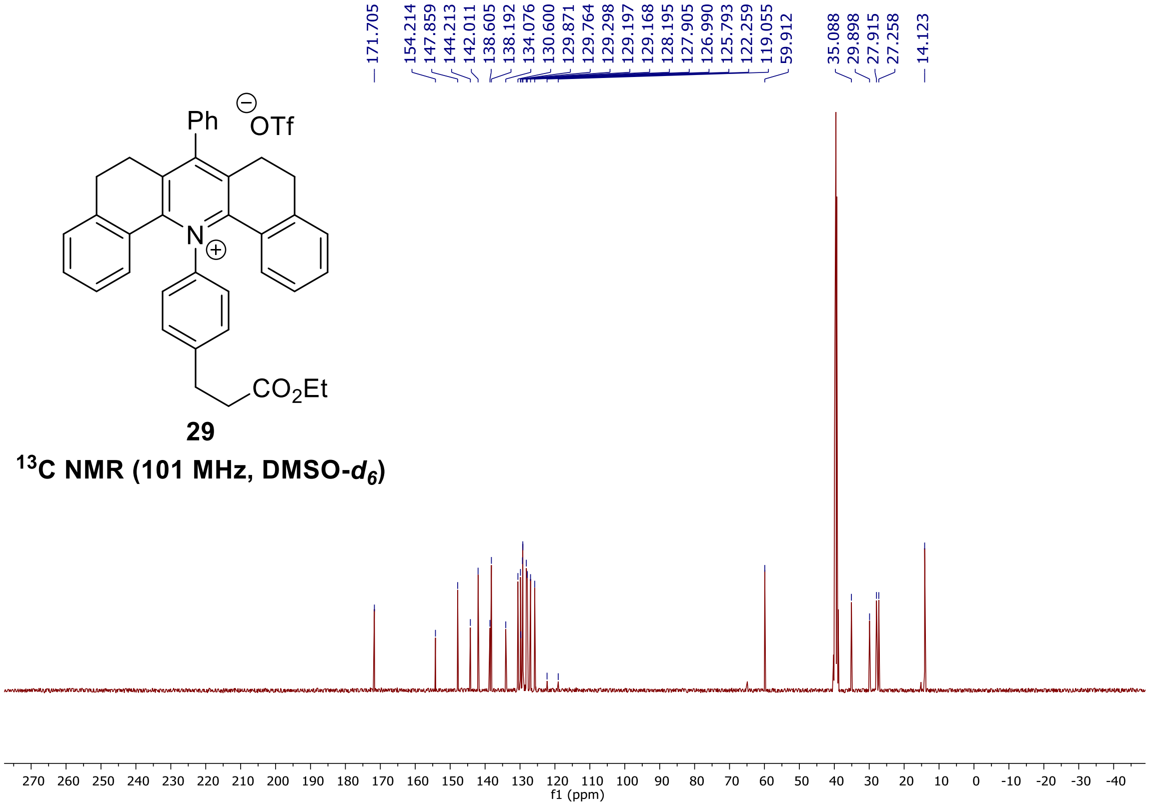

29

^{13}C NMR (101 MHz, DMSO-d_6)

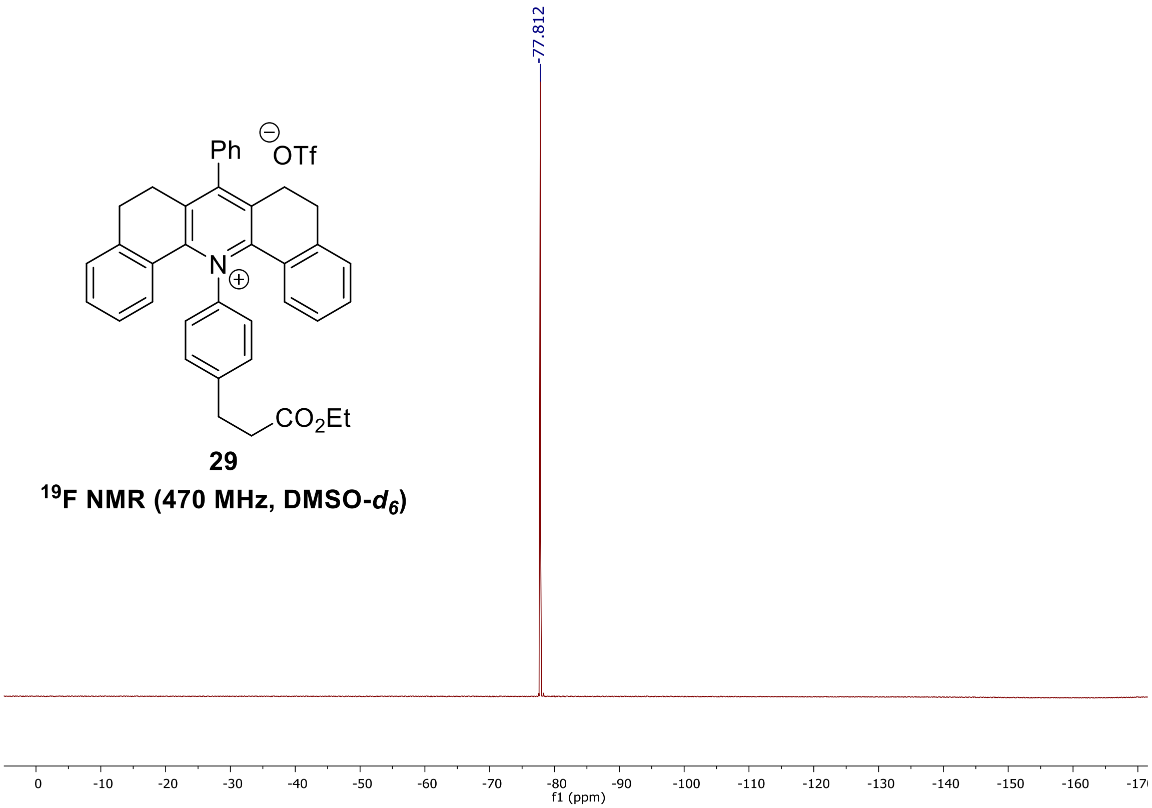

29

^{19}F NMR (470 MHz, DMSO-d_6)

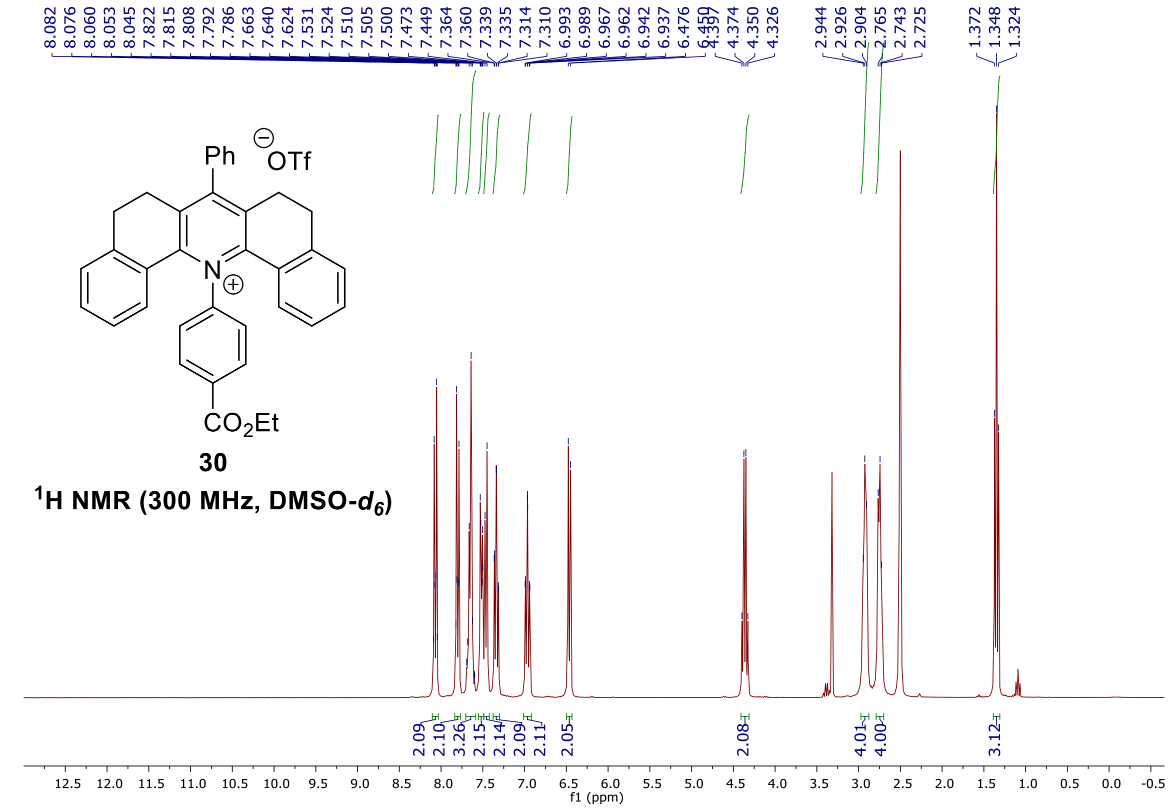
8.082
8.076
8.060
8.053
8.045
7.822
7.815
7.808
7.792
7.786
7.663
7.640
7.624
7.531
7.524
7.510
7.505
7.500
7.473
7.449
7.364
7.360
7.339
7.335
7.314
7.310
6.993
6.989
6.967
6.962
6.942
6.937
6.476
6.450
4.397
4.374
4.350
4.326
2.944
2.926
2.904
2.765
2.743
2.725
1.372
1.348
1.324
Ph
OTf
N
CO2Et
30
1H NMR (300 MHz, DMSO-d6)
2.09
2.10
3.26
2.15
2.14
2.09
2.11
2.05
2.08
4.01
4.00
3.12
12.5 12.0 11.5 11.0 10.5 10.0 9.5 9.0 8.5 8.0 7.5 7.0 6.5 6.0 5.5 5.0 4.5 4.0 3.5 3.0 2.5 2.0 1.5 1.0 0.5 0.0 -0.5
f1 (ppm)

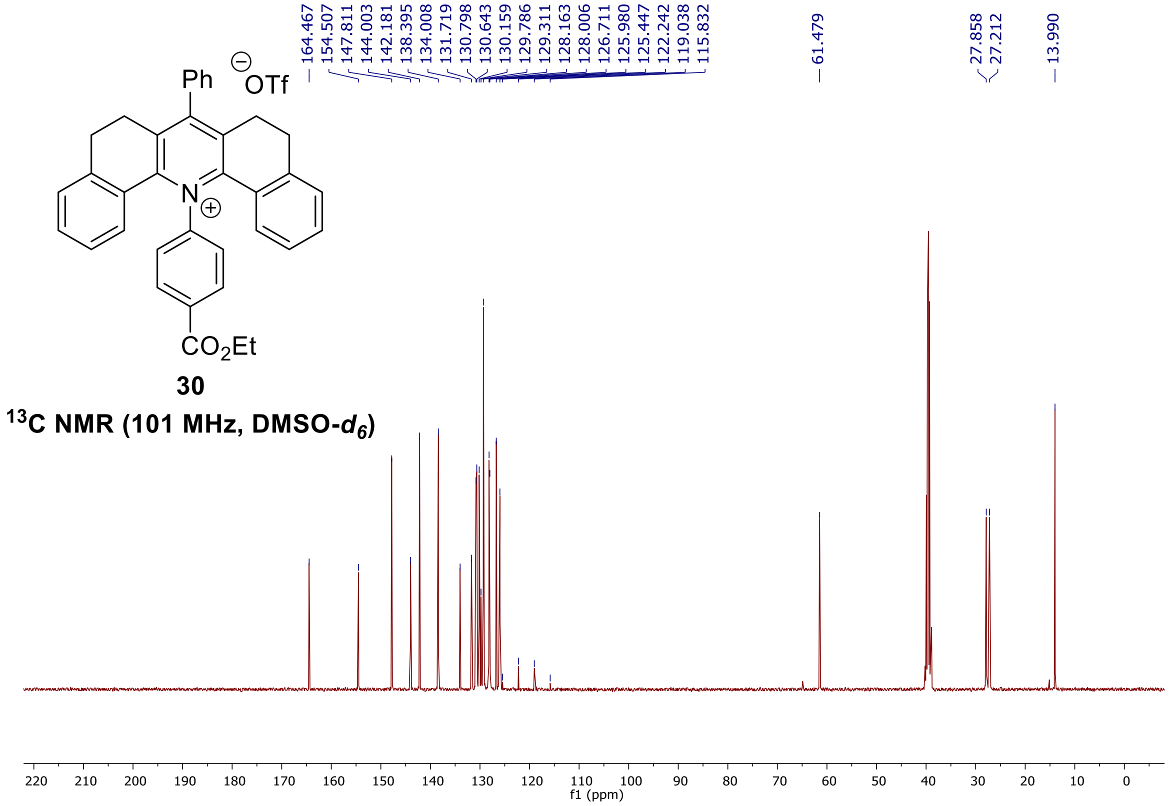
164.467
154.507
147.811
144.003
142.181
138.395
134.008
131.719
130.798
130.643
130.159
129.786
129.311
128.163
128.006
126.711
125.980
125.447
122.242
119.038
115.832
61.479
27.858
27.212
13.990
Ph
OTf
N
CO2Et
30
13C NMR (101 MHz, DMSO-d6)
220 210 200 190 180 170 160 150 140 130 120 110 100 90 80 70 60 50 40 30 20 10 0
f1 (ppm)

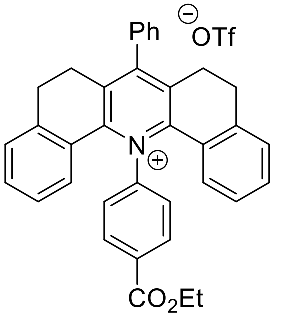

30

^{19}F NMR (470 MHz, DMSO-d_6)

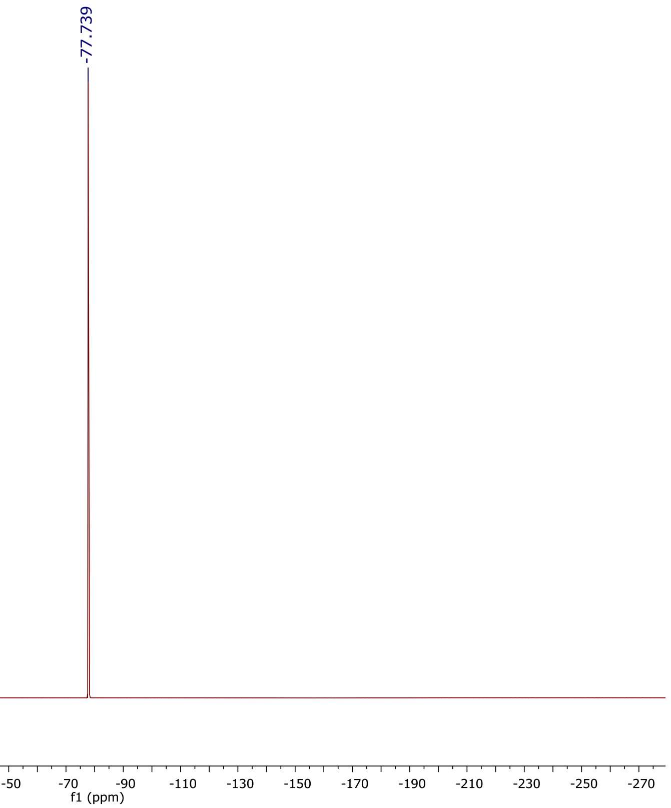

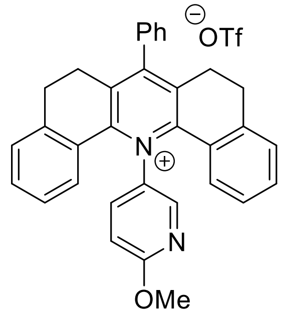

31

^{1}H NMR (400 MHz, DMSO-d_6)

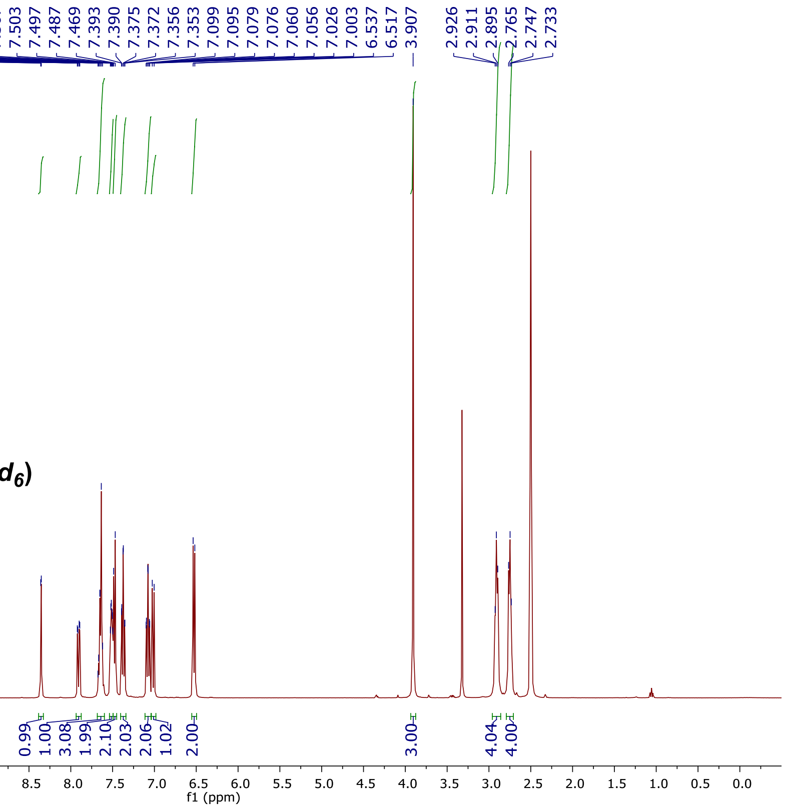

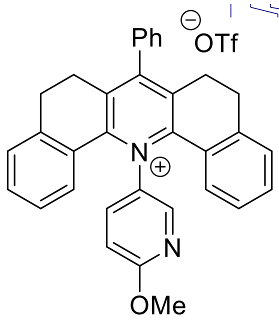

31

^{13}C NMR (101 MHz, DMSO-d_6)

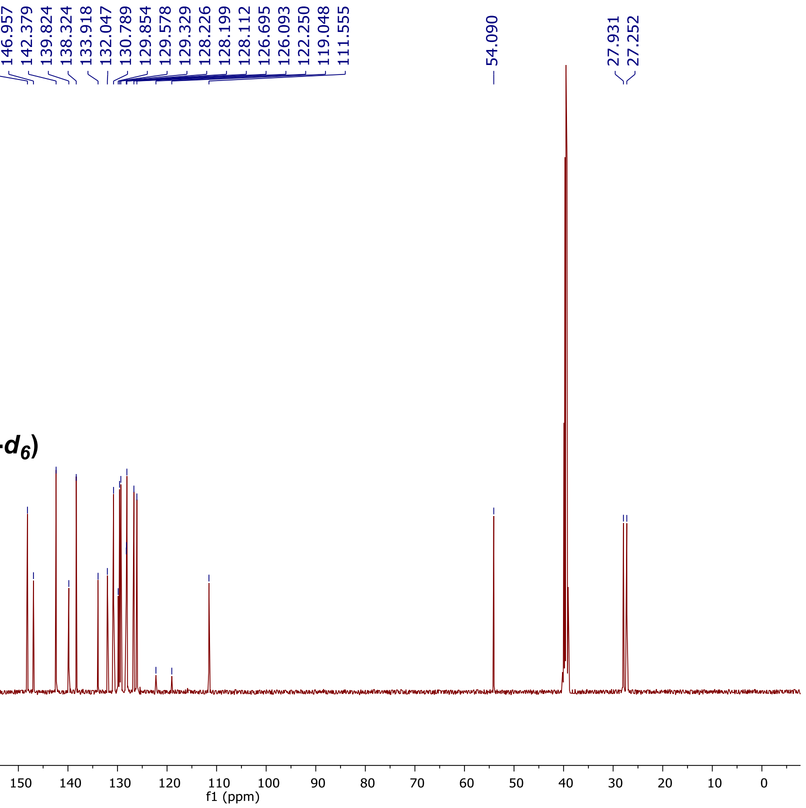

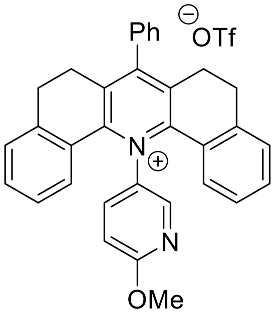

31

^{19}F NMR (470 MHz, DMSO-*d*$_6$)

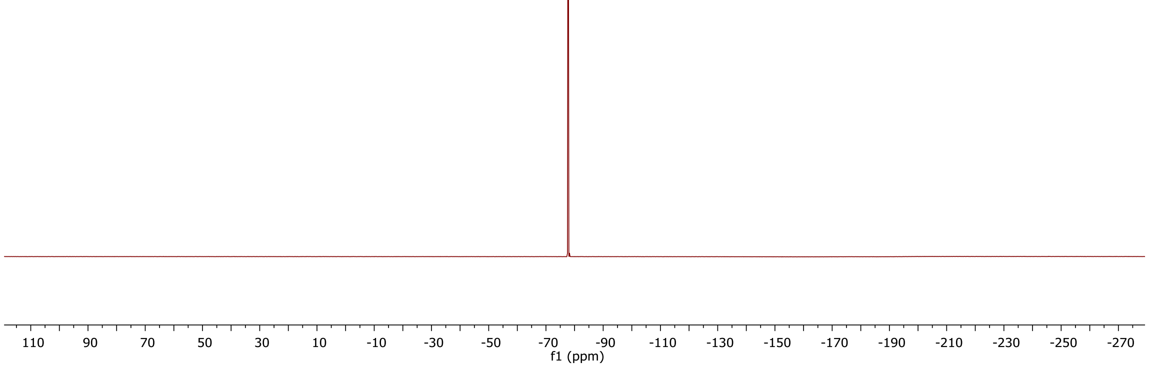

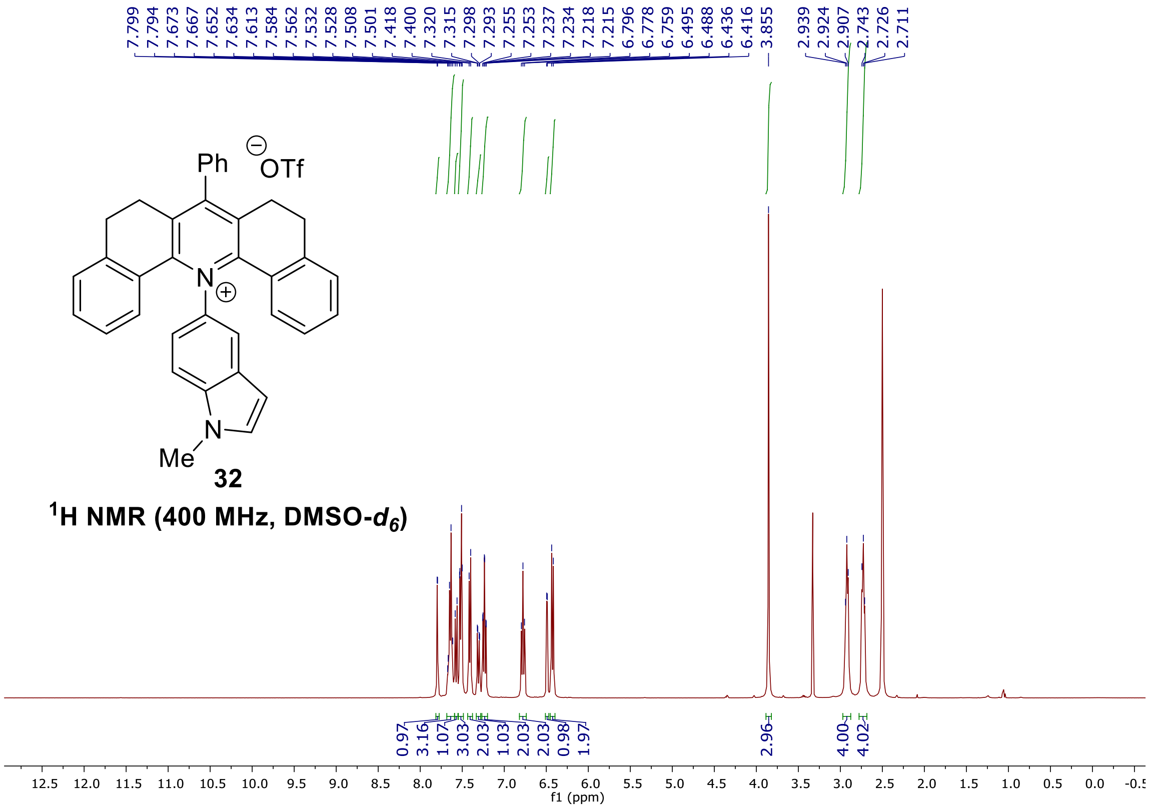
7.799 7.794 7.673 7.667 7.652 7.634 7.613 7.584 7.562 7.532 7.528 7.508 7.501 7.418 7.400 7.320 7.315 7.298 7.293 7.255 7.253 7.237 7.234 7.218 7.215 6.796 6.778 6.759 6.495 6.488 6.436 6.416 3.855 2.939 2.924 2.907 2.743 2.726 2.711
Ph
OTf
N
Me
32
1H NMR (400 MHz, DMSO-d6)
0.97 3.16 1.07 3.03 2.03 1.03 2.03 2.03 0.98 1.97 2.96 4.00 4.02
f1 (ppm)

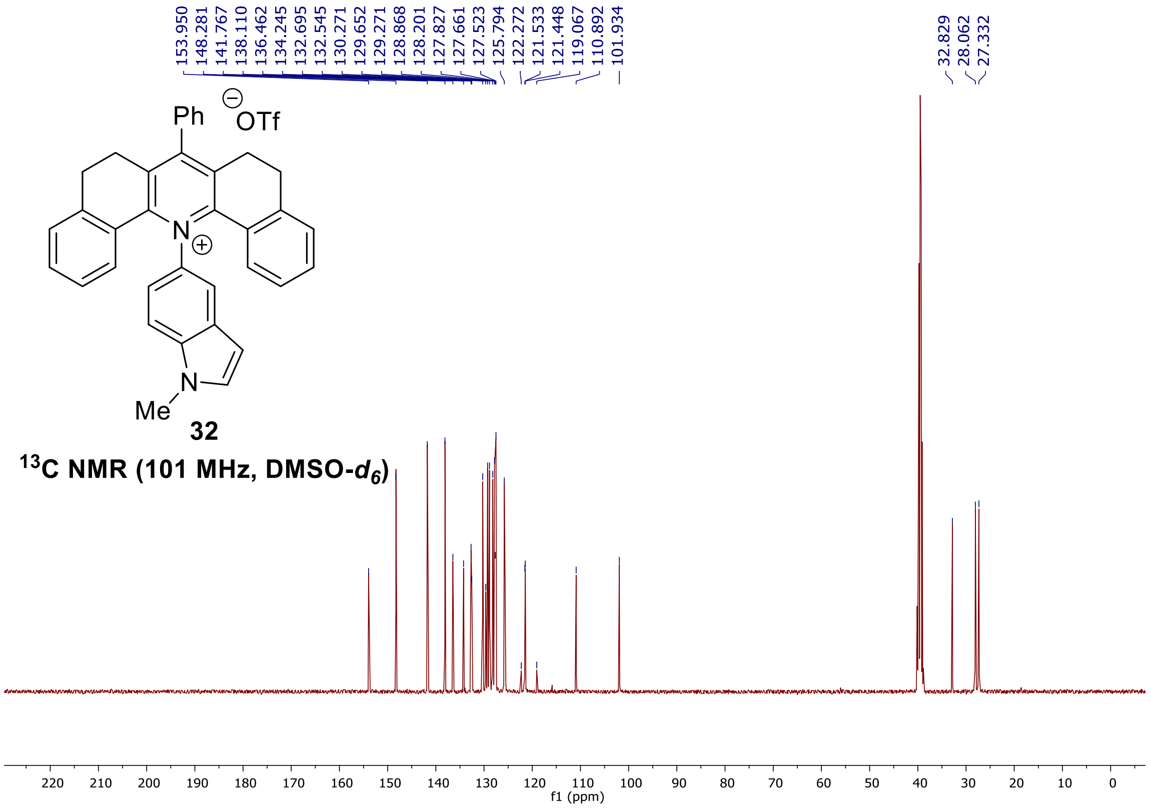
153.950 148.281 141.767 138.110 136.462 134.245 132.695 132.545 130.271 129.652 129.271 128.868 128.201 127.827 127.661 127.523 125.794 122.272 121.533 121.448 119.067 110.892 101.934
32.829 28.062 27.332
Ph
OTf
N
Me
32
13C NMR (101 MHz, DMSO-d6)
f1 (ppm)

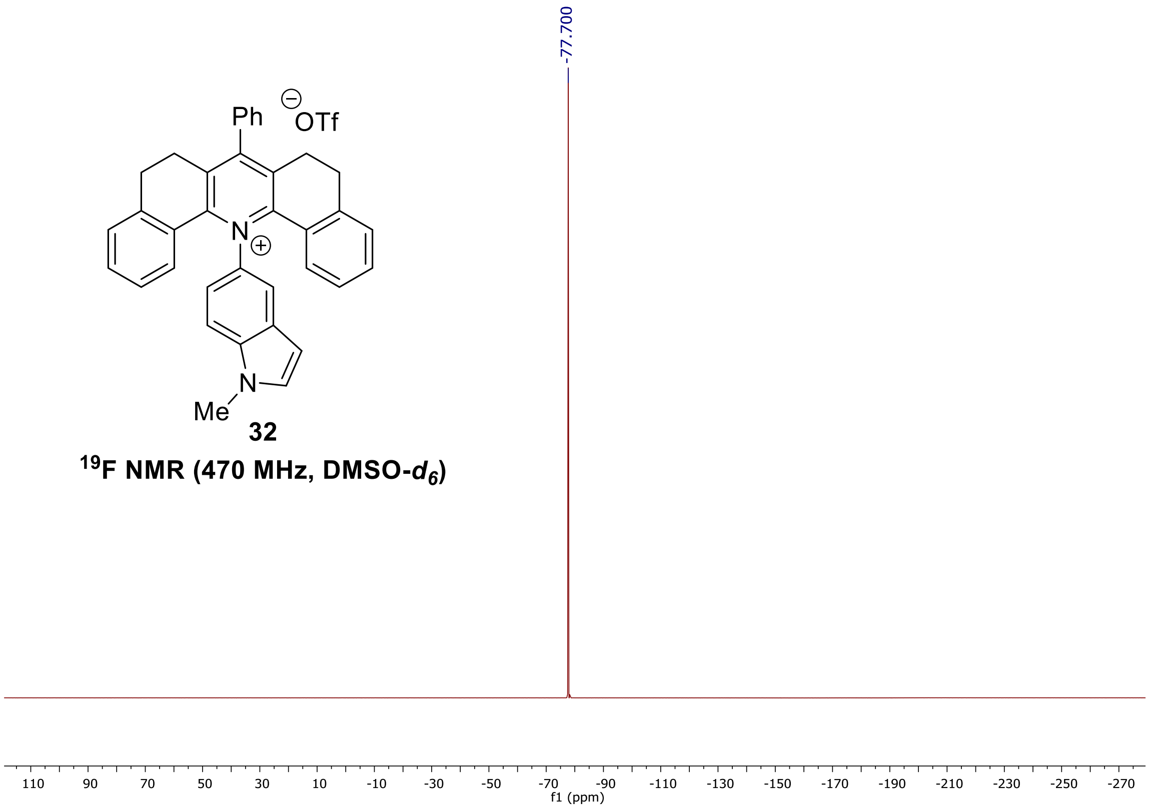

32

^{19}F NMR (470 MHz, DMSO-d_6)

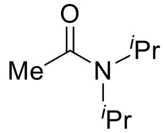

***N*,*N*-diisopropylacetamide**

^{1}H NMR (300 MHz, $CDCl_3$)

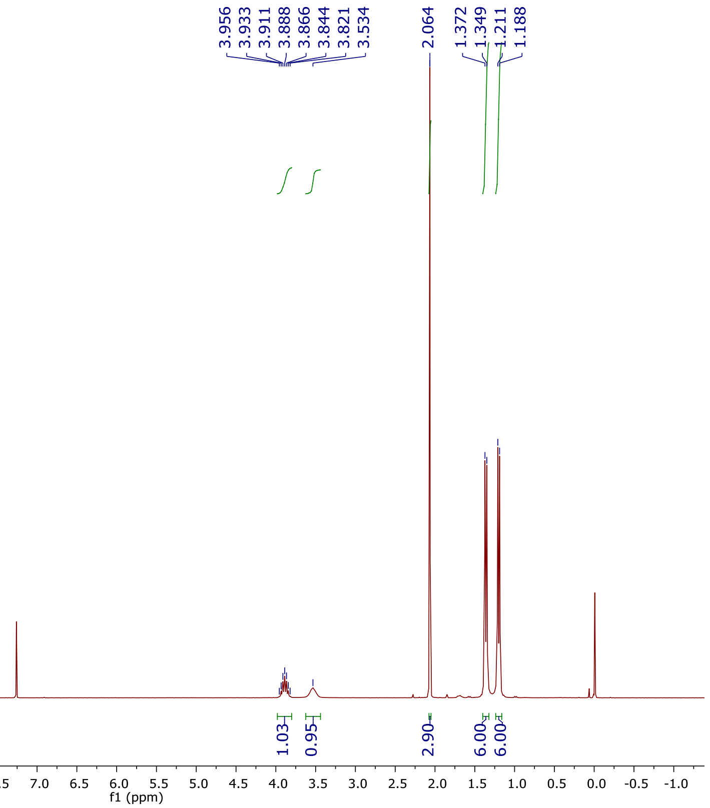

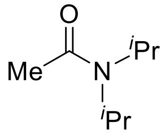

***N*,*N*-diisopropylacetamide**

^{13}C NMR (101 MHz, $CDCl_3$)

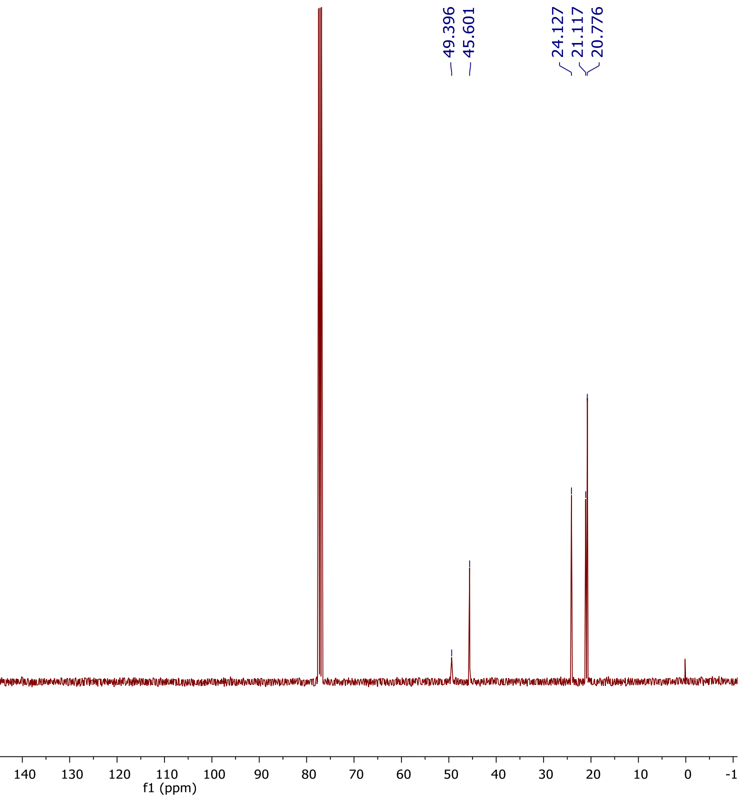

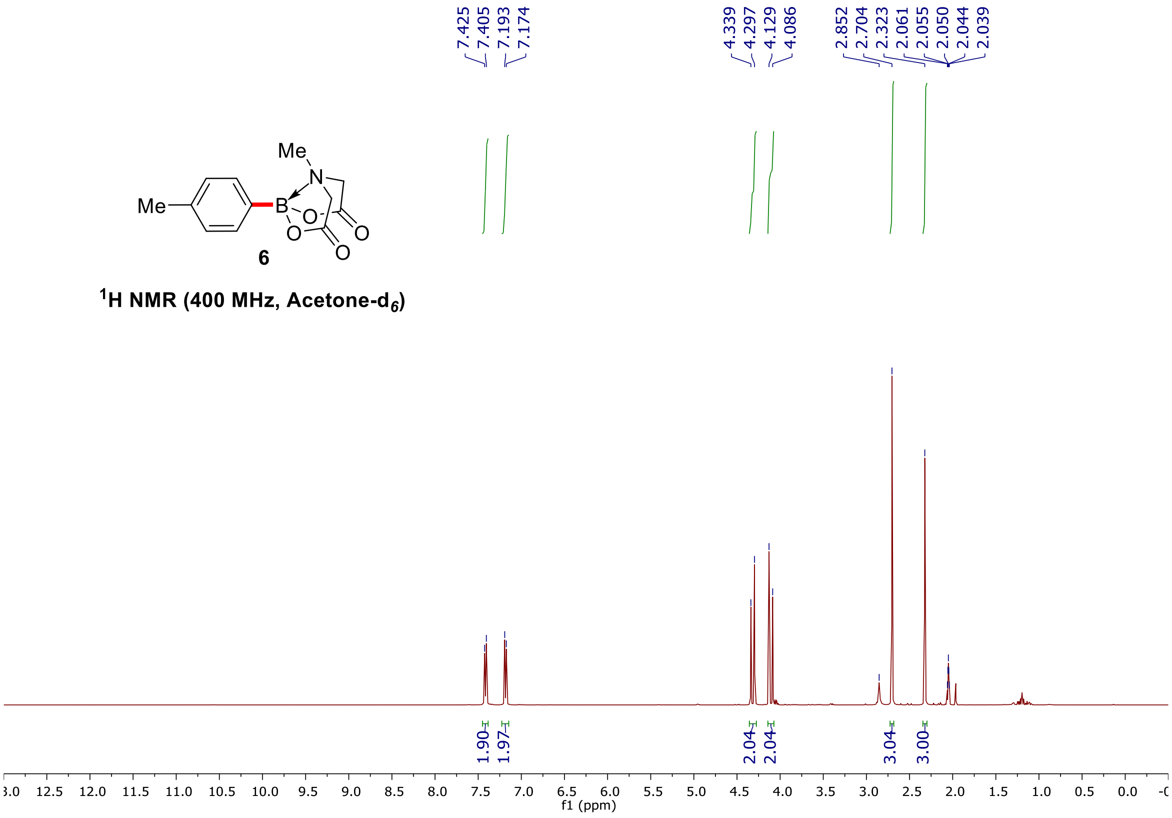
7.425
7.405
7.193
7.174
4.339
4.297
4.129
4.086
2.852
2.704
2.323
2.061
2.055
2.050
2.044
2.039
Me
N
Me
B
O
O
O
O
6
1H NMR (400 MHz, Acetone-d6)
1.90
1.97
2.04
2.04
3.04
3.00
f1 (ppm)

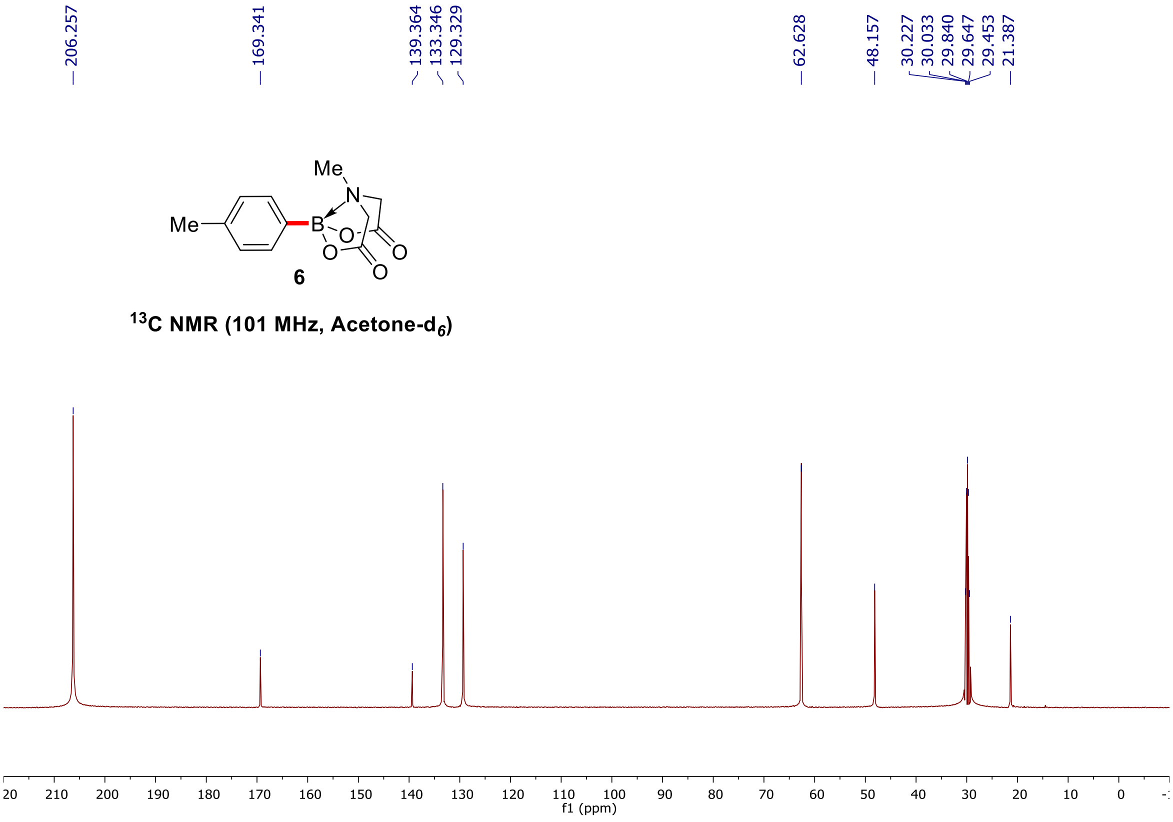
206.257
169.341
139.364
133.346
129.329
62.628
48.157
30.227
30.033
29.840
29.647
29.453
21.387
Me
N
Me
B
O
O
O
O
6
13C NMR (101 MHz, Acetone-d6)
f1 (ppm)

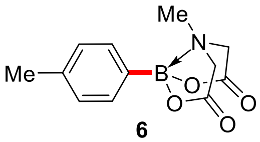

6

^{11}B NMR (128 MHz, Acetone-d$_6$)

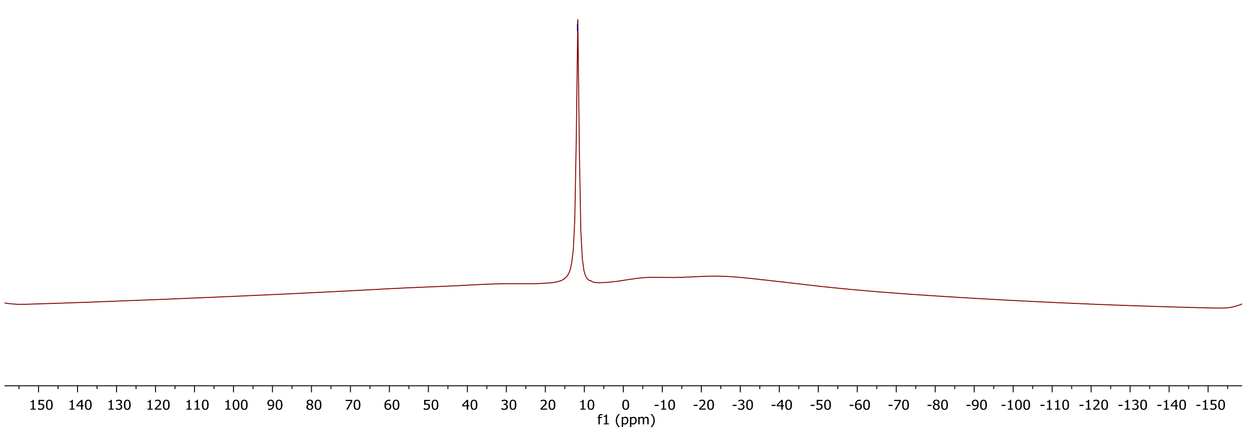

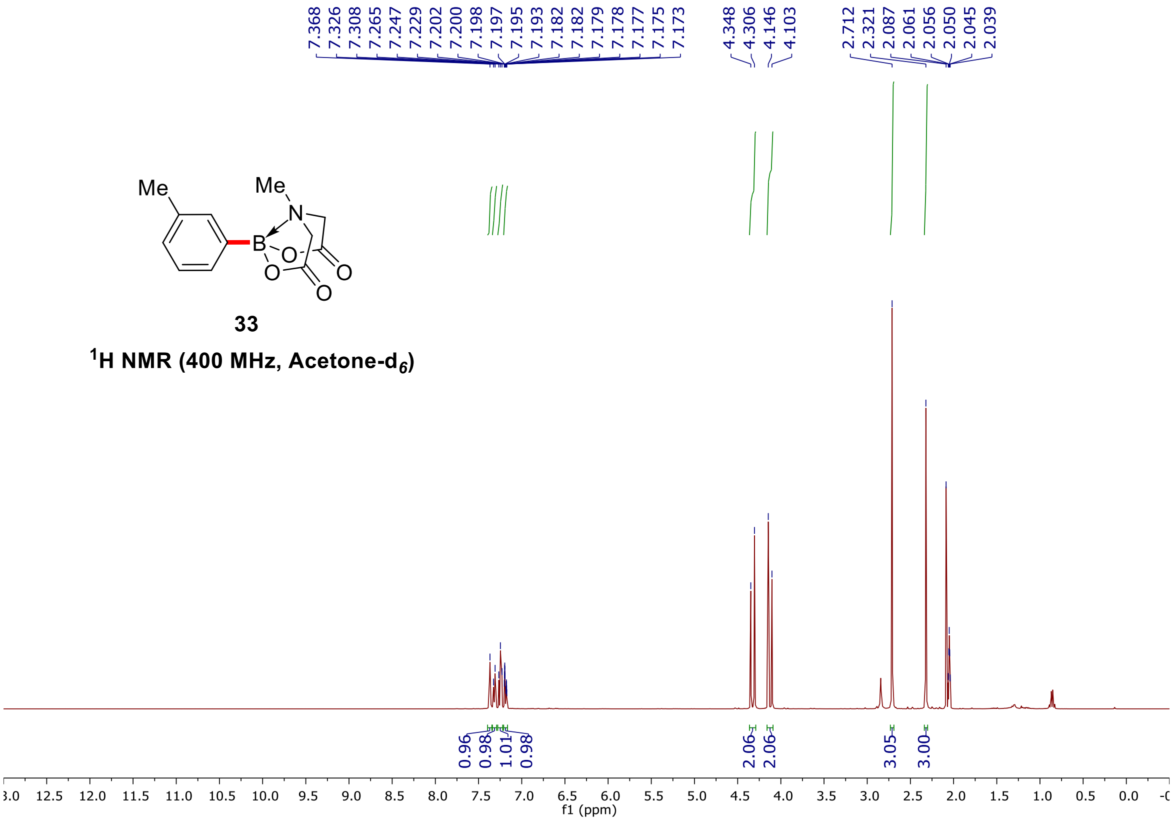

33

^{1}H NMR (400 MHz, Acetone-d_6)

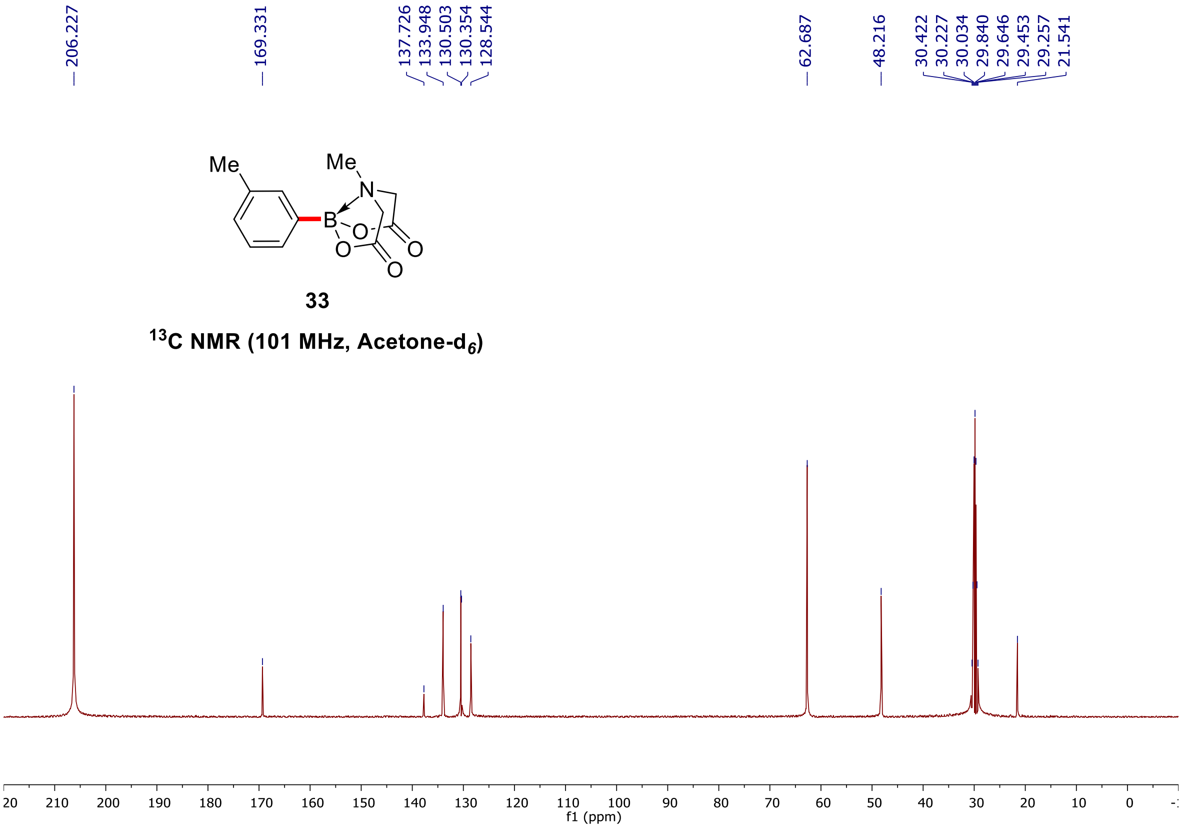

33

^{13}C NMR (101 MHz, Acetone-d_6)

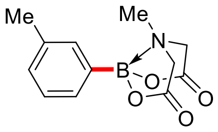

33

^{11}B NMR (128 MHz, Acetone-d$_6$)

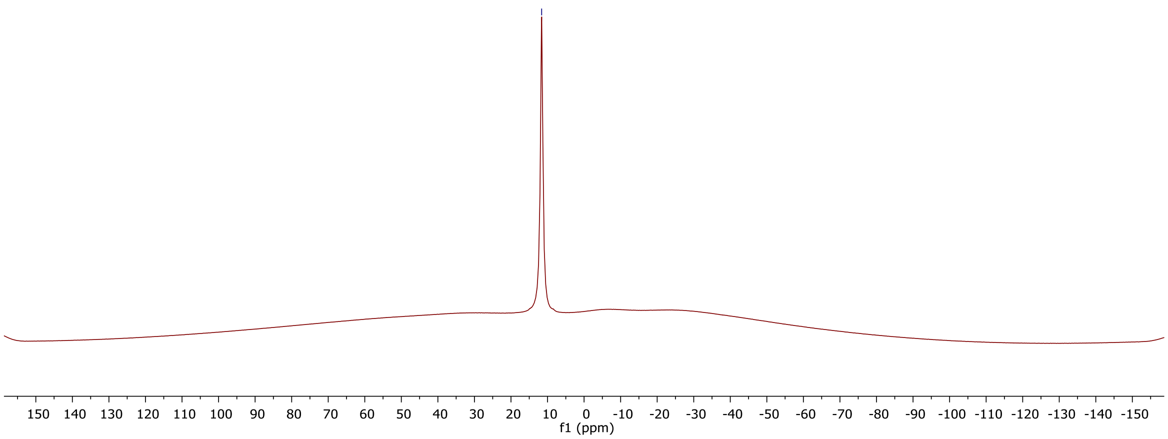

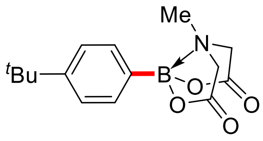

34

^{1}H NMR (400 MHz, Acetone-d_6)

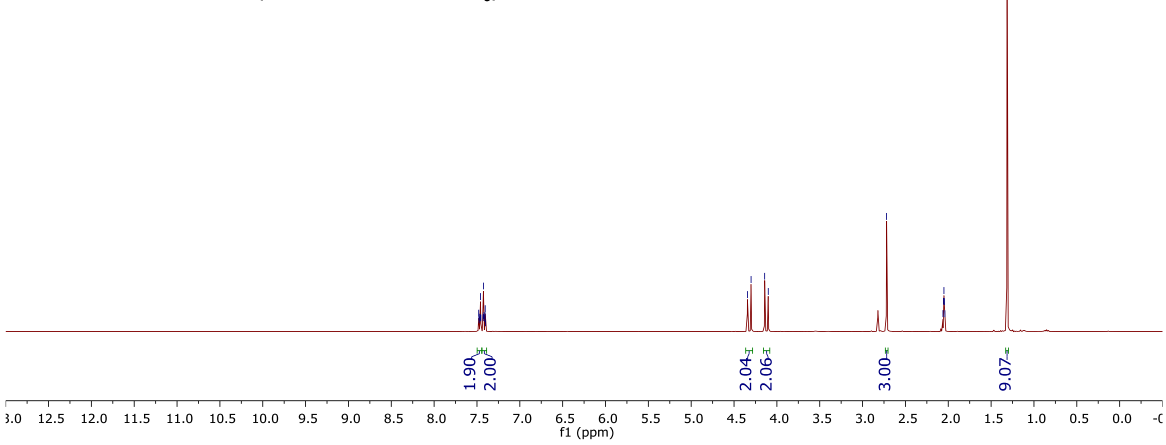

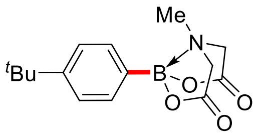

34

^{13}C NMR (101 MHz, Acetone-d_6)

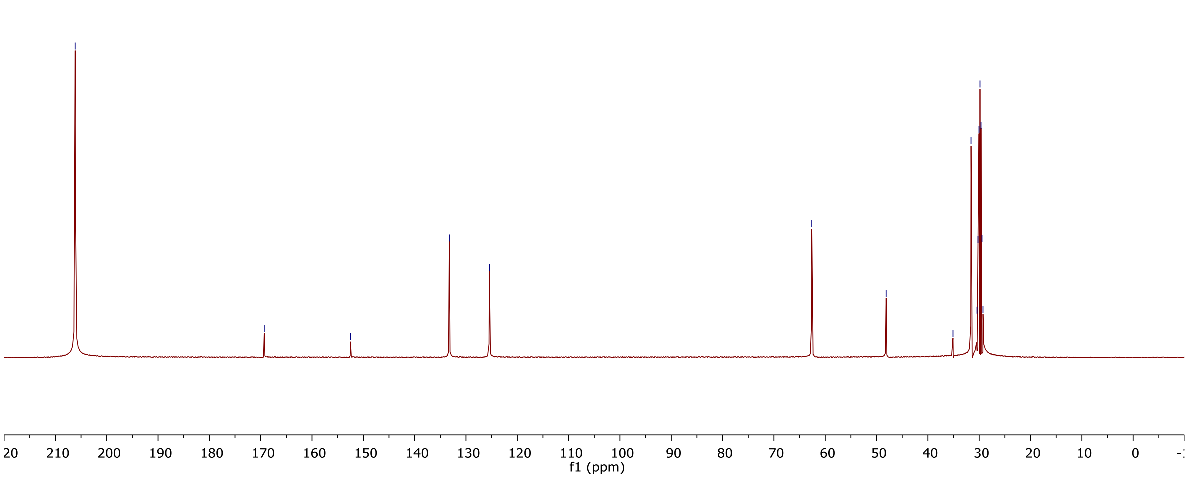

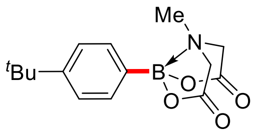

34

^{11}B NMR (128 MHz, Acetone-d$_6$)

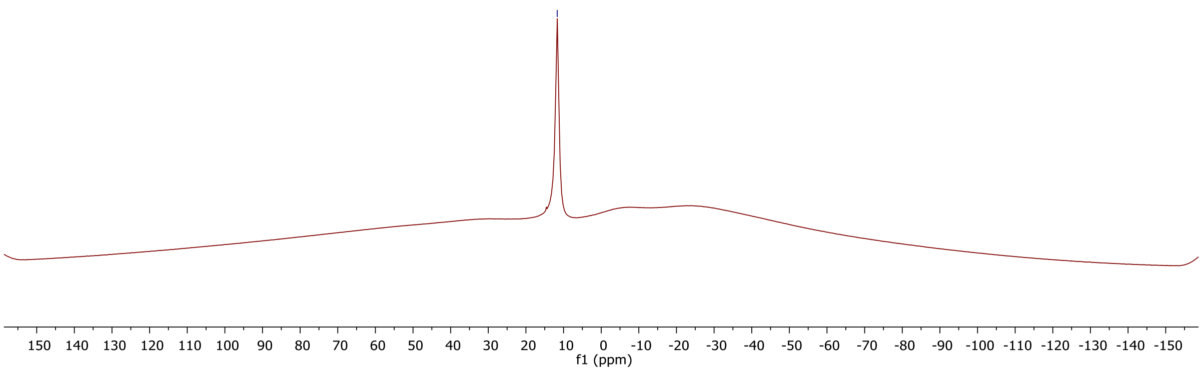

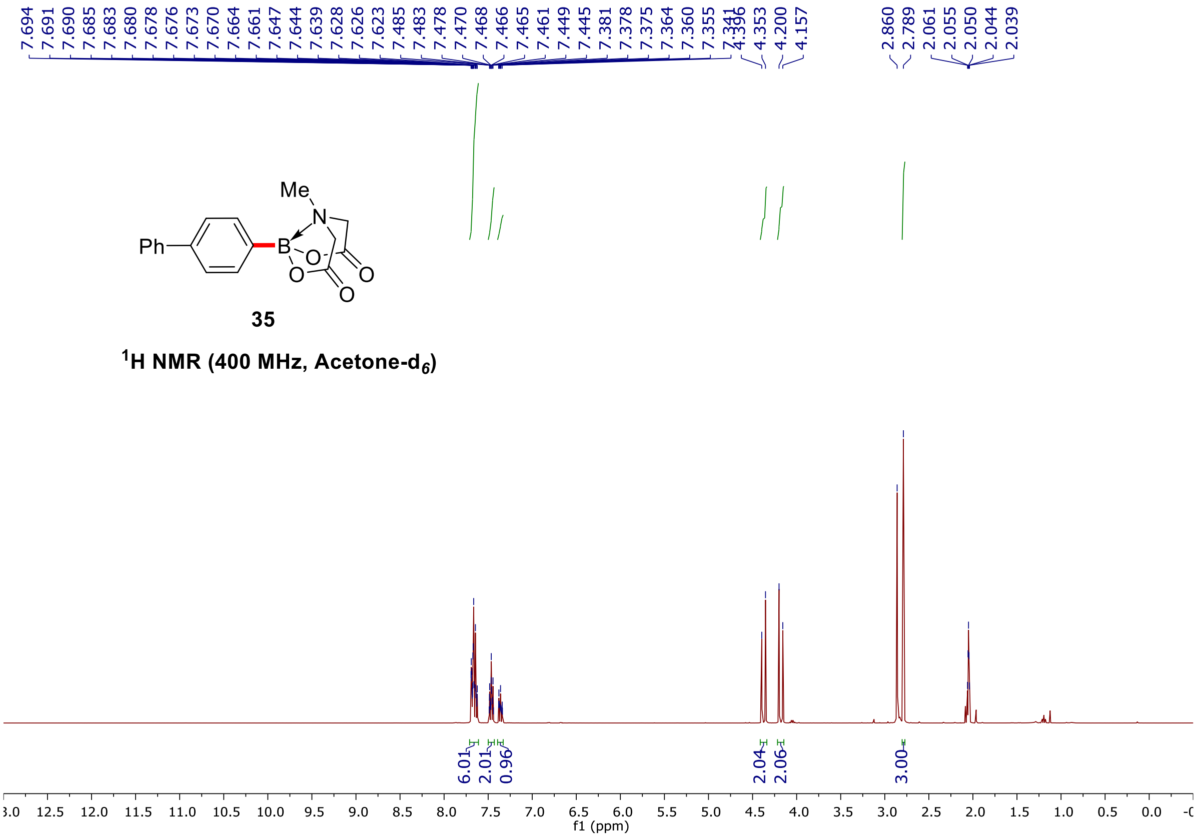
7.694 7.691 7.690 7.685 7.683 7.680 7.678 7.676 7.673 7.670 7.664 7.661 7.647 7.644 7.639 7.628 7.626 7.623 7.485 7.483 7.478 7.470 7.468 7.466 7.465 7.461 7.449 7.445 7.381 7.378 7.375 7.364 7.360 7.355 7.341 4.396 4.353 4.200 4.157
2.860 2.789 2.061 2.055 2.050 2.044 2.039
Me
N
Ph
B
O
O
O
O
35
1H NMR (400 MHz, Acetone-d6)
6.01
2.01
0.96
2.04
2.06
3.00
f1 (ppm)

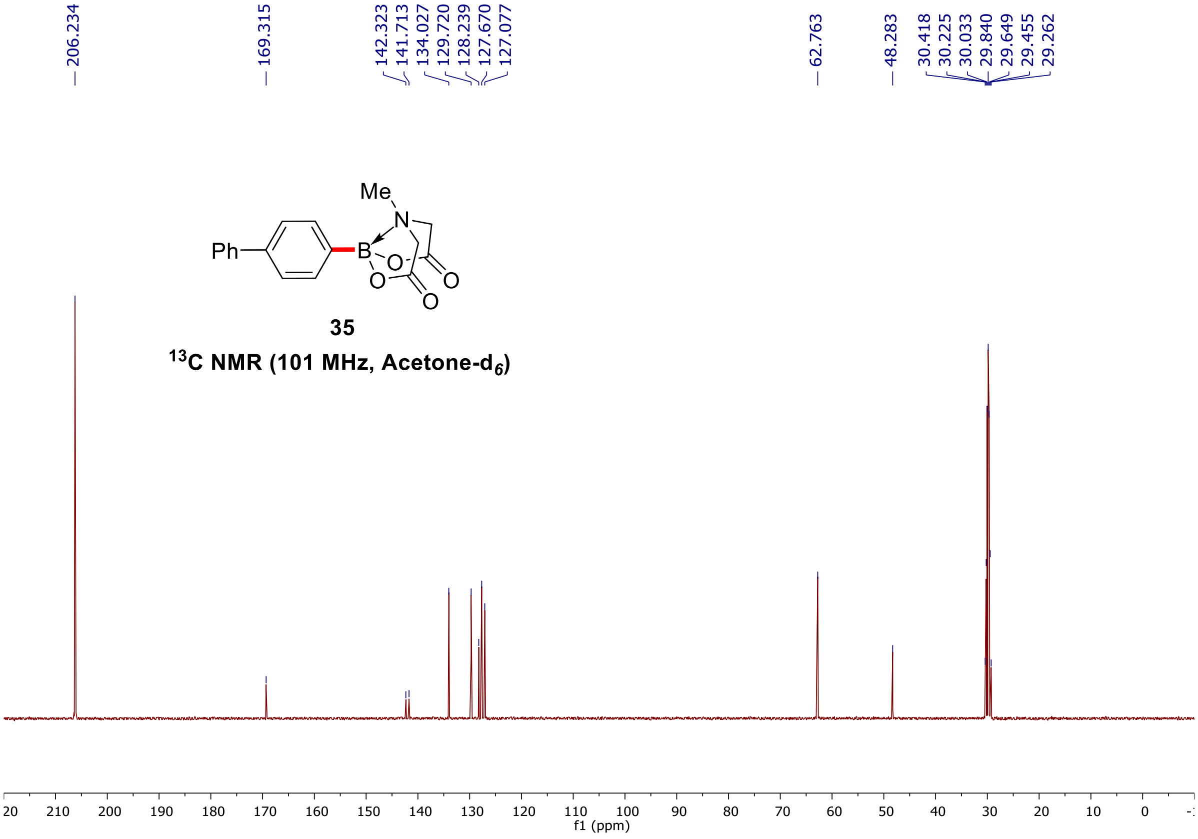
206.234
169.315
142.323 141.713 134.027 129.720 128.239 127.670 127.077
62.763
48.283
30.418 30.225 30.033 29.840 29.649 29.455 29.262
Me
N
Ph
B
O
O
O
O
35
13C NMR (101 MHz, Acetone-d6)
f1 (ppm)

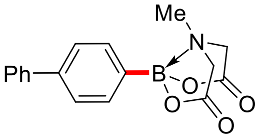

35

^{11}B NMR (128 MHz, Acetone-d_6)

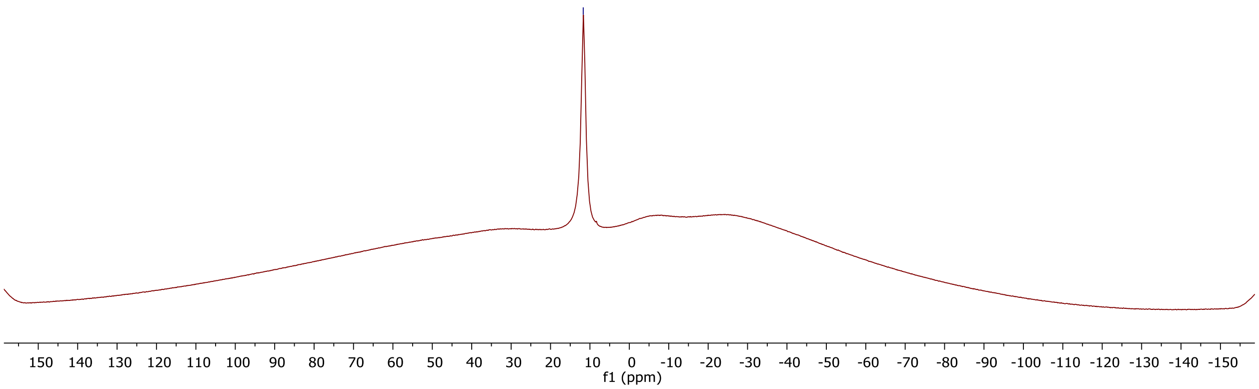

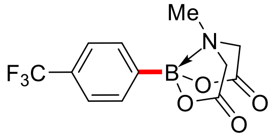

36

^{1}H NMR (400 MHz, Acetone-d$_6$)

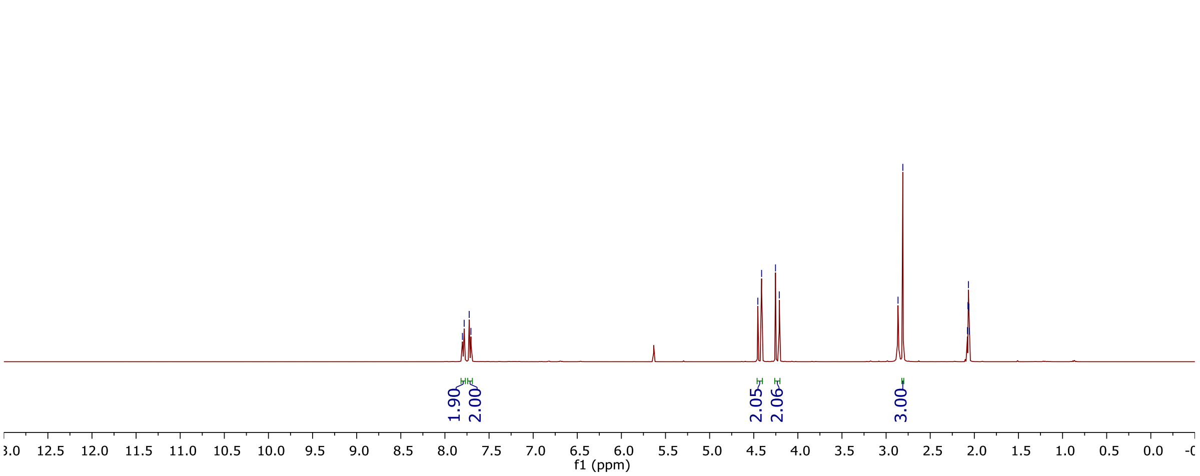

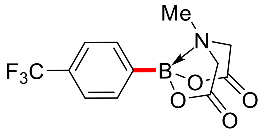

36

^{13}C NMR (101 MHz, Acetone-d$_6$)

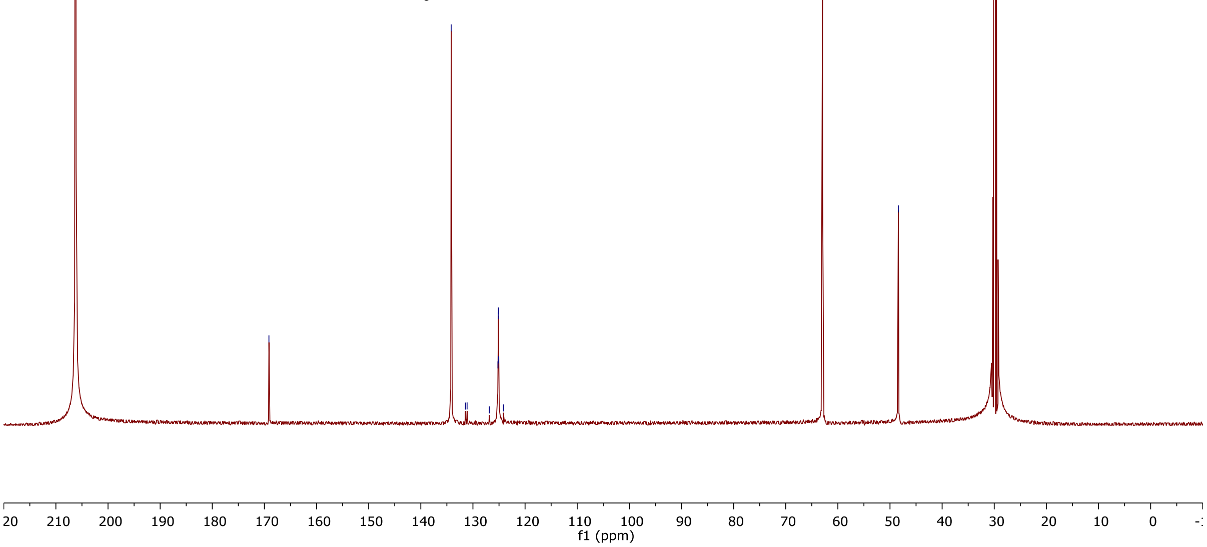

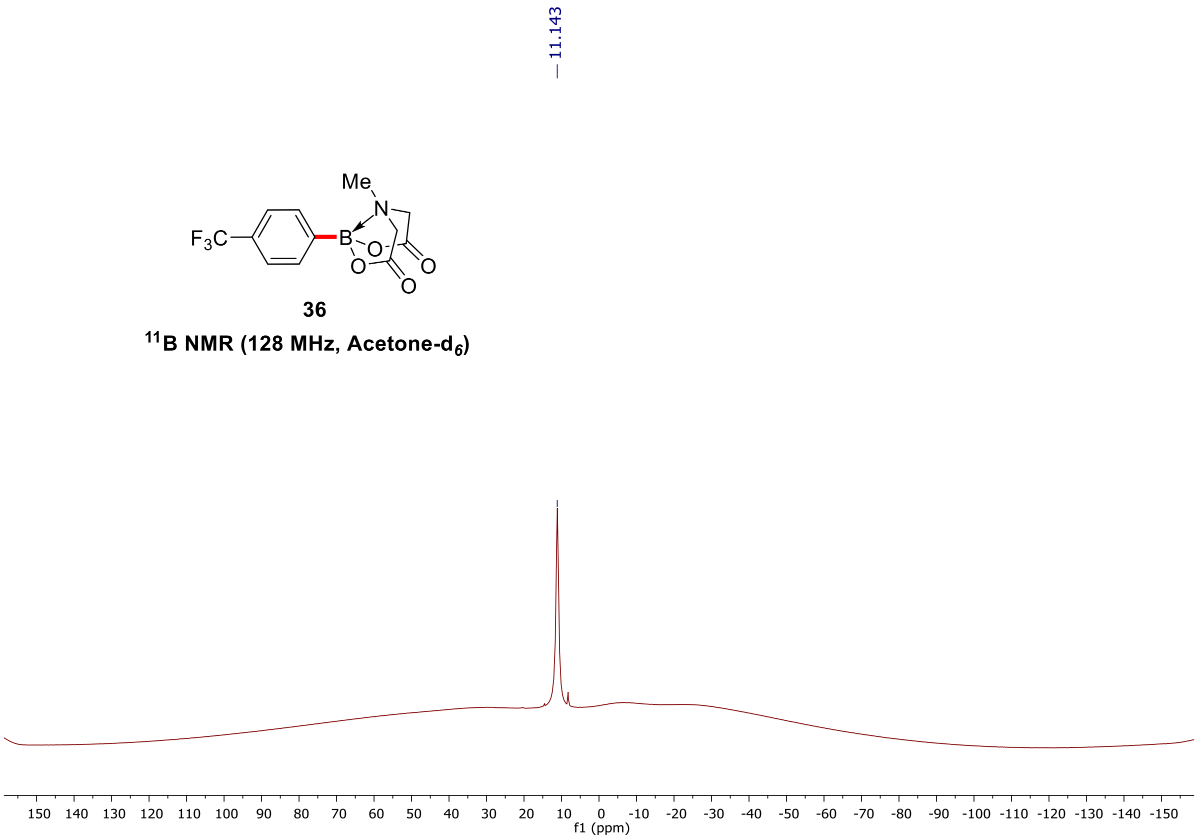

36

^{11}B NMR (128 MHz, Acetone-d_6)

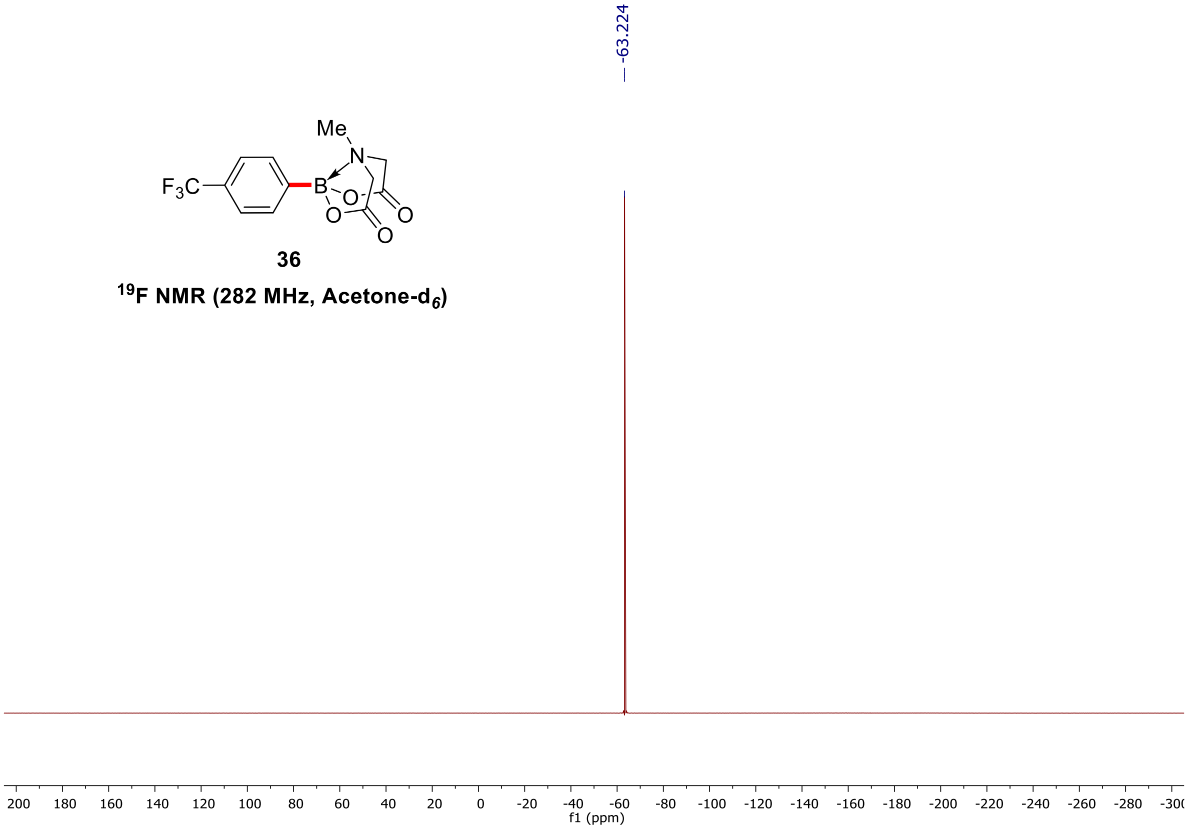

36

^{19}F NMR (282 MHz, Acetone-d_6)

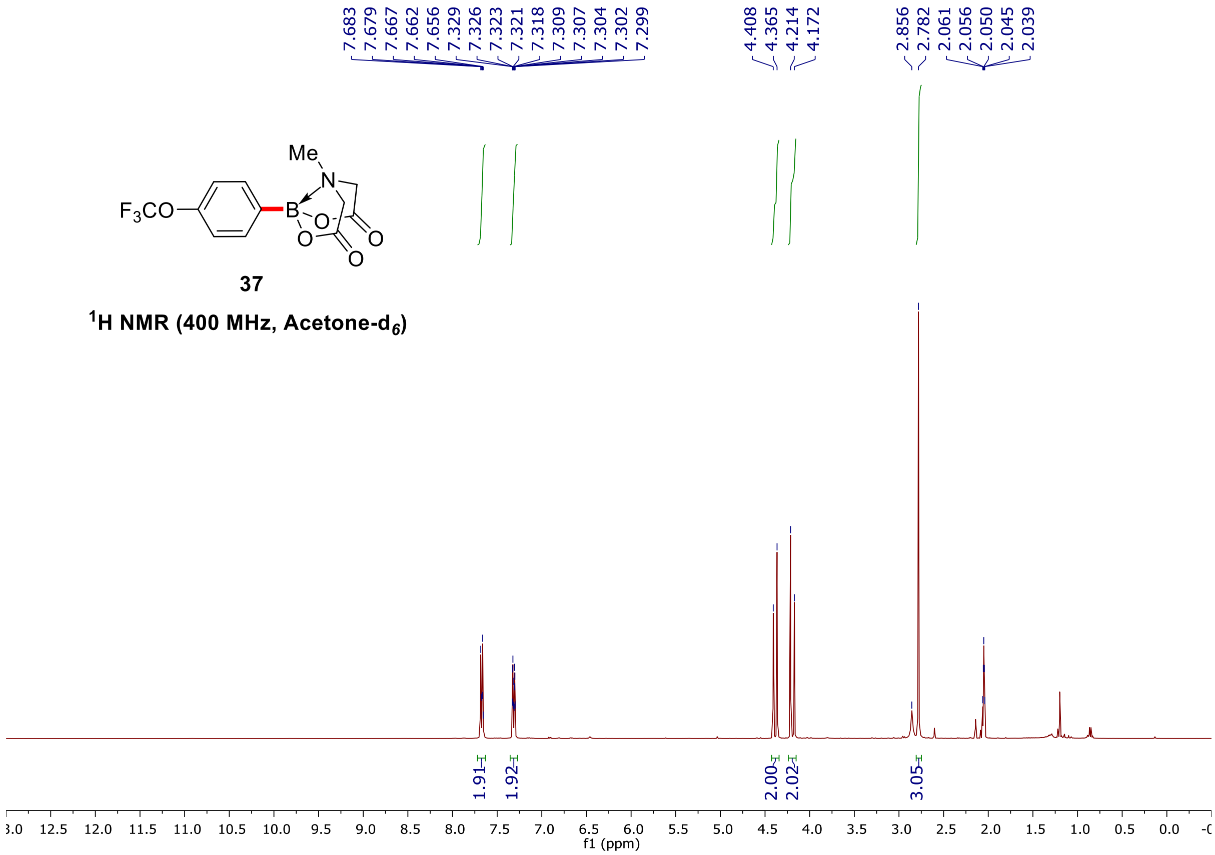
7.683 7.679 7.667 7.662 7.656 7.329 7.326 7.323 7.321 7.318 7.309 7.307 7.304 7.302 7.299
4.408 4.365 4.214 4.172
2.856 2.782 2.061 2.056 2.050 2.045 2.039
Me
N
F_3CO
B
O
O
O
O
37
^{1}H NMR (400 MHz, Acetone-d$_6$)
1.91
1.92
2.00
2.02
3.05
f1 (ppm)

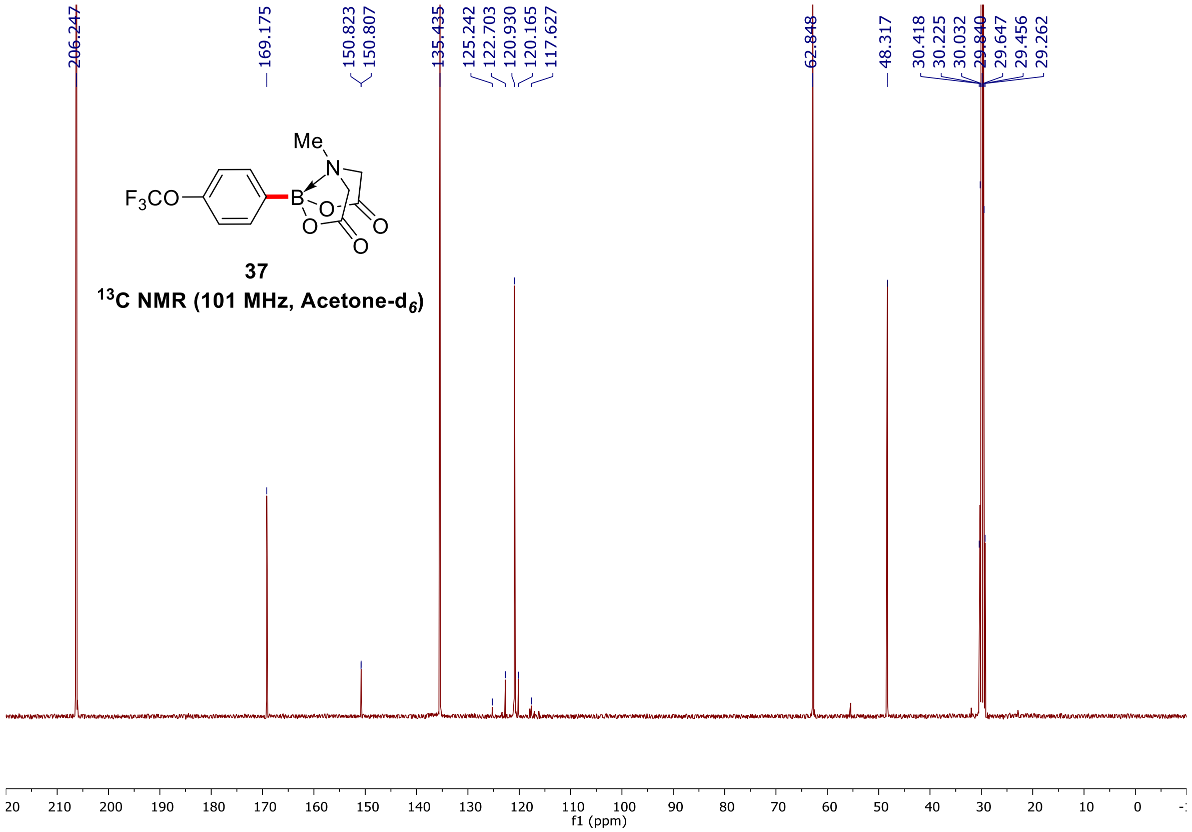
206.247
169.175
150.823 150.807
135.435
125.242 122.703 120.930 120.165 117.627
62.848
48.317
30.418 30.225 30.032 29.840 29.647 29.456 29.262
Me
N
F_3CO
B
O
O
O
O
37
^{13}C NMR (101 MHz, Acetone-d$_6$)
f1 (ppm)

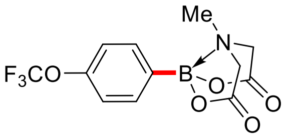

37

^{11}B NMR (128 MHz, Acetone-d_6)

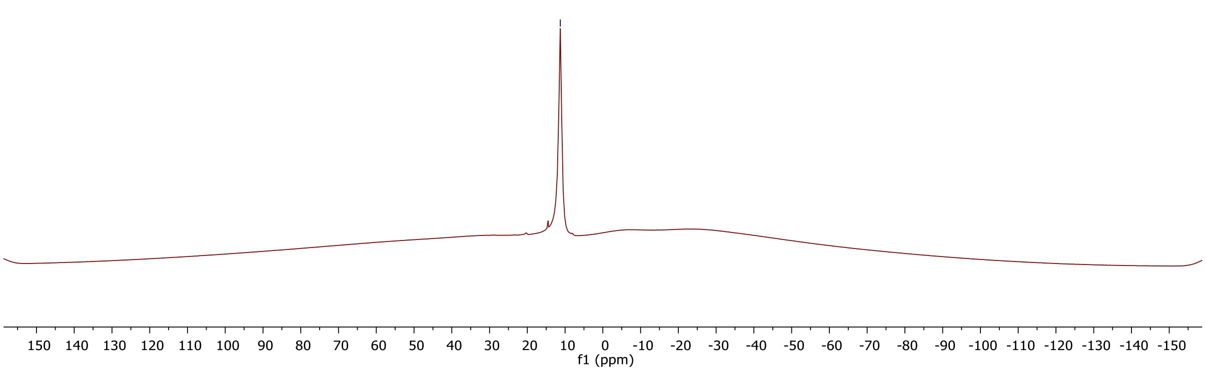

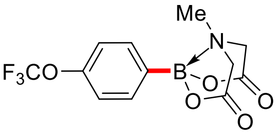

37

^{19}F NMR (471 MHz, Acetone-d_6)

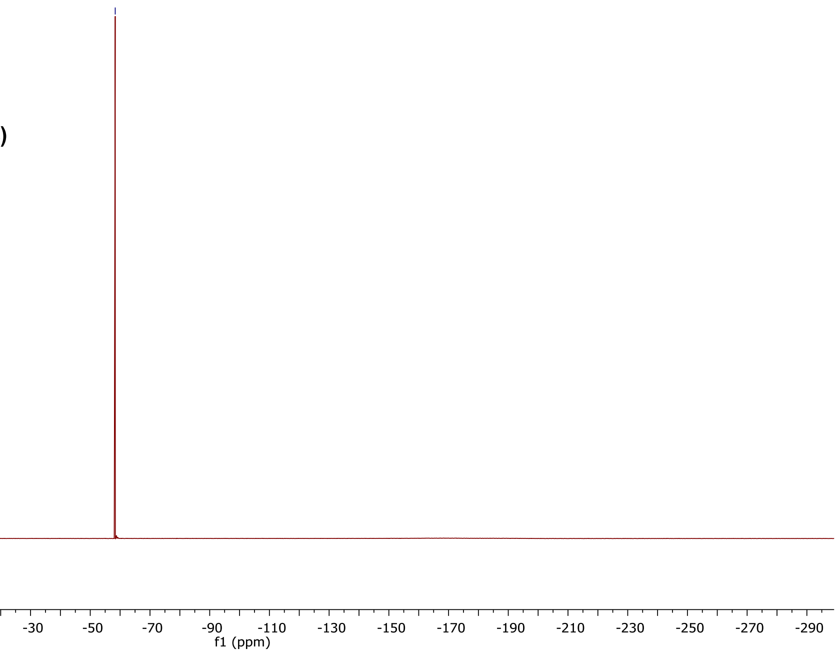

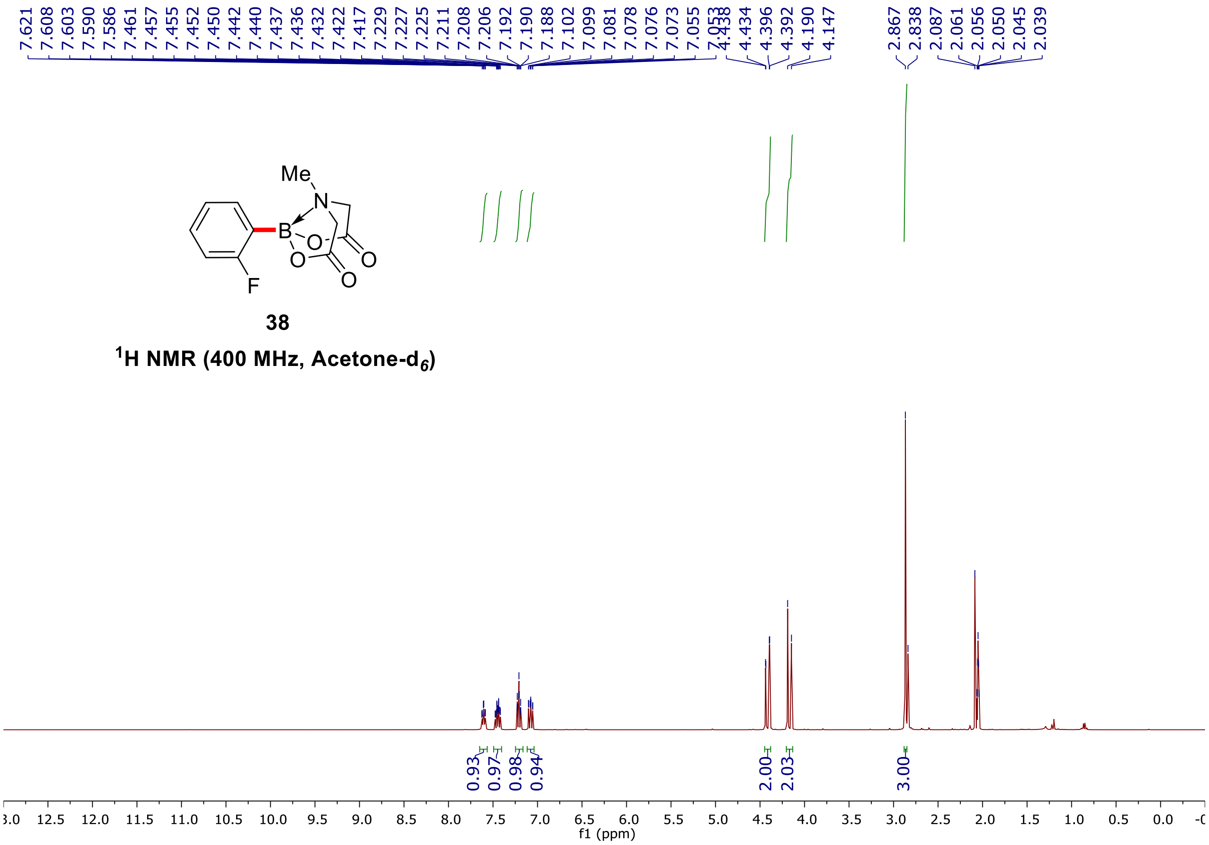

38

^{1}H NMR (400 MHz, Acetone-d_6)

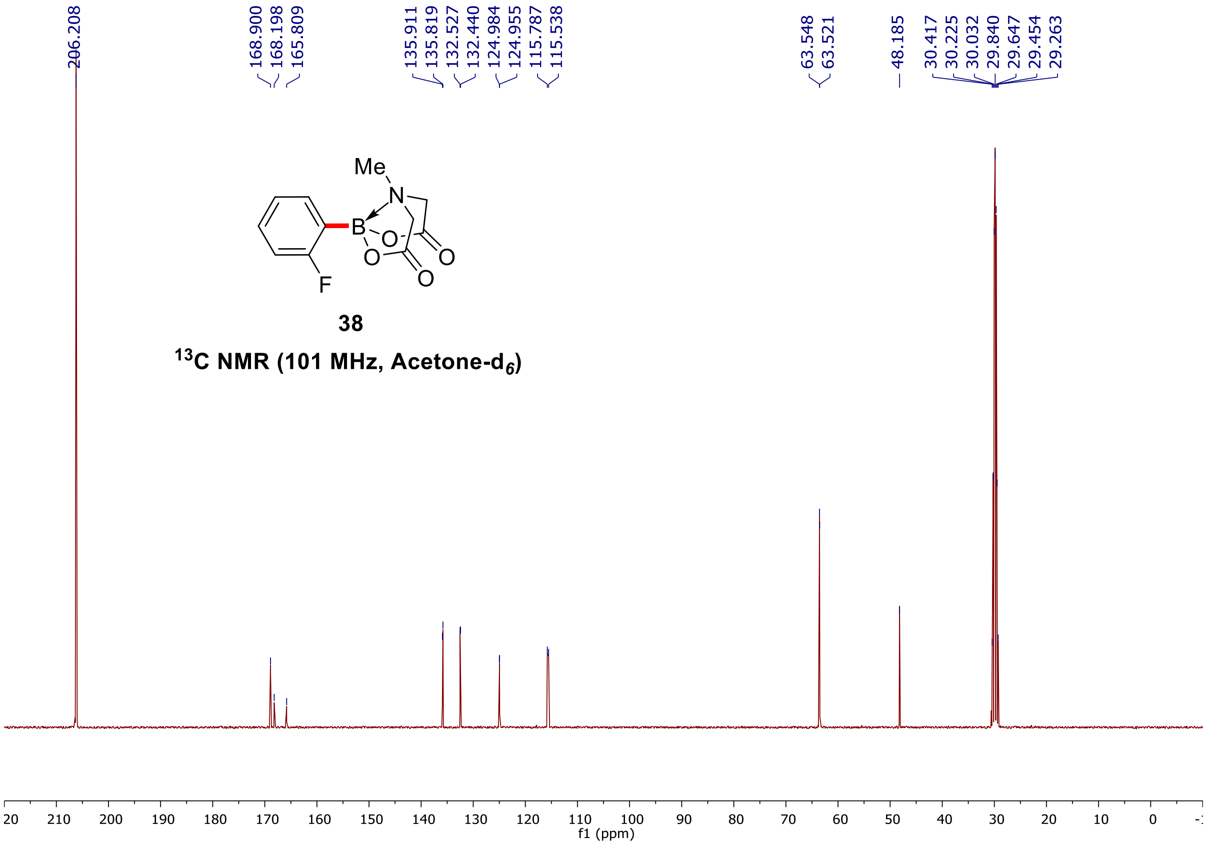

38

^{13}C NMR (101 MHz, Acetone-d_6)

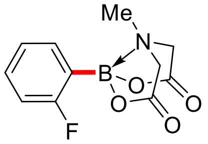

38

^{11}B NMR (128 MHz, Acetone-d$_6$)

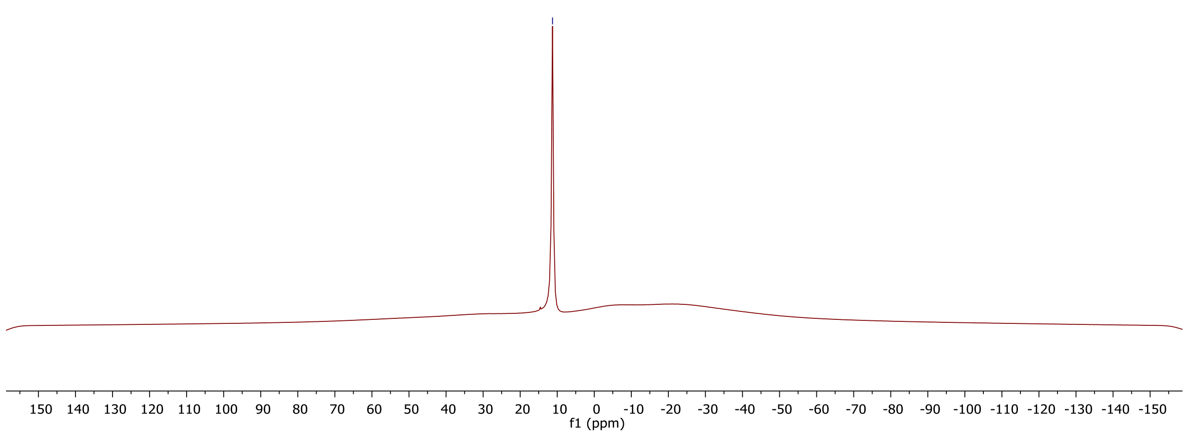

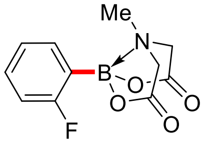

38

^{19}F NMR (471 MHz, Acetone-d$_6$)

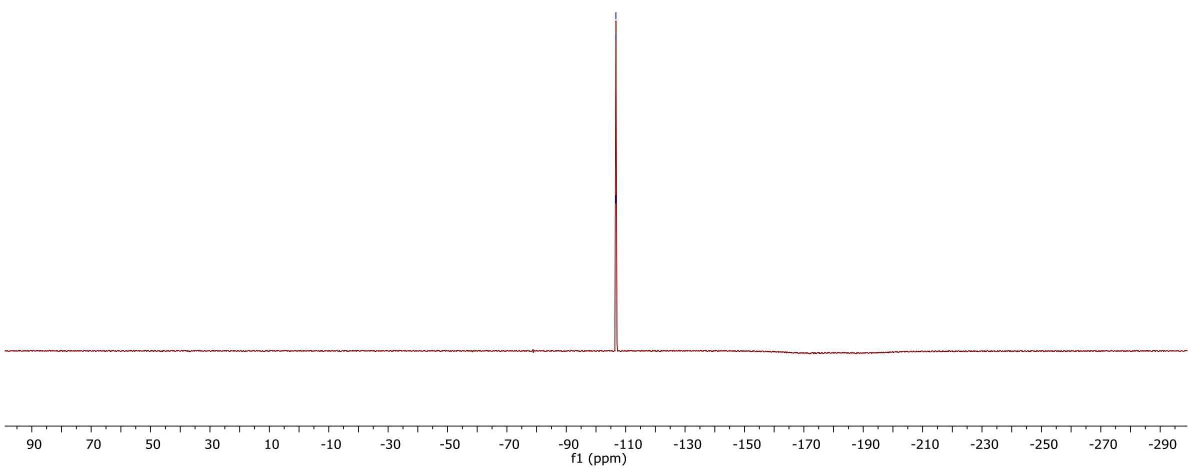

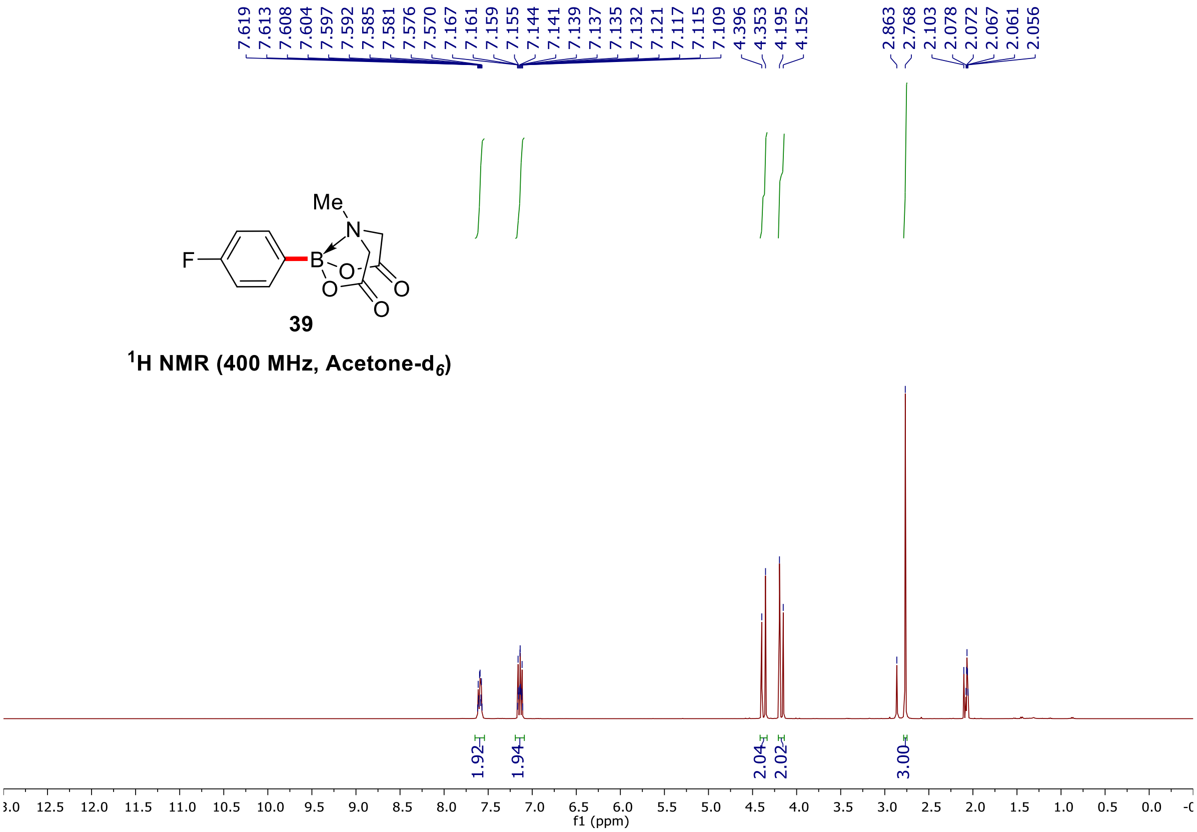
7.619
7.613
7.608
7.604
7.597
7.592
7.585
7.581
7.576
7.570
7.167
7.161
7.159
7.155
7.144
7.141
7.139
7.137
7.135
7.132
7.121
7.117
7.115
7.109
4.396
4.353
4.195
4.152
2.863
2.768
2.103
2.078
2.072
2.067
2.061
2.056
Me
N
F
B
O
O
O
O
39
1H NMR (400 MHz, Acetone-d6)
1.92
1.94
2.04
2.02
3.00
f1 (ppm)

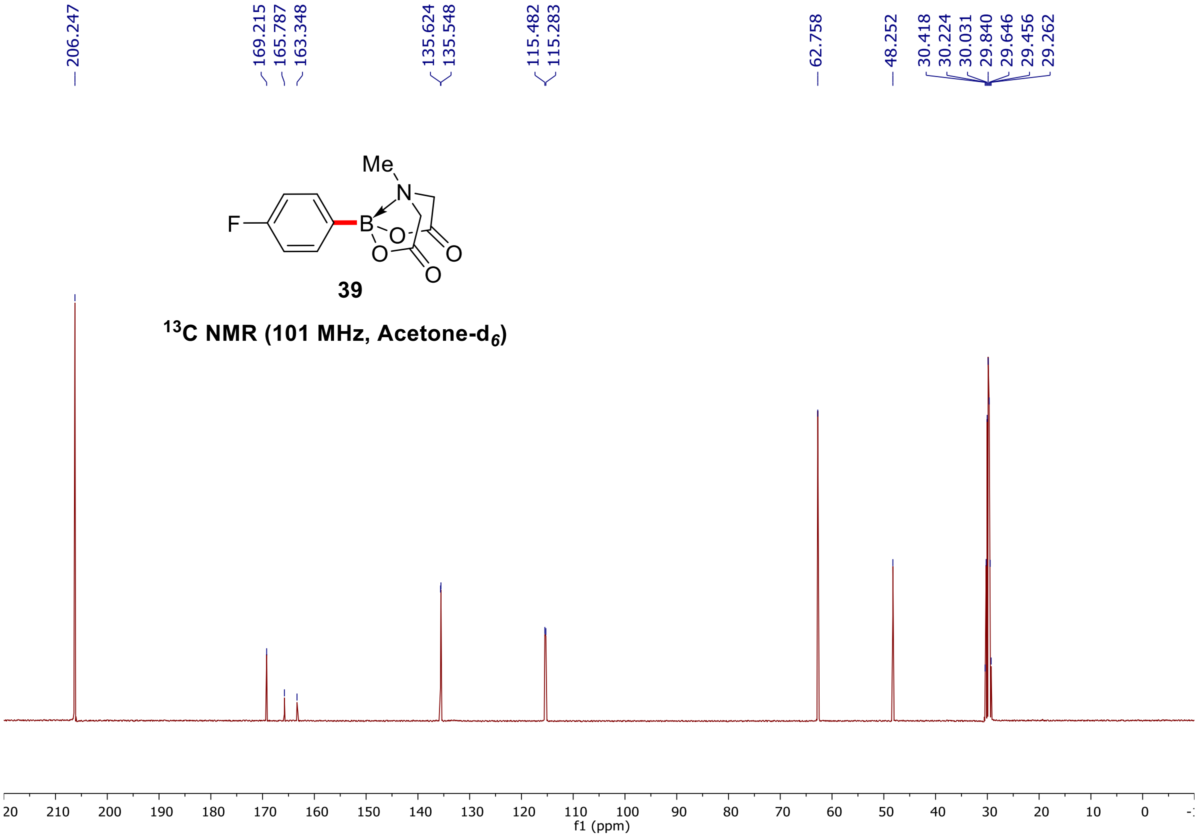
206.247
169.215
165.787
163.348
135.624
135.548
115.482
115.283
62.758
48.252
30.418
30.224
30.031
29.840
29.646
29.456
29.262
Me
N
F
B
O
O
O
O
39
13C NMR (101 MHz, Acetone-d6)
f1 (ppm)

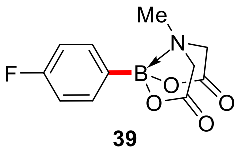

39

^{11}B NMR (128 MHz, Acetone-d_6)

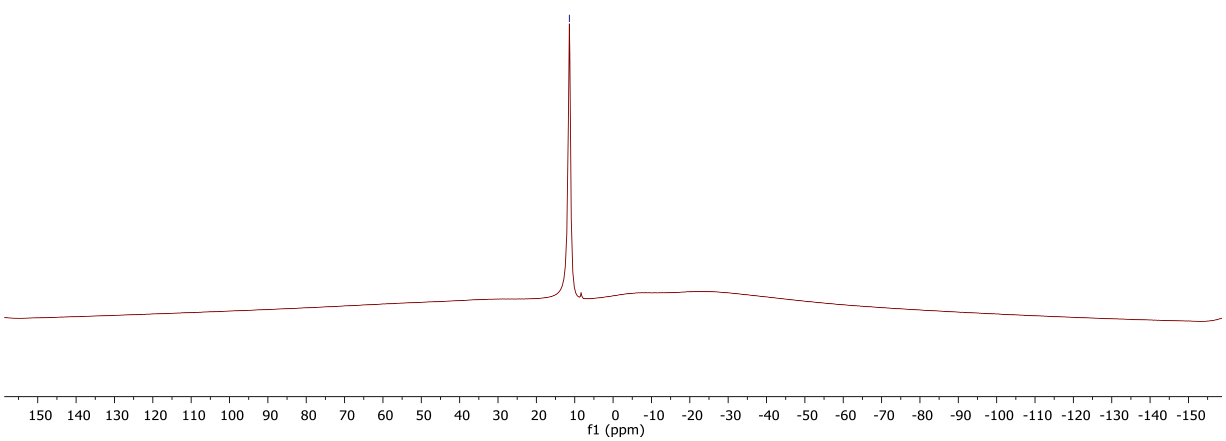

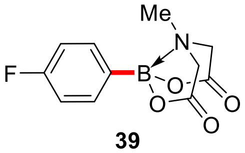

39

^{19}F NMR (282 MHz, Acetone-d_6)

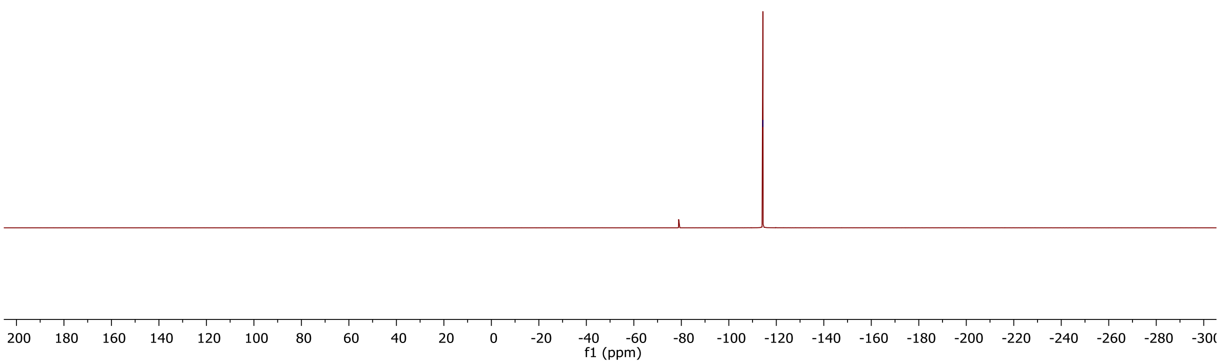

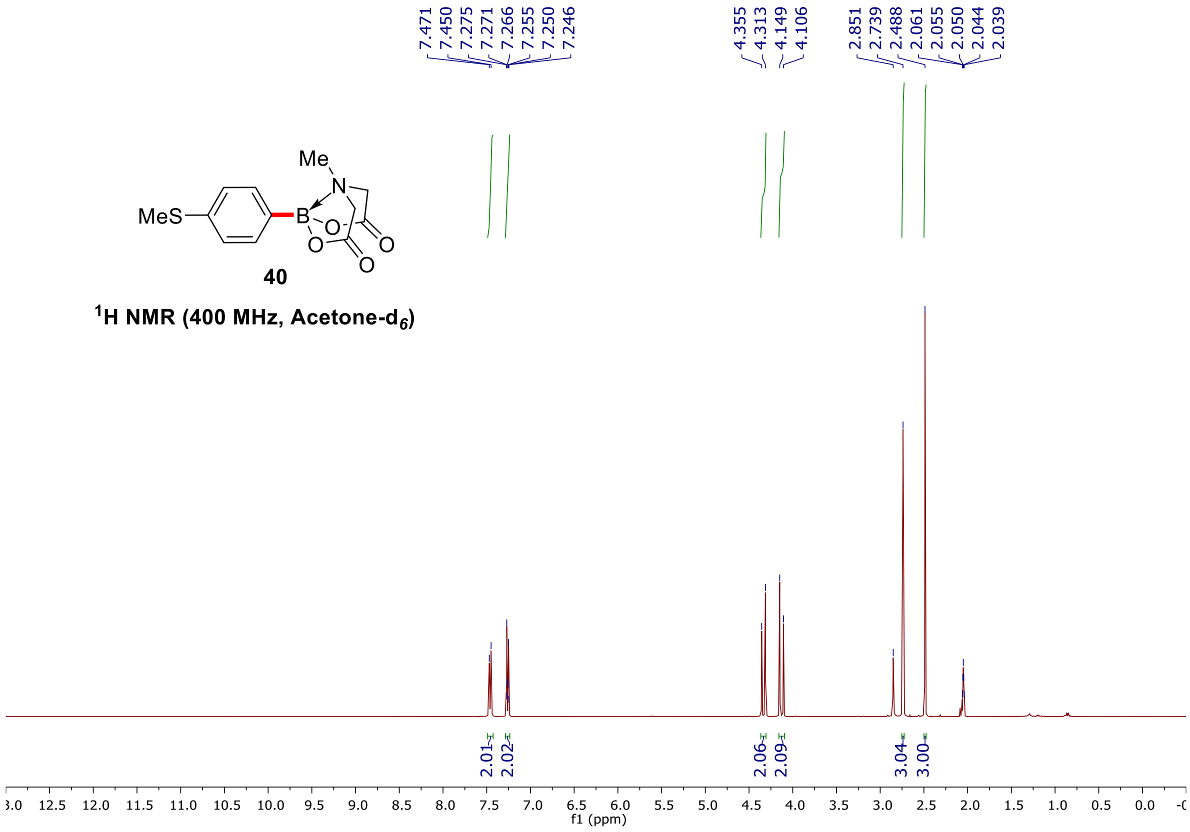
7.471
7.450
7.275
7.271
7.266
7.255
7.250
7.246
4.355
4.313
4.149
4.106
2.851
2.739
2.488
2.061
2.055
2.050
2.044
2.039
Me
N
MeS
B
O
O
O
O
40
1H NMR (400 MHz, Acetone-d6)
2.01
2.02
2.06
2.09
3.04
3.00
f1 (ppm)

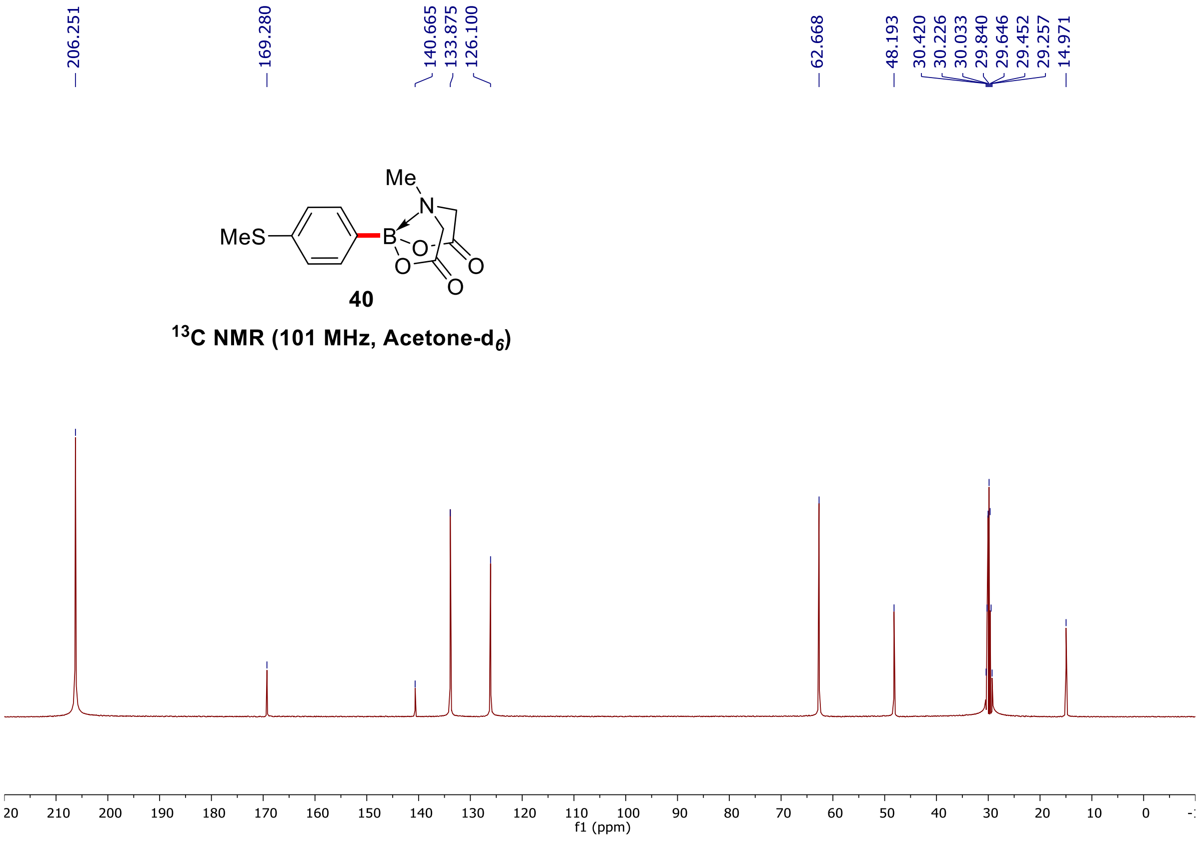
206.251
169.280
140.665
133.875
126.100
62.668
48.193
30.420
30.226
30.033
29.840
29.646
29.452
29.257
14.971
Me
N
MeS
B
O
O
O
O
40
13C NMR (101 MHz, Acetone-d6)
f1 (ppm)

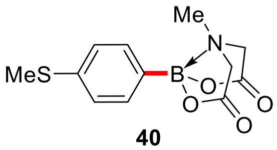

40

^{11}B NMR (128 MHz, Acetone-d_6)

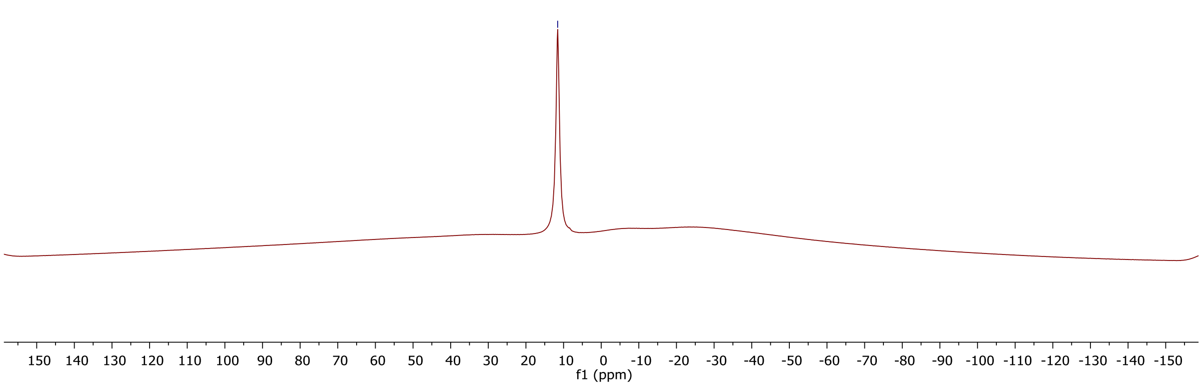

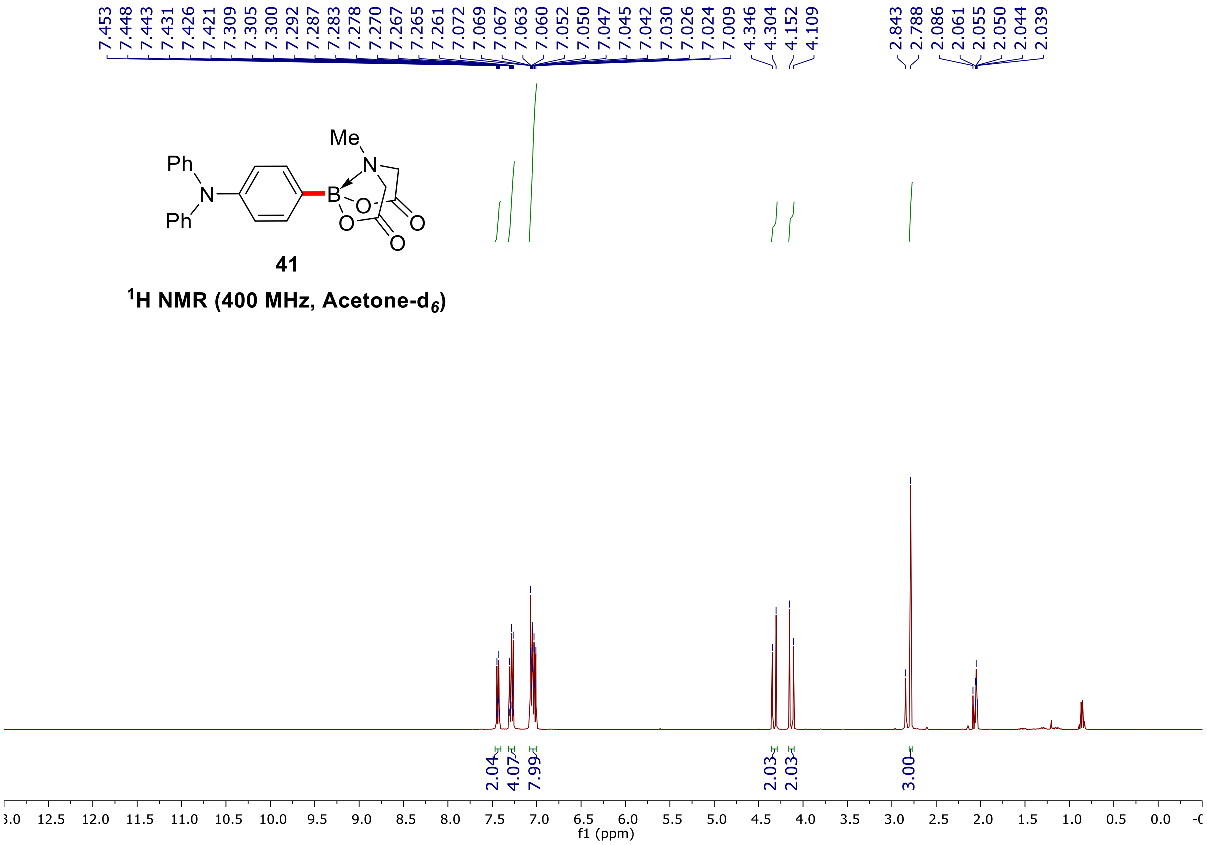
7.453 7.448 7.443 7.431 7.426 7.421 7.309 7.305 7.300 7.292 7.287 7.283 7.278 7.270 7.267 7.265 7.261 7.072 7.069 7.067 7.063 7.060 7.052 7.050 7.047 7.045 7.042 7.030 7.026 7.024 7.009
4.346 4.304 4.152 4.109
2.843 2.788 2.086 2.061 2.055 2.050 2.044 2.039
Me
N
Ph
N
Ph
B
O
O
O
O
41
^{1}H NMR (400 MHz, Acetone-d$_6$)
2.04
4.07
7.99
2.03
2.03
3.00
f1 (ppm)

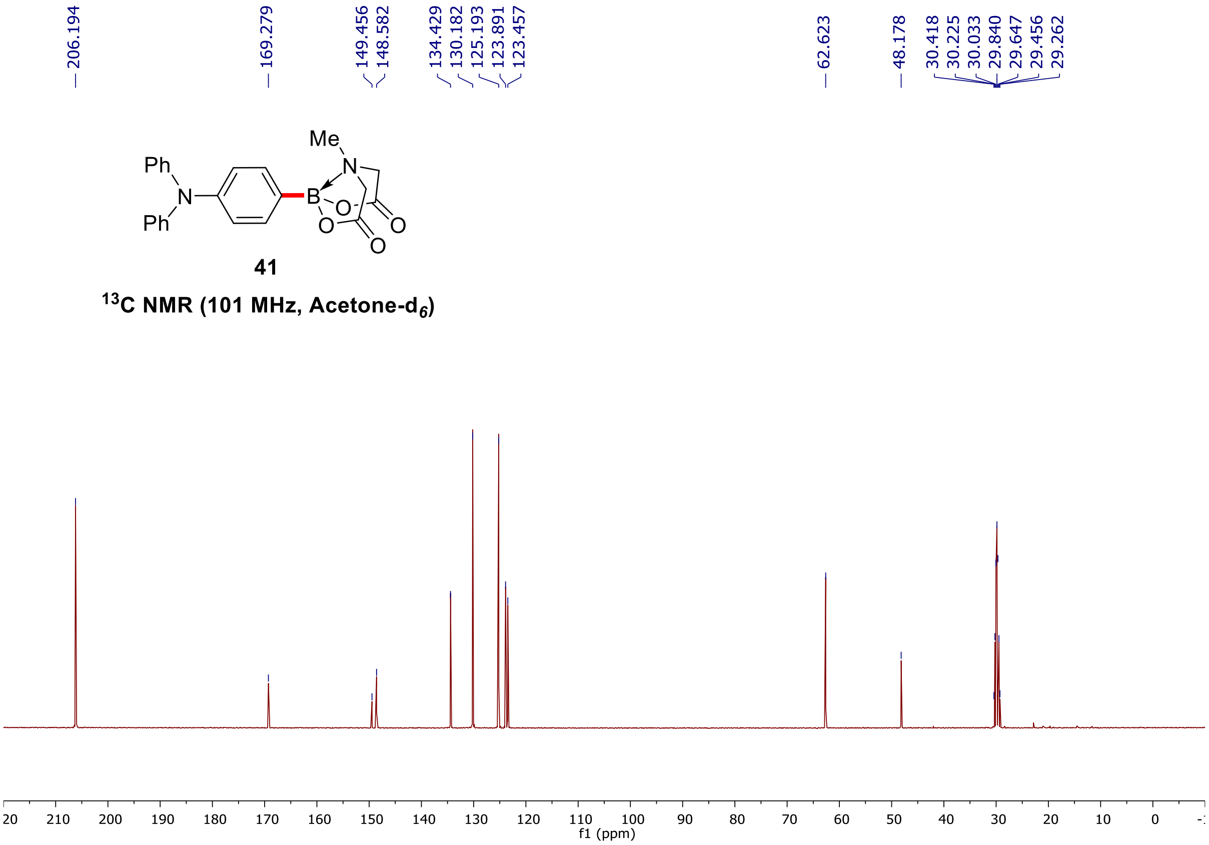
206.194
169.279
149.456 148.582
134.429 130.182 125.193 123.891 123.457
62.623
48.178
30.418 30.225 30.033 29.840 29.647 29.456 29.262
Me
N
Ph
N
Ph
B
O
O
O
O
41
^{13}C NMR (101 MHz, Acetone-d$_6$)
f1 (ppm)

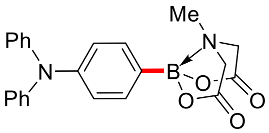

41

^{11}B NMR (128 MHz, Acetone-d_6)

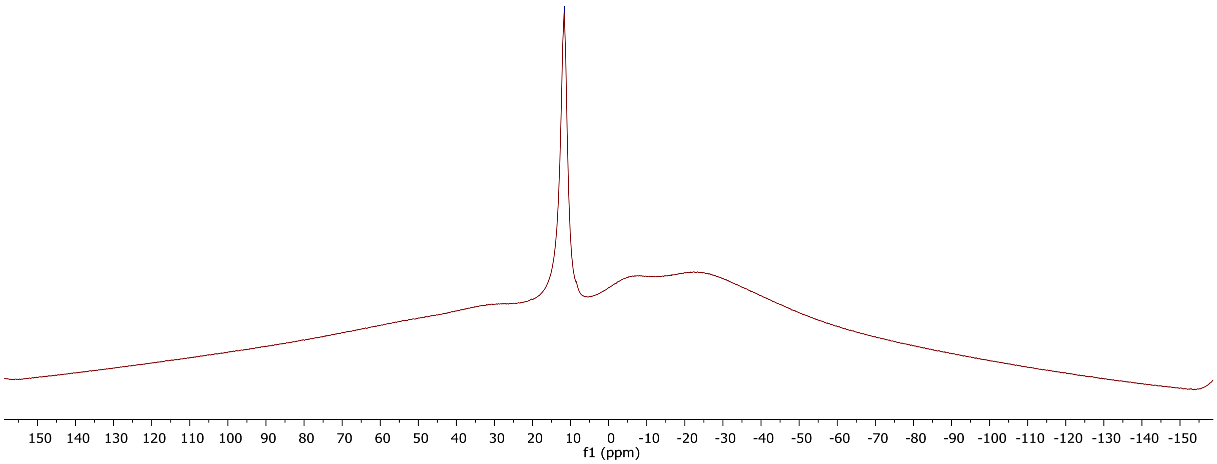

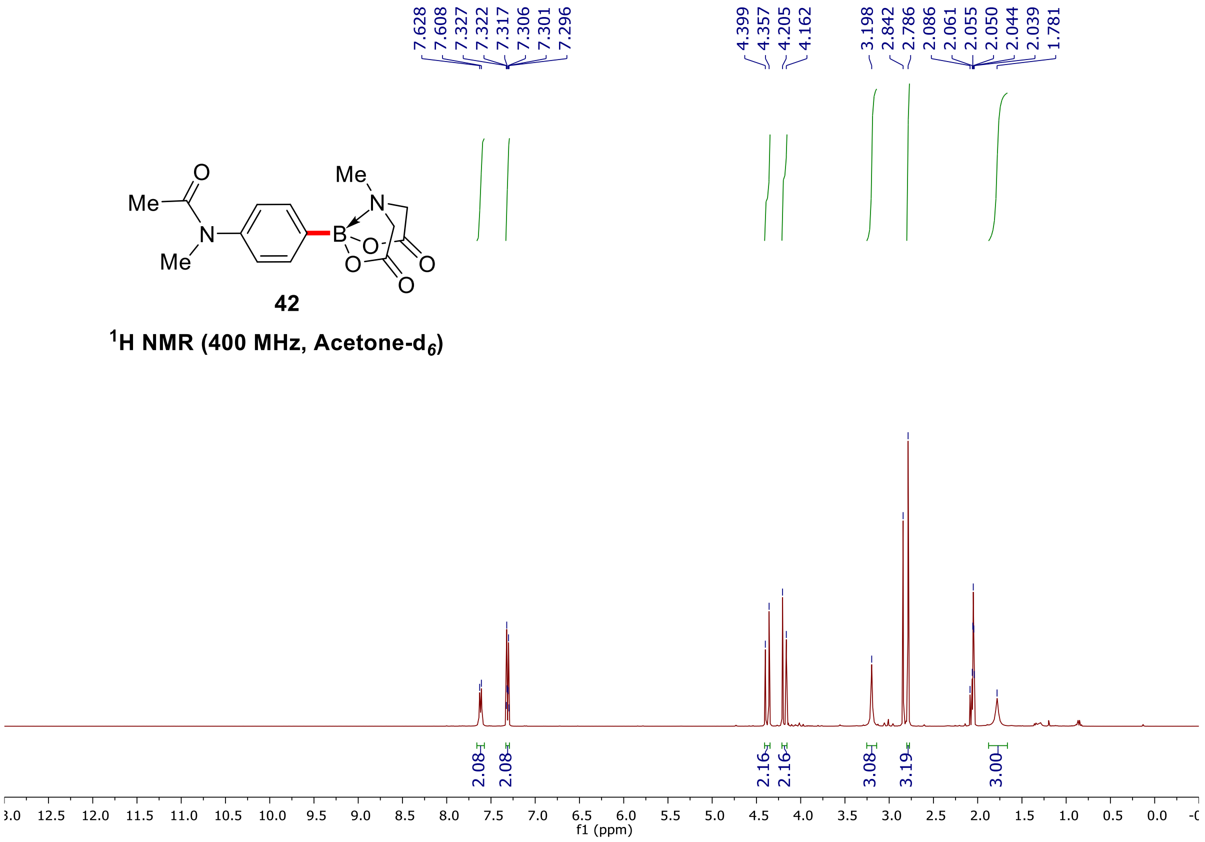
7.628
7.608
7.327
7.322
7.317
7.306
7.301
7.296
4.399
4.357
4.205
4.162
3.198
2.842
2.786
2.086
2.061
2.055
2.050
2.044
2.039
1.781
Me
O
N
Me
B
O
O
O
O
42
^{1}H NMR (400 MHz, Acetone-d$_6$)
2.08
2.08
2.16
2.16
3.08
3.19
3.00
f1 (ppm)

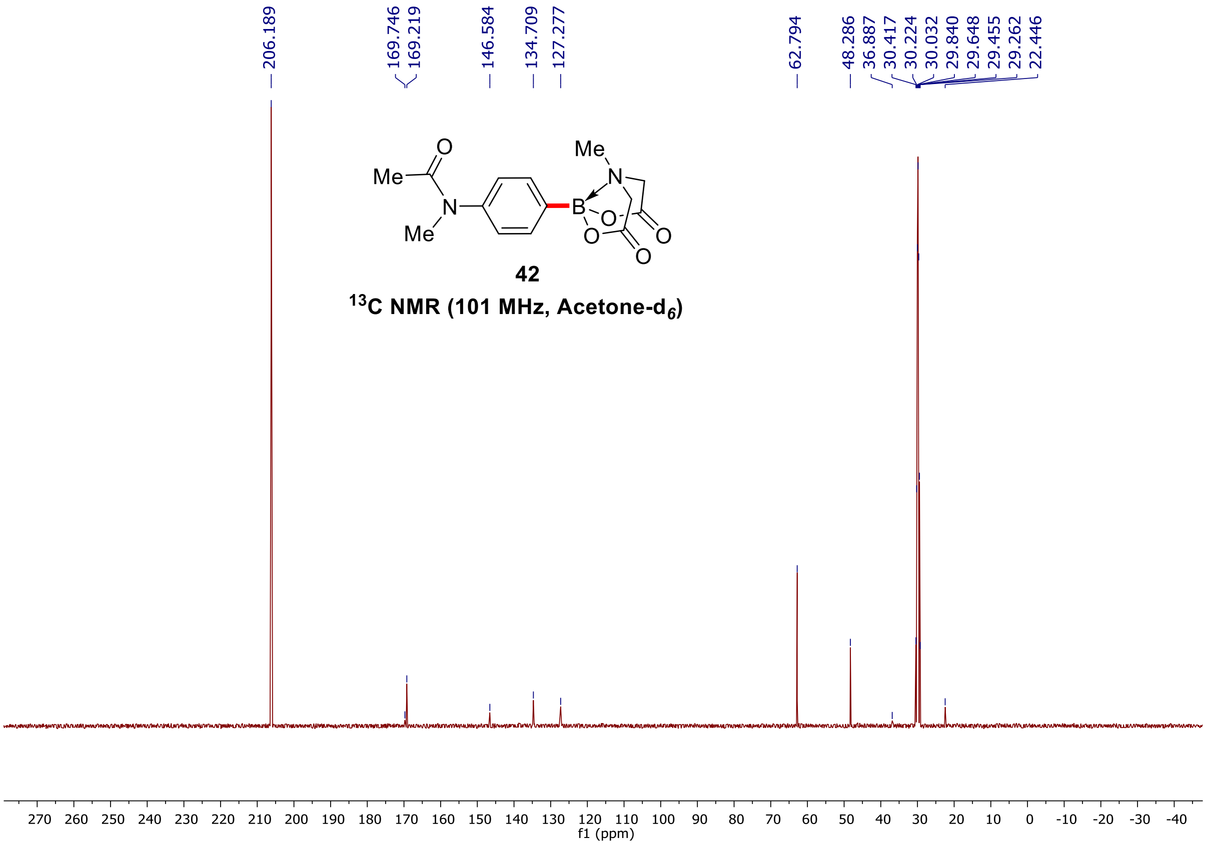
206.189
169.746
169.219
146.584
134.709
127.277
62.794
48.286
36.887
30.417
30.224
30.032
29.840
29.648
29.455
29.262
22.446
Me
O
N
Me
B
O
O
O
O
42
^{13}C NMR (101 MHz, Acetone-d$_6$)
f1 (ppm)

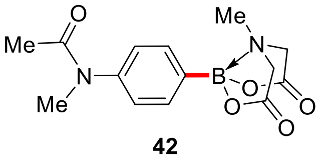

42

^{11}B NMR (128 MHz, Acetone-d$_6$)

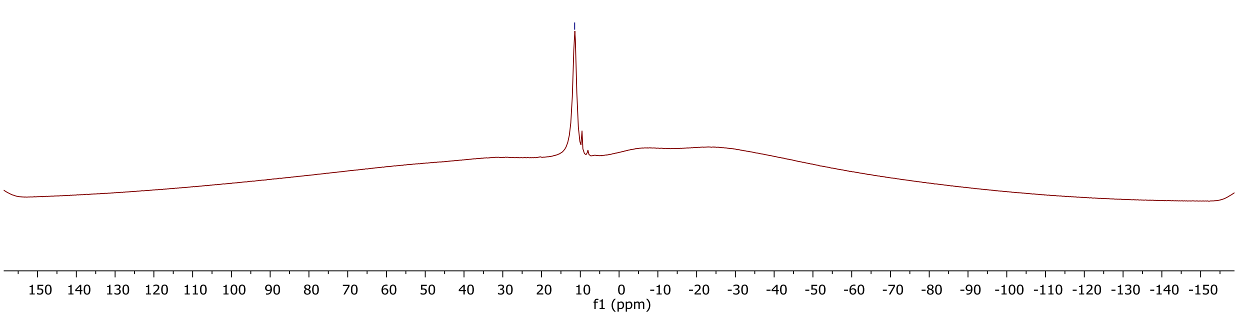

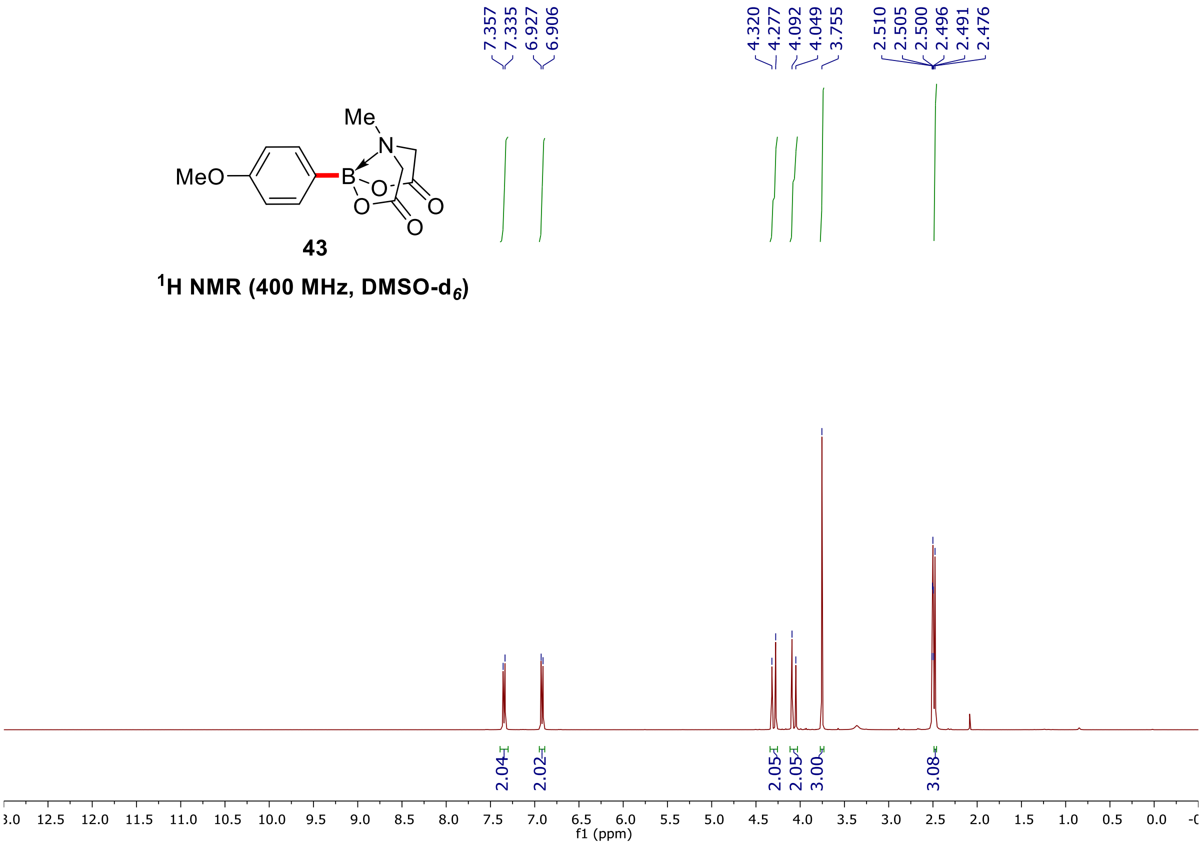

7.357
7.335
6.927
6.906
4.320
4.277
4.092
4.049
3.755
2.510
2.505
2.500
2.496
2.491
2.476
Me
N
MeO
B
O
O
O
O
43
1H NMR (400 MHz, DMSO-d6)
2.04
2.02
2.05
2.05
3.00
3.08
f1 (ppm)

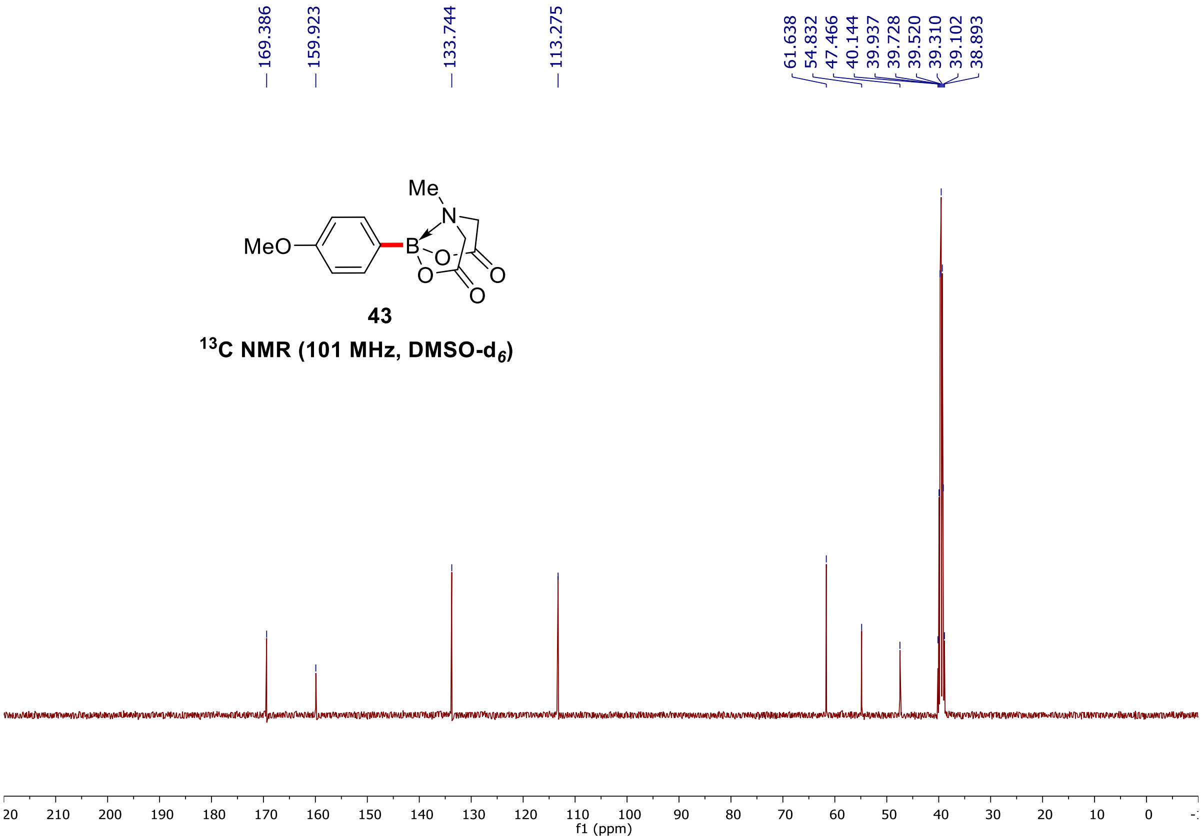

169.386
159.923
133.744
113.275
61.638
54.832
47.466
40.144
39.937
39.728
39.520
39.310
39.102
38.893
Me
N
MeO
B
O
O
O
O
43
13C NMR (101 MHz, DMSO-d6)
f1 (ppm)

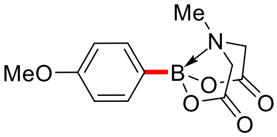

43

^{11}B NMR (128 MHz, DMSO-d_6)

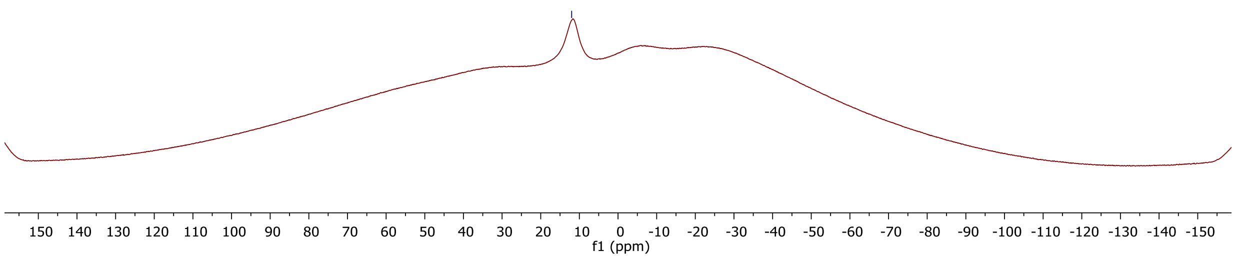

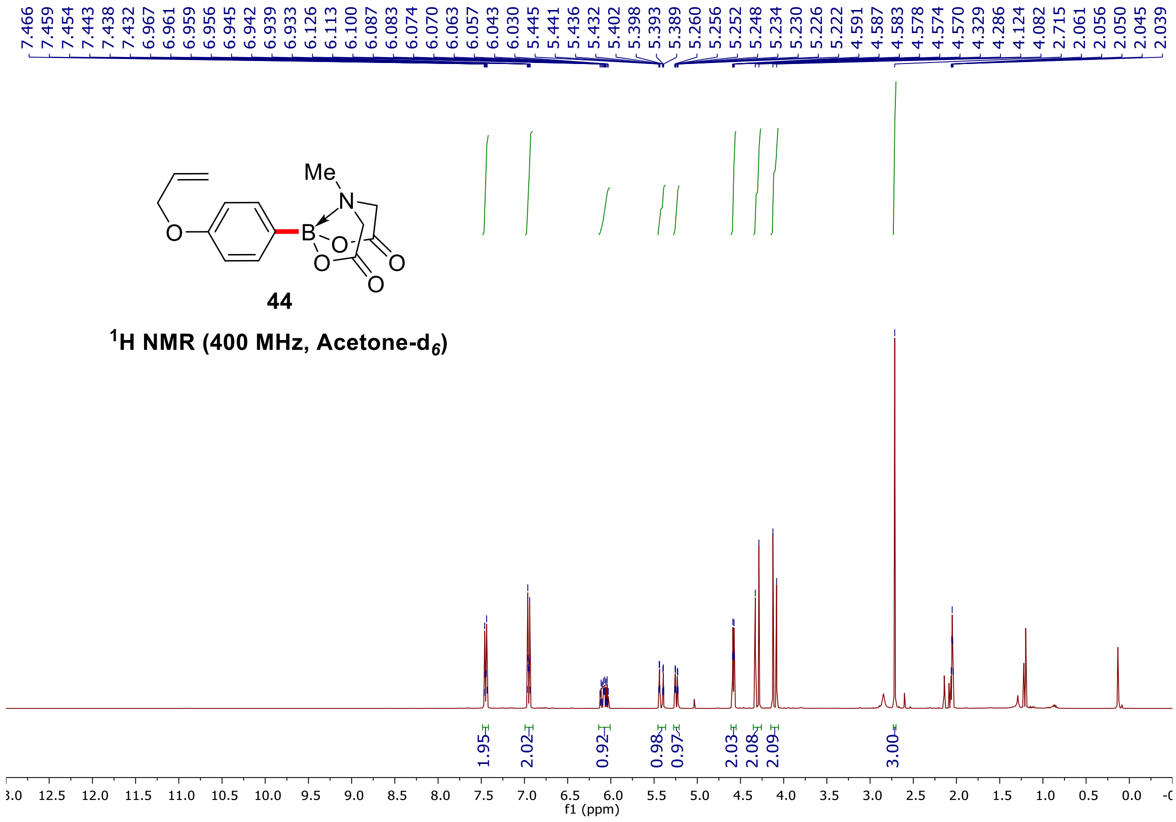
7.466
7.459
7.454
7.443
7.438
7.432
6.967
6.961
6.959
6.956
6.945
6.942
6.939
6.933
6.126
6.113
6.100
6.087
6.083
6.074
6.070
6.063
6.057
6.043
6.030
5.445
5.441
5.436
5.432
5.402
5.398
5.393
5.389
5.260
5.256
5.252
5.248
5.234
5.230
5.226
5.222
4.591
4.587
4.583
4.578
4.574
4.570
4.329
4.286
4.124
4.082
2.715
2.061
2.056
2.050
2.045
2.039
Me
N
B
O
O
O
O
O
44
1H NMR (400 MHz, Acetone-d6)
1.95
2.02
0.92
0.98
0.97
2.03
2.08
2.09
3.00
f1 (ppm)

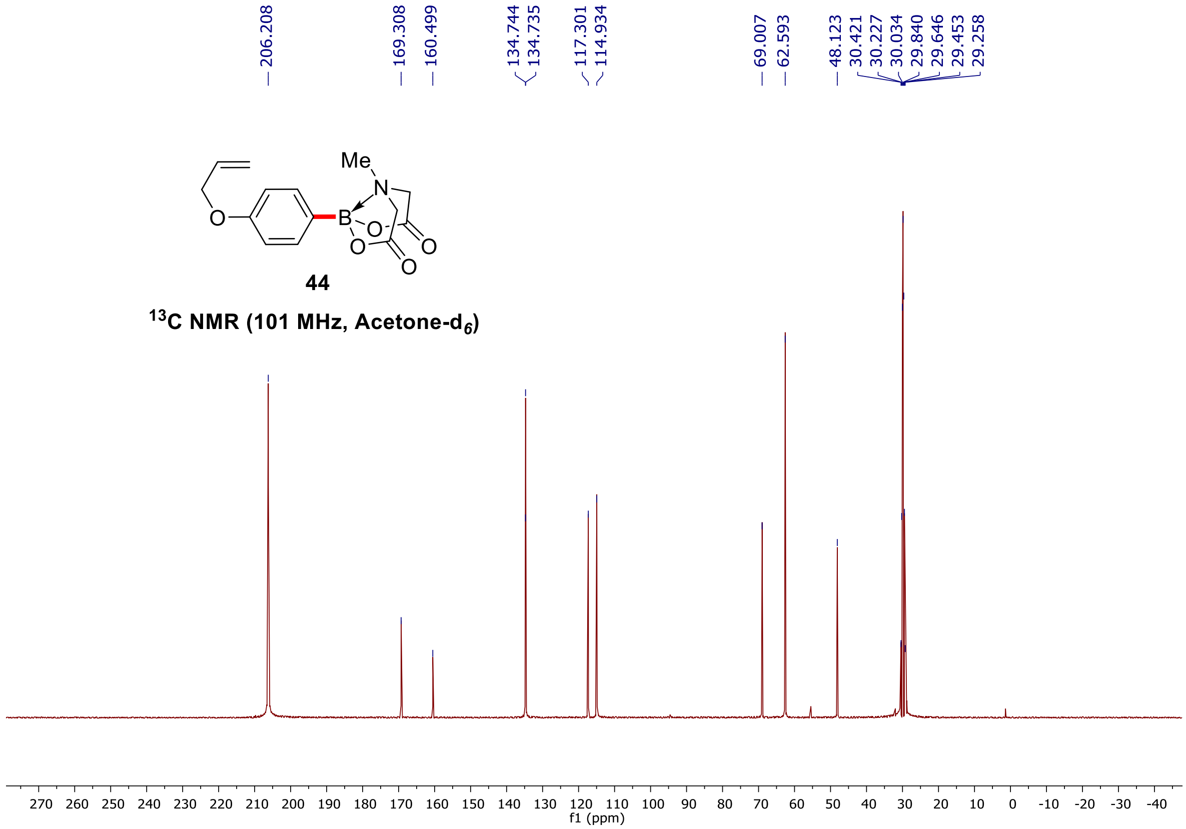
206.208
169.308
160.499
134.744
134.735
117.301
114.934
69.007
62.593
48.123
30.421
30.227
30.034
29.840
29.646
29.453
29.258
Me
N
B
O
O
O
O
O
44
13C NMR (101 MHz, Acetone-d6)
f1 (ppm)

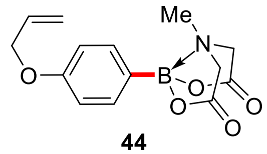

44

^{11}B NMR (128 MHz, Acetone-d_6)

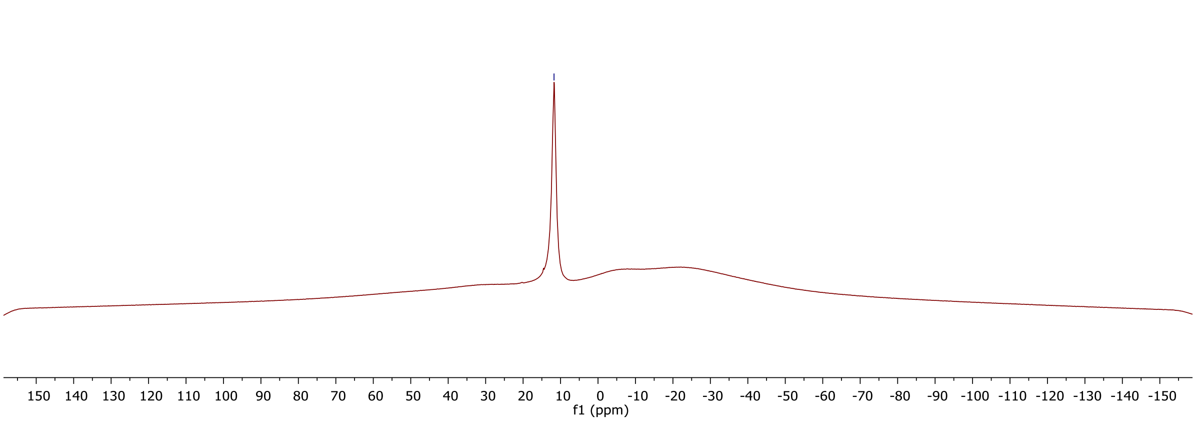

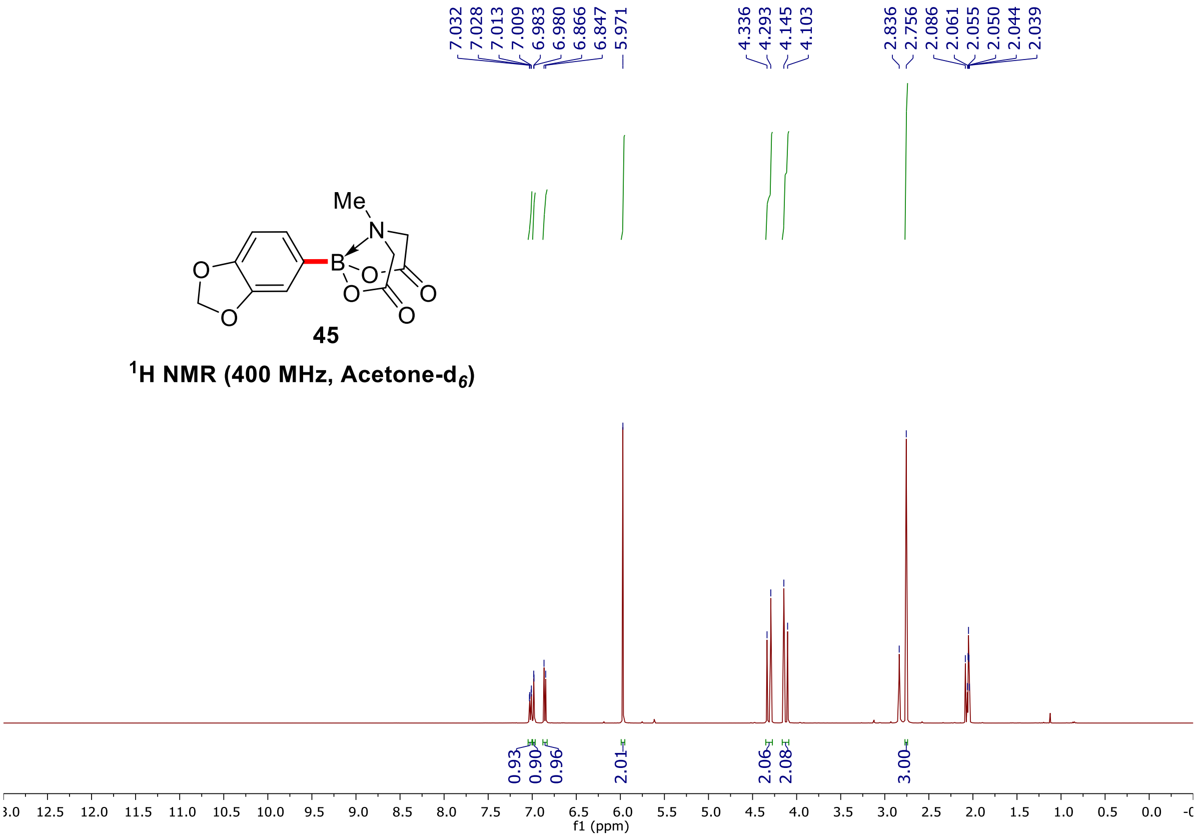

7.032
7.028
7.013
7.009
6.983
6.980
6.866
6.847
5.971
4.336
4.293
4.145
4.103
2.836
2.756
2.086
2.061
2.055
2.050
2.044
2.039
Me
N
O
B
O
O
O
O
O
45
¹H NMR (400 MHz, Acetone-d6)
0.93
0.90
0.96
2.01
2.06
2.08
3.00
f1 (ppm)

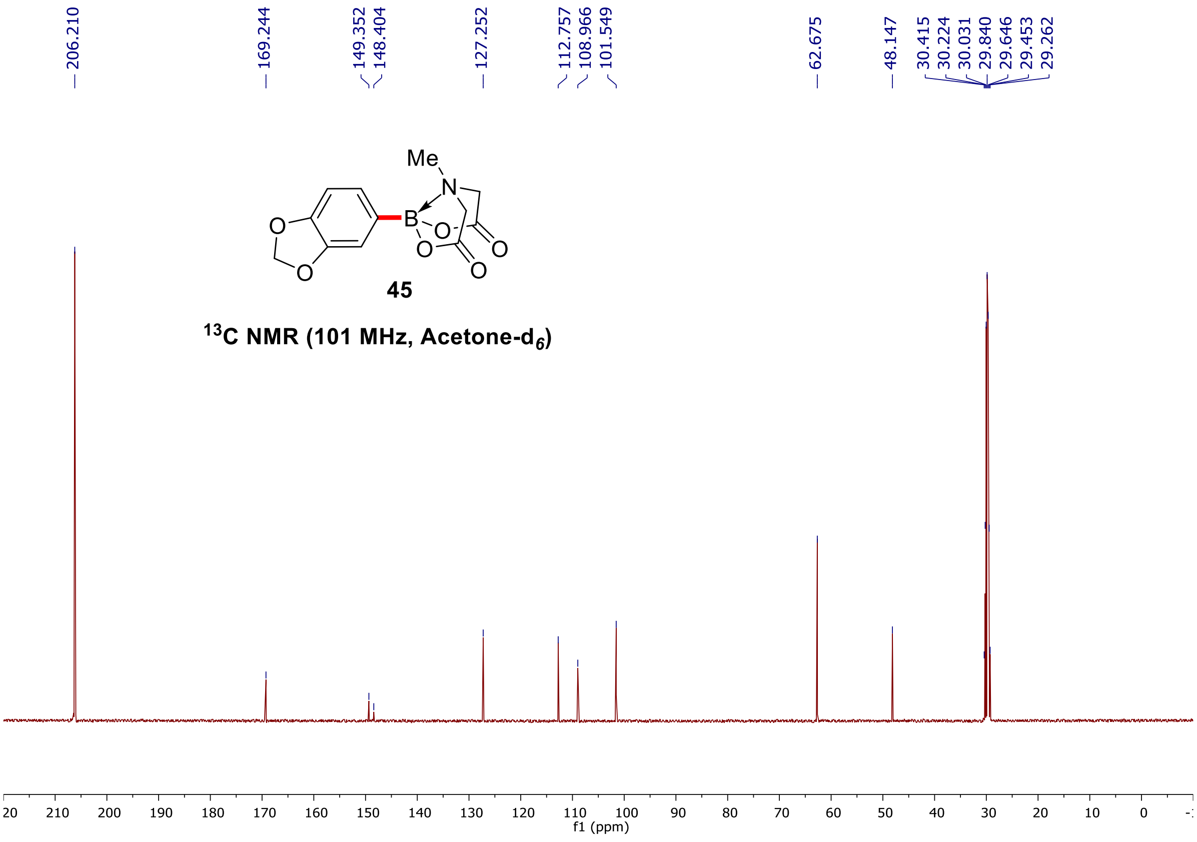

206.210
169.244
149.352
148.404
127.252
112.757
108.966
101.549
62.675
48.147
30.415
30.224
30.031
29.840
29.646
29.453
29.262
Me
N
O
B
O
O
O
O
O
45
¹³C NMR (101 MHz, Acetone-d6)
f1 (ppm)

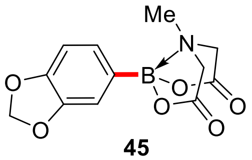

45

^{11}B NMR (128 MHz, Acetone-d_6)

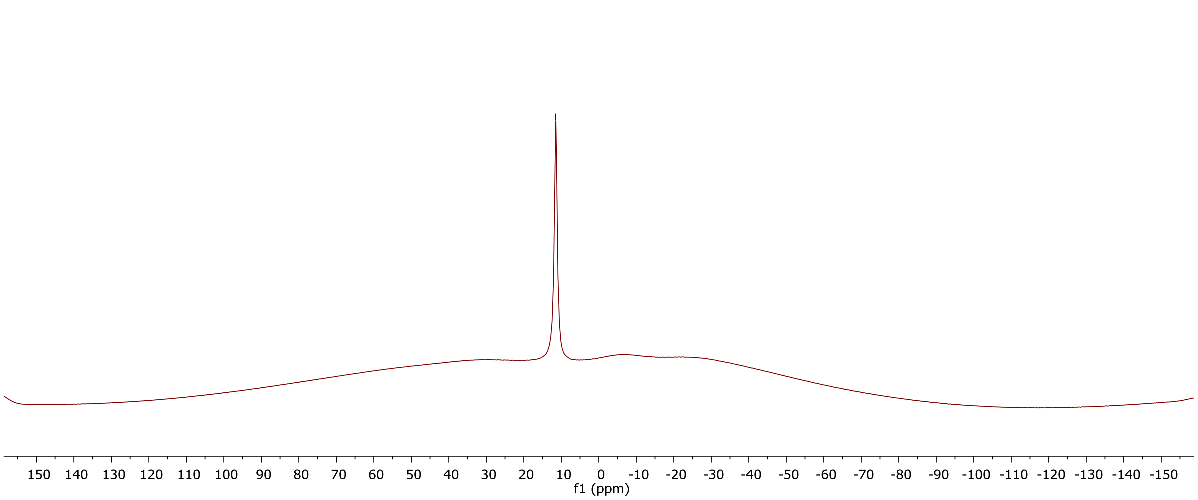

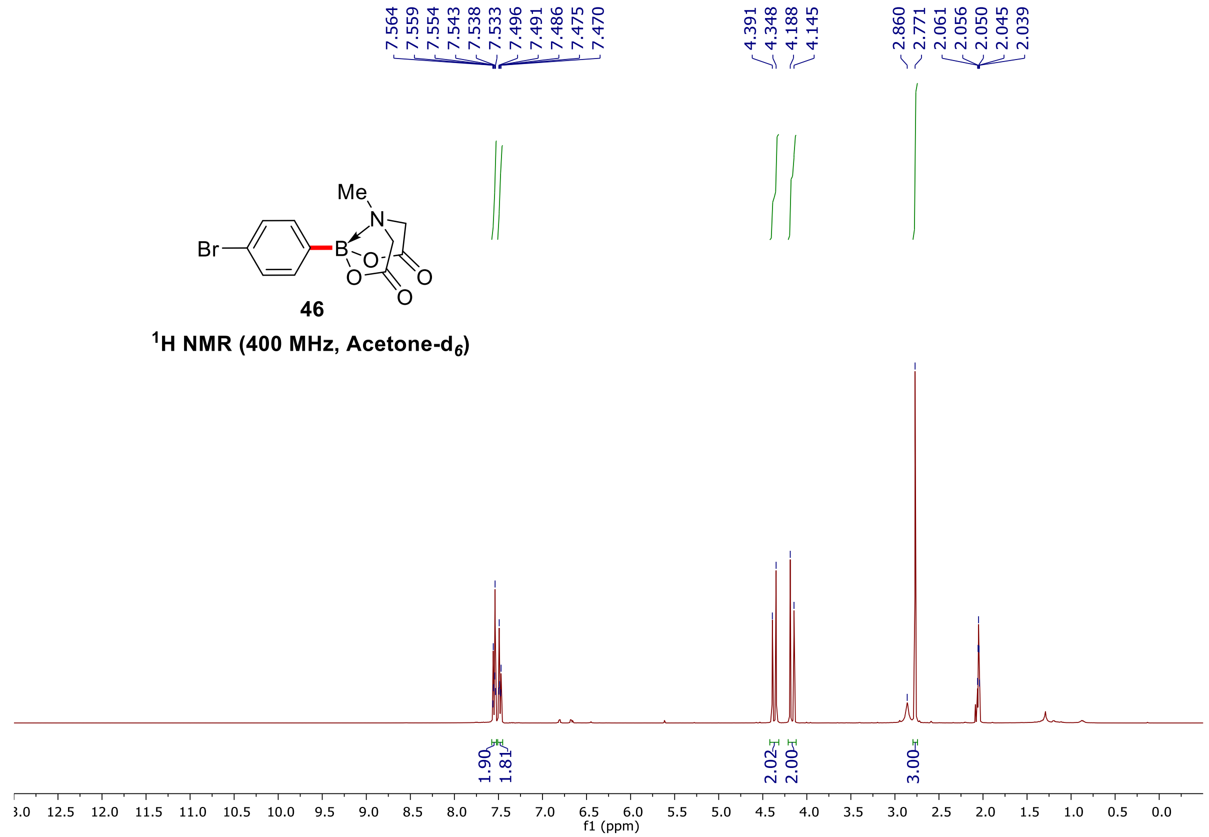
7.564 7.559 7.554 7.543 7.538 7.533 7.496 7.491 7.486 7.475 7.470
4.391 4.348 4.188 4.145
2.860 2.771 2.061 2.056 2.050 2.045 2.039
Me
N
Br
B
O
O
O
O
46
1H NMR (400 MHz, Acetone-d6)
1.90
1.81
2.02
2.00
3.00
f1 (ppm)

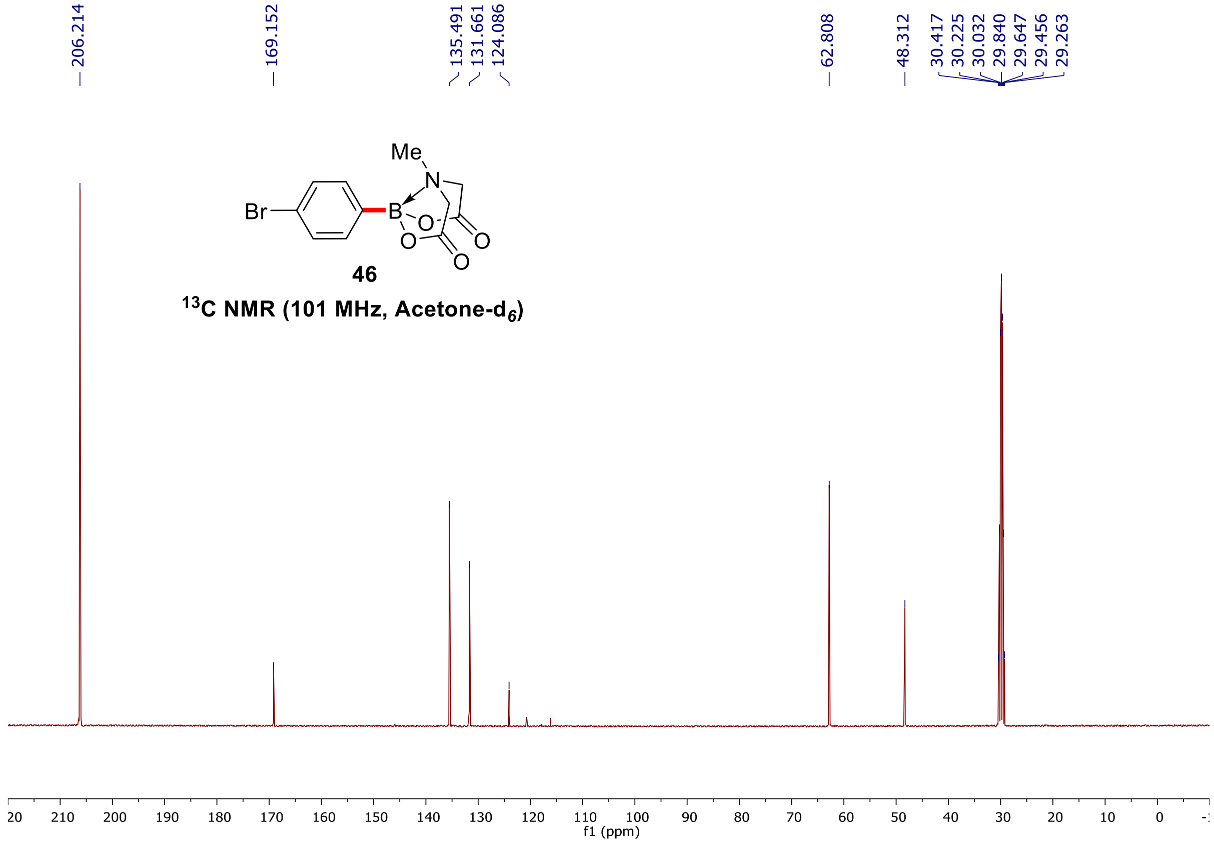
206.214
169.152
135.491 131.661 124.086
62.808
48.312
30.417 30.225 30.032 29.840 29.647 29.456 29.263
Me
N
Br
B
O
O
O
O
46
13C NMR (101 MHz, Acetone-d6)
f1 (ppm)

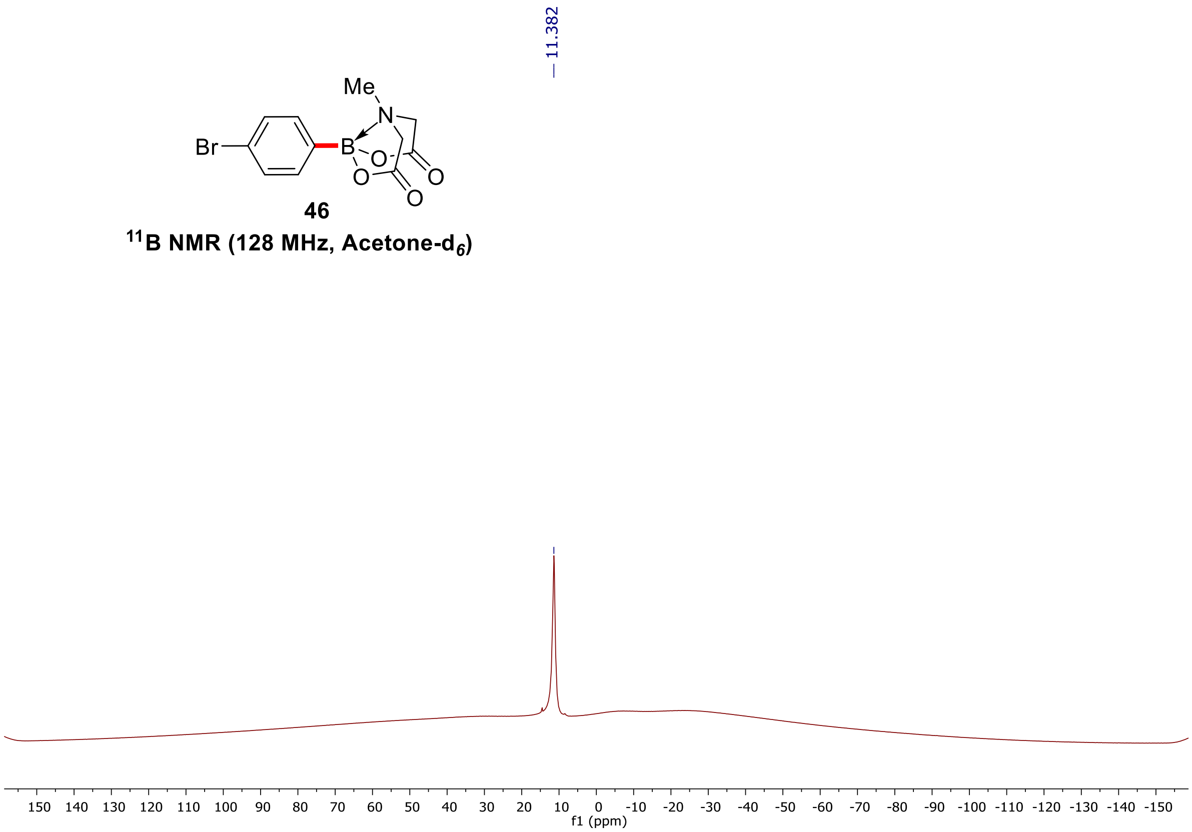

46

^{11}B NMR (128 MHz, Acetone-d$_6$)

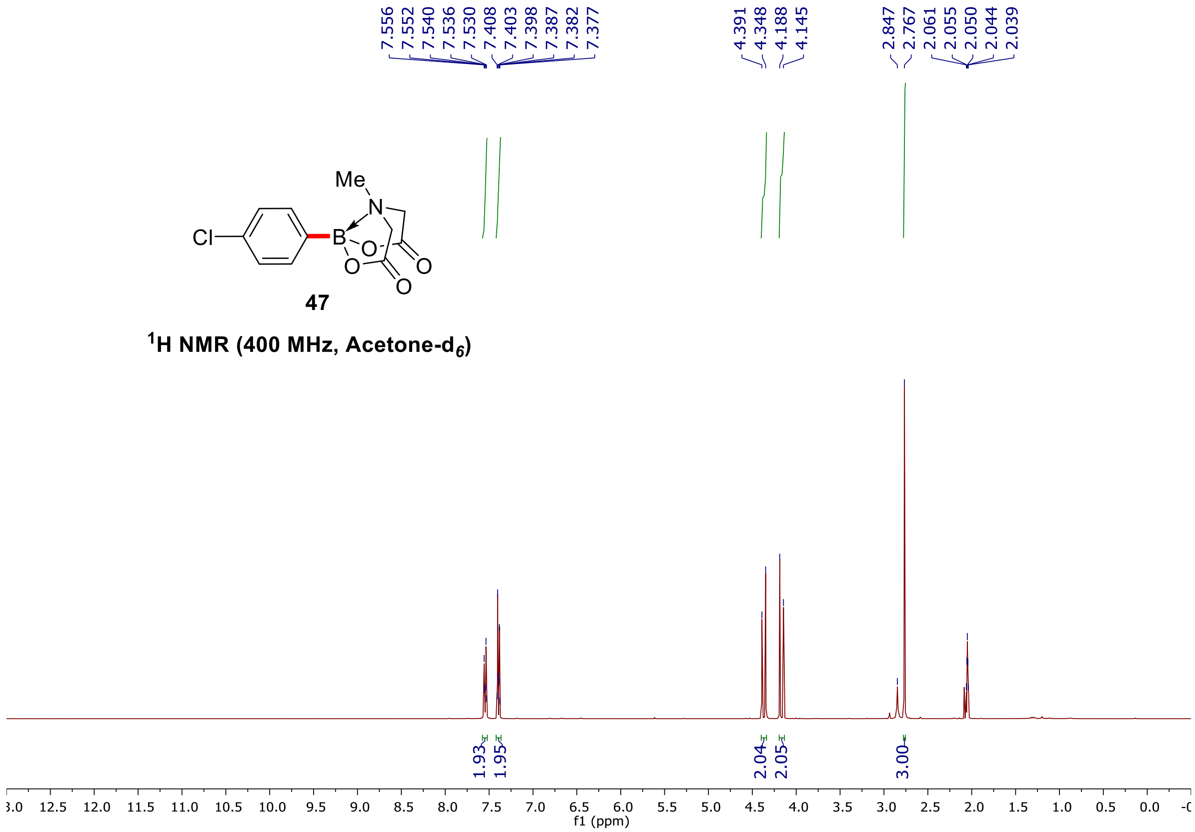
7.556
7.552
7.540
7.536
7.530
7.408
7.403
7.398
7.387
7.382
7.377
4.391
4.348
4.188
4.145
2.847
2.767
2.061
2.055
2.050
2.044
2.039
Me
N
Cl
B
O
O
O
O
47
^{1}H NMR (400 MHz, Acetone-d_6)
1.93
1.95
2.04
2.05
3.00
f1 (ppm)

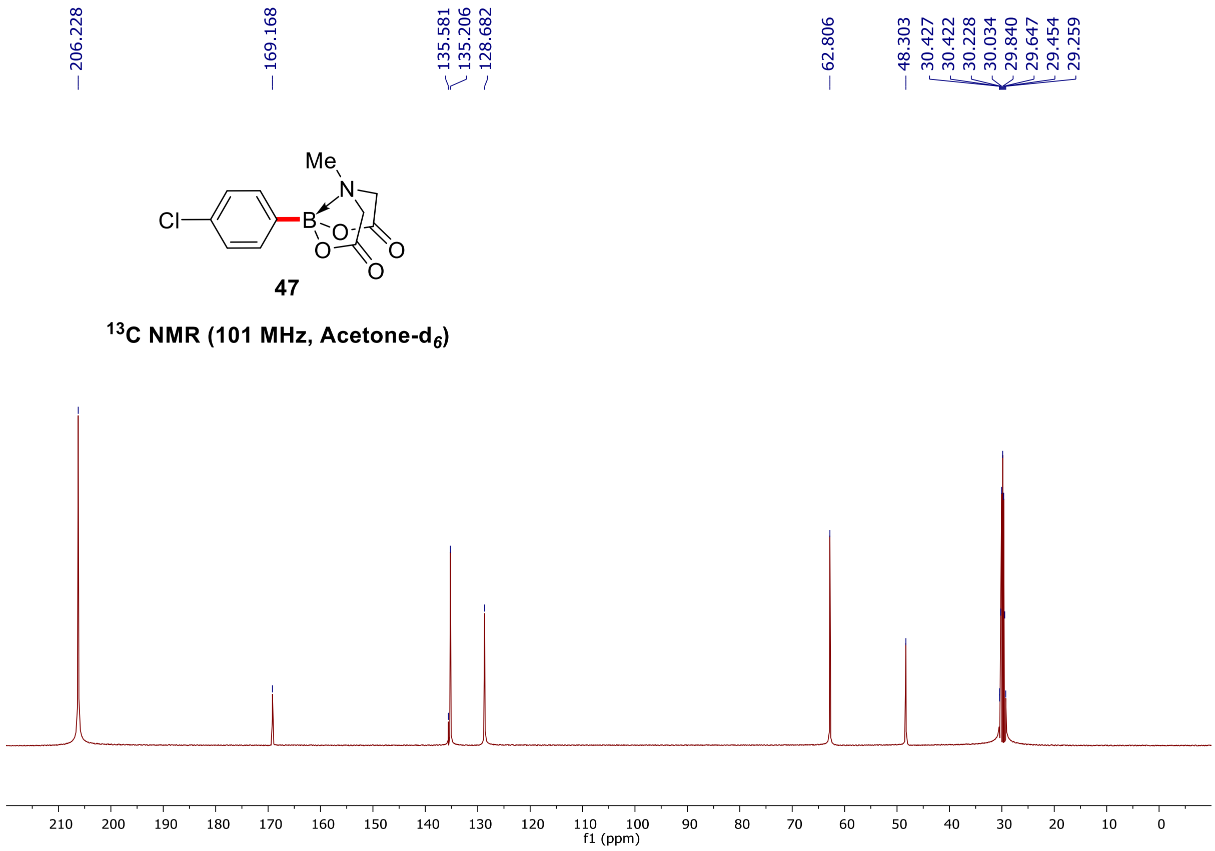
206.228
169.168
135.581
135.206
128.682
62.806
48.303
30.427
30.422
30.228
30.034
29.840
29.647
29.454
29.259
Me
N
Cl
B
O
O
O
O
47
^{13}C NMR (101 MHz, Acetone-d_6)
f1 (ppm)

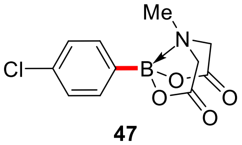

47

^{11}B NMR (128 MHz, Acetone-d$_6$)

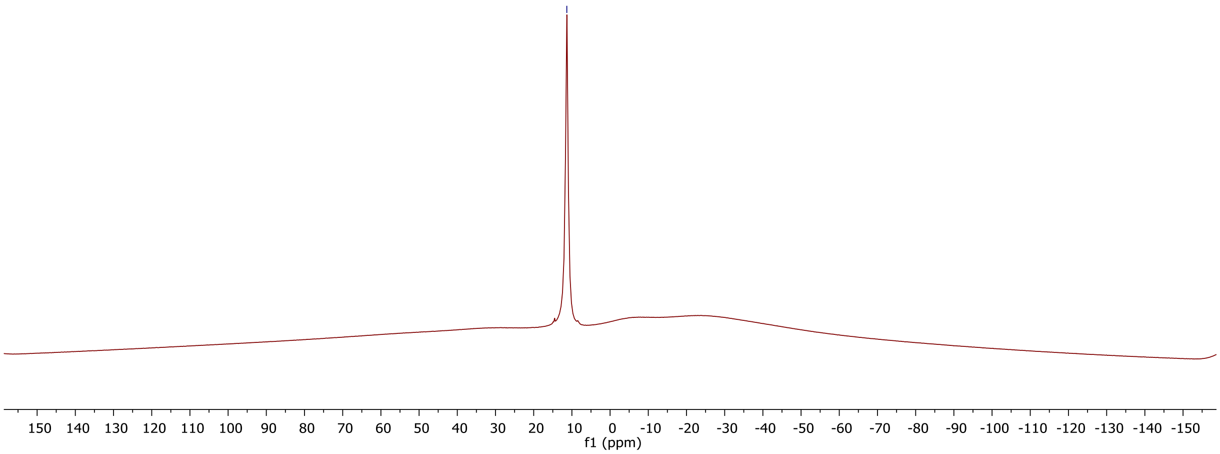

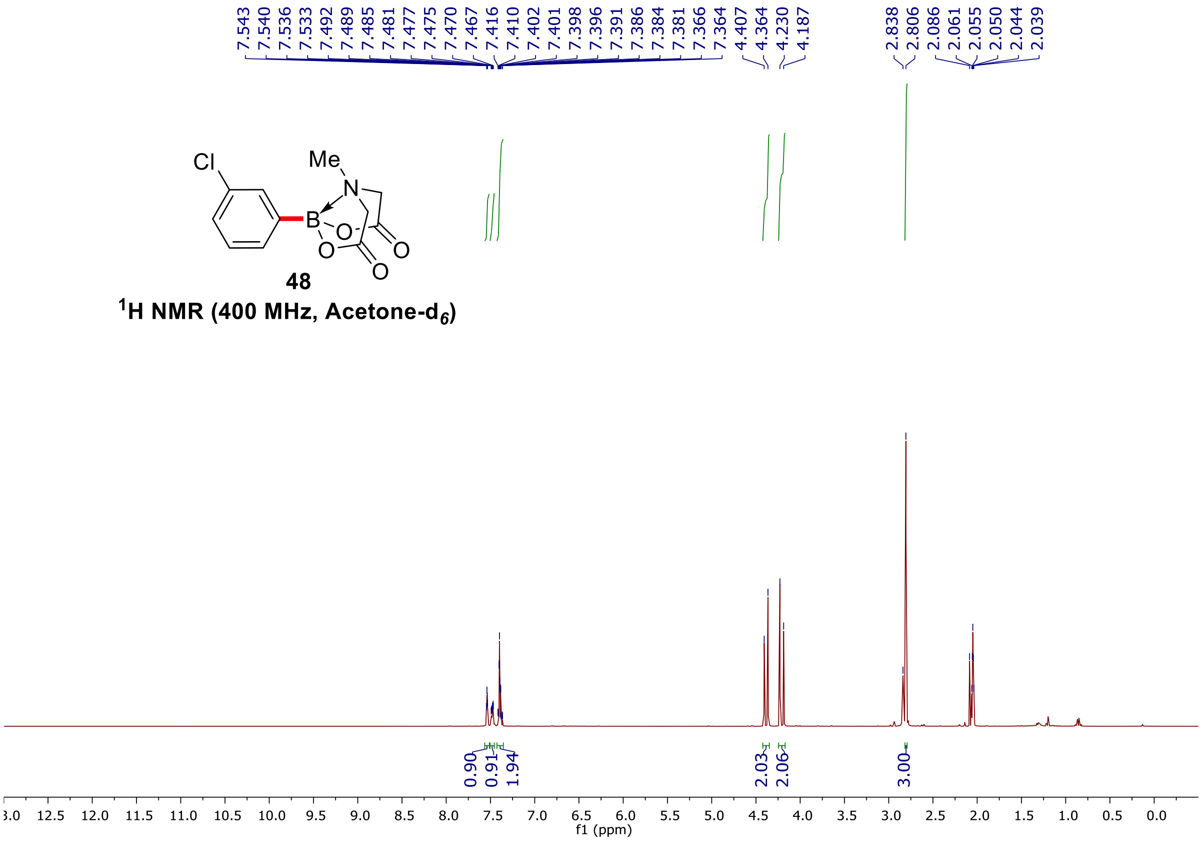

48

^{1}H NMR (400 MHz, Acetone-d$_6$)

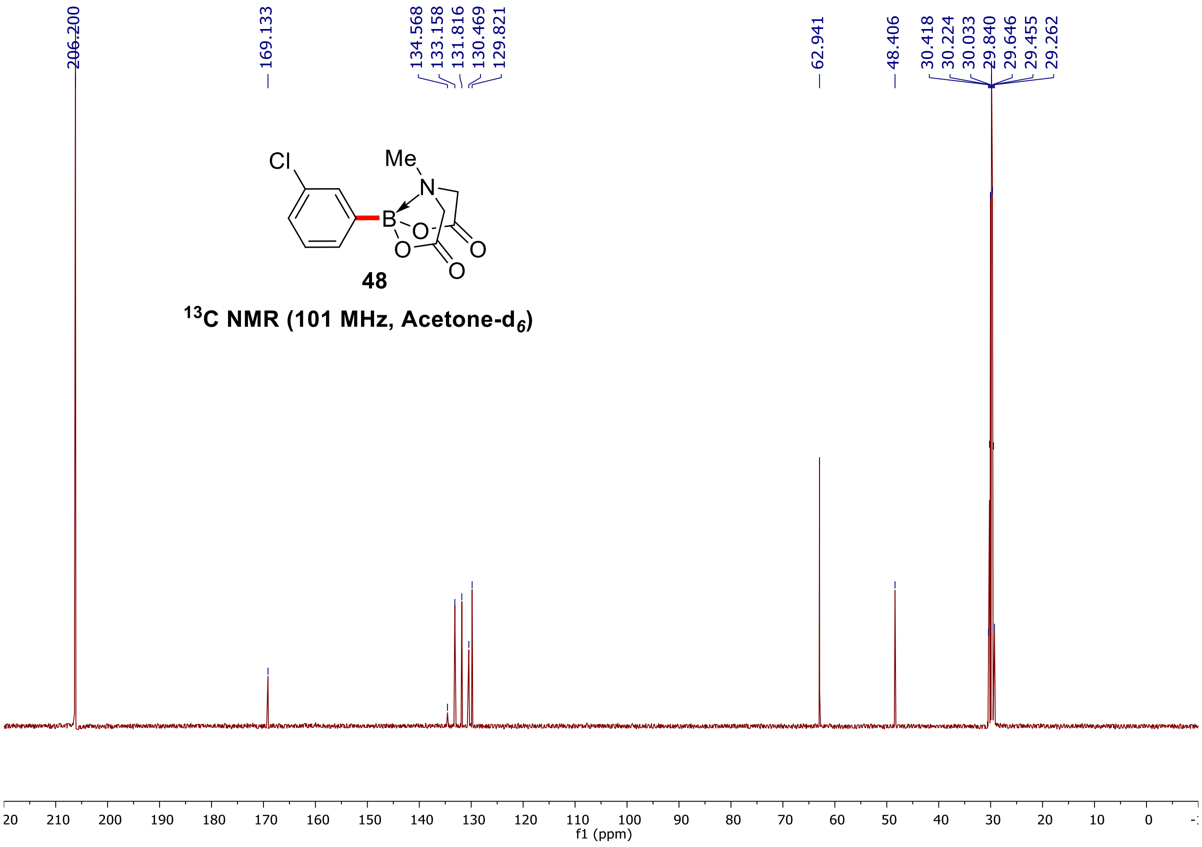

48

^{13}C NMR (101 MHz, Acetone-d$_6$)

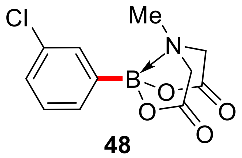

48

^{11}B NMR (128 MHz, Acetone-d_6)

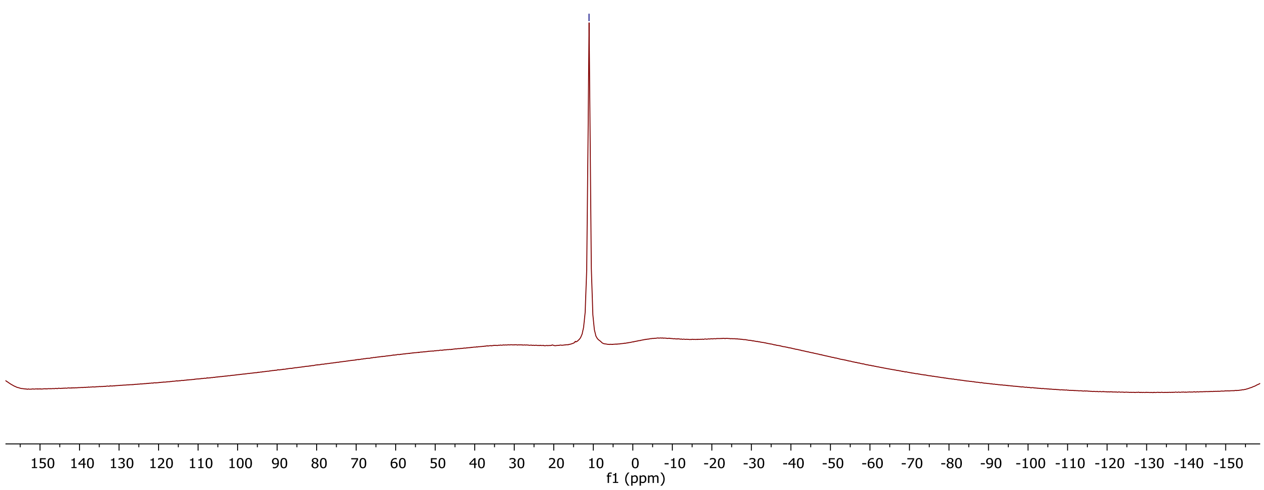

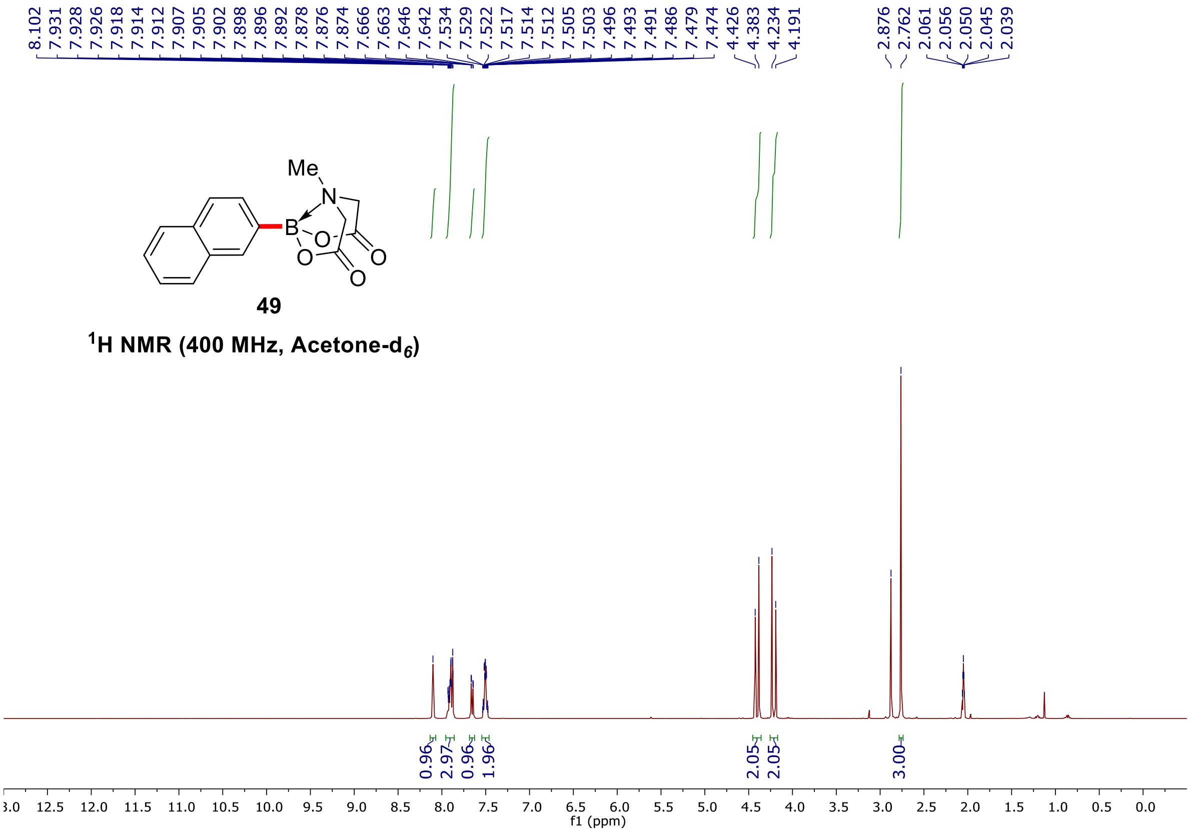

49

^{1}H NMR (400 MHz, Acetone-d$_6$)

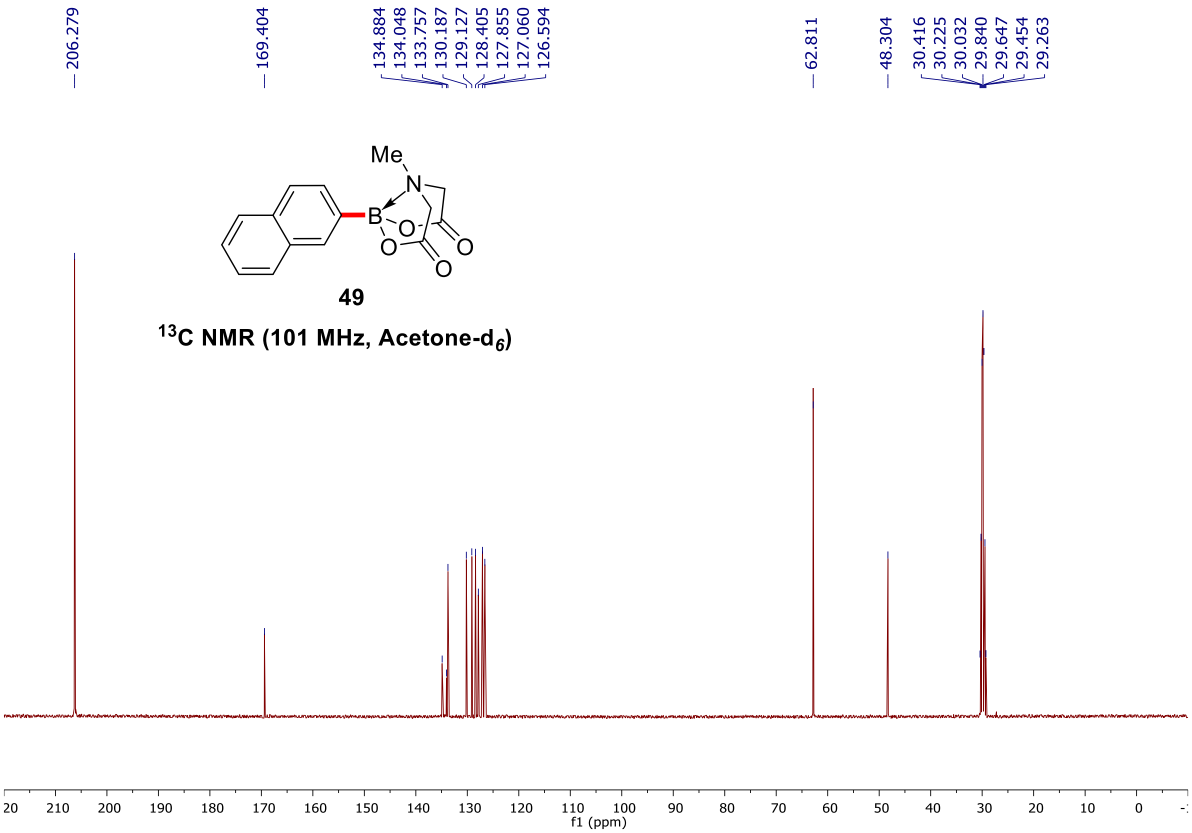

49

^{13}C NMR (101 MHz, Acetone-d$_6$)

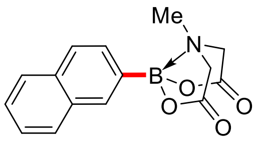

49

^{11}B NMR (128 MHz, Acetone-d$_6$)

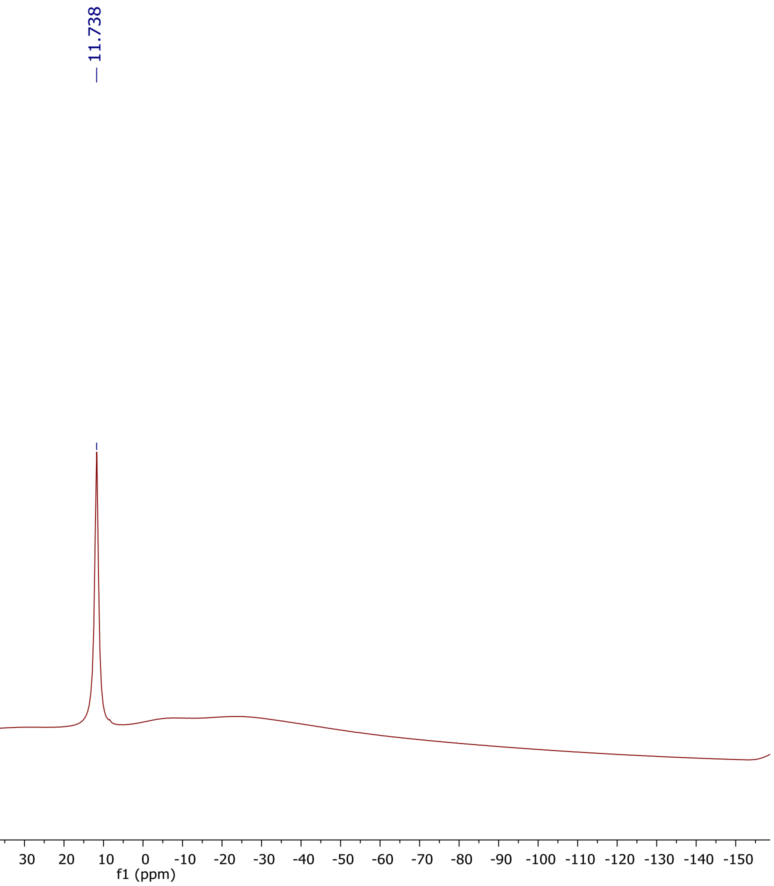

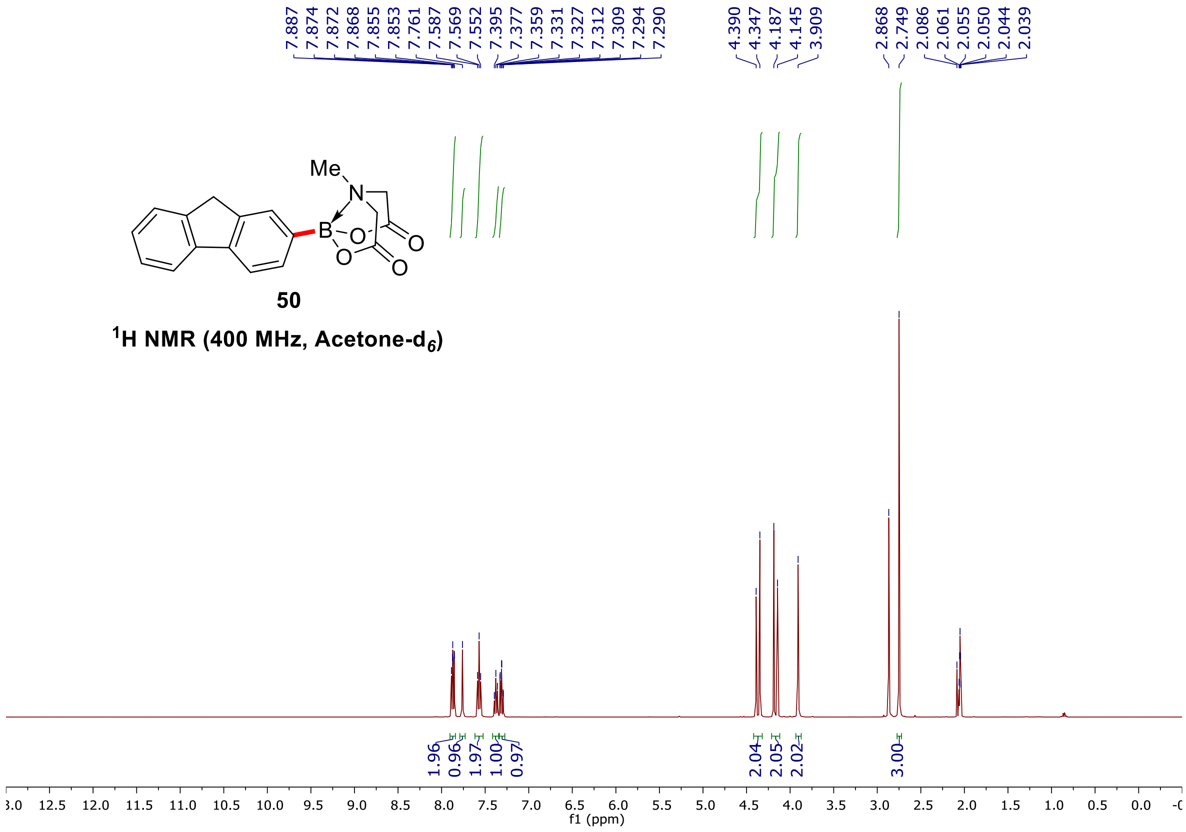
7.887 7.874 7.872 7.868 7.855 7.853 7.761 7.587 7.569 7.552 7.395 7.377 7.359 7.331 7.327 7.312 7.309 7.294 7.290
4.390 4.347 4.187 4.145 3.909
2.868 2.749 2.086 2.061 2.055 2.050 2.044 2.039
Me
N
B
O
O
O
O
50
1H NMR (400 MHz, Acetone-d6)
1.96 0.96 1.97 1.00 0.97
2.04 2.05 2.02
3.00
f1 (ppm)

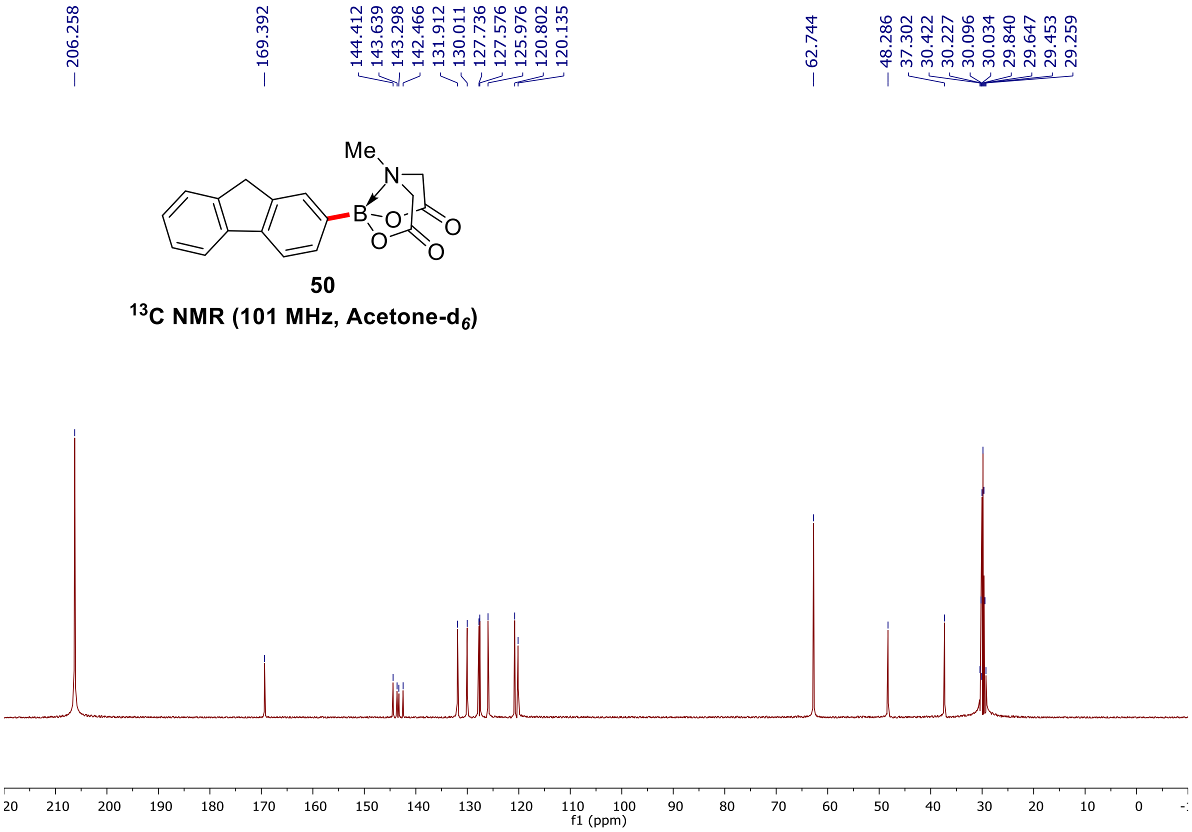
206.258
169.392
144.412 143.639 143.298 142.466 131.912 130.011 127.736 127.576 125.976 120.802 120.135
62.744
48.286 37.302 30.422 30.227 30.096 30.034 29.840 29.647 29.453 29.259
Me
N
B
O
O
O
O
50
13C NMR (101 MHz, Acetone-d6)
f1 (ppm)

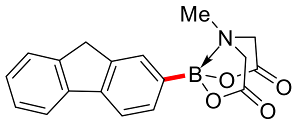

50

^{11}B NMR (128 MHz, Acetone-d_6)

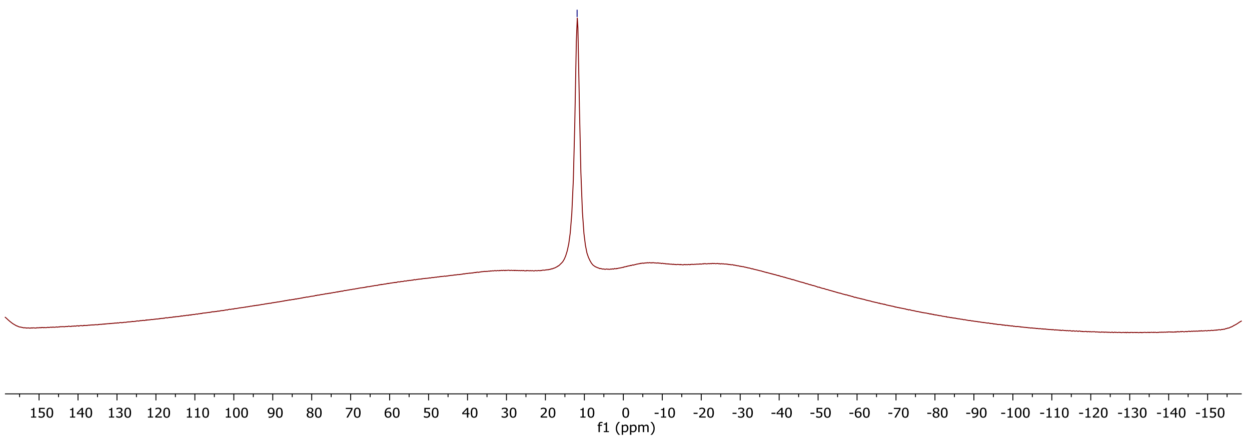

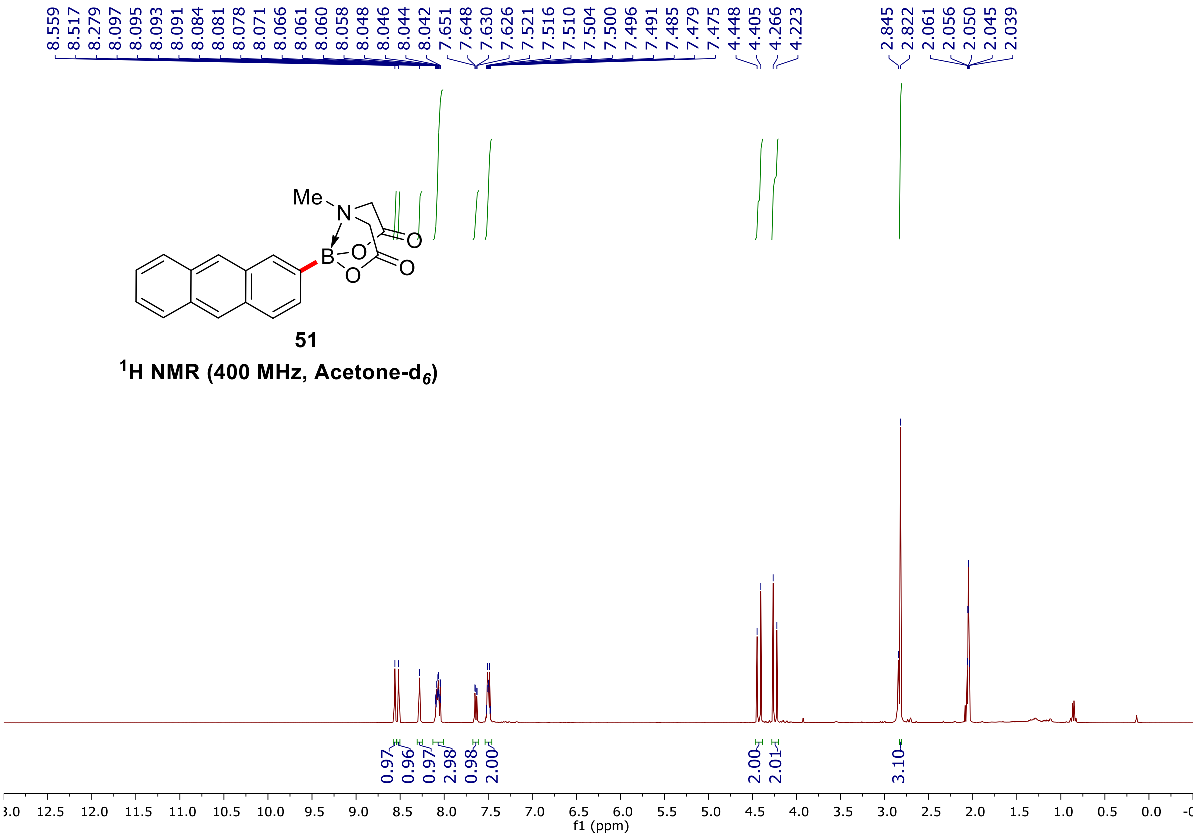
8.559
8.517
8.279
8.097
8.095
8.093
8.091
8.084
8.081
8.078
8.071
8.066
8.061
8.060
8.058
8.048
8.046
8.044
8.042
7.651
7.648
7.630
7.626
7.521
7.516
7.510
7.504
7.500
7.496
7.491
7.485
7.479
7.475
4.448
4.405
4.266
4.223
2.845
2.822
2.061
2.056
2.050
2.045
2.039
Me
N
B
O
O
O
O
51
^{1}H NMR (400 MHz, Acetone-d$_6$)
0.97
0.96
0.97
2.98
0.98
2.00
2.00
2.01
3.10
f1 (ppm)

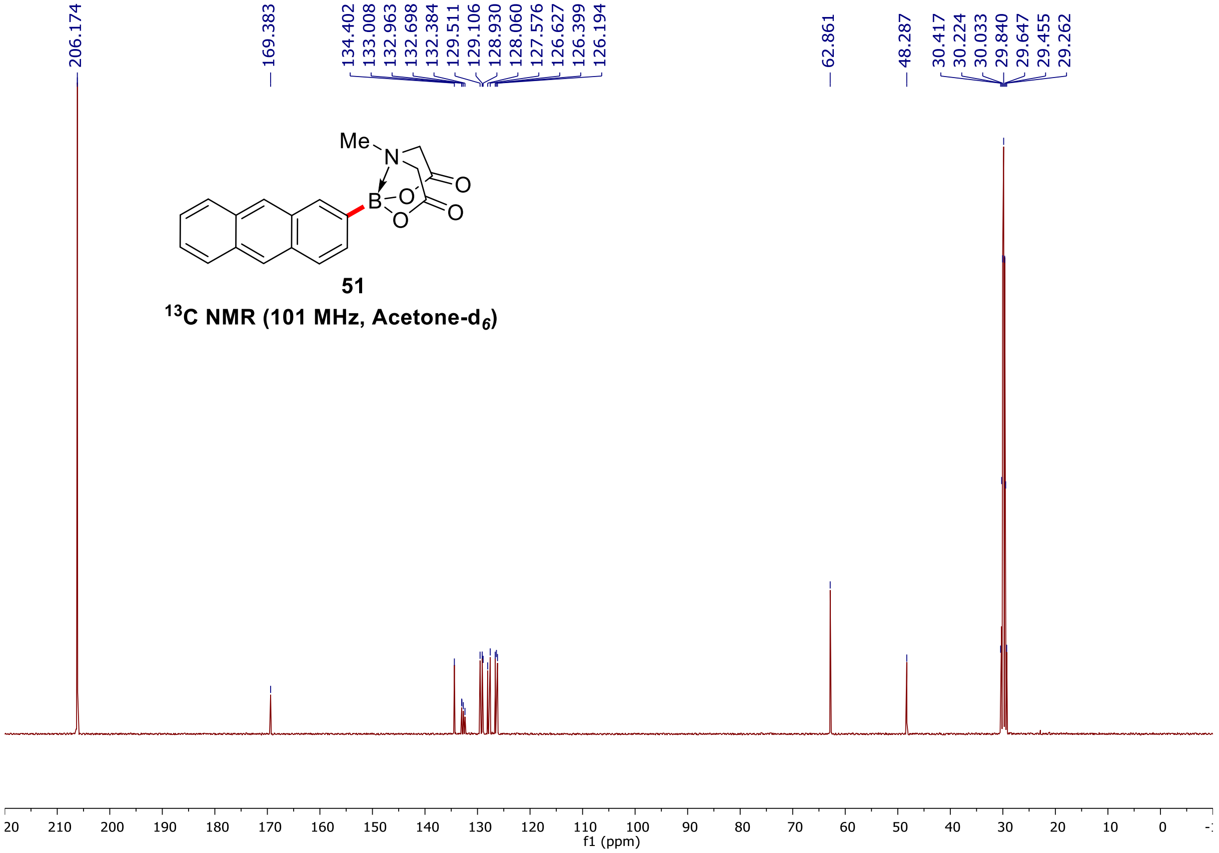
206.174
169.383
134.402
133.008
132.963
132.698
132.384
129.511
129.106
128.930
128.060
127.576
126.627
126.399
126.194
62.861
48.287
30.417
30.224
30.033
29.840
29.647
29.455
29.262
Me
N
B
O
O
O
O
51
^{13}C NMR (101 MHz, Acetone-d$_6$)
f1 (ppm)

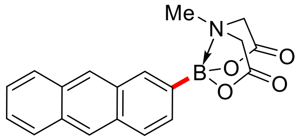

51

^{11}B NMR (128 MHz, Acetone-d_6)

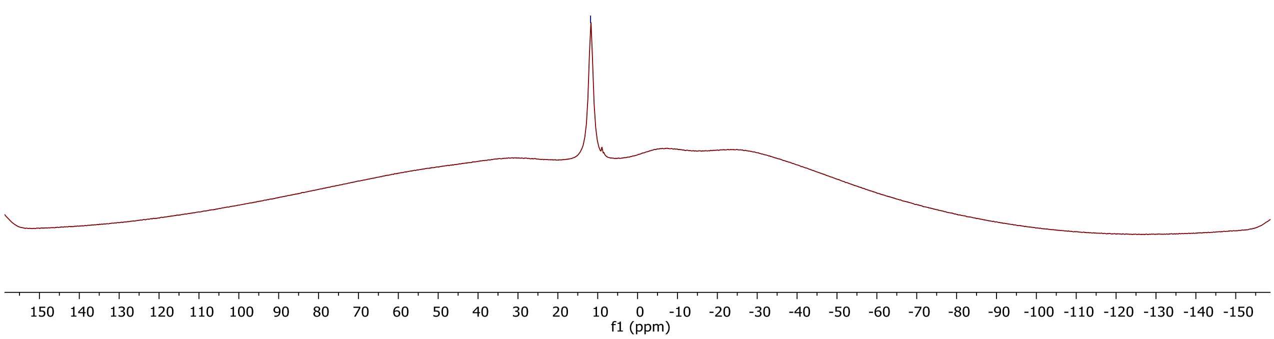

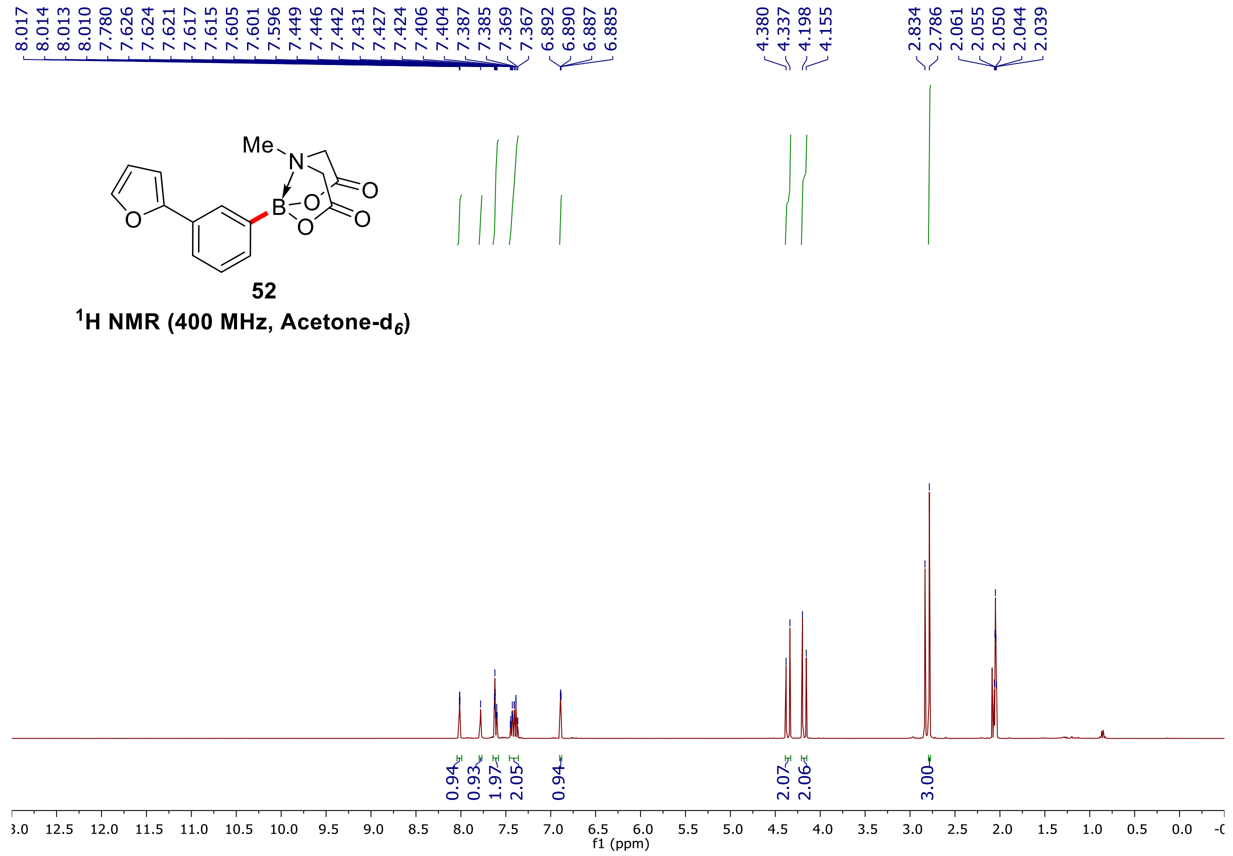
8.017 8.014 8.013 8.010 7.780 7.626 7.624 7.621 7.617 7.615 7.605 7.601 7.596 7.449 7.446 7.442 7.431 7.427 7.424 7.406 7.404 7.387 7.385 7.369 7.367 6.892 6.890 6.887 6.885
4.380 4.337 4.198 4.155
2.834 2.786 2.061 2.055 2.050 2.044 2.039
Me
N
B
O
52
^{1}H NMR (400 MHz, Acetone-d$_6$)
0.94 0.93 1.97 2.05 0.94
2.07 2.06
3.00
f1 (ppm)

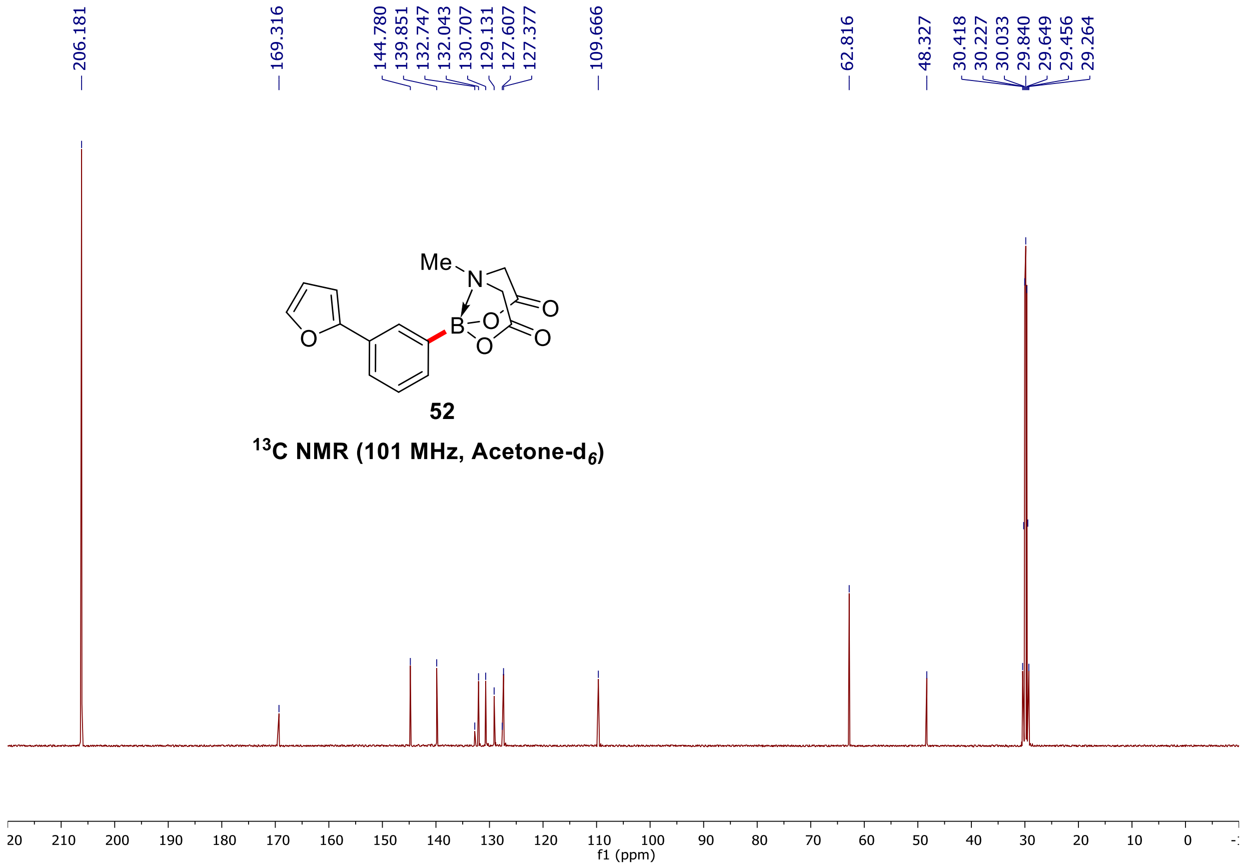
206.181
169.316
144.780 139.851 132.747 132.043 130.707 129.131 127.607 127.377
109.666
62.816
48.327
30.418 30.227 30.033 29.840 29.649 29.456 29.264
Me
N
B
O
52
^{13}C NMR (101 MHz, Acetone-d$_6$)
f1 (ppm)

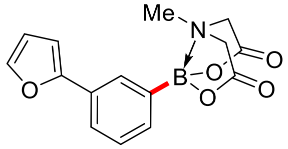

52

^{11}B NMR (128 MHz, Acetone-d$_6$)

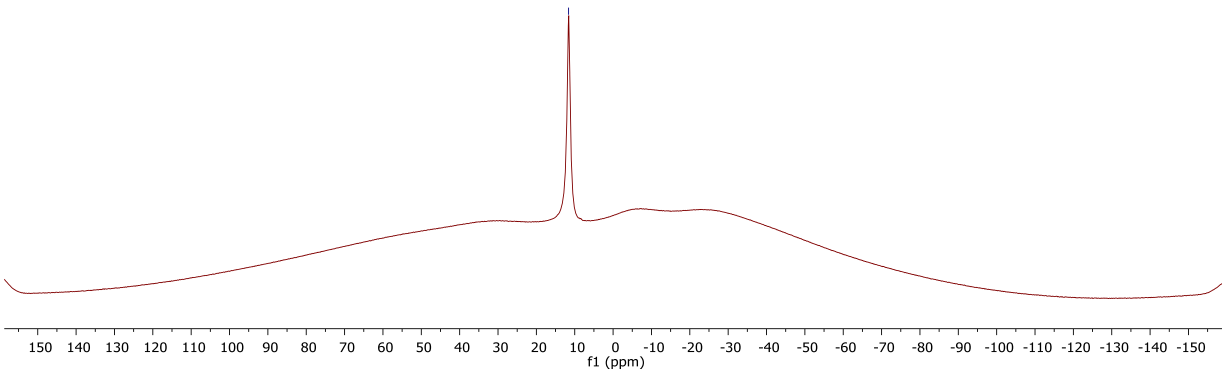

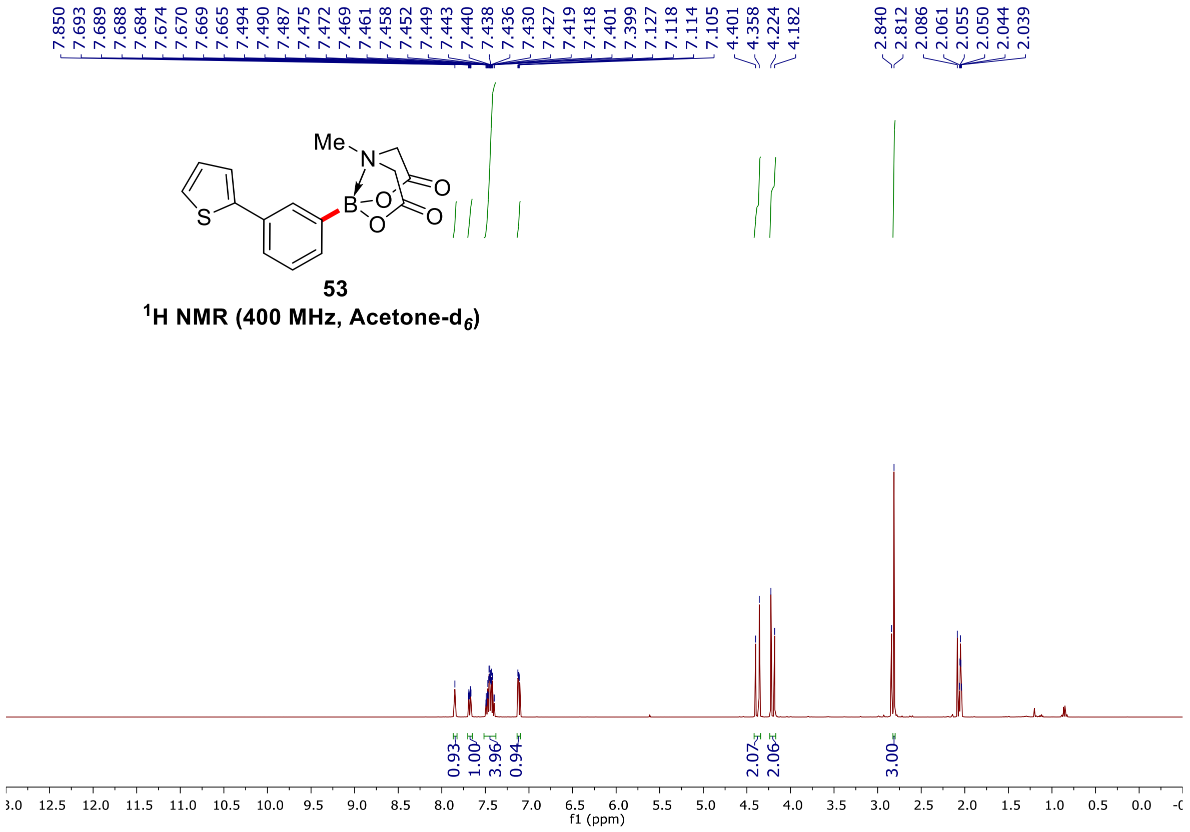
7.850 7.693 7.689 7.688 7.684 7.674 7.670 7.669 7.665 7.494 7.490 7.487 7.475 7.472 7.469 7.461 7.458 7.452 7.449 7.443 7.440 7.438 7.436 7.430 7.427 7.419 7.418 7.401 7.399 7.127 7.118 7.114 7.105 4.401 4.358 4.224 4.182 2.840 2.812 2.086 2.061 2.055 2.050 2.044 2.039
Me
N
B
O
O
O
O
S
53
1H NMR (400 MHz, Acetone-d6)
0.93 1.00 3.96 0.94 2.07 2.06 3.00
f1 (ppm)

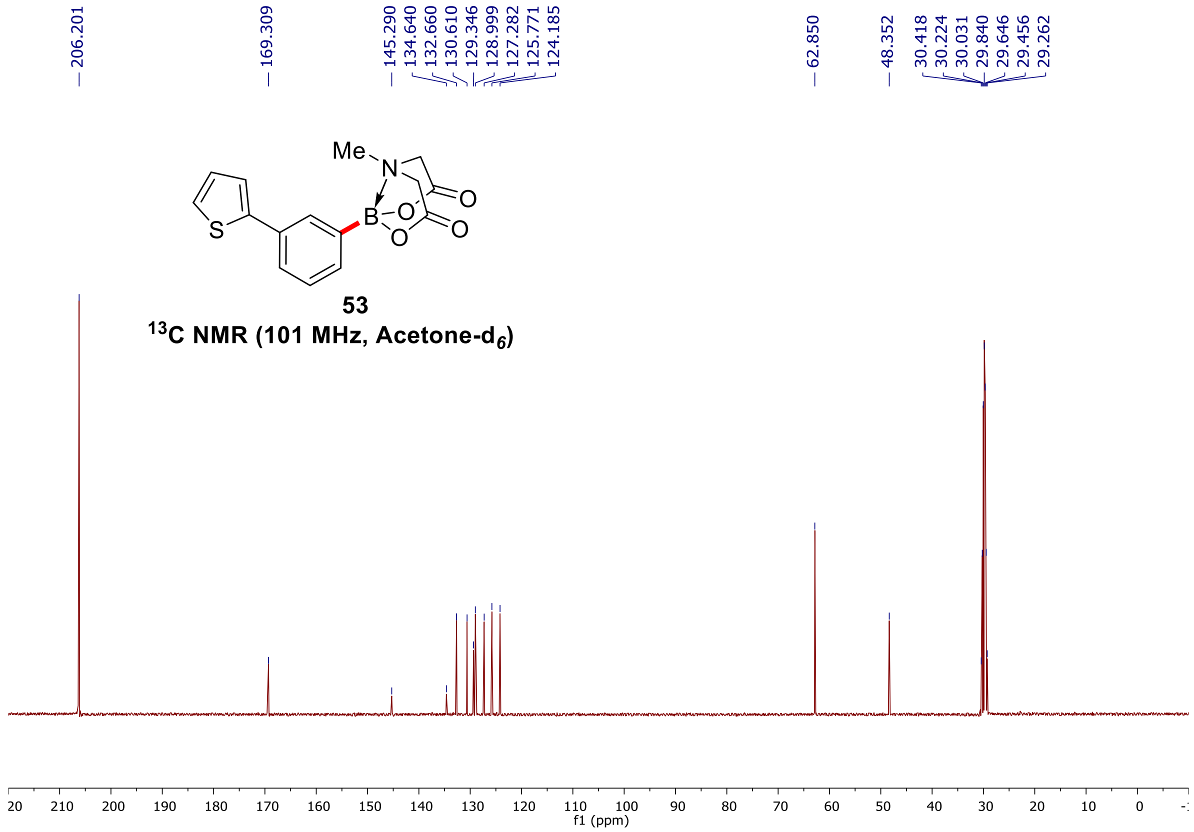
206.201 169.309 145.290 134.640 132.660 130.610 129.346 128.999 127.282 125.771 124.185 62.850 48.352 30.418 30.224 30.031 29.840 29.646 29.456 29.262
Me
N
B
O
O
O
O
S
53
13C NMR (101 MHz, Acetone-d6)
f1 (ppm)

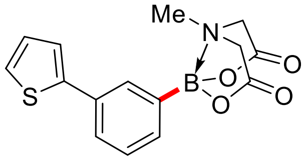

53

^{11}B NMR (128 MHz, Acetone-d_6)

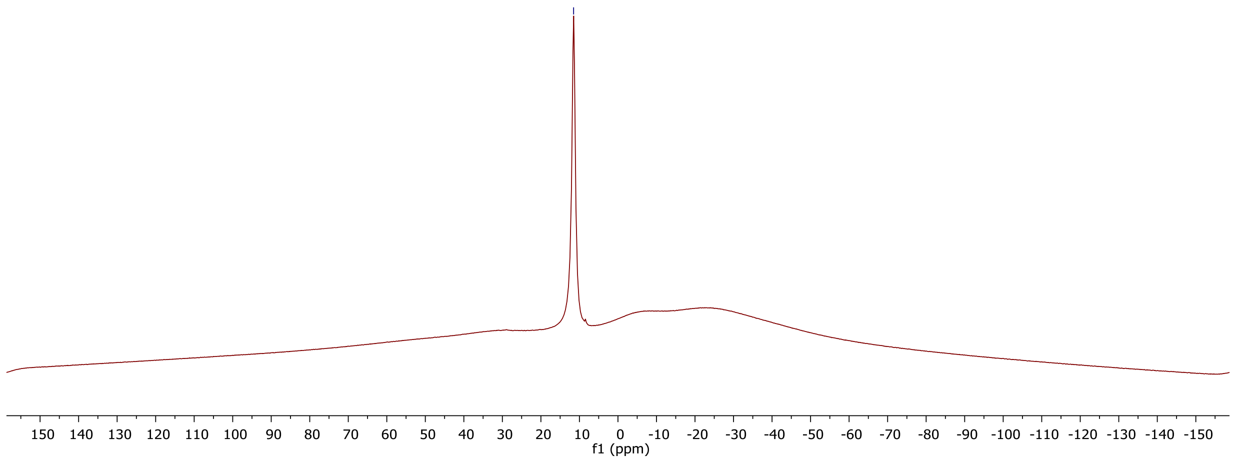

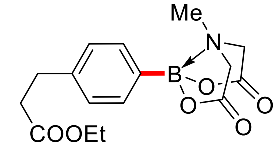

54

^{1}H NMR (400 MHz, DMSO-d$_6$)

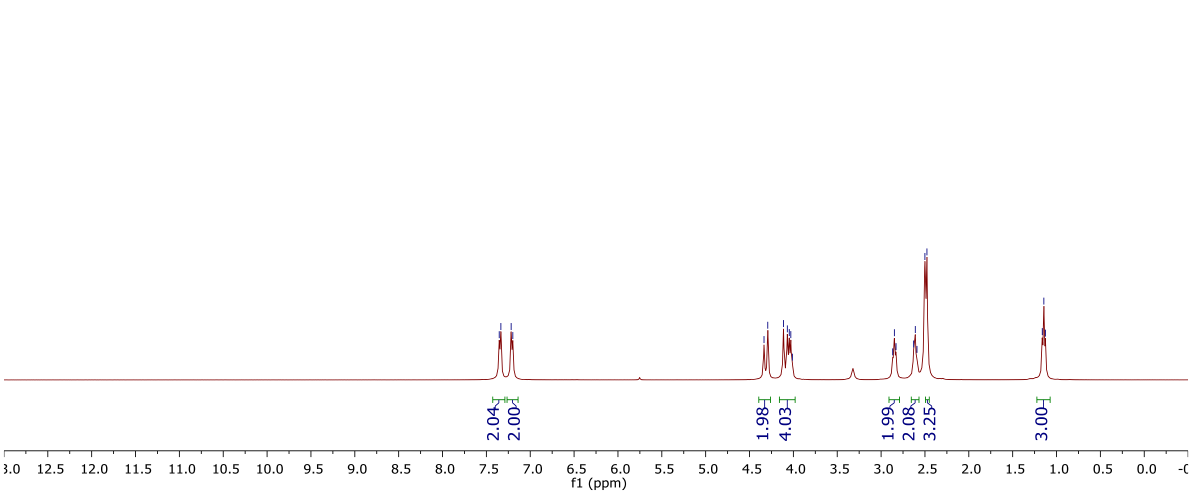

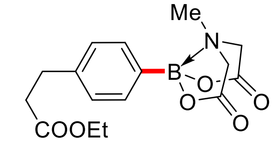

54

^{13}C NMR (101 MHz, DMSO-d$_6$)

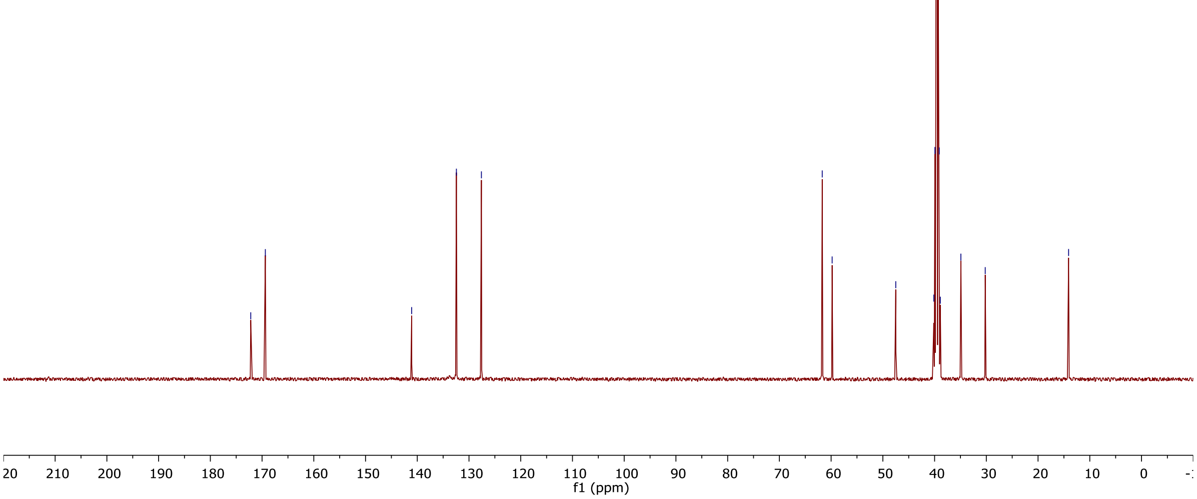

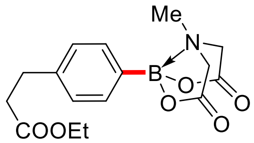

54

^{11}B NMR (128 MHz, DMSO-d_6)

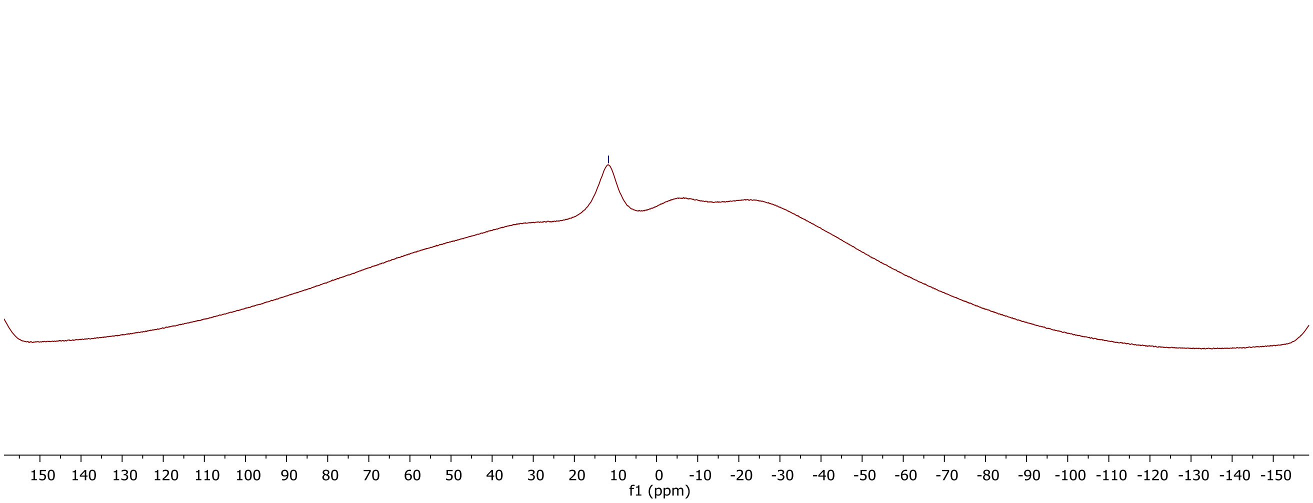

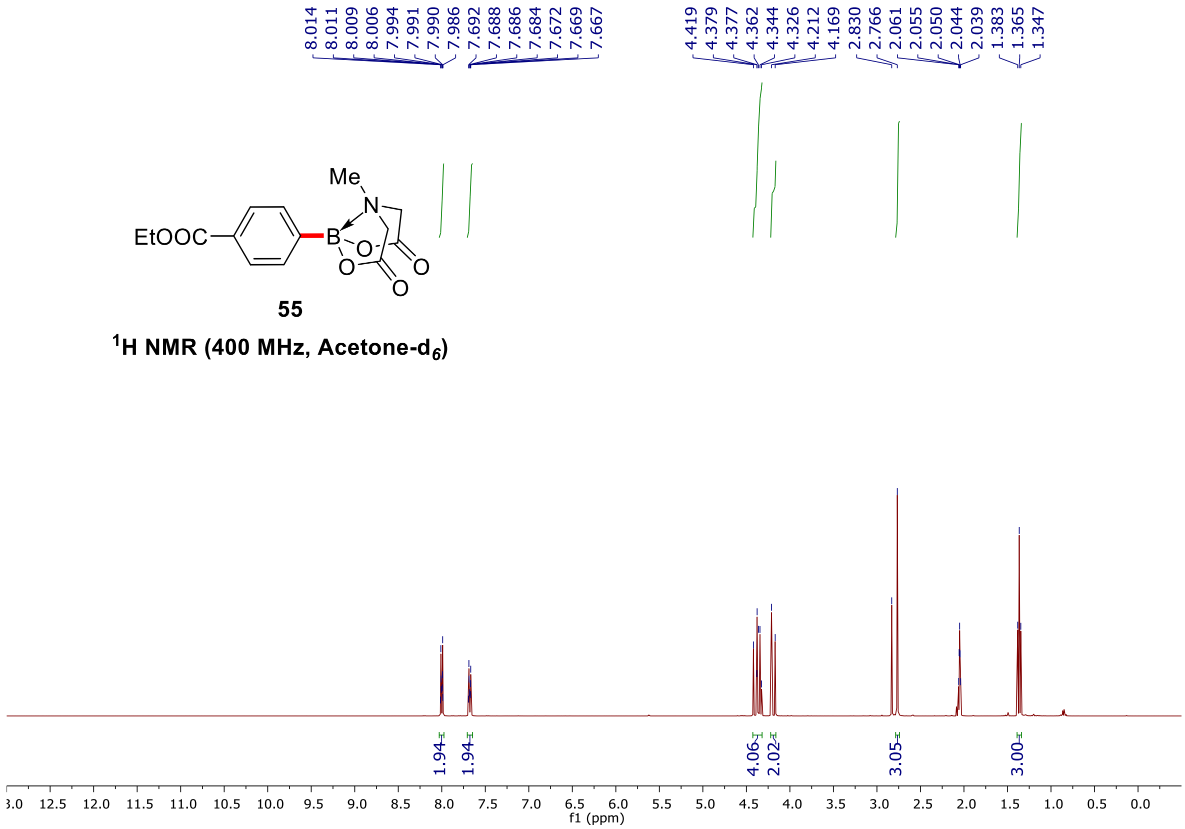

55

^{1}H NMR (400 MHz, Acetone-d_6)

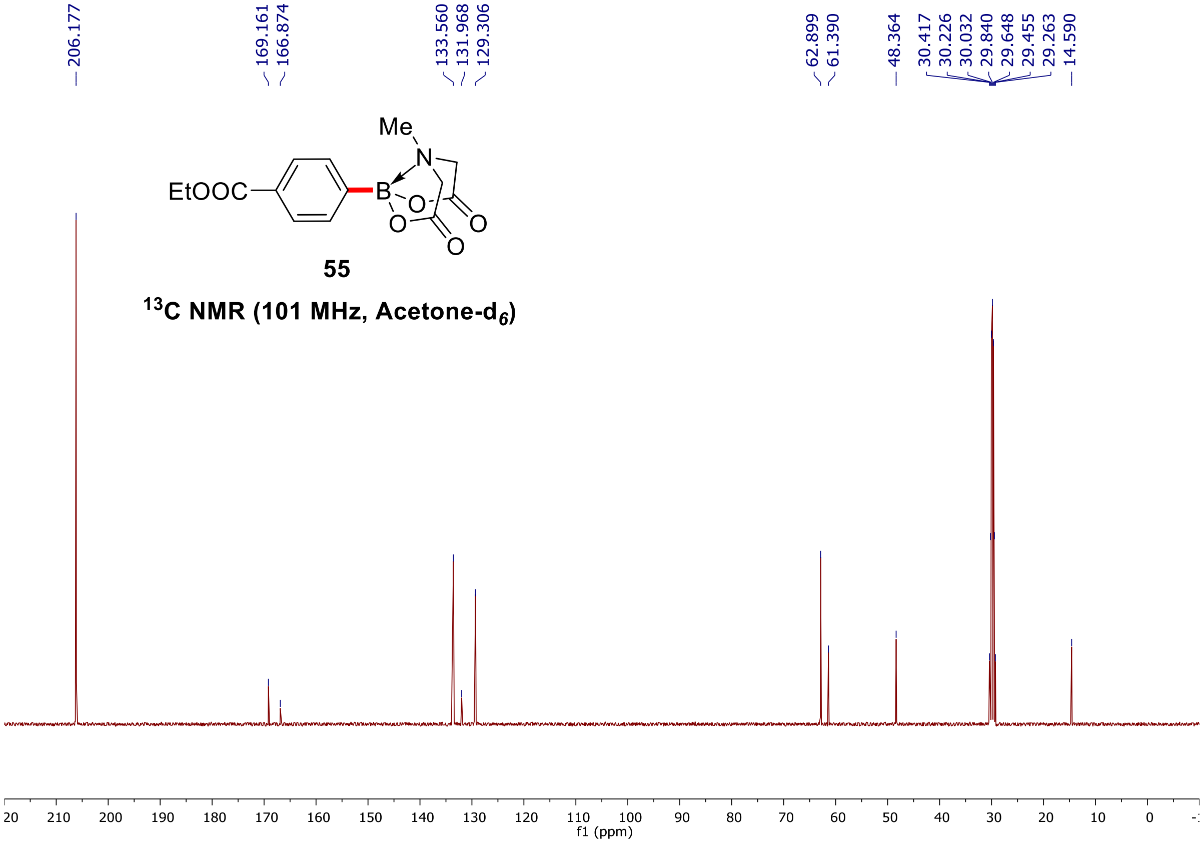

55

^{13}C NMR (101 MHz, Acetone-d_6)

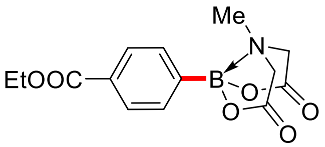

55

^{11}B NMR (128 MHz, Acetone-d_6)

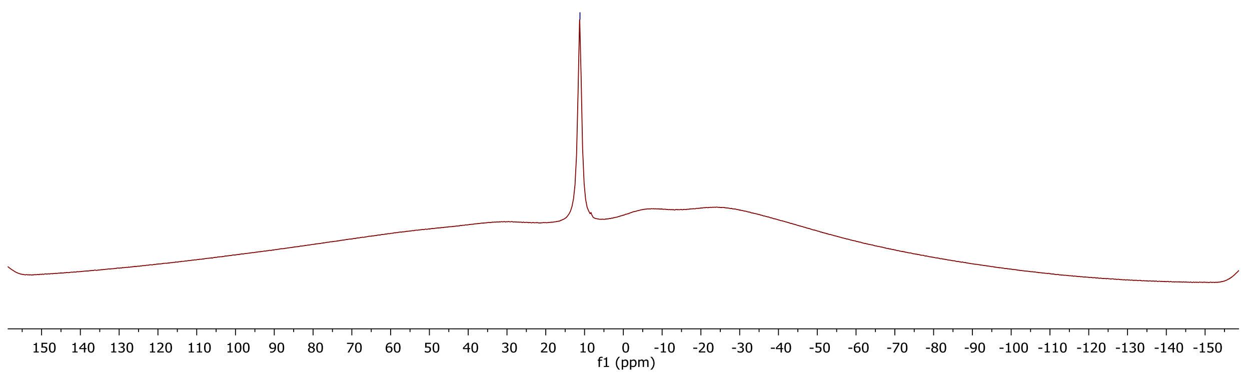

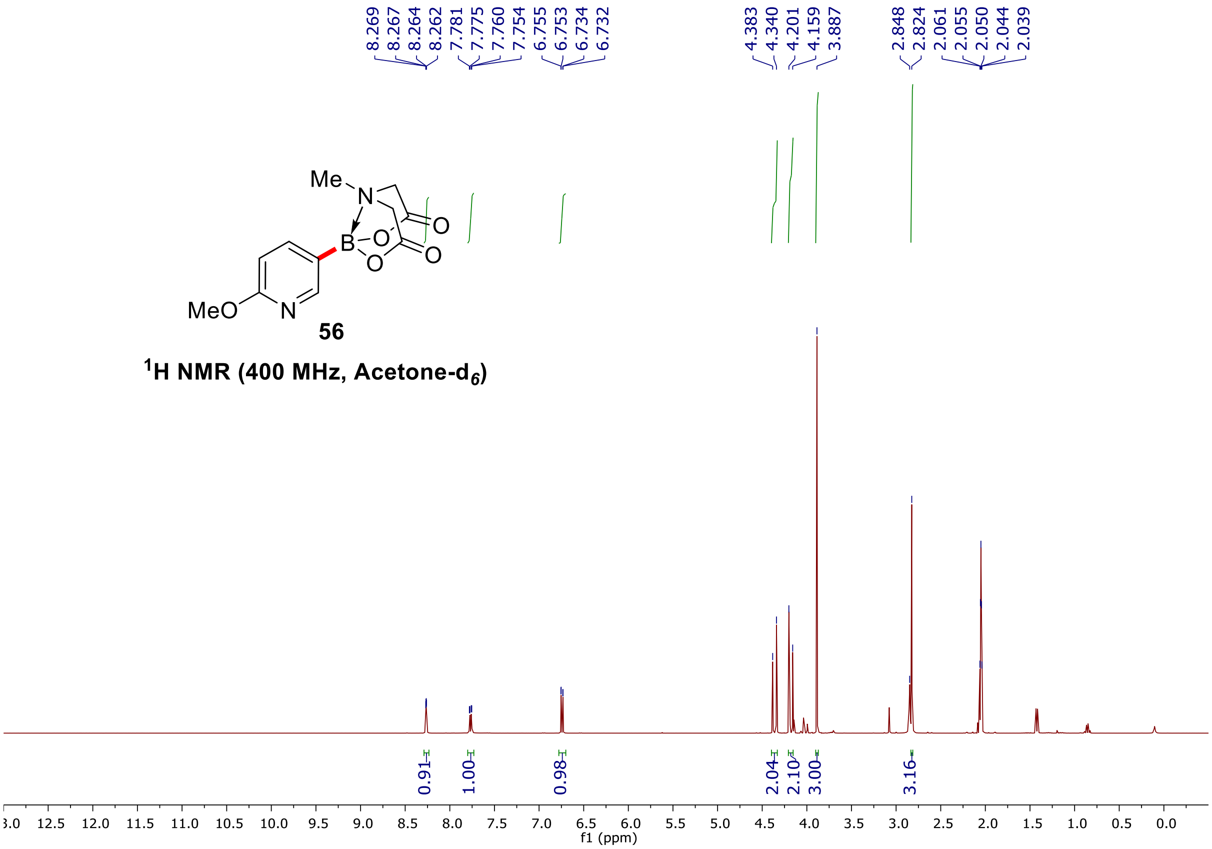

8.269
8.267
8.264
8.262
7.781
7.775
7.760
7.754
6.755
6.753
6.734
6.732
4.383
4.340
4.201
4.159
3.887
2.848
2.824
2.061
2.055
2.050
2.044
2.039
Me
N
B
O
O
O
O
MeO
N
56
¹H NMR (400 MHz, Acetone-d6)
0.91
1.00
0.98
2.04
2.10
3.00
3.16
f1 (ppm)

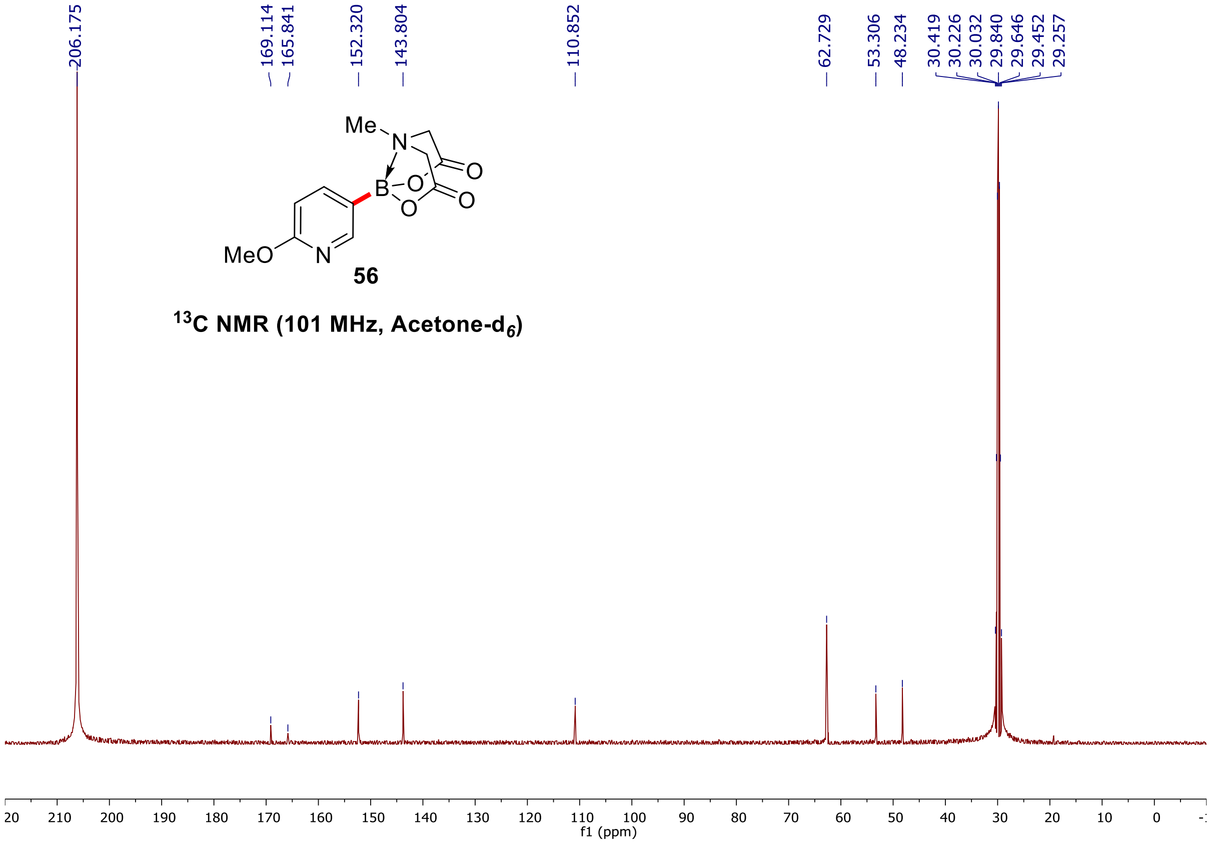

206.175
169.114
165.841
152.320
143.804
110.852
62.729
53.306
48.234
30.419
30.226
30.032
29.840
29.646
29.452
29.257
Me
N
B
O
O
O
O
MeO
N
56
¹³C NMR (101 MHz, Acetone-d6)
f1 (ppm)

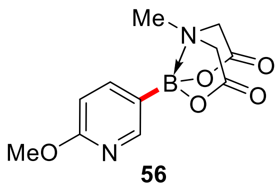

56

^{11}B NMR (128 MHz, Acetone-d$_6$)

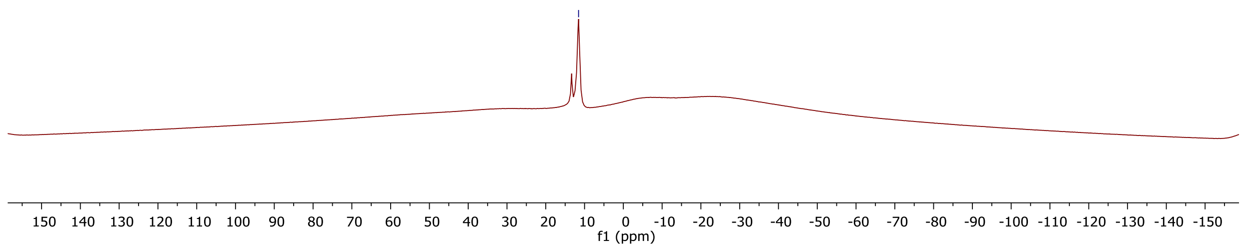

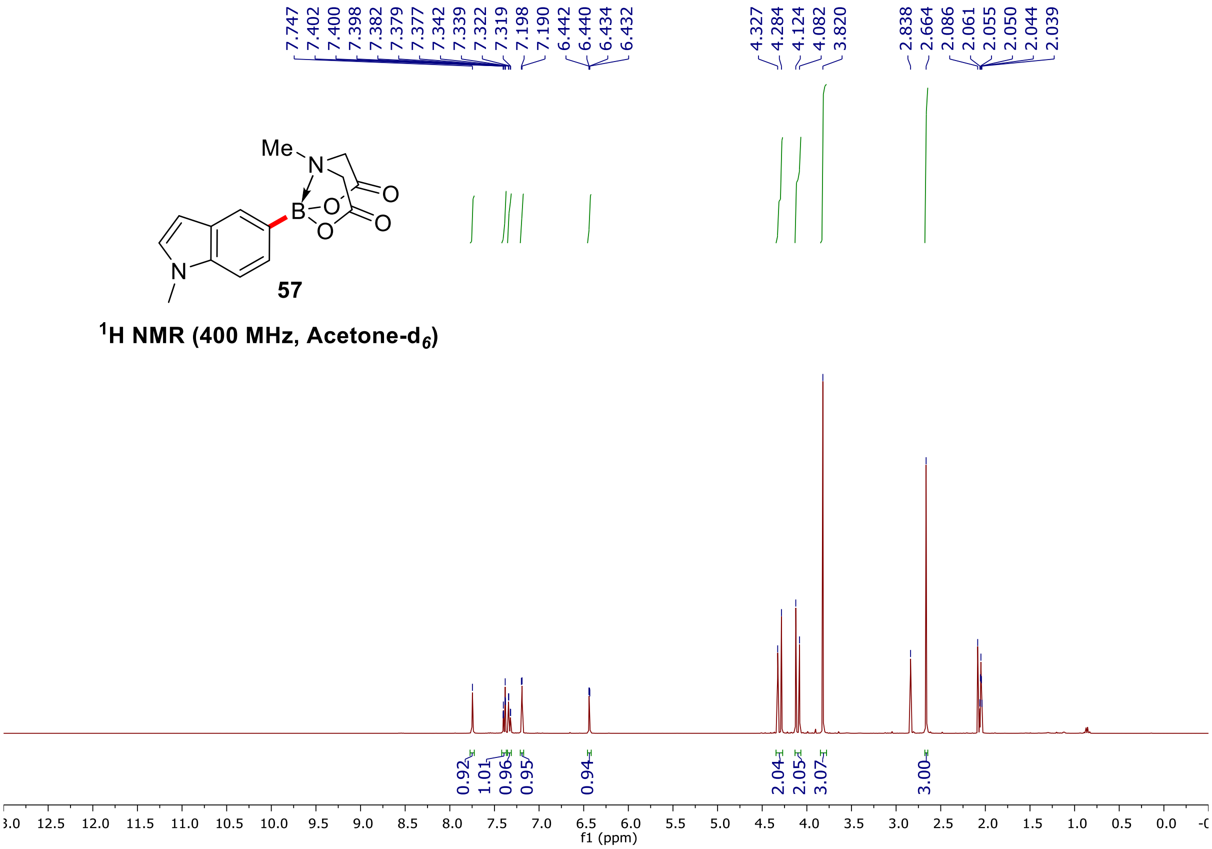

7.747
7.402
7.400
7.398
7.382
7.379
7.377
7.342
7.339
7.322
7.319
7.198
7.190
6.442
6.440
6.434
6.432
4.327
4.284
4.124
4.082
3.820
2.838
2.664
2.086
2.061
2.055
2.050
2.044
2.039
Me
N
B
O
O
O
O
N
57
^{1}H NMR (400 MHz, Acetone-d$_6$)
0.92
1.01
0.96
0.95
0.94
2.04
2.05
3.07
3.00
f1 (ppm)

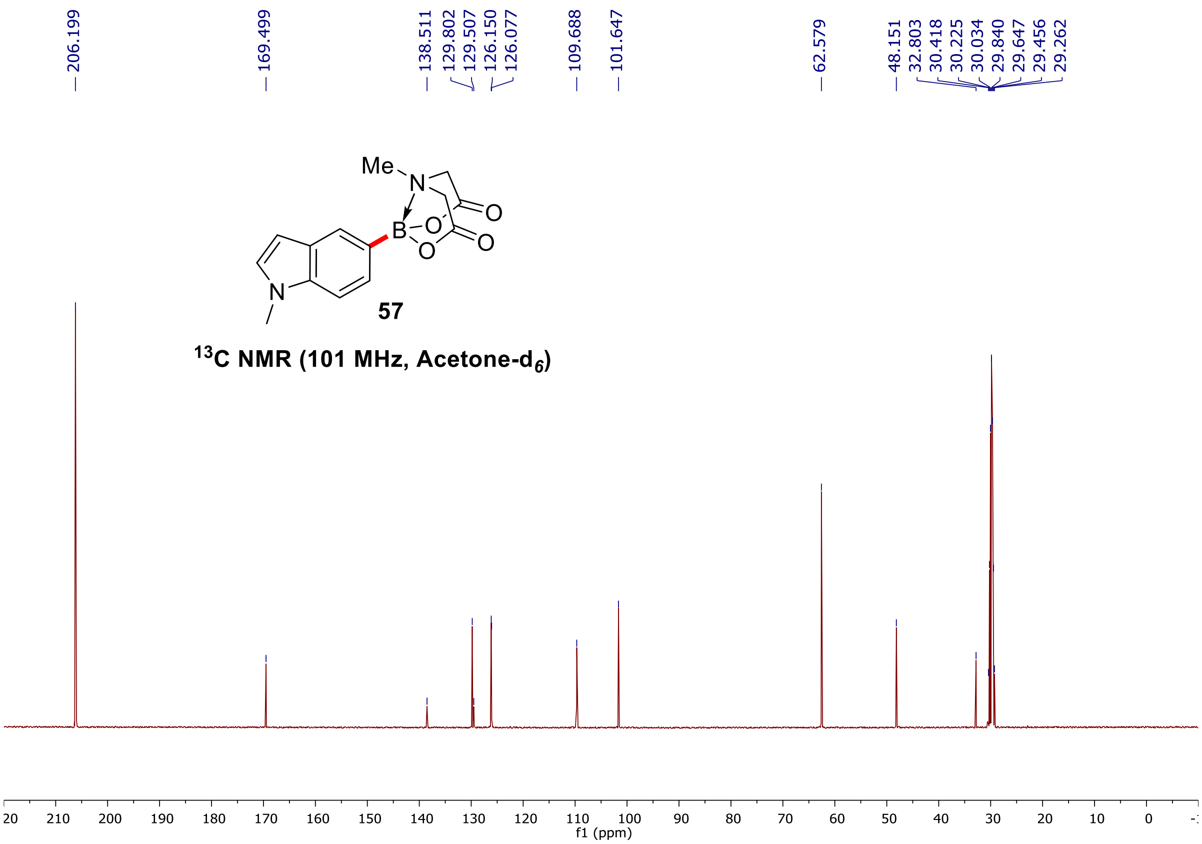

206.199
169.499
138.511
129.802
129.507
126.150
126.077
109.688
101.647
62.579
48.151
32.803
30.418
30.225
30.034
29.840
29.647
29.456
29.262
Me
N
B
O
O
O
O
N
57
^{13}C NMR (101 MHz, Acetone-d$_6$)
f1 (ppm)

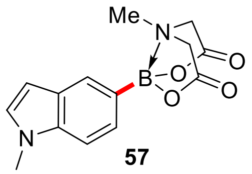

57

^{11}B NMR (128 MHz, Acetone-d$_6$)

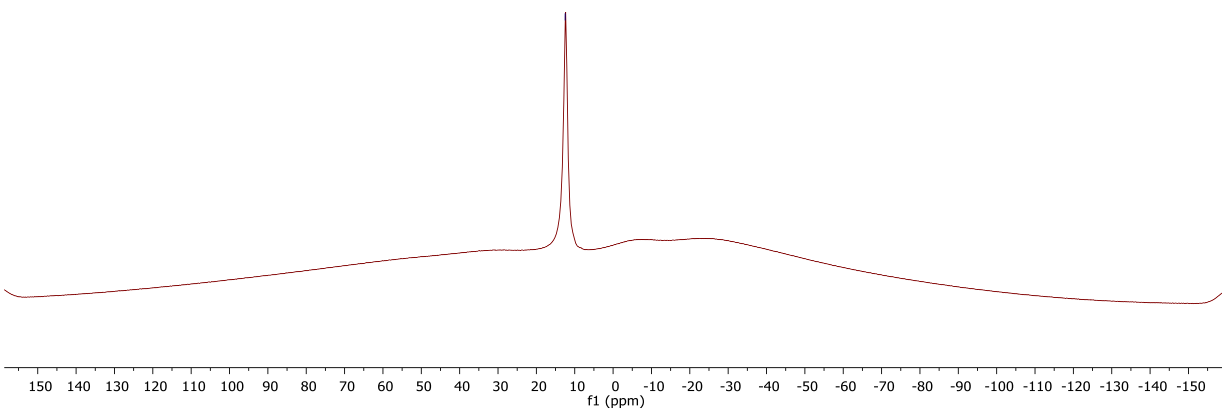

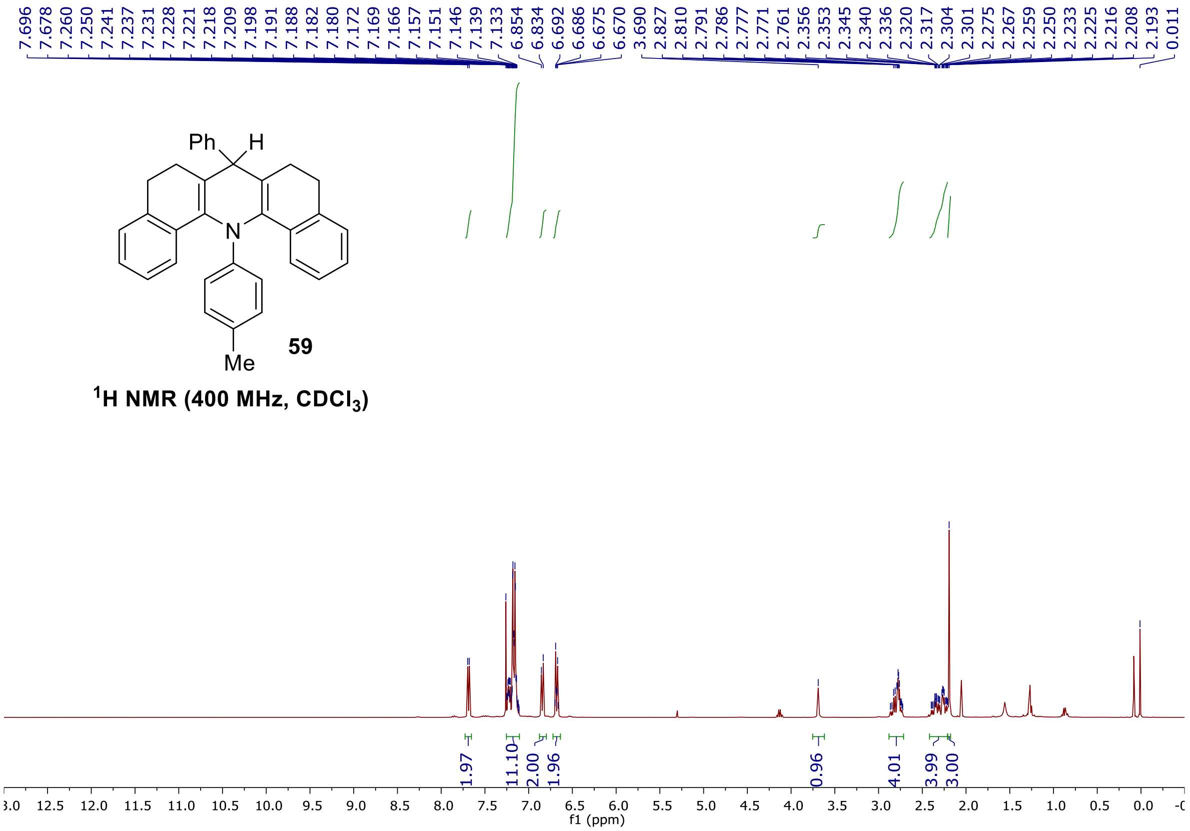

59

^{1}H NMR (400 MHz, $CDCl_3$)

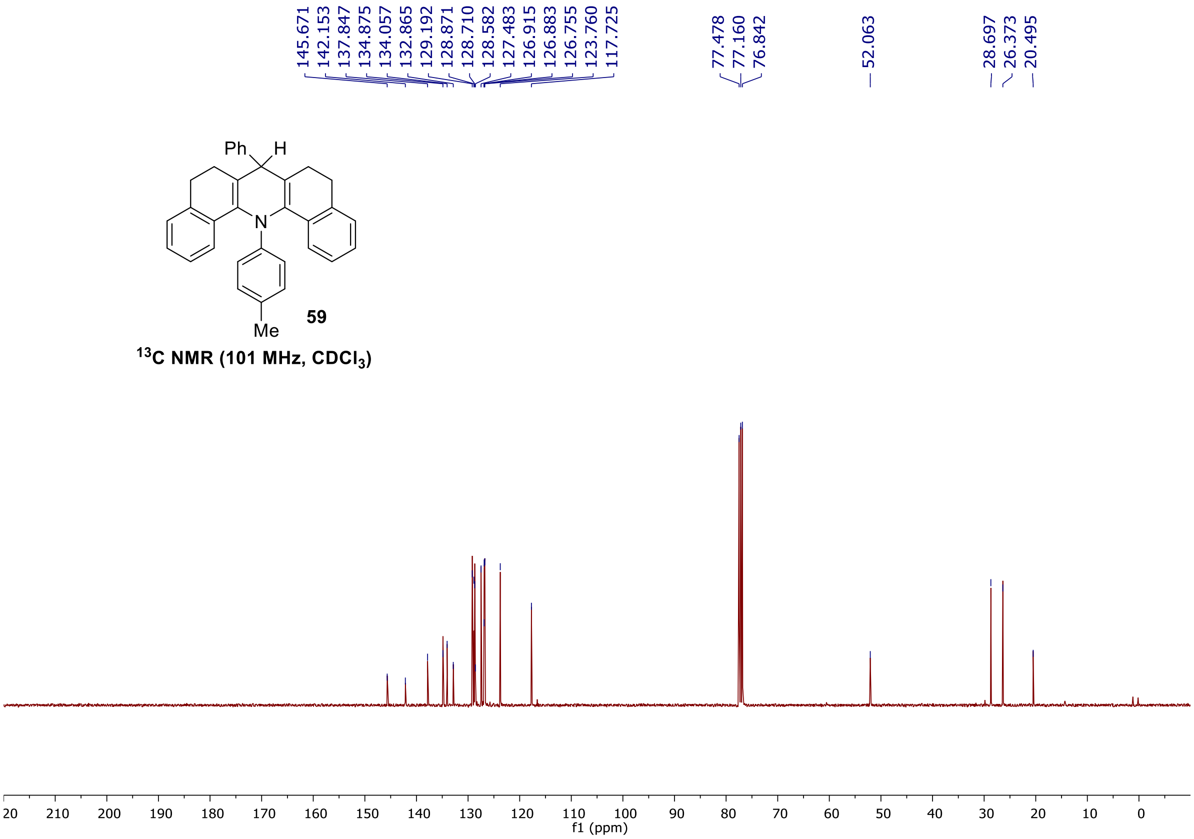

59

^{13}C NMR (101 MHz, $CDCl_3$)
